# Supplementary material for: Tetrafluoroisopropylation of alkenes and alkynes enabled by photocatalytic consecutive difluoromethylation with CF2HSO2Na
Source: Nat Commun. 2024 Jul 6;15:5685. doi: 10.1038/s41467-024-50081-x (PMC11227567; doi:10.1038/s41467-024-50081-x)
Supplement: Supplementary file 1 — Supplementary Information [file 41467_2024_50081_MOESM1_ESM.pdf]

Supplementary Information to

Tetrafluoroisopropylation of alkenes and alkynes enabled by photocatalytic consecutive difluoromethylation with CF_2HSO_2Na

Yuwei Hong,[‡] Jiayan Qiu,[‡] Zhenzhen Wu, Sangxuan Xu, Hanliang Zheng,* and Gangguo Zhu*

Key Laboratory of the Ministry of Education for Advanced Catalysis Materials, College of Chemistry and Materials Science, Zhejiang Normal University, 688 Yingbin Road, Jinhua 321004, China. Email: hanliang@zjnu.edu.cn, gangguo@zjnu.cn

Table of contents

1. General information……………………………..…………………..................................…3

2. Optimization of the photocatalytic carbotetrafluoroisopropylation of alkynyl ketones……………………………...…………………………………..…………...…...…...…..4

3. Information for starting materials...…5

4. General procedures for experiments and analytical data……………………………………....6

4.1 General procedures and analytical data for hydrotetrafluoroisopropylation of alkenes…………………..………………………………………………….......…..……...…..6

4.2 General procedures and analytical data for carbotetrafluoroisopropylation of alkynes…………………………………………………………………………...……....…..21

5. Mechanistic experiments……………...…………………….………………………….......34

6. Evaluation of PPARα transactivation activities.……………….…………………….….......39

7. NMR spectra...…………...………………………...……………………..................…....40

8. X-Ray crystallographic data...…………………………………………………………........131

9. Supplementary references...……………………………………………………….............133

1. General information

Unless otherwise noted, materials obtained from commercial suppliers were used directly without further purification. Melting points reported were measured by a melting point instrument and were uncorrected. ^{1}H, ^{13}C, and ^{19}F NMR spectra were measured on a 600 or 400 MHz NMR spectrometer using $CDCl_3$ as the solvent with tetramethylsilane (TMS) as the internal standard. Chemical shifts (δ) are given in parts per million relative to TMS or the residual of solvent signal (TMS, δ_H = 0.00 ppm; $CDCl_3$, δ_H = 7.26 ppm, δ_C = 77.0 ppm), and the coupling constants are given in hertz. The following abbreviations were used to explain the multiplicities: s = singlet, d = doublet, t = triplet, q = quartet, m = multiplet. High-resolution mass spectrometry (HRMS) analyses were carried out using a TOF MS instrument with ESI source. GC-MS measurements were performed on a 7890B GC system with an Agilent 5975 MSD detector. Column chromatography was performed using silica gel (200-300 mesh). PE = petroleum ethers; EtOAc = ethyl acetate.

Photochemical reactions were performed by placing reaction vessel on the 24 W photoreactor (455-460 nm) shown as below.

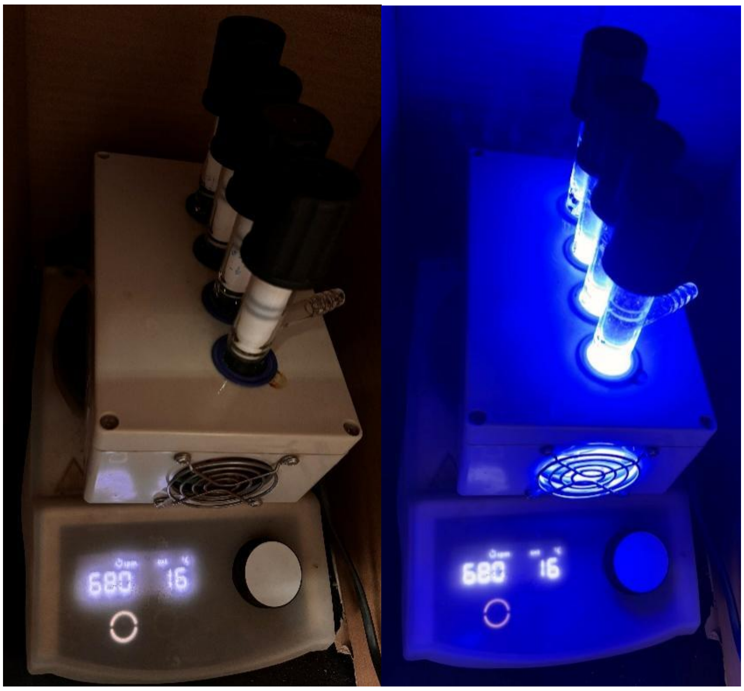

Supplementary Figure 1 The reaction set up apparatus.

2. Optimization of the photocatalytic carbotetrafluoroisopropylation of alkynyl ketones

Supplementary Table 1 Optimization of conditions.[a]

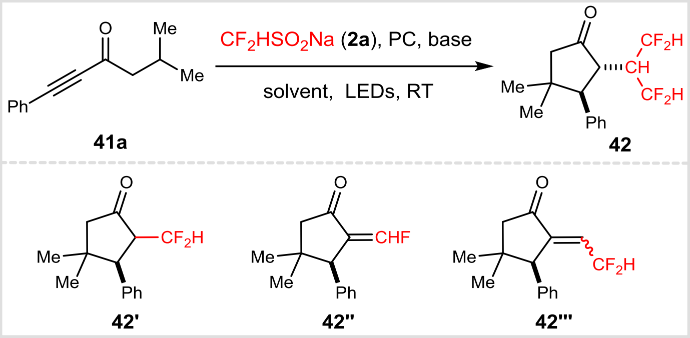

entry	PC	base	solvent	yield of **42/42'/42''/42'''** (%)[b]	dr[c]
1	4CzIPN	none	DMF	64/0/0/0	10:1
2	4CzIPN	none	THF	55/0/0/0	2:1
3	4CzIPN	none	DMSO	85/0/0/0	10:1
4	4CzIPN	none	MeCN	90/0/0/0	9:1
5	Eosin Y	none	MeCN	81/0/0/0	3:1
6	DCA	none	MeCN	48/12/0/13	6:1
7	4DPAIPN	none	MeCN	65/0/0/0	9:1
8	4CzIPN	LiOH	MeCN	83/0/0/0	6:1
9	4CzIPN	K_2CO_3	MeCN	93/0/0/0	8:1
10	4CzIPN	K_2HPO_4	MeCN	75/0/0/0	5:1
11	4CzIPN	Cs_2CO_3	MeCN	91(86)[d]/0/0/0	>20:1

[a]Reaction conditions: **41a** (0.2 mmol), **2a** (0.8 mmol), PC (2 mol %), base (0.4 mmol), solvent (2 mL), 25 °C, 24 W blue LEDs, 18 h. [b]The ^{19}F NMR yield with *para*-fluoroiodobenzene as the internal standard. [c]Determined by the ^{19}F NMR analysis. [d]Isolated yield.

3. Information for starting materials

Alkenes **1a-1x** were purchased from suppliers and used without purification, others functionalized alkenes **1y-1zh** were synthesized according to the reported method and the resultant data are matched with the literature.[1] Ynones **41a-41v** were synthesized according to the reported method and the resultant data are matched with the literature.[2]

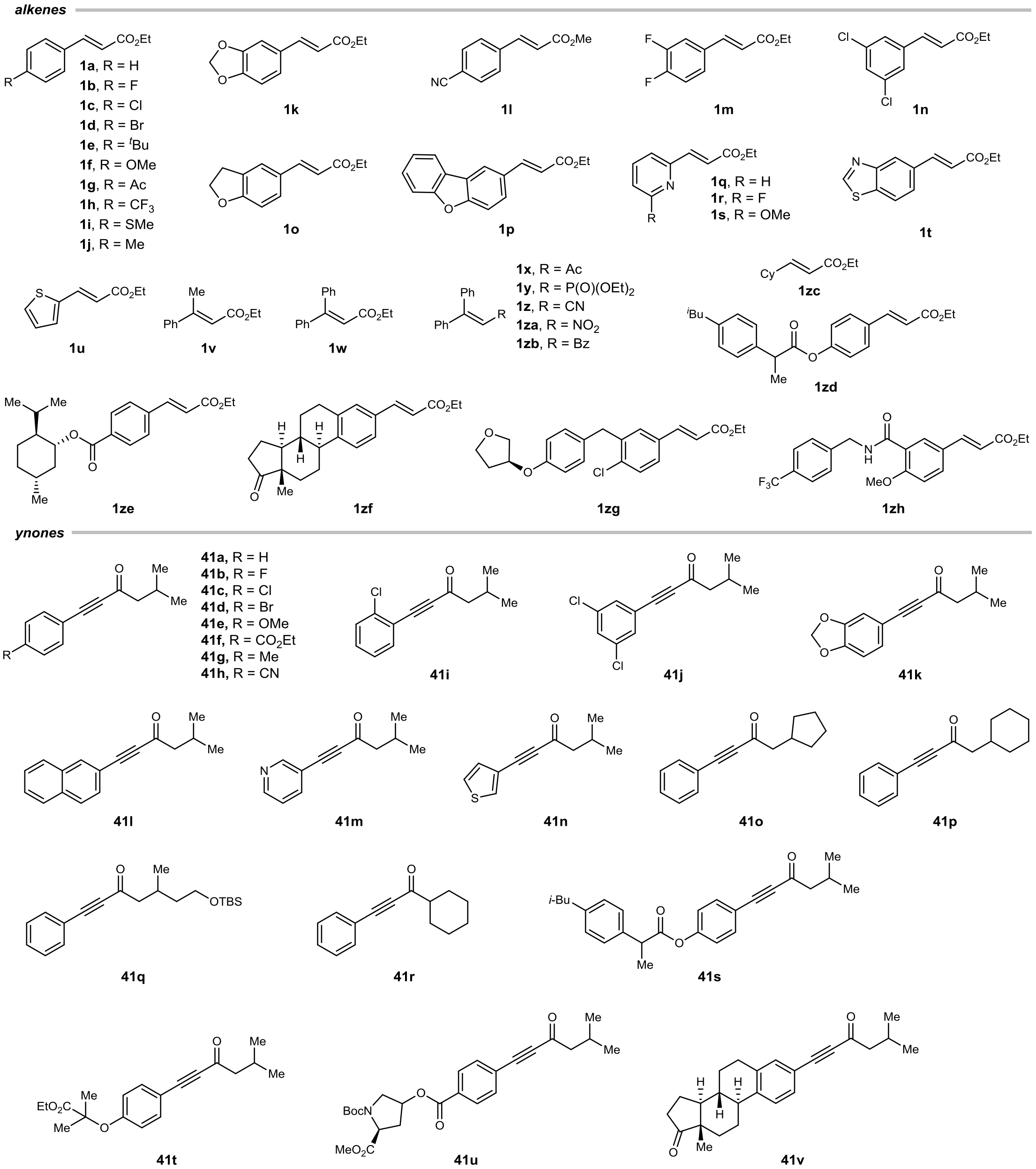

Supplementary Figure 2 Substrates examined in this work.

4. General procedures for experiments and analytical data

4.1 General procedures and analytical data for hydrotetrafluoroisopropylation of Alkenes

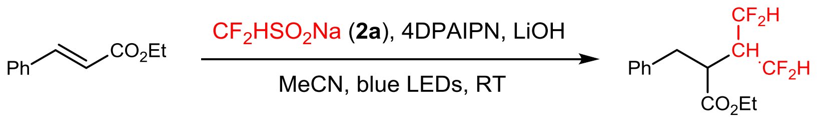

1a **3**

To a mixture of CF_2HSO_2Na (112 mg, 0.8 mmol), 4DPAIPN (15.9 mg, 0.02 mmol), H_2O (36.0 mg, 2.0 mmol) and LiOH (19.2 mg, 0.8 mmol) in 2 mL of MeCN was added **1a** (35.2 mg, 0.2 mmol) under a nitrogen atmosphere. After 24 h of irradiation at a distance of ~2 cm with 24 W of blue LEDs (PINO® lamps, 100% light intensity) at 25 °C, the reaction mixture was quenched with water, extracted with EtOAc, washed with brine, dried over anhydrous Na_2SO_4, and concentrated. Column chromatography on silica gel (PE/EtOAc = 50:1) gave 46 mg (79% yield) of **3** as a yellow oil. ^{1}H NMR (600 MHz, $CDCl_3$) δ 7.31–7.28 (m, 2H), 7.25–7.22 (m, 1H), 7.20–7.19 (m, 1H), 6.19 (td, J = 54.9, 4.6 Hz, 1H), 6.13 (td, J = 54.9, 4.6 Hz, 1H), 4.08–4.02 (m, 2H), 3.19 (ddd, J = 9.1, 7.0, 4.8 Hz, 1H), 3.10 (dd, J = 13.7, 9.1 Hz, 1H), 3.03 (dd, J = 13.7, 7.0 Hz, 1H), 2.70–2.65 (m, 1H), 1.11 (t, J = 7.2 Hz, 3H); ^{13}C NMR (151 MHz, $CDCl_3$) δ 172.3, 137.7, 129.0, 128.6, 126.9, 114.5 (tdd, J = 244.1, 7.8, 4.7 Hz), 114.3 (tt, J = 244.1, 7.3 Hz), 61.2, 47.9 (quintet, J = 19.9 Hz), 42.1 (quintet, J = 3.1 Hz), 35.7, 13.8; ^{19}F NMR (565 MHz, $CDCl_3$) δ -119.4 – -120.6 (m), -120.1 – -122.7 (m); HRMS (ESI) m/z: $[M + H]^+$ Calcd for $C_{14}H_{16}F_4O_2+H^+$: 293.1159; Found: 293.1160.

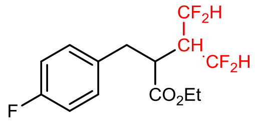

8

*Compound **8**:* 50 mg, 81% yield, yellow oil; Flash column chromatography conditions: PE/EtOAc = 50:1. ^{1}H NMR (600 MHz, $CDCl_3$) δ 7.21–7.16 (m, 2H), 7.03–6.99 (m, 2H), 6.20 (td, J = 54.9, 4.5 Hz, 1H), 6.15 (td, J = 54.9, 3.4 Hz, 1H), 4.13–4.03 (m, 2H), 3.16 (ddd, J = 9.3, 6.7, 4.9 Hz, 1H), 3.09 (dd, J = 13.7, 9.3 Hz, 1H), 3.03 (dd, J = 13.7, 6.7 Hz, 1H), 2.72–2.66 (m, 1H), 1.14 (t, J = 7.2 Hz, 3H); ^{13}C NMR (151 MHz, $CDCl_3$) δ 172.2, 161.8 (d, J = 245.0 Hz), 133.4 (d, J = 3.3 Hz), 130.5 (d, J = 8.1 Hz), 115.4 (d, J = 21.4 Hz), 114.5 (tdd, J = 243.6, 7.3, 4.9 Hz), 114.3 (tt, J = 244.0, 7.4 Hz), 61.2, 48.0 (quintet, J = 20.0 Hz), 42.1 (quintet, J = 3.1 Hz), 34.9, 13.8; ^{19}F NMR (565 MHz,

$CDCl_3$) *δ* -115.9, -119.5 – -120.6 (m), -120.0 – -122.8 (m); HRMS (ESI) *m/z*: [*M* + H]$^+$ Calcd for $C_{14}H_{15}F_5O_2$+H$^+$: 311.1065; Found: 311.1064.

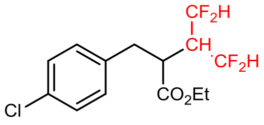

9

Compound ***9*****:** 52 mg, 80% yield, yellow oil; Flash column chromatography conditions: PE/EtOAc = 50:1. ^{1}H NMR (600 MHz, $CDCl_3$) *δ* 7.28–7.26 (m, 2H), 7.15–7.12 (m, 2H), 6.18 (td, *J* = 54.9, 4.6 Hz, 1H), 6.13 (td, *J* = 54.9, 3.4 Hz, 1H), 4.10–4.02 (m, 1H), 3.14 (ddd, *J* = 9.3, 6.5, 4.8 Hz, 1H), 3.07 (dd, *J* = 13.7, 9.3 Hz, 1H), 3.00 (dd, *J* = 13.7, 6.6 Hz, 1H), 2.74–2.59 (m, 1H), 1.13 (t, *J* = 7.1 Hz, 3H);^{13}C NMR (151 MHz, $CDCl_3$) *δ* 172.1, 136.3, 132.7, 130.4, 128.7, 114.5 (tdd, *J* = 243.9, 7.1, 4.9 Hz), 114.2 (tt, *J* = 244.0, 7.3 Hz), 61.3, 48.0 (quintet, *J* = 20.0 Hz), 42.0 (quintet, *J* = 2.9 Hz), 35.0, 13.9; ^{19}F NMR (565 MHz, $CDCl_3$) *δ* -119.5 – -120.6 (m), -120.0 – -122.7 (m); HRMS (ESI) *m/z*: [*M* + H]$^+$ Calcd for $C_{14}H_{15}ClF_4O_2$+H$^+$: 327.0769; Found: 327.0770.

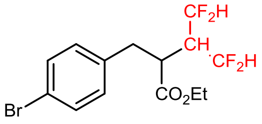

10

Compound ***10*****:** 61 mg, 82% yield, yellow oil; Flash column chromatography conditions: PE/EtOAc = 50:1. ^{1}H NMR (600 MHz, $CDCl_3$) *δ* 7.44–7.41 (m, 2H), 7.09–7.06 (m, 2H), 6.18 (td, *J* = 54.9, 4.6 Hz, 1H), *δ* 6.13 (td, *J* = 54.9, 3.4 Hz, 1H), 4.11–4.02 (m, 2H), 3.14 (ddd, *J* = 9.4, 6.6, 4.8 Hz, 1H), 3.06 (dd, *J* = 13.8, 9.3 Hz, 1H), 2.98 (dd, *J* = 13.7, 6.6 Hz, 1H), 2.72–2.62 (m, 1H), 1.13 (t, *J* = 7.2 Hz, 3H); ^{13}C NMR (151 MHz, $CDCl_3$) *δ* 172.1, 136.8, 131.7, 130.8, 120.8, 114.5 (tdd, *J* = 244.0, 7.2, 4.8 Hz), 114.3 (tt, *J* = 243.9, 7.3 Hz), 61.3, 48.0 (quintet, *J* = 20.0 Hz), 41.9 (quintet, *J* = 2.9 Hz), 35.1, 13.9; ^{19}F NMR (565 MHz, $CDCl_3$) *δ* -119.5 – -120.6 (m), -120.0 – -122.7 (m); HRMS (ESI) *m/z*: [M + H]$^+$ Calcd for $C_{14}H_{15}BrF_4O_2$+H$^+$: 371.0264; Found: 371.0267.

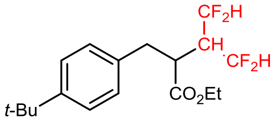

11

Compound **11:** 53 mg, 77% yield, yellow oil; Flash column chromatography conditions: PE/EtOAc = 50:1. ^{1}H NMR (600 MHz, $CDCl_3$) δ 7.33–7.30 (m, 2H), 7.13–7.10 (m, 2H), 6.19 (td, J = 54.9, 4.6 Hz, 1H), 6.13 (td, J = 54.9, 3.5 Hz, 1H), 4.05 (q, J = 7.1 Hz, 2H), 3.17 (ddd, J = 8.5, 7.1, 4.7 Hz, 1H), 3.06 (dd, J = 13.8, 9.0 Hz, 1H), 2.99 (dd, J = 13.8, 7.1 Hz, 1H), 2.71–2.65 (m, 1H), 1.30 (s, 9H), 1.09 (t, J = 7.2 Hz, 3H); ^{13}C NMR (151 MHz, $CDCl_3$) δ 172.5, 149.8, 134.5, 128.7, 125.5, 114.6 (tdd, J = 243.9, 7.6, 5.0 Hz), 114.4 (tt, J = 243.9, 7.3 Hz), 61.1, 47.9 (quintet, J = 19.9 Hz), 42.0 (quintet, J = 2.7 Hz), 35.2, 34.4, 31.3, 13.8; ^{19}F NMR (565 MHz, $CDCl_3$) δ -119.3 – -120.6 (m), -120.1 – -122.7 (m); HRMS (ESI) m/z: $[M + H]^+$ Calcd for $C_{18}H_{24}F_4O_2$+H^+: 349.1785; Found: 349.1788.

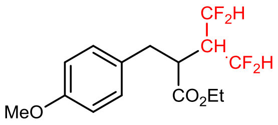

12

Compound **12:** The title compound was prepared according to the general procedure except that Cs_2CO_3 was used, instead of LiOH, in 78% yield (50 mg) as a yellow oil; Flash column chromatography conditions: PE/EtOAc = 50:1. ^{1}H NMR (600 MHz, $CDCl_3$) δ 7.13–7.09 (m, 2H), 6.86–6.82 (m, 2H), 6.18 (td, J = 54.9, 4.7 Hz, 1H), 6.12 (td, J = 54.9, 3.4 Hz, 1H), 4.12–3.99 (m, 2H), 3.79 (s, 3H), 3.14 (ddd, J = 8.9, 7.1, 4.7 Hz, 1H), 3.05 (dd, J = 13.8, 9.0 Hz, 1H), 2.96 (dd, J = 13.8, 7.1 Hz, 1H), 2.72–2.61 (m, 1H), 1.14 (t, J = 7.1 Hz, 3H); ^{13}C NMR (151 MHz, $CDCl_3$) δ 172.4, 158.5, 130.0, 129.6, 114.5 (tdd, J = 244.0, 7.3, 5.1 Hz), 114.3 (tt, J = 244.0, 7.3 Hz), 61.1, 55.2, 47.9 (quintet, J = 19.8 Hz), 42.2 (quintet, J = 3.0 Hz), 34.9, 13.9; ^{19}F NMR (377 MHz, $CDCl_3$) δ -119.1 – -120.8 (m), -120.0 – -122.8 (m); HRMS (ESI) m/z: $[M + H]^+$ Calcd for $C_{15}H_{18}F_4O_3$+H^+: 323.1265; Found: 323.1271.

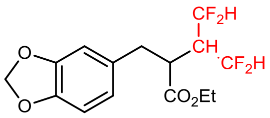

13

Compound **13:** The title compound was prepared according to the general procedure except that Cs_2CO_3 was used, instead of LiOH, in 65% yield (44 mg) as a yellow oil; Flash column chromatography conditions: PE/EtOAc = 50:1. ^{1}H NMR (600 MHz, $CDCl_3$) δ 6.74 (d, J = 7.9 Hz,

1H), 6.69 (d, J = 1.7 Hz, 1H), 6.64 (dd, J = 7.9, 1.8 Hz, 1H), 6.18 (td, J = 54.9, 4.7 Hz, 1H), 6.12 (td, J = 54.9, 3.5 Hz, 1H), 5.94 (s, 2H), 4.12–4.06 (m, 2H), 3.11 (ddd, J = 8.9, 7.0, 4.7 Hz, 1H), 3.02 (dd, J = 13.8, 8.9 Hz, 1H), 2.93 (dd, J = 13.8, 7.0 Hz, 1H), 2.71–2.61 (m, 1H), 1.17 (t, J = 7.2 Hz, 3H); ^{13}C NMR (151 MHz, $CDCl_3$) δ 172.3, 147.8, 146.4, 131.3, 122.1, 114.5 (tdd, J = 244.0, 8.1, 5.1 Hz), 114.3 (tt, J = 243.3, 7.5 Hz), 109.3, 108.3, 101.0, 61.2, 47.7 (quintet, J = 19.9 Hz), 42.3 (quintet, J = 2.9 Hz), 35.4, 13.9; ^{19}F NMR (377 MHz, $CDCl_3$) δ -119.1 – -120.1 (m), -120.0 – -122.8 (m); HRMS (ESI) m/z: $[M + H]^+$ Calcd for $C_{15}H_{16}F_4O_4$+H^+: 337.1057; Found: 337.1062.

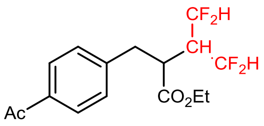

14

Compound ***14*****:** The title compound was prepared according to the general procedure except that Cs_2CO_3 was used, instead of LiOH, in 72% yield (48 mg) as a yellow oil; Flash column chromatography conditions: PE/EtOAc = 10:1. ^{1}H NMR (600 MHz, $CDCl_3$) δ 7.91 (d, J = 8.3 Hz, 2H), 7.31 (d, J = 8.2 Hz, 2H), 6.19 (td, J = 54.8, 4.6 Hz, 1H), 6.14 (td, J = 54.9, 3.5 Hz, 1H), 4.10–4.02 (m, 2H), 3.21 (ddd, J = 9.3, 6.5, 4.8 Hz, 1H), 3.16 (dd, J = 13.7, 9.3 Hz, 1H), 3.09 (dd, J = 13.2, 6.0 Hz, 1H), 2.74–2.64 (m, 1H), 2.60 (s, 3H), 1.12 (t, J = 7.1 Hz, 2H); ^{13}C NMR (151 MHz, $CDCl_3$) δ 197.7, 171.9, 143.5, 135.9, 129.3, 128.7, 114.4 (tdd, J = 244.7, 8.3, 4.9 Hz), 114.2 (tt, J = 244.0, 7.2 Hz), 61.4, 48.1 (quintet, J = 20.0 Hz), 41.7 (quintet, J = 2.9 Hz), 35.5, 26.6, 13.8; ^{19}F NMR (377 MHz, $CDCl_3$) δ -119.7 – -122.9 (m), -120.1 – -120.2 (m); HRMS (ESI) m/z: $[M + H]^+$ Calcd for $C_{16}H_{18}F_4O_3$+H^+: 335.1265; Found: 335.1268.

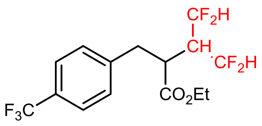

15

Compound ***15*****:** 44 mg, 61% yield, yellow oil; Flash column chromatography conditions: PE/EtOAc = 50:1. ^{1}H NMR (600 MHz, $CDCl_3$) δ 7.57 (d, J = 8.0 Hz, 2H), 7.33 (d, J = 8.0 Hz, 2H), 6.19 (td, J = 54.8, 4.6 Hz, 1H), 6.15 (td, J = 54.8, 3.4 Hz, 1H), 4.10–4.00 (m, 2H), 3.20 (ddd, J = 9.0, 6.1, 4.3 Hz, 1H), 3.17 (dd, J = 14.4, 9.0 Hz, 1H), 3.10 (dd, J = 13.0, 5.7 Hz, 1H), 2.77–2.63 (m, 1H), 1.10 (t, J = 7.1 Hz, 3H); ^{13}C NMR (151 MHz, $CDCl_3$) δ 171.9, 142.0, 129.4, 125.5 (q, J = 3.8 Hz), 124.1

(q, J = 271.8 Hz), 114.4 (tdd, J = 243.7, 7.2, 4.8 Hz), 114.2 (tt, J = 243.6, 7.3 Hz), 61.4, 48.1 (quintet, J = 20.0 Hz), 41.8 (quintet, J = 2.7 Hz), 35.4, 13.8; ^{19}F NMR (565 MHz, $CDCl_3$) δ -62.5 (s), -120.0 – -120.8 (m), -119.9 – -122.8 (m); HRMS (ESI) m/z: $[M + H]^+$ Calcd for $C_{15}H_{15}F_7O_2$+H^+: 361.1033; Found: 361.1034.

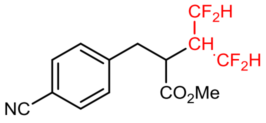

16

Compound ***16*****:** The title compound was prepared according to the general procedure except that Cs_2CO_3 was used, instead of LiOH, in 61% yield (37 mg) as a yellow oil; Flash column chromatography conditions: PE/EtOAc = 10:1. ^{1}H NMR (600 MHz, $CDCl_3$) δ 7.61 (d, J = 8.3 Hz, 2H), 7.32 (d, J = 8.2 Hz, 2H), 6.17 (td, J = 55.1, 4.5 Hz, 1H), 6.13 (td, J = 55.0, 3.6 Hz, 1H), 3.61 (s, 3H), 3.23–3.15 (m, 2H), 3.14–3.06 (m, 1H), 2.78–2.65 (m, 1H); ^{13}C NMR (151 MHz, $CDCl_3$) δ 172.1, 143.5, 132.4, 129.8, 118.6, 114.3 (tdd, J = 244.1, 7.4, 4.8 Hz), 114.1 (tt, J = 244.3, 7.3 Hz), 111.0, 52.3, 48.1 (quintet, J = 20.0 Hz), 41.5 (quintet, J = 2.8 Hz), 35.5; ^{19}F NMR (565 MHz, $CDCl_3$) δ -120.0 – -122.9 (m), -120.4 – -120.5 (m); HRMS (ESI) m/z: $[M + H]^+$ Calcd for $C_{14}H_{13}F_4NO_2$+H^+: 304.0955; Found: 304.0957.

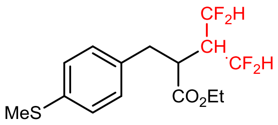

17

Compound ***17*****:** The title compound was prepared according to the general procedure except that Cs_2CO_3 was used, instead of LiOH, in 65% yield (44 mg) as a yellow oil; Flash column chromatography conditions: PE/EtOAc = 50:1. ^{1}H NMR (600 MHz, $CDCl_3$) δ 7.23–7.17 (m, 2H), 7.15–7.10 (m, 2H), 6.18 (td, J = 54.9, 4.7 Hz, 1H), 6.12 (td, J = 54.9, 3.5 Hz, 1H), 4.12–4.01 (m, 2H), 3.15 (ddd, J = 9.1, 6.9, 4.7 Hz, 1H), 3.06 (dd, J = 13.8, 9.1 Hz, 1H), 2.98 (dd, J = 13.8, 6.9 Hz, 1H), 2.73–2.60 (m, 1H), 2.47 (s, 3H), 1.14 (t, J = 7.2 Hz, 3H); ^{13}C NMR (151 MHz, $CDCl_3$) δ 172.2, 136.9, 134.5, 129.5, 126.9, 114.5 (tdd, J = 243.9, 7.5, 5.2 Hz), 114.3 (tt, J = 244.0, 7.2 Hz), 61.2, 47.9 (quintet, J = 19.9 Hz), 42.0 (quintet, J = 2.8 Hz), 35.1, 15.9, 13.9; ^{19}F NMR (565 MHz, $CDCl_3$)

δ -119.4 – -120.6 (m), -120.1 – -122.7 (m); HRMS (ESI) *m/z*: $[M + H]^+$ Calcd for $C_{15}H_{18}F_4O_2S+H^+$: 339.1036; Found: 339.1040.

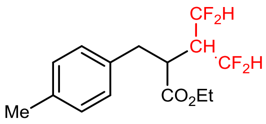

18

Compound ***18*****:** 45 mg, 74% yield, yellow oil; Flash column chromatography conditions: PE/EtOAc = 50:1. ^{1}H NMR (400 MHz, $CDCl_3$) δ 7.13–7.06 (m, 4H), 6.18 (td, *J* = 55.0, 4.6 Hz, 1H), 6.11 (td, *J* = 55.0, 3.5 Hz, 1H), 4.11–4.00 (m, 2H), 3.16 (ddd, *J* = 8.7, 7.2, 4.5 Hz, 1H), 3.07 (dd, *J* = 13.6, 8.7 Hz, 1H), 2.97 (dd, *J* = 13.6, 7.1 Hz, 1H), 2.76–2.55 (m, 1H), 2.32 (s, 3H), 1.14 (t, *J* = 7.1 Hz, 3H); ^{13}C NMR (151 MHz, $CDCl_3$) δ 172.4, 136.4, 134.5, 129.3, 128.9, 116.3–112.9 (m), 115.9–112.7 (m), 114.6 (tdd, *J* = 244.0, 7.7, 4.7 Hz), 114.4 (tt, *J* = 243.9, 7.4 Hz), 61.1, 47.7 (quintet, *J* = 20.0 Hz), 42.1 (quintet, *J* = 2.8 Hz), 35.2, 21.0, 13.8; ^{19}F NMR (377 MHz, $CDCl_3$) δ -119.1 – -120.8 (m), -120.0 – -122.8 (m); HRMS (ESI) *m/z*: $[M + H]^+$ Calcd for $C_{15}H_{18}F_4O_2+H^+$: 307.1316; Found: 307.1316.

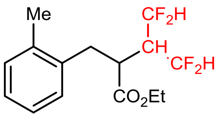

19

Compound ***19*****:** 43 mg, 70% yield, yellow oil; Flash column chromatography conditions: PE/EtOAc = 50:1. ^{1}H NMR (600 MHz, $CDCl_3$) δ 7.15 (dd, *J* = 6.1, 1.9 Hz, 2H), 7.13–7.08 (m, 2H), 6.19 (td, *J* = 55.0, 4.4 Hz, 1H), 6.17 (td, *J* = 54.9, 3.1 Hz, 1H), 4.05–3.97 (m, 2H), 3.21–3.15 (m, 1H), 3.09–3.05 (m, 2H), 2.77–2.64 (m, 1H), 2.33 (s, 3H), 1.06 (t, *J* = 7.2 Hz, 3H); ^{13}C NMR (151 MHz, $CDCl_3$) δ 172.5, 136.4, 135.7, 130.6, 129.8, 127.1, 126.0, 114.5 (tdd, *J* = 243.3, 6.6, 3.5 Hz), 114.3 (tt, *J* = 243.6, 6.9 Hz), 61.1, 48.1 (quintet, *J* = 20.0 Hz), 40.4 (quintet, *J* = 2.7 Hz), 33.4, 19.2, 13.8; ^{19}F NMR (565 MHz, $CDCl_3$) δ -119.4 – -120.7 (m), -120.0 – -122.8 (m); HRMS (ESI) *m/z*: $[M + H]^+$ Calcd for $C_{15}H_{18}F_4O_2+H^+$: 307.1316; Found: 307.1312.

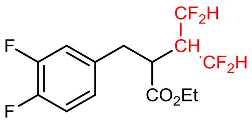

20

*Compound **20***: 50 mg, 76% yield, yellow oil; Flash column chromatography conditions: PE/EtOAc = 50:1. ^{1}H NMR (600 MHz, $CDCl_3$) δ 7.18–7.13 (m, 1H), 6.85–6.78 (m, 2H), 6.18 (td, J = 54.8, 4.6 Hz, 1H), 6.15 (td, J = 54.8, 3.5 Hz, 1H); 4.14–4.00 (m, 2H), 3.20 (ddd, J = 8.7, 7.2, 4.9 Hz, 1H), 3.07 (d, J = 7.9 Hz, 2H), 2.72–2.67 (m, 1H), 1.13 (t, J = 7.1 Hz, 3H); ^{13}C NMR (151 MHz, $CDCl_3$) δ 172.0, 162.1 (dd, J = 248.1, 12.1 Hz), 161.2 (dd, J = 248.6, 12.0 Hz), 132.1 (dd, J = 9.4, 6.1 Hz), 114.4 (tdd, J = 244.3, 7.8, 4.7 Hz), 114.3 (tt, J = 243.9, 7.2 Hz), 111.2 (dd, J = 21.0, 3.8 Hz), 103.9 (t, J = 25.6 Hz), 61.3, 48.3 (quintet, J = 19.9 Hz), 40.5 (quintet, J = 2.8 Hz), 29.1, 13.8; ^{19}F NMR (377 MHz, $CDCl_3$) δ -111.51 (d, J = 7.6 Hz), -113.27 (d, J = 7.5 Hz), -119.3 – -121.1 (m), -120.2 – -122.6 (m); HRMS (ESI) *m*/*z*: [*M* + H]$^+$ Calcd for $C_{14}H_{14}F_6O_2$+H$^+$: 329.0971; Found: 329.0970.

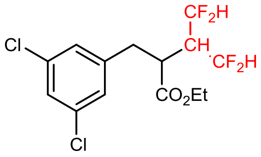

21

*Compound **21***: 53 mg, 69% yield, yellow oil; Flash column chromatography conditions: PE/EtOAc = 50:1. ^{1}H NMR (600 MHz, $CDCl_3$) δ 7.25 (t, J = 1.9 Hz, 1H), 7.10 (d, J = 1.9 Hz, 2H), 6.17 (td, J = 54.9, 4.5 Hz, 1H), 6.14 (td, J = 54.7, 3.4 Hz, 1H); 4.11–4.07 (m, 2H), 3.14 (ddd, J = 8.7, 7.2, 4.9 Hz, 1H), 3.05 (dd, J = 13.8, 9.7 Hz, 1H), 2.99 (dd, J = 13.7, 5.9 Hz, 1H), 2.76–2.67 (m, 1H), 1.15 (t, J = 7.2 Hz, 3H); ^{13}C NMR (151 MHz, $CDCl_3$) δ 171.7, 141.3, 135.0, 127.6, 127.2, 114.3 (tdd, J = 244.0, 7.1, 4.6 Hz), 114.1 (tt, J = 243.9, 7.3 Hz), 61.5, 48.1 (quintet, J = 19.9 Hz), 41.7 (quintet, J = 2.9 Hz), 35.1, 13.9; ^{19}F NMR (565 MHz, $CDCl_3$) δ -119.7 – -122.7 (m), -119.8 – -120.9 (m); HRMS (ESI) *m*/*z*: [*M* + Na]$^+$ Calcd for $C_{14}H_{14}Cl_2F_4O_2$+Na$^+$: 383.0199; Found: 383.0197.

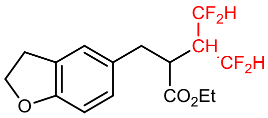

22

Compound ***22*****:** The title compound was prepared according to the general procedure except that Cs_2CO_3 was used, instead of LiOH, in 61% yield (41 mg) as a yellow oil; Flash column chromatography conditions: PE/EtOAc = 50:1. ^{1}H NMR (600 MHz, $CDCl_3$) δ 7.03 (d, J = 1.9 Hz, 1H), 6.91 (dd, J = 8.1, 1.9 Hz, 1H), 6.70 (d, J = 8.1 Hz, 1H), 6.19 (td, J = 54.9, 4.7 Hz, 1H), 6.12 (td, J = 55.0, 3.4 Hz, 1H), 4.56 (t, J = 8.7 Hz, 2H), 4.11–4.04 (m, 2H), 3.18 (td, J = 8.6, 2.8 Hz, 2H), 3.12 (ddd, J = 9.0, 7.1, 4.7 Hz, 1H), 3.03 (dd, J = 13.8, 8.9 Hz, 1H), 2.94 (dd, J = 13.8, 7.1 Hz, 1H), 2.70–2.62 (m, 1H), 1.15 (t, J = 7.2 Hz, 3H); ^{13}C NMR (151 MHz, $CDCl_3$) δ 172.5, 159.0, 129.5, 128.6, 127.3, 125.6, 114.5 (tdd, J = 243.8, 7.3, 4.9 Hz), 114.3 (tt, J = 243.6, 7.2 Hz), 109.2, 71.2, 61.1, 47.8 (quintet, J = 19.9 Hz), 42.5 (quintet, J = 2.7 Hz), 35.2, 29.7, 13.9; ^{19}F NMR (565 MHz, $CDCl_3$) δ -119.2 – -120.6 (m), -120.2 – -122.7 (m); HRMS (ESI) m/z: $[M + H]^+$ Calcd for $C_{16}H_{18}F_4O_3+H^+$: 335.1265; Found: 335.1265.

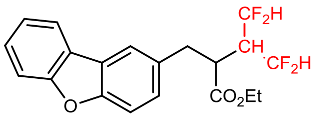

23

Compound ***23*****:** The title compound was prepared according to the general procedure except that Cs_2CO_3 was used, instead of LiOH, in 72% yield (55 mg) as a yellow oil; Flash column chromatography conditions: PE/EtOAc = 50:1. ^{1}H NMR (600 MHz, $CDCl_3$) δ 7.95–7.93 (m, 1H), 7.78 (d, J = 1.8 Hz, 1H), 7.56 (dt, J = 8.1, 0.8 Hz, 1H), 7.50 (d, J = 8.4 Hz, 1H), 7.46 (ddd, J = 8.4, 7.3, 1.3 Hz, 1H), 7.35 (td, J = 7.5, 1.0 Hz, 1H), 7.29 (dd, J = 8.4, 1.9 Hz, 1H), 6.23 (td, J = 54.9, 4.6 Hz, 1H), 6.16 (td, J = 54.9, 3.3 Hz, 1H); 4.08–4.00 (m, 2H), 3.27–3.25 (m, 2H), 3.20 (dd, J = 10.2, 7.4 Hz, 1H), 2.78–2.68 (m, 1H), 1.07 (t, J = 7.1 Hz, 3H); ^{13}C NMR (151 MHz, $CDCl_3$) δ 172.4, 156.5, 155.2, 132.2, 128.1, 127.3, 124.5, 123.9, 122.7, 121.0, 120.7, 114.6 (tdd, J = 244.0, 7.3, 5.2 Hz), 114.3 (tt, J = 243.7, 7.1 Hz), 111.7, 111.6, 61.2, 48.0 (quintet, J = 19.7 Hz), 42.6 (quintet, J = 2.7 Hz), 35.7, 13.9; ^{19}F NMR (565 MHz, $CDCl_3$) δ -119.3 – -120.6 (m), -120.0 – -122.6 (m); HRMS (ESI) m/z: $[M + H]^+$ Calcd for $C_{20}H_{18}F_4O_3+H^+$: 383.1265; Found: 383.1265.

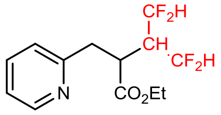

24

Compound ***24*****:** The title compound was prepared according to the general procedure except that Cs_2CO_3 was used, instead of LiOH, in 70% yield (41 mg) as a yellow oil; Flash column chromatography conditions: PE/EtOAc = 10:1. ^{1}H NMR (400 MHz, $CDCl_3$) δ 8.55–8.53 (m, 1H), 7.62 (td, J = 7.7, 1.8 Hz, 1H), 7.20–7.14 (m, 2H), 6.19 (tdd, J = 54.9, 5.3, 4.2 Hz, 2H), 4.12–4.04 (m, 2H), 3.65 (ddd, J = 9.0, 6.1, 4.3 Hz, 1H), 3.34 (dd, J = 14.4, 9.0 Hz, 1H), 3.16 (dd, J = 14.4, 6.1 Hz, 1H), 2.89–2.72 (m, 1H), 1.15 (t, J = 7.1 Hz, 3H); ^{13}C NMR (151 MHz, $CDCl_3$) δ 172.3, 157.8, 149.3, 136.5, 123.8, 121.8, 114.6 (tdd, J = 244.0, 7.2, 4.8 Hzj), 114.5 (tt, J = 243.9, 7.1 Hz), 61.2, 48.1 (quintet, J = 20.1 Hz), 39.8 (quintet, J = 3.1 Hz), 37.1, 13.8; ^{19}F NMR (377 MHz, $CDCl_3$) δ -119.2 – -120.8 (m), -120.0 – -122.2 (m); HRMS (ESI) m/z: $[M + H]^+$ Calcd for $C_{13}H_{15}F_4NO_2+H^+$: 294.1112; Found: 294.1107.

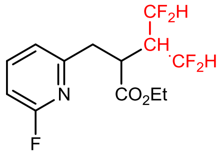

25

Compound **25:** The title compound was prepared according to the general procedure except that Cs_2CO_3 was used, instead of LiOH, in 64% yield (40 mg) as a yellow oil; Flash column chromatography conditions: PE/EtOAc = 50:1. ^{1}H NMR (600 MHz, CDCl3) δ 7.73–7.69 (m, 1H), 7.08 (dd, J = 7.3, 2.4 Hz, 1H), 6.79 (dd, J = 8.2, 2.8 Hz, 1H), 6.17 (tdd, J = 55.0, 8.0, 4.3 Hz, 2H), 4.15–4.06 (m, 2H), 3.61 (ddd, J = 9.7, 5.8, 4.3 Hz, 1H), 3.31 (dd, J = 14.6, 9.3 Hz, 1H), 3.10 (dd, J = 14.6, 5.8 Hz, 1H), 2.84–2.73 (m, 1H), 1.17 (t, J = 7.1 Hz, 3H); ^{13}C NMR (151 MHz, CDCl3) δ 172.0, 163.2 (d, J = 239.5 Hz), 157.1 (d, J = 13.0 Hz), 141.4 (d, J = 7.8 Hz), 121.0 (d, J = 4.2 Hz), 114.6 (tdd, J = 244.1, 6.7, 4.8 Hz), 114.4 (tt, J = 244.1, 7.0 Hz), 107.4 (d, J = 36.9 Hz), 61.3, 48.2 (quintet, J = 19.9 Hz, 39.4 (quintet, J = 3.0 Hz), 36.4, 13.9; ^{19}F NMR (565 MHz, CDCl3) δ -67.1, -119.6 – -120.6 (m), -120.2 – -122.0 (m); HRMS (ESI) m/z: $[M + H]^+$ Calcd for $C_{13}H_{14}F_5NO_2+H^+$: 312.1017; Found: 312.1013.

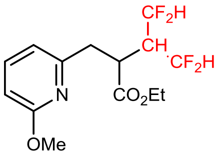

26

*Compound 26***:** The title compound was prepared according to the general procedure except that Cs_2CO_3 was used, instead of LiOH, in 73% yield (47 mg) as a yellow oil; Flash column chromatography conditions: PE/EtOAc = 50:1. ^{1}H NMR (600 MHz, $CDCl_3$) δ 7.48 (dd, J = 8.3, 7.2 Hz, 1H), 6.74 (d, J = 7.2 Hz, 1H), 6.59 (d, J = 8.2 Hz, 1H), 6.19 (tdd, J = 55.0, 6.2, 4.4 Hz, 2H), 4.17–4.09 (m, 2H), 3.89 (s, 3H), 3.69 (td, J = 7.4, 4.1 Hz, 1H), 3.27 (dd, J = 14.8, 7.9 Hz, 1H), 3.07 (dd, J = 14.8, 6.9 Hz, 1H), 2.87–2.76 (m, 1H), 1.19 (t, J = 7.2 Hz, 3H); ^{13}C NMR (101 MHz, $CDCl_3$) δ 172.5, 163.7, 155.2, 138.9, 116.1, 114.7 (tdd, J = 244.0, 7.2, 4.8 Hz), 114.6 (tt, J = 243.9, 7.3 Hz), 108.7, 61.2, 53.2, 47.8 (quintet, J = 19.9 Hz), 39.1 (quintet, J = 3.1 Hz), 36.4, 13.9; ^{19}F NMR (565 MHz, $CDCl_3$) δ -119.5 – -120.6 (m), -120.5 – -122.3 (m); HRMS (ESI) m/z: $[M + H]^+$ Calcd for $C_{14}H_{17}F_4NO_3+H^+$: 324.1217; Found: 324.1214.

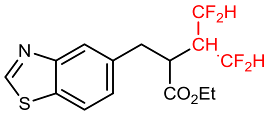

27

*Compound 27***:** The title compound was prepared according to the general procedure except that Cs_2CO_3 was used, instead of LiOH, in 54% yield (38 mg) as a yellow oil; Flash column chromatography conditions: PE/EtOAc = 50:1. ^{1}H NMR (600 MHz, $CDCl_3$) δ 8.98 (s, 1H), 8.08 (d, J = 8.4 Hz, 1H), 7.81 (d, J = 1.7 Hz, 1H), 7.37 (dd, J = 8.4, 1.8 Hz, 1H), 6.21 (td, J = 54.9, 4.6 Hz, 1H), 6.15 (td, J = 54.9, 3.4 Hz, 1H), 4.10–3.98 (m, 2H), 3.28–3.23 (m, 2H), 3.22–3.18 (m, 1H), 2.78–2.67 (m, 1H), 1.07 (t, J = 7.2 Hz, 3H); ^{13}C NMR (151 MHz, $CDCl_3$) δ 172.1, 153.9, 152.3, 135.6, 134.1, 127.4, 123.6, 122.2, 114.5 (tdd, J = 244.0, 7.2, 4.9 Hz), 114.3 (tt, J = 244.0, 7.2 Hz), 61.3, 48.1 (quintet, J = 20.2 Hz), 42.3 (quintet, J = 2.9 Hz), 35.7, 13.9; ^{19}F NMR (565 MHz, $CDCl_3$) δ -119.5 – -120.5 (m), -120.0 – -122.7 (m); HRMS (ESI) m/z: $[M + H]^+$ Calcd for $C_{15}H_{15}F_4NO_2S+H^+$: 350.0832; Found: 350.0828.

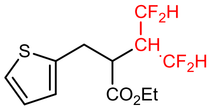

28

Compound ***28*****:** The title compound was prepared according to the general procedure except that Cs_2CO_3 was used, instead of LiOH, in 40% yield (24 mg) as a yellow oil; Flash column chromatography conditions: PE/EtOAc = 50:1. ^{1}H NMR (600 MHz, $CDCl_3$) δ 7.18 (dd, J = 5.1, 1.2 Hz, 1H), 6.93 (dd, J = 5.1, 3.4 Hz, 1H), 6.87–6.85 (m, 1H), 6.18 (td, J = 55.0, 4.7 Hz, 1H), 6.13 (td, J = 55.1, 3.7 Hz, 1H), 4.16–4.11 (m, 2H), 3.37 (ddd, J = 9.0, 6.1, 4.3 Hz, 1H), 3.25 (dd, J = 14.8, 7.0 Hz, 1H), 3.18 (dd, J = 14.8, 6.1 Hz, 1H), 2.77– 2.66 (m, 1H), 1.20 (t, J = 7.2 Hz, 3H); ^{13}C NMR (151 MHz, $CDCl_3$) δ 171.9, 139.8, 126.9, 126.5, 124.6, 114.5 (tdd, J = 244.9, 7.6, 4.8 Hz), 114.3 (tt, J = 244.5, 7.5 Hz), 61.4, 47.7 (quintet, J = 19.9 Hz), 42.5 (quintet, J = 3.0 Hz), 29.9, 13.9; ^{19}F NMR (565 MHz, $CDCl_3$) δ -119.4 – -120.5 (m), -120.1 – -122.6 (m); HRMS (ESI) *m*/*z*: [*M* + H]$^+$ Calcd for $C_{12}H_{14}F_4O_2S$+H$^+$: 299.0723; Found: 299.0725.

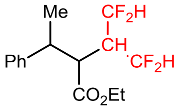

29

Compound ***29*****:** 40 mg, 65% yield, dr = 1.5:1, yellow oil; Flash column chromatography conditions: PE/EtOAc = 50:1. ^{1}H NMR (600 MHz, $CDCl_3$) data of major isomer δ 7.36–7.33 (m, 2H), 7.25–7.22 (m, 1H), 7.21–7.19 (m, 2H), 6.24 (td, J = 55.8, 5.8 Hz, 1H), 5.90 (td, J = 55.3, 2.9 Hz, 1H), 4.26– 4.22 (m, 2H), 3.30–3.23 (m, 1H), 3.11 (dt, J = 11.6, 2.6 Hz, 1H), 2.30–2.19 (m, 1H), 1.31 (t, J = 7.2 Hz, 3H), 1.27 (d, J = 6.9 Hz, 3H); ^{13}C NMR (151 MHz, $CDCl_3$) data of major isomer δ 172.5, 142.8, 129.0, 127.5, 127.0, 114.9 (tdd, J = 244.5, 7.8, 4.8 Hz), 114.3 (tt, J = 243.4, 8.3 Hz), 61.2 , 46.6 (quintet, J = 19.9 Hz), 46.3 (quintet, J = 3.1 Hz), 40.0. 21.2, 14.0; ^{19}F NMR (565 MHz, $CDCl_3$) data of major isomer δ -117.0 – -120.5 (m), -122.5 – -124.1 (m); HRMS (ESI) *m*/*z*: [*M* + H]$^+$ Calcd for $C_{15}H_{18}F_4O_2$+H$^+$: 307.1316; Found: 307.1316.

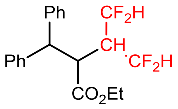

30

*Compound **30***: 55 mg, 75% yield, yellow oil; Flash column chromatography conditions: PE/EtOAc = 50:1. ^{1}H NMR (600 MHz, $CDCl_3$) δ 7.37–7.31 (m, 6H), 7.28–7.21 (m, 3H), 7.16 (td, J = 8.0, 7.3, 1.3 Hz, 1H), 6.35 (td, J = 55.7, 6.0 Hz, 1H), 6.00 (td, J = 55.3, 3.2 Hz,1H), 4.44 (d, J = 12.5 Hz, 1H), 3.93–3.81 (m, 3H), 2.58–2.45 (m, 1H), 0.90 (t, J = 7.2 Hz, 3H); ^{13}C NMR (151 MHz, $CDCl_3$) δ 171.6, 141.3, 140.4, 129.2, 128.5, 128.0, 127.9, 127.2, 127.0, 114.9 (tdd, J = 244.7, 7.6, 4.7 Hz), 114.3 (tt, J = 242.0, 8.3 Hz), 61.0, 51.9, 46.5 (quintet, J = 19.9 Hz), 44.2 (quintet, J = 3.3 Hz), 13.5; ^{19}F NMR (377 MHz, $CDCl_3$) δ -116.7 – -120.6 (m), -122.3 – -124.3 (m); HRMS (ESI) *m*/*z*: [*M* + H]$^+$ Calcd for $C_{20}H_{20}F_4O_2$+H$^+$: 369.1472; Found: 369.1475.

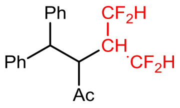

31

*Compound **31***: The title compound was prepared according to the general procedure except that Cs_2CO_3 was used, instead of LiOH, in 53% yield (38 mg) as a yellow oil; Flash column chromatography conditions: PE/EtOAc = 50:1. ^{1}H NMR (600 MHz, $CDCl_3$) δ 7.37–7.33 (m, 4H), 7.34–7.28 (m, 3H), 7.27–7.22 (m, 2H), 7.20–7.16 (m, 1H), 6.29 (td, J = 56.4, 6.8 Hz, 1H), 5.96 (td, J = 55.5, 3.2 Hz,1H), 4.36 (d, J = 12.6 Hz, 1H), 4.12 (d, J = 12.7 Hz, 1H), 2.69–2.52 (m, 1H), 1.79 (s, 3H); ^{13}C NMR (151 MHz, $CDCl_3$) δ 210.3, 141.2, 140.3, 129.2, 128.9, 128.2, 128.0, 127.2, 127.2, 114.9 (tdd, J = 239.5, 7.6, 5.1 Hz), 114.6 (tt, J = 242.1, 7.4 Hz), 52.5, 48.9, 47.5 (quintet, J = 19.8 Hz), 33.2; ^{19}F NMR (377 MHz, $CDCl_3$) δ -116.4 – -119.3 (m), -119.7 – -123.2 (m); HRMS (ESI) *m*/*z*: [*M* + Na]$^+$ Calcd for $C_{19}H_{18}F_4O$+Na$^+$: 361.1186; Found: 361.1183.

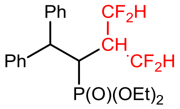

32

*Compound **32***: 48 mg, 56% yield, yellow solid; Flash column chromatography conditions: PE/EtOAc = 5:1. ^{1}H NMR (400 MHz, $CDCl_3$) δ 7.40–7.36 (m, 2H), 7.32 (d, J = 4.3 Hz, 4H), 7.26–7.11 (m, 4H), 6.38 (td, J = 56.4, 7.0 Hz, 1H), 6.28 (td, J = 55.2, 5.6 Hz, 1H), 4.40 (dd, J = 12.4, 8.4 Hz, 1H), 3.91–3.70 (m, 4H), 3.39–3.25 (m, 1H), 2.79–2.61 (m, 1H), 1.21 (t, J = 7.2 Hz, 3H), 1.08 (t, J = 7.2 Hz, 3H); ^{13}C NMR (151 MHz, $CDCl_3$) δ 142.0, 141.5 (d, J = 15.3 Hz), 129.3, 128.9 (d, J

= 36.6 Hz), 128.4, 128.2, 128.0, 127.0 (d, J = 49.8 Hz), 115.0 (tdd, J = 242.7, 7.6, 5.2 Hz), 114.8 (tt, J = 243.2, 7.2 Hz), 62.1 (d, J = 7.2 Hz), 61.6 (d, J = 6.8 Hz), 51.6, 47.9 (quintet, J = 20.2 Hz), 38.1 (d, J = 140.2 Hz), 16.1 (d, J = 6.1 Hz), 15.9 (d, J = 6.6 Hz); ^{19}F NMR (377 MHz, $CDCl_3$) δ -115.0 – -116.7 (m), -115.4 – -122.6 (m); ^{31}P NMR (162 MHz, $CDCl_3$) δ 26.8; HRMS (ESI) m/z: $[M + H]^+$ Calcd for $C_{21}H_{25}F_4O_3P+H^+$: 433.1550; Found: 433.1545.

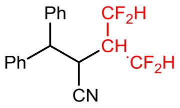

33

Compound ***33*****:** 28 mg, 44% yield, yellow oil; Flash column chromatography conditions: PE/EtOAc = 50:1. ^{1}H NMR (600 MHz, $CDCl_3$) δ 7.41–7.38 (m, 2H), 7.38–7.34 (m, 4H), 7.30–7.27 (m, 4H), 6.22 (td, J = 54.6, 6.6 Hz, 1H), 6.02 (td, J = 54.8, 2.6 Hz, 1H), 4.38 (d, J = 12.0 Hz, 1H), 3.93 (d, J = 12.0 Hz, 1H), 2.58–2.46 (m, 1H); ^{13}C NMR (151 MHz, $CDCl_3$) δ 139.6, 138.6, 129.5, 129.1, 128.0, 127.8, 127.7, 117.1, 114.7 (tdd, J = 242.1, 7.6, 2.7 Hz), 113.2 (tt, J = 246.6, 7.2 Hz), 52.0 (quintet, J = 2.5 Hz), 45.8 (quintet, J = 20.7 Hz), 30.5; ^{19}F NMR (565 MHz, $CDCl_3$) δ -119.3 – -120.6 (m), -122.3 – -125.4 (m); HRMS (ESI) m/z: $[M + H]^+$ Calcd for $C_{18}H_{15}F_4N+H^+$: 322.1213; Found: 322.1218.

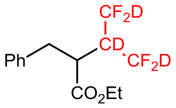

36

Compound ***36*****:** 47 mg, 80% yield, yellow oil; Flash column chromatography conditions: PE/EtOAc = 50:1. ^{1}H NMR (600 MHz, $CDCl_3$) δ 7.33–7.28 (m, 2H), 7.25–7.22 (m, 1H), 7.21–7.18 (m, 2H), 4.10–4.02 (m, 2H), 3.18 (dd, J = 9.0, 7.0 Hz, 1H), 3.10 (dd, J = 13.7, 9.1 Hz, 1H), 3.02 (dd, J = 13.7, 7.0 Hz, 1H), 1.11 (t, J = 7.1 Hz, 3H); ^{13}C NMR (151 MHz, $CDCl_3$) δ 172.4, 137.7, 129.0, 128.6, 126.9, 114.5 (tdd, J = 244.0, 7.2, 4.8 Hz), 114.3 (tt, J = 243.9, 7.3 Hz), 61.2, 47.3 (quintet, J = 20.0 Hz), 41.9 (quintet, J = 2.7 Hz), 35.7, 13.8; ^{19}F NMR (565 MHz, $CDCl_3$) δ -120.2 – -121.5 (m), -121.0 – -123.6 (m); HRMS (ESI) m/z: $[M + H]^+$ Calcd for $C_{14}H_{13}D_3F_4O_2+H^+$: 296.1347; Found: 296.1347.

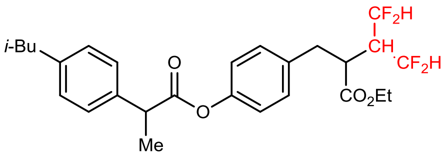

37

Compound ***37*****:** The title compound was prepared according to the general procedure except that Cs_2CO_3 was used, instead of LiOH, in 50% yield (50 mg) as a yellow oil, dr = 1:1, Flash column chromatography conditions: PE/EtOAc = 30:1. ^1H NMR (600 MHz, $CDCl_3$) δ 7.29 (d, J = 8.0 Hz, 2H), 7.19–7.12 (m, 4H), 6.93 (d, J = 8.4 Hz, 2H), 6.17 (td, J = 54.9, 4.6 Hz, 1H), 6.11 (td, J = 54.9, 3.4 Hz, 1H), 4.10–4.00 (m, 2H), 3.92 (q, J = 7.1 Hz, 1H), 3.14 (ddd, J = 9.2, 6.6, 4.8 Hz, 1H), 3.07 (dd, J = 13.7, 9.2 Hz, 1H), 2.99 (dd, J = 13.7, 6.6 Hz, 1H), 2.71–2.60 (m, 1H), 2.47 (d, J = 7.2 Hz, 2H), 1.88–1.84 (m, 1H), 1.59 (d, J = 7.2 Hz, 3H), 1.12 (t, J = 7.1 Hz, 3H), 0.91 (d, J = 6.6 Hz, 6H); ^{13}C NMR (151 MHz, $CDCl_3$) δ 173.2, 172.2, 149.8, 140.8, 137.2, 135.2, 129.9, 129.5, 127.2, 121.5, 114.5 (tdd, J = 244.0, 7.2, 4.8 Hz), 114.3 (tt, J = 243.9, 7.3 Hz), 61.3, 48.0 (quintet, J = 20.0 Hz), 45.2, 45.0, 42.0 (quintet, J = 2.9 Hz), 35.1, 30.2, 22.4, 18.5, 13.8; ^{19}F NMR (565 MHz, $CDCl_3$) δ -119.5 – -120.5 (m), -120.2 – -122.7 (m); HRMS (ESI) m/z: $[M + H]^+$ Calcd for $C_{27}H_{32}F_4O_4+H^+$: 497.2309; Found: 497.2329.

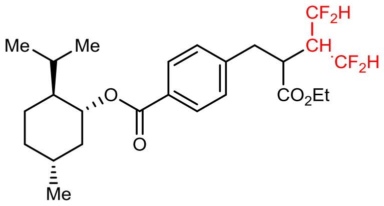

38

Compound ***38*****:** The title compound was prepared according to the general procedure except that Cs_2CO_3 was used, instead of LiOH, in 60% yield (57 mg) as a yellow oil, dr = 1:1; Flash column chromatography conditions: PE/EtOAc = 50:1. ^1H NMR (600 MHz, $CDCl_3$) δ 8.03–7.98 (m, 2H), 7.33–7.27 (m, 2H), 6.21 (td, J = 54.9, 4.6 Hz, 1H), 6.15 (td, J = 54.9, 3.5 Hz, 1H), 4.94 (td, J = 10.9, 4.4 Hz, 1H), 4.12–4.05 (m, 2H), 3.25–3.16 (m, 1H), 3.10 (dd, J = 13.0, 5.9 Hz, 1H), 2.76–2.64 (m, 1H), 2.14 (dd, J = 12.1, 4.4 Hz, 1H), 2.0–1.94 (m, 1H), 1,78–1.73 (m, 2H), 1.60–1.54 (m, 2H), 1.17–1.09 (m, 5H), 0.98–0.89 (m, 8H), 0.81 (d, J = 7.0 Hz, 3H); ^{13}C NMR (151 MHz, $CDCl_3$) δ 172.0 (d, J = 2.5 Hz), 165.8, 142.9, 129.9, 129.6, 129.0, 114.5 (tdd, J = 243.4, 7.3, 5.5 Hz), 114.2 (tt, J = 244.0, 7.6 Hz), 74.8, 61.4, 48.0 (quintet, J = 19.9 Hz), 47.2, 41.8 (quintet, J = 2.8 Hz), 41.0, 35.5,

34.3, 31.4, 26.5 (d, J = 4.7 Hz), 23.6 (d, J = 5.2 Hz), 22.0, 20.8 (d, J = 2.6 Hz), 16.5 (d, J = 4.9 Hz), 13.8 (d, J = 1.4 Hz); ^{19}F NMR (565 MHz, $CDCl_3$) δ -120.0 – -122.7 (m), -120.1 – -120.2 (m); HRMS (ESI) m/z: $[M + H]^+$ Calcd for $C_{25}H_{34}F_4O_4$+H^+: 475.2466; Found: 475.2466.

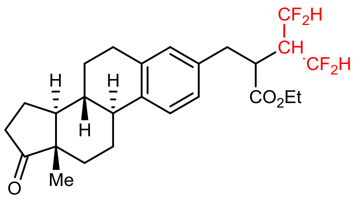

39

Compound ***39*****:** The title compound was prepared according to the general procedure except that Cs_2CO_3 was used, instead of LiOH, in 63% yield (59 mg) as a yellow oil, dr = 1:1; Flash column chromatography conditions: PE/EtOAc = 10:1. ^{1}H NMR (600 MHz, $CDCl_3$) δ 7.22 (d, J = 8.0 Hz, 1H), 6.98 (d, J = 8.0 Hz, 1H), 6.92 (s, 1H), 6.20 (td, J = 54.9, 4.6 Hz, 1H), 6.12 (td, J = 55.0, 3.6 Hz, 1H), 4.11–4.06 (m, 2H), 3.17 (q, J = 7.6 Hz, 1H), 3.06 (dd, J = 13.8, 8.7 Hz, 1H), 2.94 (dd, J = 13.9, 7.2 Hz, 1H), 2.93–2.87 (m, 2H), 2.75–2.65 (m, 1H), 2.51 (dd, J = 19.1, 8.7 Hz, 1H), 2.41 (dt, J = 9.7, 2.9 Hz, 1H), 2.28 (td, J = 10.9, 4.1 Hz, 1H), 2.15 (dt, J = 19.1, 8.9 Hz, 1H), 2.11–2.00 (m, 2H), 2.00–1.92 (m, 1H), 1.60–1.56 (m, 2H), 1.55–1.47 (m, 3H), 1.48–1.40 (m, 1H), 1.16 (td, J = 7.1, 1.6 Hz, 3H), 0.91 (s, 3H); ^{13}C NMR (151 MHz, $CDCl_3$) δ 220.8, 172.4, 138.3, 136.7, 135.1, 129.6, 126.3 (d, J = 3.1 Hz), 125.6 (d, J = 3.8 Hz), 114.5 (tdd, J = 244.1, 7.7, 4.6 Hz), 114.3 (tt, J = 244.0, 7.6 Hz), 61.1, 50.5, 48.0 (quintet, J = 19.9 Hz), 47.9, 44.3 (d, J = 1.9 Hz), 42.0 (quintet, J = 2.8 Hz), 38.1, 35.8, 35.1, 31.6, 29.3, 26.5, 25.7 (d, J = 3.8 Hz), 21.6, 13.8; ^{19}F NMR (565 MHz, $CDCl_3$) δ -119.2 – -120.5 (m), -120.2 – -122.6 (m); HRMS (ESI) m/z: $[M + H]^+$ Calcd for $C_{26}H_{32}F_4O_3$+H^+: 469.2360; Found: 469.2354.

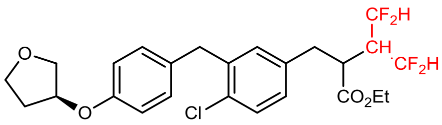

40

Compound ***40*****:** The title compound was prepared according to the general procedure except that Cs_2CO_3 was used, instead of LiOH, in 74% yield (74 mg) as a yellow oil, dr = 1:1; Flash column chromatography conditions: PE/EtOAc = 10:1. ^{1}H NMR (600 MHz, $CDCl_3$) δ 7.31–7.27 (m, 1H),

7.10–7.06 (m, 2H), 7.00 (dd, J = 6.0, 2.3 Hz, 2H), 6.80–6.75 (m, 2H), 6.15 (td, J = 55.0, 4.6 Hz, 1H), 6.10 (td, J = 55.0, 3.5 Hz, 1H), 4.89 (ddt, J = 6.4, 4.4, 2.1 Hz, 1H), 4.07–4.02 (m, 1H), 4.02–3.94 (m, 6H), 3.89 (td, J = 8.3, 4.4 Hz, 1H), 3.10 (ddd, J = 9.2, 6.8, 4.7 Hz, 1H), 3.03 (dd, J = 13.7, 9.2 Hz, 1H), 2.94 (dd, J = 13.7, 6.8 Hz, 1H), 2.67–2.57 (m, 1H), 2.21–2.12 (m, 2H), 1.10 (t, J = 7.1 Hz, 3H); ^{13}C NMR (151 MHz, $CDCl_3$) δ 172.0, 155.9, 139.2, 136.6, 132.7, 131.8, 131.6, 129.8, 129.7, 128.2, 115.4, 114.4 (tdd, J = 243.9, 8.3, 4.8 Hz), 114.2 (tt, J = 243.9, 7.1 Hz), 73.1, 67.2, 61.2, 48.0 (quintet, J = 20.0 Hz), 42.0 (quintet, J = 2.9 Hz), 38.2, 35.0, 33.0, 13.9; ^{19}F NMR (565 MHz, $CDCl_3$) δ -119.2 – -120.8 (m), -119.8 – -122.8 (m); HRMS (ESI) m/z: $[M + H]^+$ Calcd for $C_{25}H_{27}ClF_4O_4+H^+$: 503.1607; Found: 503.1616.

4.2 General procedures and analytical data for carbotetrafluoroisopropylation of alkynes

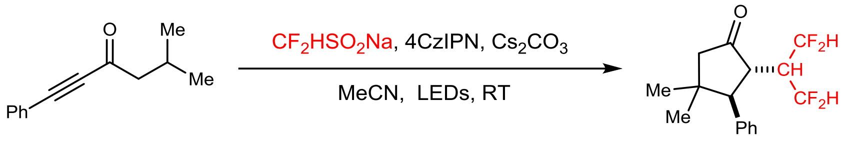

41a **42**

To a mixture of CF_2HSO_2Na (112 mg, 0.8 mmol), 4CzIPN (3.2 mg, 0.004 mmol) and Cs_2CO_3 (130 mg, 0.4 mmol) in 2 mL of MeCN was added **41a** (35.2 mg, 0.2 mmol) under a nitrogen atmosphere. After 18 h of irradiation at a distance of ~2 cm with 24 W of blue LEDs (PINO® lamps, 100% light intensity) at 25 °C, the reaction mixture was quenched with water, extracted with EtOAc, washed with brine, dried over anhydrous Na_2SO_4, and concentrated. Column chromatography on silica gel (PE/EtOAc = 50:1) gave 52 mg (86% yield, dr > 20:1) of **42** as a yellow oil. ^{1}H NMR (400 MHz, $CDCl_3$) δ 7.49–7.31 (m, 3H), 7.23 (dd, J = 7.0, 1.8 Hz, 2H), 6.09 (td, J = 55.2, 4.7 Hz, 1H), 5.58 (td, J = 55.5, 6.6 Hz, 1H), 3.38 (d, J = 13.6 Hz, 1H), 3.15 (dt, J = 13.6, 1.3 Hz, 1H), 2.95–2.81 (m, 1H), 2.55–2.22 (m, 2H), 1.16 (s, 3H), 0.87 (s, 3H); ^{13}C NMR (151 MHz, $CDCl_3$) δ 214.4, 135.7, 128.9, 128.3, 127.5, 114.5 (tdd, J = 244.0, 9.6, 4.6 Hz), 114.2 (tt, J = 243.1, 7.5 Hz), 55.5, 53.6, 47.0 (quintet, J = 4.1 Hz), 46.7 (quintet, J = 20.4 Hz), 38.3, 27.7, 22.4; ^{19}F NMR (377 MHz, $CDCl_3$) δ -118.2 – -122.0 (m), -119.5 – -121.5 (m); HRMS (ESI) m/z: $[M + H]^+$ Calcd for $C_{16}H_{18}F_4O+H^+$: 303.1367; Found: 303.1368.

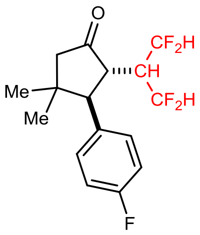

43

Compound ***43*****:** 58 mg, 91% yield, dr > 20:1, yellow oil; Flash column chromatography conditions: PE/EtOAc = 50:1. ^{1}H NMR (400 MHz, $CDCl_3$) δ 7.18 (dd, J = 8.5, 5.3 Hz, 2H), 7.07 (t, J = 8.6 Hz, 2H), 6.06 (td, J = 55.2, 4.7 Hz, 1H), 5.58 (td, J = 55.5, 6.5 Hz, 1H), 3.34 (d, J = 13.6 Hz, 1H), 3.05 (d, J = 13.7 Hz, 1H), 2.93–2.80 (m, 2H), 2.50–2.25 (m, 2H), 1.12 (s, 3H), 0.83 (s, 3H); ^{13}C NMR (151 MHz, $CDCl_3$) δ 214.0, 162.2 (d, J = 246.4 Hz), 131.4 (d, J = 3.3 Hz), 130.4, 114.4 (tdd, J = 244.0, 9.7, 4.7 Hz), 114.2 (tt, J = 243.1, 7.3 Hz), 115.3 (d, J = 21.2 Hz), 54.8, 53.4, 47.1 (quintet, J = 4.2 Hz), 46.6 (quintet, J = 20.2 Hz), 38.3, 27.6, 22.2; ^{19}F NMR (377 MHz, $CDCl_3$) δ -114.9, -118.1 – -122.0 (m), -119.6 – -121.6 (m); HRMS (ESI) *m/z*: [*M* + H]$^+$ Calcd for $C_{16}H_{17}F_5O+H^+$: 321.1272; Found: 321.1283.

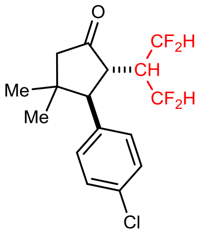

44

Compound ***44*****:** 58 mg, 86% yield, dr > 20:1, yellow oil; Flash column chromatography conditions: PE/EtOAc = 50:1. ^{1}H NMR (400 MHz, $CDCl_3$) δ 7.35 (d, J = 8.4 Hz, 2H), 7.15 (d, J = 8.5 Hz, 2H), 6.06 (td, J = 55.2, 4.7 Hz, 1H), 5.58 (td, J = 55.5, 6.5 Hz, 1H), 3.33 (d, J = 13.6 Hz, 1H), 3.06 (dt, J = 13.6, 1.4 Hz, 1H), 2.94–2.80 (m, 1H), 2.54–2.25 (m, 2H), 1.12 (s, 3H), 0.83 (s, 3H); ^{13}C NMR (151 MHz, $CDCl_3$) δ 213.9, 134.3, 133.5, 130.2, 128.6, 114.4 (tdd, J = 244.2, 9.7, 4.8 Hz), 114.1 (tt, J = 243.2, 7.5 Hz), 54.9, 53.4, 46.9 (quintet, J = 4.0 Hz), 46.6 (quintet, J = 20.2 Hz), 38.3, 27.6, 22.3; ^{19}F NMR (377 MHz, $CDCl_3$) δ -118.1 – -122.0 (m), -119.6 – -121.6 (m); HRMS (ESI) *m/z*: [*M* + H]$^+$ Calcd for $C_{16}H_{17}ClF_4O+Na^+$: 359.0796; Found: 359.0797.

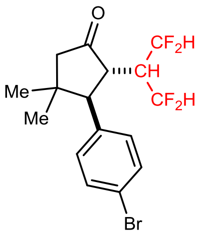

45

Compound ***45*****:** 65 mg, 86% yield, dr > 20:1, yellow oil; Flash column chromatography conditions: PE/EtOAc = 50:1. ^{1}H NMR (400 MHz, $CDCl_3$) δ 7.50 (d, J = 8.4 Hz, 2H), 7.09 (d, J = 8.3 Hz, 2H), 6.06 (td, J = 55.2, 4.7 Hz, 1H), 5.58 (td, J = 55.5, 6.6 Hz, 1H), 3.32 (d, J = 13.6 Hz, 1H), 3.16–3.01 (m, 1H), 2.51–2.25 (m, 2H), 1.12 (s, 3H), 0.83 (s, 3H); ^{13}C NMR (151 MHz, $CDCl_3$) δ 213.8, 134.8, 131.5, 130.5, 121.5, 114.4 (tdd, J = 244.3, 9.7, 4.8 Hz), 114.1 (tt, J = 243.2, 7.4 Hz), 55.0, 53.4, 46.8 (quintet, J = 4.1 Hz), 46.7 (quintet, J = 20.3 Hz), 38.3, 27.6, 22.2; ^{19}F NMR (377 MHz, $CDCl_3$) δ -118.0 – -122.0 (m), -119.6 – -121.6 (m); HRMS (ESI) *m*/*z*: [*M* + H]$^+$ Calcd for $C_{16}H_{17}BrF_4O+H^+$: 381.0472; Found: 381.0470.

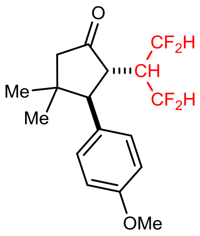

46

Compound ***46*****:** 55 mg, 83% yield, dr > 20:1, yellow oil; Flash column chromatography conditions: PE/EtOAc = 50:1. ^{1}H NMR (400 MHz, $CDCl_3$) δ 7.13 (d, J = 8.7 Hz, 2H), 6.90 (d, J = 8.7 Hz, 2H), 6.05 (td, J = 55.2, 4.7 Hz, 1H), 5.57 (td, J = 55.6, 6.7 Hz, 1H), 3.82 (s, 3H), 3.29 (d, J = 13.6 Hz, 1H), 3.05 (d, J = 13.7 Hz, 1H), 2.95–2.82 (m, 1H), 2.55–2.25 (m, 2H), 1.11 (s, 3H), 0.83 (s, 3H); ^{13}C NMR (151 MHz, $CDCl_3$) δ 214.6, 159.0, 129.9, 127.5, 114.5 (tdd, J = 244.1, 10.0, 4.9 Hz), 114.2 (tt, J = 242.9, 7.3 Hz), 113.7, 55.2, 54.7, 53.5, 47.1 (quintet, J = 4.1 Hz), 46.7 (quintet, J = 20.2 Hz), 38.3, 27.7, 22.3; ^{19}F NMR (377 MHz, $CDCl_3$) δ -118.2 – -122.0 (m), -119.6 – -121.5 (m); HRMS (ESI) *m*/*z*: [*M* + H]$^+$ Calcd for $C_{17}H_{20}F_4O_2+H^+$: 333.1472; Found: 333.1474.

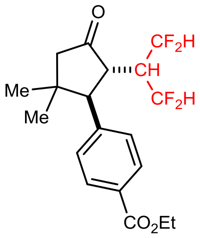

47

Compound ***47*****:** 57 mg, 76% yield, dr > 20:1, yellow oil; Flash column chromatography conditions: PE/EtOAc = 50:1. ^{1}H NMR (400 MHz, $CDCl_3$) δ 8.06 (d, J = 8.2 Hz, 2H), 7.30 (d, J = 8.0 Hz, 2H), 6.06 (td, J = 55.1, 4.7 Hz, 1H), 5.56 (td, J = 55.5, 6.4 Hz, 1H), 4.39 (q, J = 7.0 Hz, 2H), 3.43 (d, J = 13.5 Hz, 1H), 3.15 (dd, J = 13.5, 2.1 Hz, 1H), 3.06–2.69 (m, 1H), 2.56–2.28 (m, 2H), 1.41 (t, J = 7.1 Hz, 3H), 1.14 (s, 3H), 0.84 (s, 3H); ^{13}C NMR (151 MHz, $CDCl_3$) δ 213.7, 166.2, 141.1, 129.9, 129.5, 128.9, 114.4 (tdd, J = 243.9, 7.7, 5.2 Hz), 114.1 (tt, J = 243.2, 7.6 Hz), 61.0, 55.4, 53.4, 46.8 (quintet, J = 4.0 Hz), 46.7 (quintet, J = 20.1 Hz), 38.5, 27.7, 22.4, 14.3; ^{19}F NMR (377 MHz, $CDCl_3$) δ -118.2 – -122.0 (m), -119.6 – -121.6 (m); HRMS (ESI) *m*/*z*: $[M + H]^+$ Calcd for $C_{19}H_{22}F_4O_3+H^+$: 375.1578; Found: 375.1577.

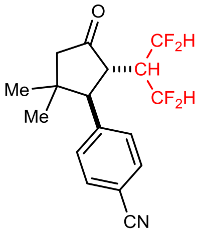

48

Compound ***48*****:** 56 mg, 86% yield, dr > 20:1, yellow oil; Flash column chromatography conditions: PE /EtOAc = 50:1. ^{1}H NMR (400 MHz, $CDCl_3$) δ 7.69 (d, J = 8.3 Hz, 2H), 7.34 (d, J = 8.1 Hz, 2H), 6.08 (td, J = 55.1, 4.8 Hz, 1H), 5.59 (td, J = 55.4, 6.3 Hz, 1H), 3.42 (d, J = 13.5 Hz, 1H), 3.12 (d, J = 13.6 Hz, 1H), 2.94–2.80 (m, 1H), 2.61–2.14 (m, 2H), 1.14 (s, 3H), 0.84 (s, 3H); ^{13}C NMR (151 MHz, $CDCl_3$) δ 213.0, 141.6, 132.1, 129.6, 118.4, 114.2 (tdd, J = 244.0, 9.7, 4.7 Hz), 114.0 (tt, J = 243.1, 7.3 Hz), 111.7, 55.6, 53.3, 46.7 (quintet, J = 20.2 Hz), 46.6 (quintet, J = 4.1 Hz), 38.7, 27.6, 22.3; ^{19}F NMR (377 MHz, $CDCl_3$) δ -117.8 – -122.0 (m), -119.5 – -121.7 (m); HRMS (ESI) *m*/*z*: $[M + H]^+$ Calcd for $C_{17}H_{17}F_4NO+H^+$: 328.1319; Found: 328.1318.

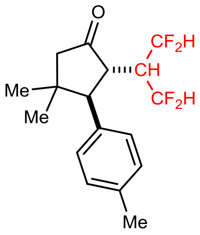

49

*Compound **49**:* 52 mg, 83% yield, dr > 20:1, yellow oil; Flash column chromatography conditions: PE/EtOAc = 50:1. ^{1}H NMR (400 MHz, $CDCl_3$) δ 7.17 (d, J = 7.8 Hz, 2H), 7.09 (d, J = 7.9 Hz, 2H), 6.05 (td, J = 55.2, 4.7 Hz, 1H), 5.57 (td, J = 55.6, 6.6 Hz, 1H), 3.31 (d, J = 13.6 Hz, 1H), 3.09 (dt, J = 13.7, 1.4 Hz, 1H), 2.96–2.57 (m, 1H), 2.44–2.33 (m, 2H), 2.36 (s, 3H), 1.12 (s, 3H), 0.83 (s, 3H); ^{13}C NMR (151 MHz, $CDCl_3$) δ 214.6, 137.2, 132.5, 129.0, 128.8, 114.4 (tdd, J = 244.4, 10.0, 4.8 Hz), 114.2 (tt, J = 242.9, 7.4 Hz), 55.1, 53.6, 47.0 (quintet, J = 3.9 Hz), 46.6 (quintet, J = 20.3 Hz), 38.3, 27.7, 22.3, 21.0; ^{19}F NMR (377 MHz, $CDCl_3$) δ -118.2 – -122.0 (m), -119.6 – -121.5 (m); HRMS (ESI) m/z: $[M + H]^+$ Calcd for $C_{17}H_{20}F_4O+H^+$: 317.1523; Found: 317.1523.

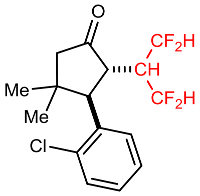

50

*Compound **50**:* 46 mg, 68% yield, dr > 20:1, yellow oil; Flash column chromatography conditions: PE/EtOAc = 50:1. ^{1}H NMR (400 MHz, $CDCl_3$) δ 7.51–7.46 (m, 1H), 7.35–7.23 (m, 3H), 6.05 (td, J = 55.1, 5.2 Hz, 1H), 5.60 (td, J = 55.4, 6.0 Hz, 1H), 4.18 (d, J = 13.6 Hz, 1H), 3.09 (dd, J = 13.6, 2.1 Hz, 1H), 2.97–2.74 (m, 1H), 2.47 (s, 2H), 1.21 (s, 3H), 0.94 (s, 3H); ^{13}C NMR (151 MHz, $CDCl_3$) δ 213.9, 135.9, 133.7, 130.3, 128.9, 128.6, 126.4, 114.2 (tdd, J = 243.9, 8.9, 5.4 Hz), 113.9 (tt, J = 243.4, 7.4 Hz), 53.7, 48.9, 47.6 (quintet, J = 4.4 Hz), 46.5 (quintet, J = 20.2 Hz), 39.7, 27.9, 22.8; ^{19}F NMR (377 MHz, $CDCl_3$) δ -118.8 – -122.1 (m), -119.6 – -121.7 (m); HRMS (ESI) m/z: $[M + H]^+$ Calcd for $C_{16}H_{17}ClF_4O+H^+$: 337.0977; Found: 337.0976.

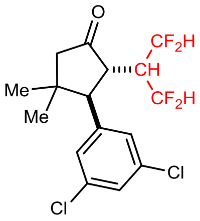

51

Compound ***51*****:** 53 mg, 72% yield, dr > 20:1, yellow oil; Flash column chromatography conditions: PE/EtOAc = 50:1. ^{1}H NMR (400 MHz, $CDCl_3$) δ 7.34 (s, 1H), 7.10 (d, J = 1.8 Hz, 2H), 6.09 (td, J = 55.2, 4.8 Hz, 1H), 5.66 (td, J = 55.4, 6.3 Hz, 1H), 3.30 (d, J = 13.5 Hz, 1H), 3.03 (d, J = 13.5 Hz, 1H), 2.91–2.78 (m, 1H), 2.48–2.27 (m, 2H), 1.16 (s, 3H), 0.86 (s, 3H); ^{13}C NMR (151 MHz, $CDCl_3$) δ 213.0, 139.6, 135.1, 127.3, 114.2 (tdd, J = 244.2, 9.5, 4.9 Hz), 114.1 (tt, J = 243.1, 7.6 Hz), 55.1, 53.3, 46.7 (quintet, J = 3.3 Hz), 46.5 (quintet, J = 20.2 Hz), 38.5, 27.7, 22.3; ^{19}F NMR (377 MHz, $CDCl_3$) δ -117.7 – -122.0 (m), -119.5 – -121.7 (m); HRMS (ESI) *m*/*z*: [*M* + H]$^+$ Calcd for $C_{16}H_{16}Cl_2F_4O+Na^+$: 393.0407; Found: 393.0406.

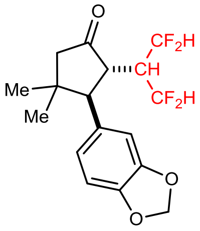

52

Compound ***52*****:** 55 mg, 79% yield, dr > 20:1, yellow oil; Flash column chromatography conditions: PE/EtOAc = 50:1. ^{1}H NMR (400 MHz, $CDCl_3$) δ 6.81 (d, J = 7.9 Hz, 1H), 6.73–6.63 (m, 2H), 6.07 (td, J = 55.2, 4.8 Hz, 1H), 5.98 (s, 2H), 5.64 (td, J = 55.6, 6.6 Hz, 1H), 3.26 (d, J = 13.6 Hz, 1H), 3.00 (dt, J = 13.7, 1.4 Hz, 1H), 2.92–2.79 (m, 1H), 2.42–2.31 (m, 2H), 1.13 (s, 3H), 0.85 (s, 3H); ^{13}C NMR (151 MHz, $CDCl_3$) δ 214.3, 147.7, 147.0, 129.4, 122.2, 114.4 (tdd, J = 244.2, 10.0, 4.8 Hz), 114.3 (tt, J = 243.2, 7.3 Hz), 109.0, 108.1, 101.1, 55.3, 53.5, 47.2 (quintet, J = 3.9 Hz), 46.7 (quintet, J = 20.2 Hz), 38.3, 27.8, 22.4; ^{19}F NMR (377 MHz, $CDCl_3$) δ -118.1 – -122.0 (m), -119.5 – -121.5 (m); HRMS (ESI) *m*/*z*: [*M* + H]$^+$ Calcd for $C_{17}H_{18}F_4O_3+H^+$: 347.1265; Found: 347.1265.

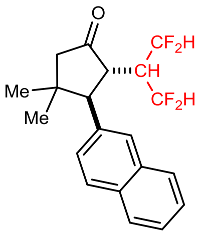

53

Compound ***53*****:** 32 mg, 45% yield, dr > 20:1, yellow oil; Flash column chromatography conditions: PE/EtOAc = 50:1. ^{1}H NMR (400 MHz, $CDCl_3$) δ 7.96–7.79 (m, 3H), 7.71 (d, J = 1.8 Hz, 1H), 7.59–7.49 (m, 2H), 7.37 (dd, J = 8.5, 1.8 Hz, 1H), 6.10 (td, J = 55.2, 4.7 Hz, 1H), 5.58 (td, J = 55.6, 6.7 Hz, 1H), 3.56 (d, J = 13.6 Hz, 1H), 3.29 (dd, J = 13.6, 2.1 Hz, 1H), 3.07–2.85 (m, 1H), 2.48 (dd, J = 2.7 Hz, 2H), 1.21 (s, 3H), 0.92 (s, 3H); ^{13}C NMR (101 MHz, $CDCl_3$) δ 214.4, 133.3, 133.2, 132.8, 128.1, 127.9, 127.8, 127.6, 126.6, 126.3, 126.1, 114.5 (tdd, J = 366.3, 9.5, 4.4 Hz), 114.2 (tt, J = 364.5, 7.5 Hz), 55.6, 53. 6, 47.0 (quintet, J = 3.7 Hz), 46.7 (quintet, J = 30.3 Hz), 38.7, 27.8, 22.5; ^{19}F NMR (377 MHz, $CDCl_3$) δ -118.2 – -122.0 (m), -119.5 – -121.5 (m); HRMS (ESI) m/z: $[M + H]^+$ Calcd for $C_{20}H_{20}F_4O+H^+$: 353.1523; Found: 353.1523.

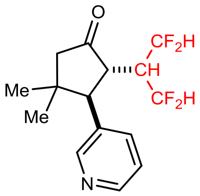

54

Compound ***54*****:** 40 mg, 66% yield, dr > 20:1, yellow oil; Flash column chromatography conditions: PE/EtOAc = 1:1. ^{1}H NMR (400 MHz, $CDCl_3$) δ 8.78–8.33 (m, 2H), 7.55 (d, J = 8.0 Hz, 1H), 7.34 (dd, J = 8.0, 4.7 Hz, 1H), 6.08 (td, J = 55.2, 4.8 Hz, 1H), 5.62 (td, J = 55.4, 6.2 Hz, 1H), 3.37 (d, J = 13.5 Hz, 1H), 3.13 (d, J = 13.6 Hz, 1H), 2.96–2.72 (m, 1H), 2.64–2.27 (m, 2H), 1.15 (s, 3H), 0.86 (s, 3H); ^{13}C NMR (151 MHz, $CDCl_3$) δ 213.4, 150.6, 149.1, 135.7, 131.5, 123.2, 114.2 (tdd, J = 244.3, 9.5, 5.1 Hz), 114.0 (tt, J = 242.6, 7.1 Hz), 53.3, 53.0, 46.7 (quintet, J = 20.0 Hz), 46.4 (quintet, J = 3.6 Hz), 38.4, 27.5, 22.2; ^{19}F NMR (377 MHz, $CDCl_3$) δ -118.0 – -122.0 (m), -119.6 – -121.7 (m); HRMS (ESI) m/z: $[M + H]^+$ Calcd for $C_{15}H_{17}F_4NO+H^+$: 304.1319; Found: 304.1318.

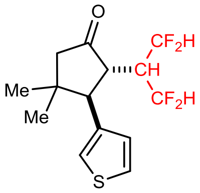

55

Compound ***55*****:** 37 mg, 60% yield, dr > 20:1, yellow oil; Flash column chromatography conditions: PE/EtOAc = 50:1. ^{1}H NMR (600 MHz, $CDCl_3$) δ 7.35 (dd, J = 5.0, 2.9 Hz, 1H), 7.09 (dd, J = 3.0, 1.3 Hz, 1H), 6.97 (dd, J = 5.0, 1.3 Hz, 1H), 6.07 (td, J = 55.2, 4.6 Hz, 1H), 5.59 (td, J = 55.6, 6.7 Hz, 1H), 3.47 (d, J = 13.4 Hz, 1H), 3.01 (dt, J = 13.4, 1.6 Hz, 1H), 2.96–2.86 (m, 1H), 2.50–2.24 (m, 2H), 1.17 (s, 3H), 0.85 (s, 3H); ^{13}C NMR (151 MHz, $CDCl_3$) δ 214.2, 137.3, 127.6, 125.7, 122.8, 114.5 (tdd, J = 244.3, 10.2, 4.6 Hz), 114.3 (tt, J = 243.0, 7.3 Hz), 53.3, 51.1, 48.0 (quintet, J = 4.1 Hz), 46.6 (quintet, J = 20.3 Hz), 38.2, 27.8, 22.6; ^{19}F NMR (377 MHz, $CDCl_3$) δ -118.1 – -122.0 (m), -119.6 – -121.6 (m); HRMS (ESI) *m*/*z*: [*M* + H]$^+$ Calcd for $C_{14}H_{16}F_4OS+H^+$: 309.0931; Found: 309.0933.

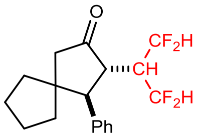

56

Compound ***56*****:** 48 mg, 73% yield, dr > 20:1, yellow oil; Flash column chromatography conditions: PE/EtOAc = 100:1. ^{1}H NMR (400 MHz, $CDCl_3$) δ 7.43–7.28 (m, 3H), 7.25–7.10 (m, 2H), 6.07 (td, J = 55.2, 4.8 Hz, 1H), 5.59 (td, J = 55.6, 6.6 Hz, 1H), 3.59 (d, J = 13.7 Hz, 1H), 3.08 (dt, J = 13.8, 1.6 Hz, 1H), 3.00–2.72 (m, 1H), 2.56 (d, J = 18.3 Hz, 1H), 2.27 (d, J = 18.3 Hz, 1H), 1.80–1.30 (m, 8H); ^{13}C NMR (151 MHz, $CDCl_3$) δ 214.4, 135.7, 129.5, 128.3, 127.5, 114.4 (tdd, J = 244.1, 9.9, 4.8 Hz), 114.3 (tt, J = 243.1, 7.4 Hz), 52.9, 51.7, 49.9, 48.3 (quintet, J = 4.1 Hz), 46.5 (quintet, J = 20.1 Hz), 36.4, 30.7, 23.1, 22.8; ^{19}F NMR (377 MHz, $CDCl_3$) δ -118.0 – -122.0 (m), -119.5 – -121.6 (m); HRMS (ESI) *m*/*z*: [*M* + H]$^+$ Calcd for $C_{18}H_{20}F_4O+H^+$: 329.1523; Found: 329.1523.

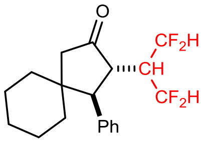

57

Compound ***57*****:** 51 mg, 75% yield, dr > 20:1, yellow oil; Flash column chromatography conditions: PE/EtOAc = 100:1. ^{1}H NMR (400 MHz, $CDCl_3$) δ 7.34 (dq, J = 14.1, 7.2 Hz, 3H), 7.19 (d, J = 7.1 Hz, 2H), 6.05 (td, J = 55.2, 4.7 Hz, 1H), 5.55 (td, J = 55.6, 6.6 Hz, 1H), 3.28 (d, J = 13.9 Hz, 1H), 3.16 (d, J = 13.9 Hz, 1H), 3.03–2.73 (m, 1H), 2.82 (d, J = 18.6 Hz, 1H), 2.16 (dd, J = 18.6, 2.0 Hz, 1H), 1.78–1.14 (m, 8H), 1.10–0.80 (m, 2H); ^{13}C NMR (151 MHz, $CDCl_3$) δ 214.5, 135.5, 129.5, 128.2, 127.5, 114.4 (tdd, J = 244.1, 10.2, 4.7 Hz), 114.3 (tt, J = 242.9, 7.3 Hz), 56.7, 48.0, 46.5 (quintet, J = 20.2 Hz), 46.4 (quintet, J = 4.2 Hz), 42.0, 37.4, 29.3, 25.7, 23.7, 22.0; ^{19}F NMR (377 MHz, $CDCl_3$) δ -118.0 – -122.0 (m), -119.5 – -121.5 (m); HRMS (ESI) *m*/*z*: [*M* + H]$^+$ Calcd for $C_{19}H_{22}F_4O+H^+$: 343.1680; Found: 343.1678.

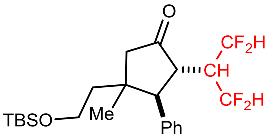

58

Compound ***58***: 53 mg, 59% yield, dr = 2:1, yellow oil; Flash column chromatography conditions: PE/EtOAc = 50:1. ^{1}H NMR (400 MHz, $CDCl_3$) data of major isomer δ 7.43–7.30 (m, 3H), 7.27–7.21 (m, 2H), 6.09 (td, J = 55.2, 4.7 Hz, 1H), 5.58 (td, J = 55.5, 6.6 Hz, 1H), 3.78–3.54 (m, 2H), 3.41 (t, J = 14.8 Hz, 1H), 3.21–3.09 (m, 1H), 2.94–2.81 (m, 1H), 2.59–2.46 (m, 2H), 1.73–1.54 (m, 2H), 0.90 (s, 9H), 0.89 (s, 3H), 0.05 (s, 6H); ^{13}C NMR (101 MHz, $CDCl_3$) data of major isomer δ 214.9, 135.8, 129.2, 128.3, 127.6, 114.4 (tdd, J = 368.2, 9.7, 4.7 Hz), 114.2 (tt, J = 364.9, 7.3 Hz), 59.8, 55.1, 52.0, 46.5 (quintet, J = 20.3 Hz), 46.1 (quintet, J = 4.2 Hz), 42.7, 40.4, 25.9, 19.9, 18.2, -5.5; ^{19}F NMR (377 MHz, $CDCl_3$) data of major isomer δ -117.8 – -121.7 (m), -118.6 – -121.0 (m), -119.5 – -121.6 (m), -120.2 – -120.8 (m); HRMS (ESI) *m*/*z*: Calcd for $C_{23}H_{34}F_4O_2Si$ [*M* + H]$^+$ 447.2337; Found: 447.2335.

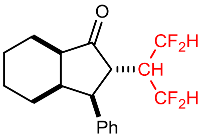

59

Compound ***59***: 35 mg, 54% yield, dr > 20:1, yellow oil; Flash column chromatography conditions: PE/EtOAc = 50:1. ^{1}H NMR (600 MHz, $CDCl_3$) δ 7.37–7.35 (m, 2H), 7.29–7.27 (m, 1H), 7.24–7.23 (m, 2H), 6.14 (td, J = 55.3, 5.0 Hz, 1H), 5.65 (td, J = 55.4, 6.4 Hz, 1H), 3.71 (dd, J = 12.9, 5.9 Hz, 1H), 3.05 (dd, J = 12.9, 2.3 Hz, 1H), 2.99–2.89 (m, 1H), 2.66 (t, J = 6.8 Hz, 1H), 2.49–2.43 (m, 1H), 2.30–2.67 (m, 1H), 1.65–1.62 (m, 1H), 1.56–1.55 (m, 1H), 1.50–1.44 (m, 1H), 1.21– 1.70 (m, 1H), 1.05–0.95 (m, 2H), 0.90–0.83 (m, 1H); ^{13}C NMR (151 MHz, $CDCl_3$) δ 216.1, 137.5, 128.6, 128.0, 127.2, 114.6 (tdd, J = 244.1, 9.4, 5.4 Hz), 114.4 (tt, J = 243.3, 7.5 Hz), 50.9, 47.6, 47.2 (quintet, J = 20.1 Hz), 44.2 (quintet, J = 3.3 Hz), 41.2, 24.5, 24.2, 23.0, 22.7; ^{19}F NMR (377 MHz, $CDCl_3$) δ -118.2 – -121.4 (m), -119.8 – -121.5 (m); HRMS (ESI) *m/z*: [*M* + Na]$^+$ Calcd for $C_{18}H_{20}F_4O$+Na$^+$: 351.1342; Found: 351.1342.

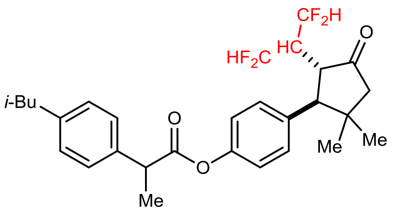

60

Compound ***60*****:** 61 mg, 60% yield, dr = 1:1, yellow oil; Flash column chromatography conditions: PE/EtOAc = 50:1. ^{1}H NMR (400 MHz, $CDCl_3$) δ 7.30 (d, J = 7.9 Hz, 2H), 7.20–7.11 (m, 4H), 7.08–6.95 (m, 2H), 6.04 (td, J = 55.2, 4.7 Hz, 1H), 5.56 (td, J = 55.5, 6.6 Hz, 1H), 3.94 (q, J = 7.1 Hz, 1H), 3.33 (d, J = 13.6 Hz, 1H), 3.17–3.04 (m, 1H), 2.95–2.73 (m, 1H), 2.47 (d, J = 7.2 Hz, 2H), 2.45–2.28 (m, 2H), 1.92–1.82 (m, 1H), 1.61 (d, J = 7.1 Hz, 3H), 1.10 (s, 3H), 0.91 (d, J = 6.6 Hz, 6H), 0.81 (s, 3H); ^{13}C NMR (151 MHz, $CDCl_3$) δ 214.2, 173.2, 150.2, 140.9, 137.1, 133.1, 129.7, 129.5, 127.2, 121.3, 114.4 (tdd, J = 244.0, 9.7, 4.7 Hz), 114.1 (tt, J = 243.1, 7.3 Hz), 54.9, 53.5, 47.0 (quintet, J = 3.1 Hz), 46.6 (quintet, J = 20.3 Hz), 45.2, 45.0, 38.3, 30.2, 27.6, 22.4, 22.2, 18.4; ^{19}F NMR (377 MHz, $CDCl_3$) δ -118.0 – -122.0 (m), -119.6 – -121.6 (m); HRMS (ESI) *m/z*: [*M* + H]$^+$ Calcd for $C_{29}H_{34}F_4O_3$+H$^+$: 507.2517; Found: 507.2530.

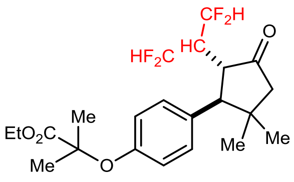

61

*Compound **61***: 61 mg, 71% yield, dr > 20:1, yellow oil; Flash column chromatography conditions: PE/EtOAc = 50:1. ^{1}H NMR (400 MHz, $CDCl_3$) δ 7.07 (d, J = 8.6 Hz, 2H), 6.83 (d, J = 8.6 Hz, 2H), 6.04 (td, J = 55.2, 4.6 Hz, 1H), 5.55 (td, J = 55.6, 6.6 Hz, 1H), 4.27–4.21 (m, 2H), 3.27 (d, J = 13.6 Hz, 1H), 3.19–2.96 (m, 1H), 2.94–2.79 (m, 1H), 2.42–2.31 (m, 2H), 1.62 (s, 6H), 1.22 (td, J = 7.1, 1.1 Hz, 3H), 1.10 (s, 3H), 0.82 (s, 3H); ^{13}C NMR (151 MHz, $CDCl_3$) δ 214.4 (d, J = 4.1 Hz), 174.1, 154.9, 129.6, 128.9, 118.5, 114.4 (tdd, J = 243.9, 10.0, 4.4 Hz), 114.2 (tt, J = 243.1, 7.4 Hz), 79.1, 61.4, 54.7, 53.4, 47.1 (quintet, J = 4.0 Hz), 46.6 (quintet, J = 20.2 Hz), 38.2, 27.6, 25.4, 25.4, 22.3, 14.0; ^{19}F NMR (377 MHz, $CDCl_3$) δ -118.2 – -122.0 (m), -119.6 – -121.6 (m); HRMS (ESI) m/z: $[M + H]^+$ Calcd for $C_{22}H_{28}F_4O_4+H^+$: 433.1996; Found: 433.2016.

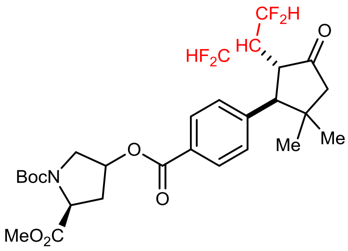

62

*Compound **62***: 78 mg, 66% yield, dr = 1.4:1, yellow oil; Flash column chromatography conditions: PE/EtOAc = 25:1. ^{1}H NMR (400 MHz, $CDCl_3$) δ 8.03 (d, J = 8.0 Hz, 2H), 7.32 (d, J = 8.0 Hz, 2H), 6.07 (td, J = 55.1, 4.6 Hz, 1H), 5.60–5.58 (m, 1H), 5.55 (td, J = 55.6, 6.6 Hz, 1H), 4.54 (t, J = 7.8 Hz, 0.42H), 4.45 (t, J = 8.1 Hz, 0.58H), 3.88–3.84 (m, 2H), 3.78 (s, 1.25H), 3.77 (s, 1.75H), 3.44 (d, J = 13.5 Hz, 1H), 3.16 (d, J = 13.0 Hz, 1H), 2.97–2.81 (m, 1H), 2.60–2.51 (m, 1H), 2.48–2.39 (m, 2H), 2.36–2.29 (m, 1H), 1.47 (s, 3.80H), 1.44 (s, 5.20H), 1.15 (s, 3H), 0.85 (s, 3H); ^{13}C NMR (101 MHz, $CDCl_3$) δ 213.6, 213.5, 173.0, 172.7, 165.5, 165.4, 154.2, 153.5, 141.8, 141.7, 129.7, 129.6, 129.0, 128.9, 114.3 (tdd, J = 364.7, 12.4, 8.1 Hz), 114.2 (tt, J = 364.7, 12.9 Hz), 80.6, 80.5, 73.3, 72.6, 57.9, 57.5, 55.4, 53.3, 52.1, 46.8 (quintet, J = 3.3 Hz), 46.6 (quintet, J = 30.0 Hz), 38.5,

36.6, 35.6, 28.2, 28.1, 27.6, 22.3; ^{19}F NMR (377 MHz, $CDCl_3$) δ -118.2 – -122.0 (m), -119.6 – -121.6 (m); HRMS (ESI) *m*/*z*: [*M* + Na]$^+$ Calcd for $C_{28}H_{35}F_4NO_7$+Na$^+$: 596.2242; Found: 596.2238.

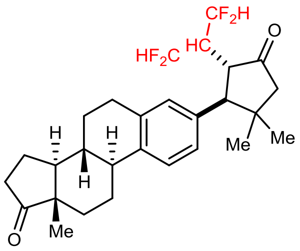

63

Compound ***63*****:** 62 mg, 65% yield, dr = 1:1, yellow oil; Flash column chromatography conditions: PE/EtOAc = 10:1. ^{1}H NMR (400 MHz, $CDCl_3$) δ 7.26 (d, *J* = 7.4 Hz, 1H), 6.97 (dd, *J* = 8.2, 2.0 Hz, 1H), 6.91 (d, *J* = 2.0 Hz, 1H), 6.07 (td, *J* = 55.2, 4.8, 1H), 5.61 (td, *J* = 55.5, 7.4 Hz, 1H), 3.28 (d, *J* = 13.6 Hz, 1H), 3.16–3.05 (m, 1H), 2.92 (dd, *J* = 9.2, 4.2 Hz, 2H), 2.87–2.77 (m, 1H), 2.52 (dd, *J* = 18.6, 8.7 Hz, 1H), 2.47–2.26 (m, 4H), 2.22–1.90 (m, 4H), 1.75–1.40 (m, 6H), 1.13 (s, 3H), 0.94 (s, 3H), 0.84 (d, *J* = 3.8 Hz, 3H); ^{13}C NMR (151 MHz, $CDCl_3$) δ 220.7, 214.7, 139.0, 136.3, 132.9 (d, *J* = 1.7 Hz), 129.6, 126.1, 125.1 (d, *J* = 1.7 Hz), 114.4 (tdd, *J* = 244.7, 9.7, 4.7 Hz), 114.3 (tt, *J* = 243.1, 7.3 Hz), 55.04 (d, *J* = 3.8 Hz), 53.6, 50.5, 47.9, 46.9 (quintet, *J* = 4.1 Hz), 46.6 (quintet, *J* = 20.3 Hz), 44.3, 38.2, 38.0, 35.8, 31.6, 29.4, 27.8, 26.5, 25.6 (d, *J* = 6.2 Hz), 22.3 (d, *J* = 2.8 Hz), 21.6, 13.8; ^{19}F NMR (377 MHz, $CDCl_3$) δ -118.0 – -121.5 (m), -119.4 – -122.0 (m); HRMS (ESI) *m*/*z*: [*M* + H]$^+$ Calcd for $C_{28}H_{34}F_4O_2$+H$^+$: 479.2568; Found: 479.2582.

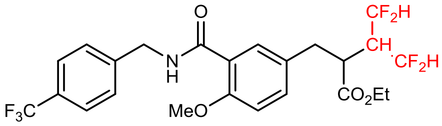

64

Compound ***64*****:** The title compound was prepared according to the general procedure except that Cs_2CO_3 was used, instead of LiOH, in 71% yield (74 mg) as a yellow oil; Flash column chromatography conditions: PE/EtOAc = 3:1. ^{1}H NMR (600 MHz, $CDCl_3$) δ 8.29 (t, *J* = 5.8 Hz, 1H), 8.10 (d, *J* = 2.5 Hz, 1H), 7.63–7.57 (m, 2H), 7.47 (d, *J* = 8.3 Hz, 2H), 7.31 (dd, *J* = 8.5, 2.5 Hz, 1H), 6.93 (d, *J* = 8.5 Hz, 1H), 6.19 (td, *J* = 54.9, 4.6 Hz, 1H), 6.12 (td, *J* = 54.9, 3.6 Hz, 1H), 4.73 (d, *J* = 5.9 Hz, 2H), 4.13–4.03 (m, 2H), 3.94 (s, 3H), 3.18 (ddd, *J* = 9.2, 6.5, 4.7 Hz, 1H), 3.11 (dd,

J = 13.7, 9.2 Hz, 1H), 3.02 (dd, J = 13.7, 6.5 Hz, 1H), 2.74–2.61 (m, 1H), 1.15 (t, J = 7.2 Hz, 3H); ^{13}C NMR (151 MHz, $CDCl_3$) δ 172.1, 165.2, 156.4, 142.9, 133.7, 132.8, 130.8, 125.5 (q, J = 3.8 Hz), 127.7, 125.5 (d, J = 3.8 Hz), 124.1 (q, J = 271.9 Hz), 121.1, 114.5 (tdd, J = 243.9, 7.5, 4.9 Hz), 114.2 (tt, J = 243.9, 7.7 Hz), 111.5, 61.2, 56.1, 47.9 (quintet, J = 19.8 Hz), 43.3, 42.1 (quintet, J = 3.1 Hz), 34.6, 13.9; ^{19}F NMR (565 MHz, $CDCl_3$) δ -62.5, -119.3 – -120.6 (m), -120.0 – -122.4 (m); HRMS (ESI) m/z: $[M + H]^+$ Calcd for $C_{24}H_{24}F_7NO_4$+H^+: 524.1666; Found: 524.1664.

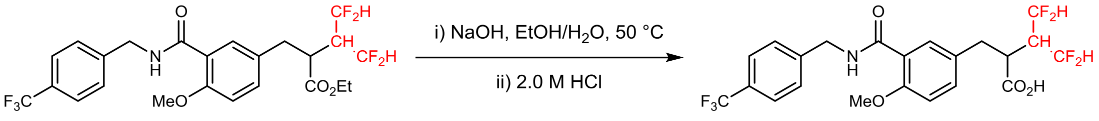

To a solution of **64** (52 mg, 0.1 mmol) in 1 mL of EtOH was added 1.5 mL of 1.0 M aqueous solution of NaOH. After stirring at 50 ºC for 4 h, EtOH was removed under reduced pressure. The resulting residue was acidified with 1 mL of 2.0 M HCl, extracted with EtOAc, dried over anhydrous Na_2SO_4, and concentrated. Column chromatography on silica gel (PE/EtOAc = 1:1) gave 47 mg (95% yield) of **65** as a yellow solid, m.p. 184–186 °C. ^{1}H NMR (600 MHz, $CDCl_3$) δ 8.42–8.36 (m, 1H), 8.09 (s, 1H), 7.57 (d, J = 8.0 Hz, 2H), 7.44 (d, J = 7.9 Hz, 2H), 7.33 (dd, J = 8.5, 2.5 Hz, 1H), 6.92 (d, J = 8.5 Hz, 1H), 6.17 (td, J = 55.0, 4.5 Hz, 1H), 6.14 (td, J = 55.0, 3.6 Hz, 1H), 4.71 (d, J = 6.0 Hz, 2H), 3.92 (s, 3H), 3.19 (ddd, J = 9.2, 6.5, 4.7 Hz, 1H), 3.14 (dd, J = 13.8, 8.9 Hz, 1H), 3.02 (dd, J = 13.7, 6.3 Hz, 1H), 2.76–2.63 (m, 1H); ^{13}C NMR (151 MHz, $CDCl_3$) δ 175.4, 165.8, 156.5, 142.5, 134.0, 132.8, 130.9, 129.5 (q, J = 32.7 Hz), 127.7, 125.5 (q, J = 3.7 Hz), 124.1 (q, J = 272.1 Hz), 120.6, 114.5 (tdd, J = 244.0, 7.4, 4.7 Hz), 114.3 (tt, J = 243.9, 7.3 Hz), 111.7, 56.1, 47.8 (quintet, J = 19.6 Hz), 43.4, 42.2 (quintet, J = 3.0 Hz), 34.3; ^{19}F NMR (565 MHz, $CDCl_3$) δ -62.5, -119.3 – -120.4 (m), -119.3 – -122.0 (m); HRMS (ESI) m/z: $[M + H]^+$ Calcd for $C_{22}H_{20}F_7NO_4$+H^+: 496.1353; Found: 496.1353.

5. Mechanistic experiments

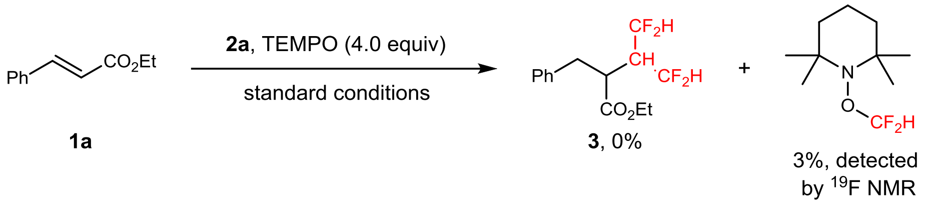

To a mixture of CF_2HSO_2Na (112 mg, 0.8 mmol), 4DPAIPN (15.9 mg, 0.02 mmol), TEMPO (125 mg, 0.8 mmol), H_2O (36.0 mg, 2.0 mmol) and LiOH (19.2 mg, 0.8 mmol) in 2 mL of MeCN was added **1a** (35.2 mg, 0.2 mmol) under a nitrogen atmosphere. After 24 h of irradiation at a distance of ~2 cm with 24 W of blue LEDs (PINO® lamps, 100% light intensity) at 25 °C, the reaction mixture was quenched with water, extracted with EtOAc, dry over anhydrous Na_2SO_4, and concentrated. ^{19}F NMR analysis with $PhCF_3$ as the internal standard showed that the TEMPO-CF_2H adduct[3] was formed in 3% yield while the product **3** was not detected. ^{19}F NMR (377 MHz, $CDCl_3$) δ -80.0 (d, J = 72.8 Hz, 2F); MS (EI) m/z: $[M]^+$ Calcd for $C_{10}H_{19}F_2NO$: 207.1; Found: 207.1.

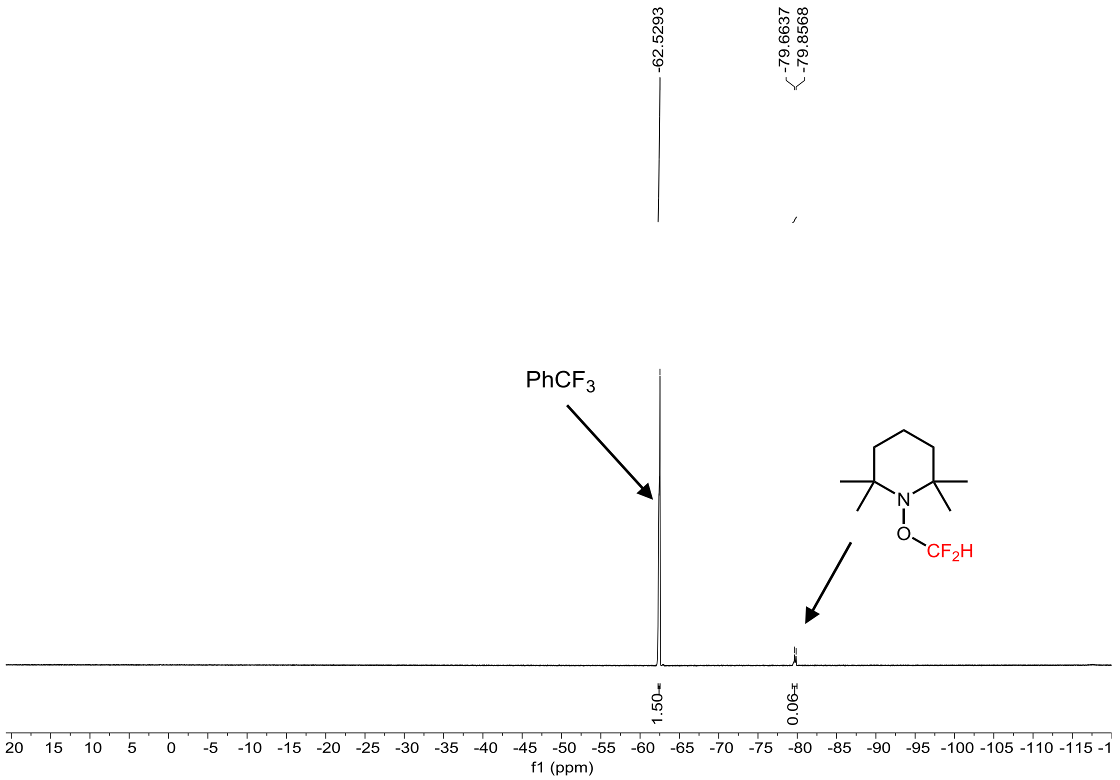

Supplementary Figure 3 ^{19}F NMR spectrum of radical trapping experiments with TEMPO.

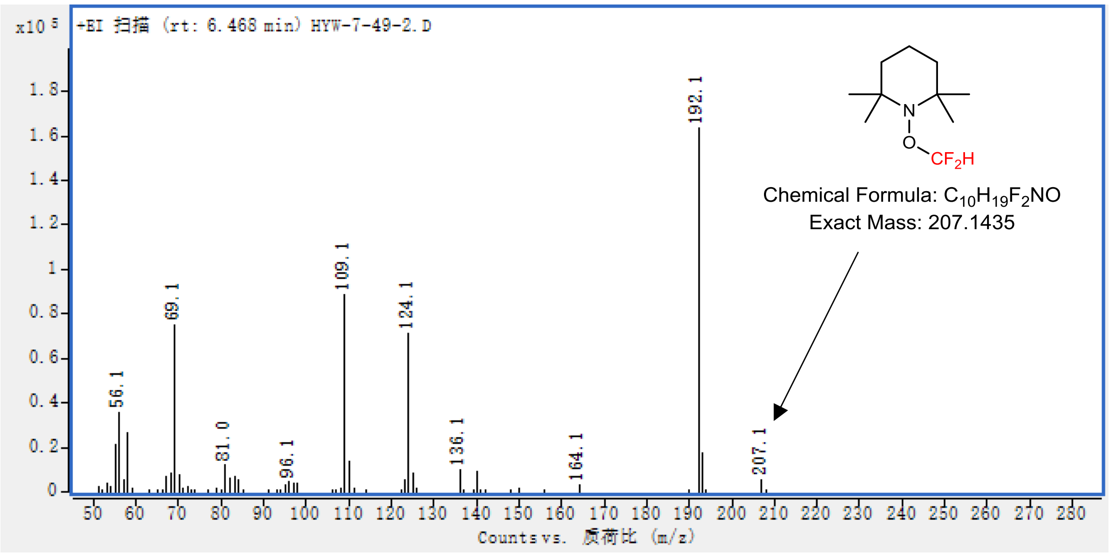

Supplementary Figure 4 GC-MS analysis of radical trapping experiments with TEMPO.

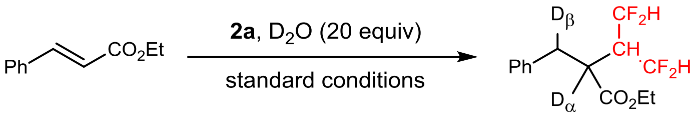

1a

[D$_2$]-**3**, 70%
D$_\alpha$: 65% D; D$_\beta$: 95% D

To a mixture of CF_2HSO_2Na (112 mg, 0.8 mmol), 4DPAIPN (15.9 mg, 0.02 mmol), D_2O (79.9 mg, 4 mmol, >98% D) and LiOH (19.2 mg, 0.8 mmol) in 2 mL of CD_3CN was added **1a** (45.6 mg, 0.2 mmol) under a nitrogen atmosphere. After 24 h of irradiation at a distance of ~2 cm with 24 W of blue LEDs (PINO® lamps, 100% light intensity) at 25 °C, the reaction mixture was quenched with water, extracted with EtOAc, washed with brine, dried over anhydrous Na_2SO_4, and concentrated. The crude ^{1}H NMR analysis indicated the respective 65% and 95% D incorporation at the α- and β-carbon atoms. Column chromatography on silica gel (PE/EtOAc = 50:1) gave 41 mg (70% yield) of [D$_2$]-**3** as a yellow oil. HRMS (ESI) *m/z*: $[M + H]^+$ Calcd for $C_{14}H_{14}D_2F_4O_2+H^+$: 295.1285; Found: 295.1278.

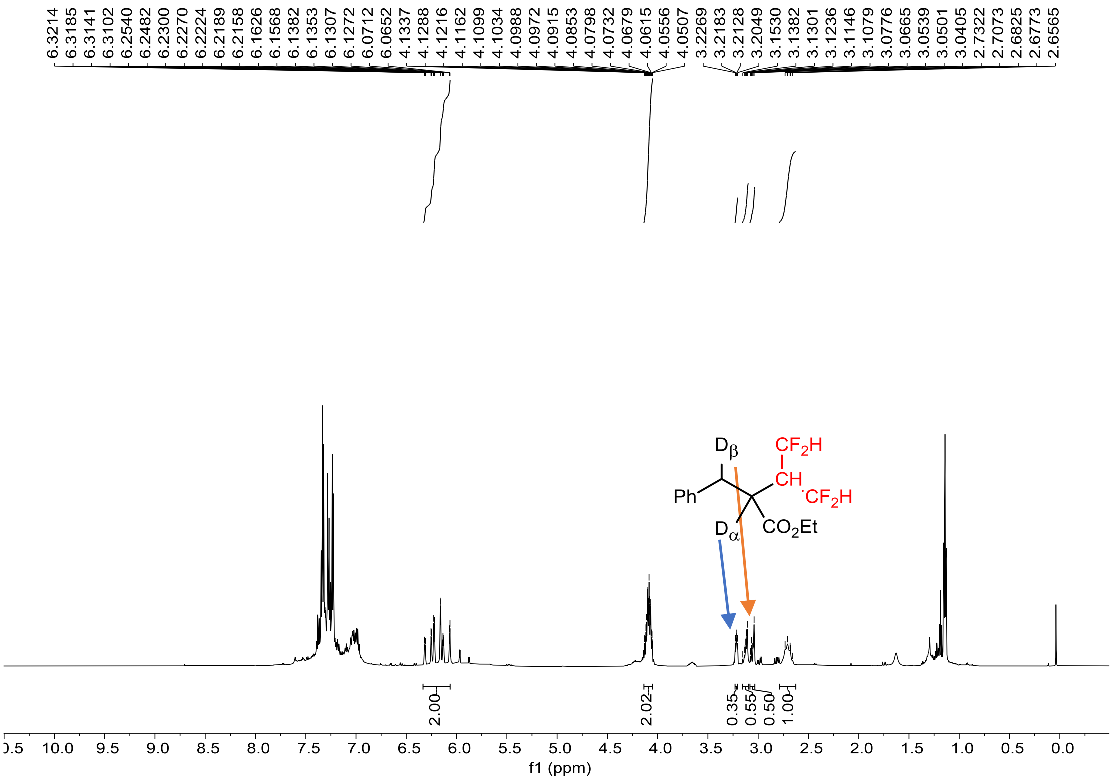

Supplementary Figure 5 ^{1}H NMR analysis of deuterium-labeling experiments with D_2O.

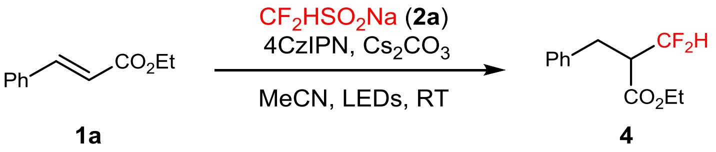

To a mixture of CF_2HSO_2Na (56 mg, 0.4 mmol), 4CzIPN (7.9 mg, 0.01 mmol), and Cs_2CO_3 (130 mg, 0.4 mmol) in 2 mL of MeCN was added **1a** (35.2 mg, 0.2 mmol) under a nitrogen atmosphere. After 12 h of irradiation at a distance of ~2 cm with 24 W of blue LEDs (PINO® lamps, 100% light intensity) at 25 °C, the reaction mixture was quenched with water, extracted with EtOAc, washed with brine, dried over anhydrous Na_2SO_4, and concentrated. Column chromatography on silica gel (PE/EtOAc = 50:1) gave 36 mg (79% yield) of **4** as a yellow oil. ^{1}H NMR (600 MHz, $CDCl_3$) δ 7.31–7.28 (m, 2H), 7.26–7.22 (m, 1H), 7.20–7.17 (m, 2H), 5.98 (td, J = 55.8, 5.6 Hz,1H), 4.09 (q, J = 7.2 Hz, 2H), 3.17–3.09 (m, 1H), 3.06–3.01 (m, 2H), 1.12 (t, J = 7.1 Hz, 3H); ^{13}C NMR (101 MHz, $CDCl_3$) δ 169.3 (dd, J = 8.8, 3.9 Hz), 136.9, 128.9, 128.6, 126.9, 115.7 (t, J = 244.4 Hz), 61.2, 52.0 (t, J = 21.5 Hz), 32.2 (dd, J = 5.7, 3.4 Hz), 13.9; ^{19}F NMR (565 MHz, $CDCl_3$) δ -118.7 – -123.9 (m); HRMS (ESI) m/z: $[M + Na]^+$ Calcd for $C_{12}H_{14}F_2O_2$+Na^+: 251.0854; Found: 251.0857.

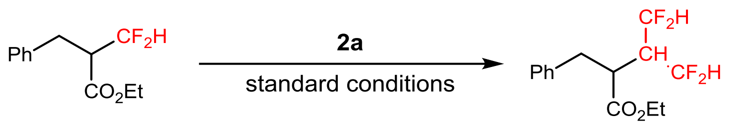

4 3

To a mixture of CF_2HSO_2Na (56 mg, 0.4 mmol), 4DPAIPN (8.0 mg, 0.01 mmol), H_2O (18.0 mg, 1.0 mmol), and LiOH (9.6 mg, 0.4 mmol) in 1 mL of MeCN was added **4** (22.8 mg, 0.1 mmol) under a nitrogen atmosphere. After 24 h of irradiation at a distance of ~2 cm with 24 W of blue LEDs (PINO® lamps, 100% light intensity) at 25 °C, the reaction mixture was quenched with water, extracted with EtOAc, washed with brine, dried over anhydrous Na_2SO_4, and concentrated. Column chromatography on silica gel (PE/EtOAc = 50:1) gave 26 mg (89% yield) of **3** as a yellow oil.

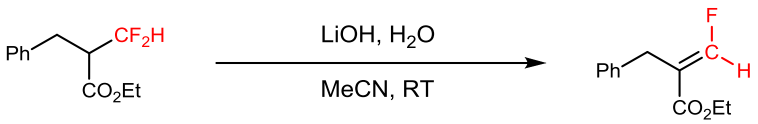

4 6

To a mixture of H_2O (72 mg, 4.0 mmol) and LiOH (38.3 mg, 1.6 mmol) in 4 mL of MeCN was added **4** (91 mg, 0.4 mmol) under a nitrogen atmosphere. After stirring at 25 °C for 2 h, the reaction mixture was quenched with water, extracted with EtOAc, washed with brine, dried over anhydrous Na_2SO_4, and concentrated. Column chromatography on silica gel (PE/EtOAc = 50:1) gave 29 mg (35% yield) of **6**[4] as a yellow oil. ^{1}H NMR (400 MHz, $CDCl_3$) δ 7.63 (d, J = 81.9 Hz, 1H), 7.27–7.16 (m, 5H), 4.17 (q, J = 7.1 Hz, 2H), 3.65 (d, J = 2.9 Hz, 2H), 1.24 (t, J = 7.1 Hz, 3H); ^{13}C NMR (151 MHz, $CDCl_3$) δ 166.2 (d, J = 17.9 Hz), 158.2 (d, J = 277.0 Hz), 138.71 (d, J = 2.5 Hz), 128.6, 128.4, 126.3, 118.1 (d, J = 9.8 Hz), 60.8, 29.2 (d, J = 2.8 Hz), 14.1; ^{19}F NMR (377 MHz, $CDCl_3$) δ -116.2.

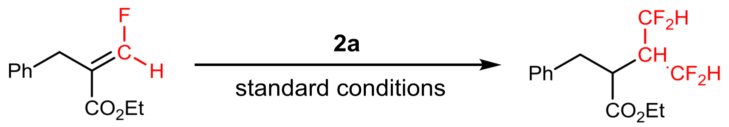

6 3

To a mixture of CF_2HSO_2Na (56 mg, 0.4 mmol), 4DPAIPN (8.0 mg, 0.01 mmol), H_2O (18.0 mg, 1.0 mmol), and LiOH (9.6 mg, 0.4 mmol) in 1 mL of MeCN was added **6** (20.8 mg, 0.1 mmol) under a nitrogen atmosphere. After 24 h of irradiation at a distance of ~2 cm with 24 W of blue LEDs (PINO® lamps, 100% light intensity) at 25 °C, the reaction mixture was quenched with water,

extracted with EtOAc, washed with brine, dried over anhydrous Na_2SO_4, and concentrated. Column chromatography on silica gel (PE/EtOAc = 50:1) gave 28 mg (96% yield) of **3** as a yellow oil.

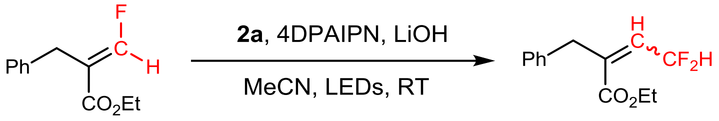

6 **7**

To a mixture of CF_2HSO_2Na (56 mg, 0.4 mmol), 4DPAIPN (16.0 mg, 0.02 mmol), and LiOH (9.6 mg, 0.4 mmol) in 4 mL of MeCN was added **6** (83 mg, 0.4 mmol) under a nitrogen atmosphere. After 1 h of irradiation at a distance of ~2 cm with 24 W of blue LEDs (PINO® lamps, 100% light intensity) at 25 °C, the reaction mixture was quenched with water, extracted with EtOAc, washed with brine, dried over anhydrous Na_2SO_4, and concentrated. Column chromatography on silica gel (PE/EtOAc = 50:1) gave 28 mg (29% yield) of **7** as a yellow oil. Z/E = 1.2:1, ^{1}H NMR (600 MHz, $CDCl_3$) δ 7.33–7.25 (m, 2H), 7.23–7.17 (m, 3H), 6.88 (td, J = 54.0, 6.3 Hz, 0.53H), 6.79–6.77 (m, 0.49 H), 6.50 (td, J = 55.0, 6.5 Hz, 0.62H), 5.87–5.83 (m, 0.53H), 4.23–4.16 (m, 2H), 3.78 (s, 1H), 3.67 (s, 1H), 1.25 (q, J = 7.3 Hz, 3H); ^{13}C NMR (151 MHz, $CDCl_3$) δ 165.9, 140.2, 136.7, 131.9 (t, J = 28.1 Hz), 129.1, 128.7, 126.9, 111.8 (t, J = 233.9 Hz), 61.5, 39.6, 32.9, 13.9; ^{19}F NMR (565 MHz, $CDCl_3$) δ -111.4, -112.1; HRMS (ESI) m/z: $[M + H]^+$ Calcd for $C_{13}H_{14}F_2O_2$+H^+: 241.1035; Found 241.1026.

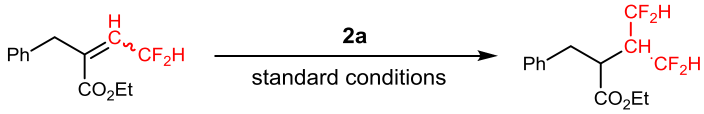

7 **3**

To a mixture of CF_2HSO_2Na (27.8 mg, 0.2 mmol), 4DPAIPN (8.0 mg, 0.01 mmol), H_2O (18.0 mg, 1.0 mmol), and LiOH (4.8 mg, 0.2 mmol) in 1 mL of MeCN was added **7** (24.0 mg, 0.1 mmol) under a nitrogen atmosphere. After 24 h of irradiation at a distance of ~2 cm with 24 W of blue LEDs (PINO® lamps, 100% light intensity) at 25 °C, the reaction mixture was quenched with water, extracted with EtOAc, washed with brine, dried over anhydrous Na_2SO_4, and concentrated. Column chromatography on silica gel (PE/EtOAc = 50:1) gave 27 mg (92% yield) of **3** as a yellow oil.

6. Evaluation of PPARα transactivation activities[5]

The transactivation activities on PPARα of compounds **65** and **66** were assessed using the Stop & Glo reagent, according to the manufacturer's instructions. HEK293 cells, purchased from American Type Culture Collection (ATCC) with a catalog number of CRL-1573, were authenticated by Short Tandem Repeat test, then seeded into 96-well plates at a density of 8×10^4 cells/well in 90 μL of cell seeding medium (97% DMEM without phenol red, 2% charcoal stripped FBS and 1% GlutaMax) together with 10 μL transfection reagent (PPARα 1.079 mg/mL and pGL4.35 1.317 mg/mL). Compounds **65** and **66** were prepared 4-fold serial dilution with DMSO starting at 400 μM, 8 points in total, then transferred 500 nL to the compound plate using an Echo liquid handler. 10-Fold dilutions of the compounds with 40 μL culture medium (88% DMEM with phenol red, 10% FBS, 1% P/S and 1% GlutaMax) followed by transferring 10 μL to cell plates, which were placed in an incubator at 37 °C for 24 h. After removing 50 μL medium from each well, 50 μL luciferase assay reagent was added to the assay plate, followed by shaking at 25 °C for 20 min. The data were read on an Envision (Perkin Elemer: Envision 2105), then analyzed using XL-fit software (Supplier: ID Business Solutions Ltd., Software version: XL fit 5.0). Effect% = (Sample value - LC)/(HC - LC) * 100.

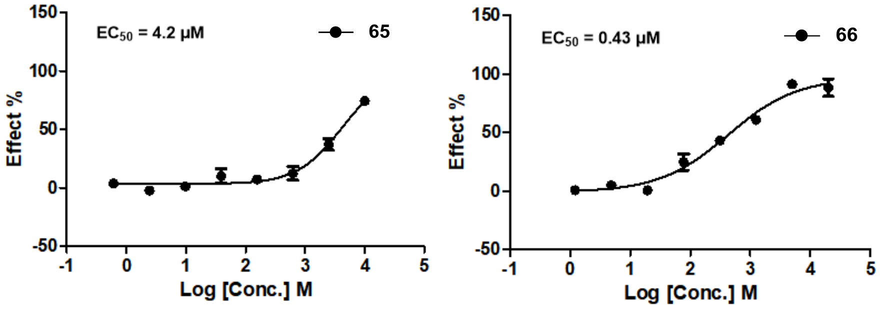

Supplementary Figure 6 The bioactivity evaluation of 65 and 66 for PPARα.

7. NMR spectra

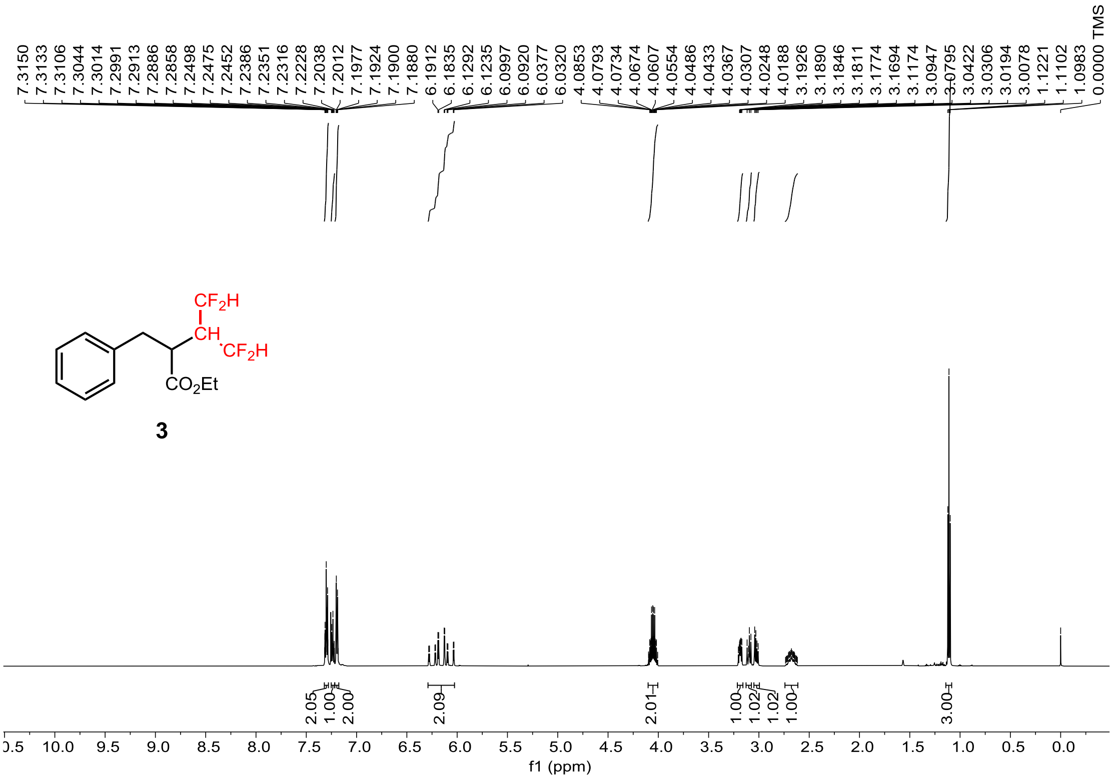

Supplementary Figure 7 ^{1}H NMR (600 MHz, $CDCl_3$) spectrum of compound 3.

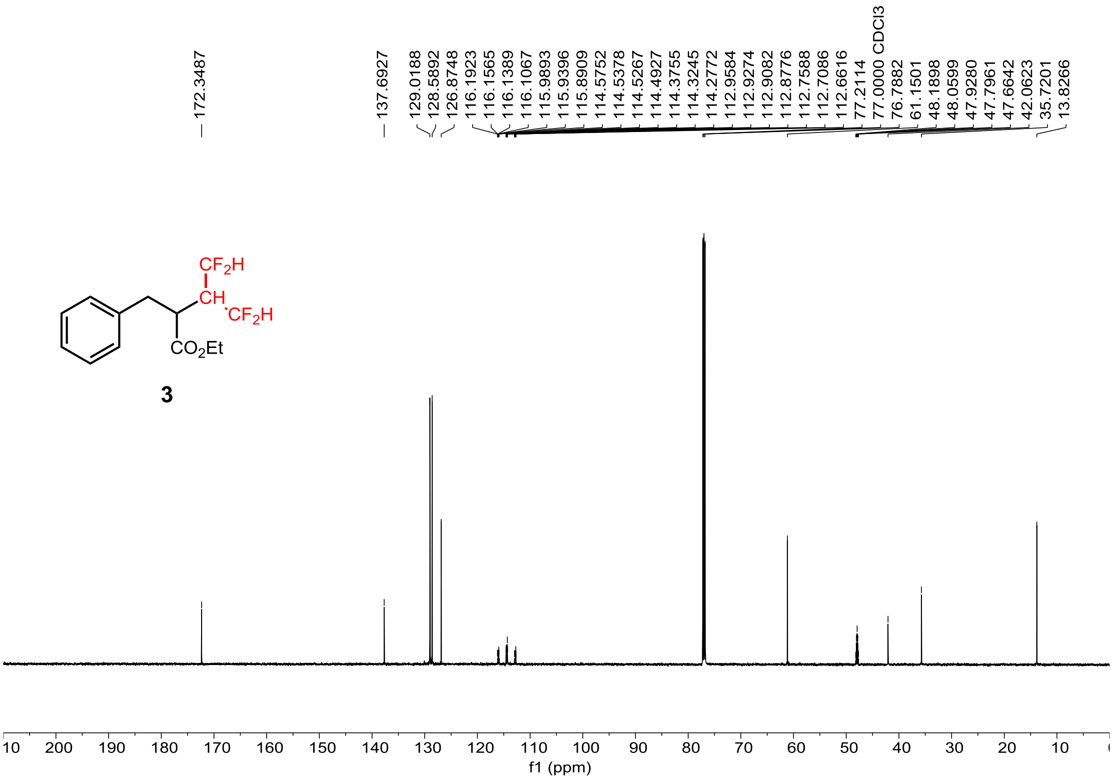

Supplementary Figure 8 ^{13}C NMR (151 MHz, $CDCl_3$) spectrum of compound 3.

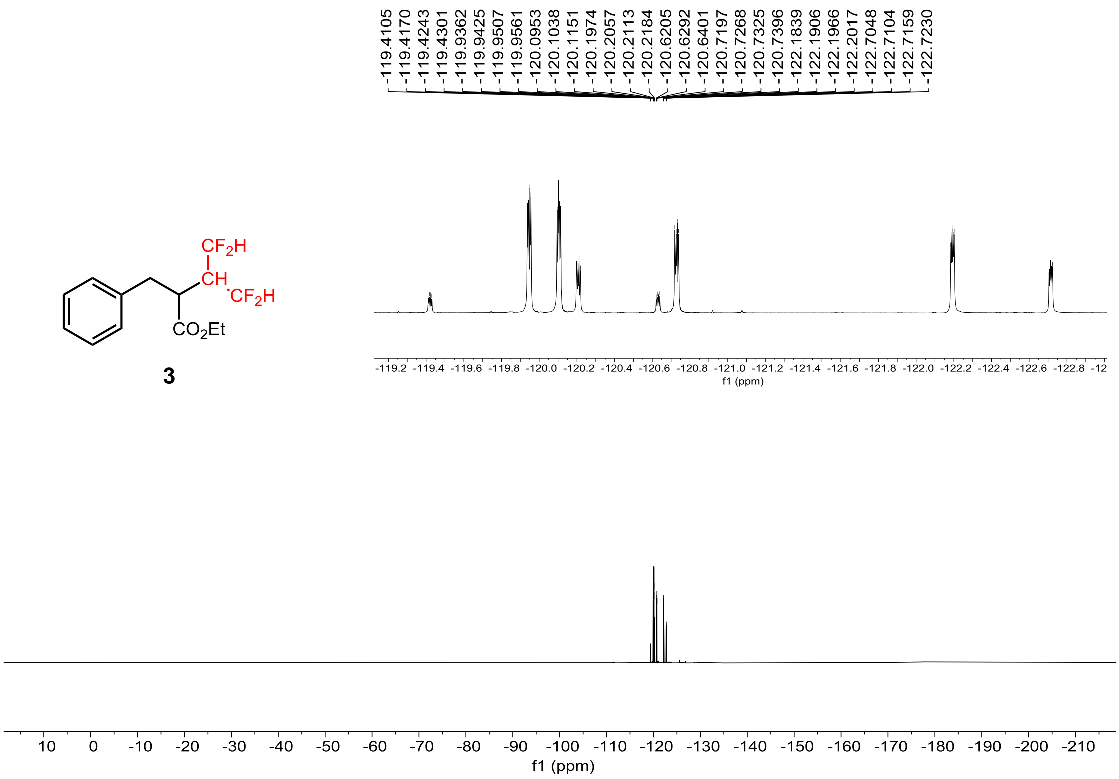

Supplementary Figure 9 ^{19}F NMR (565 MHz, $CDCl_3$) spectrum of compound 3.

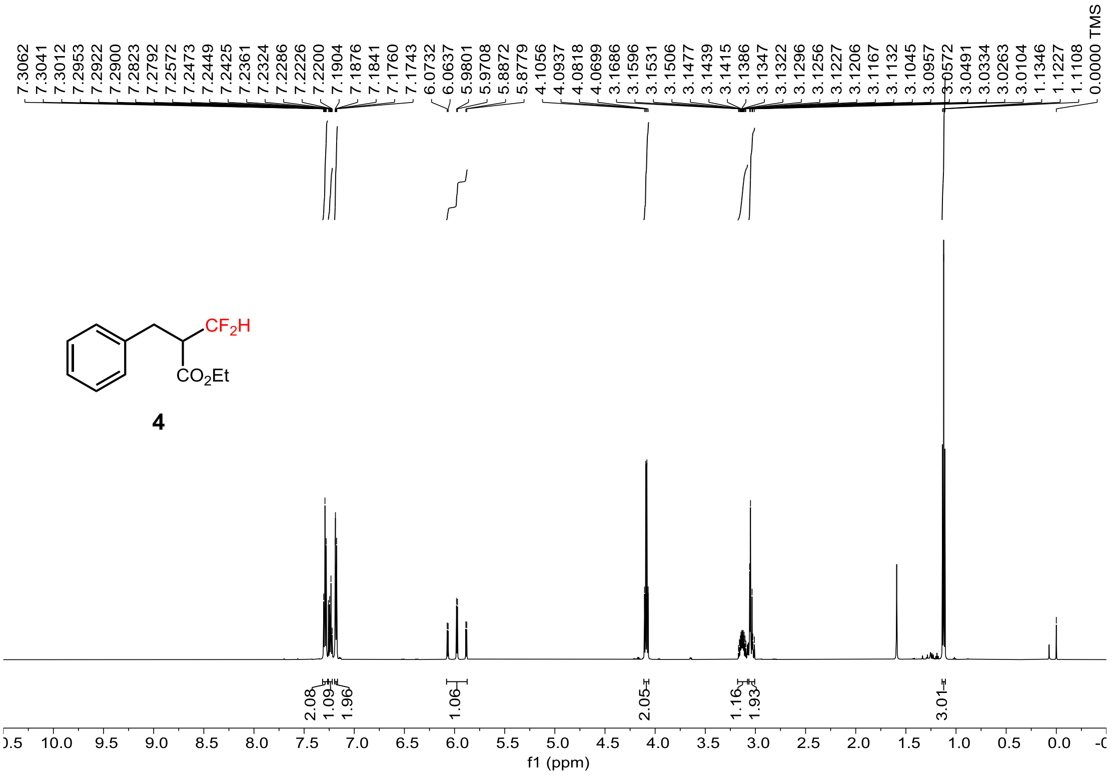

Supplementary Figure 10 ^{1}H NMR (600 MHz, $CDCl_3$) spectrum of compound 4.

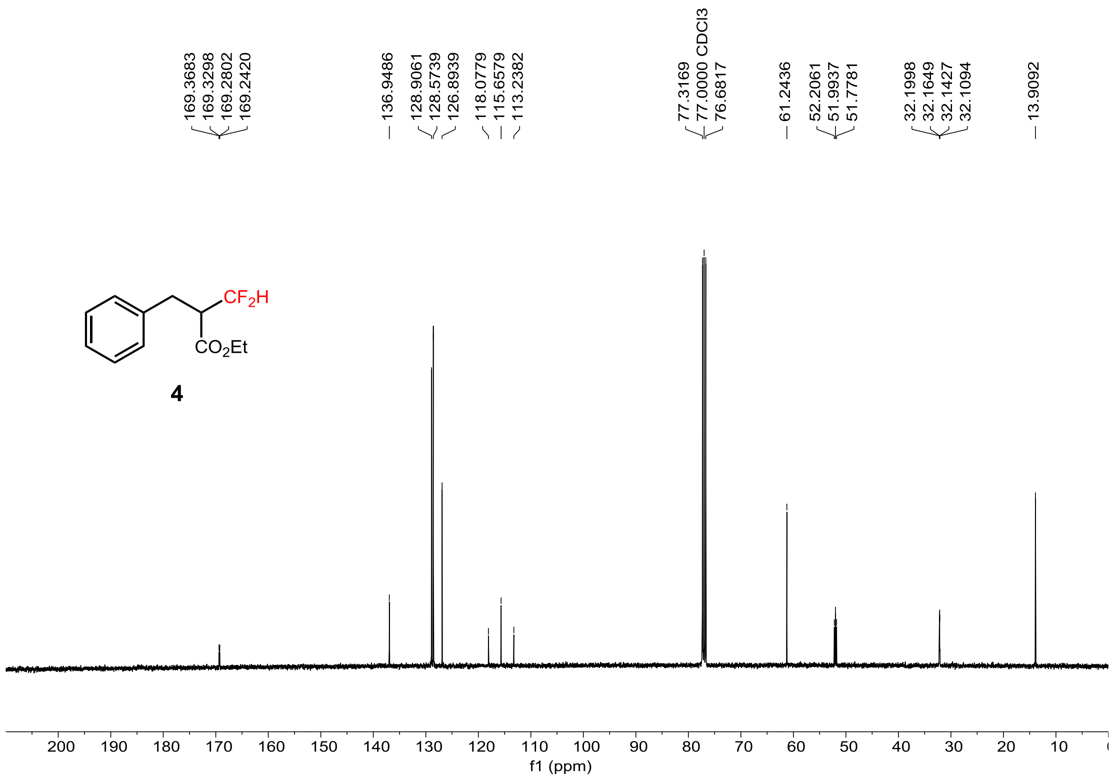

Supplementary Figure 11 ^{13}C NMR (101 MHz, $CDCl_3$) spectrum of compound 4.

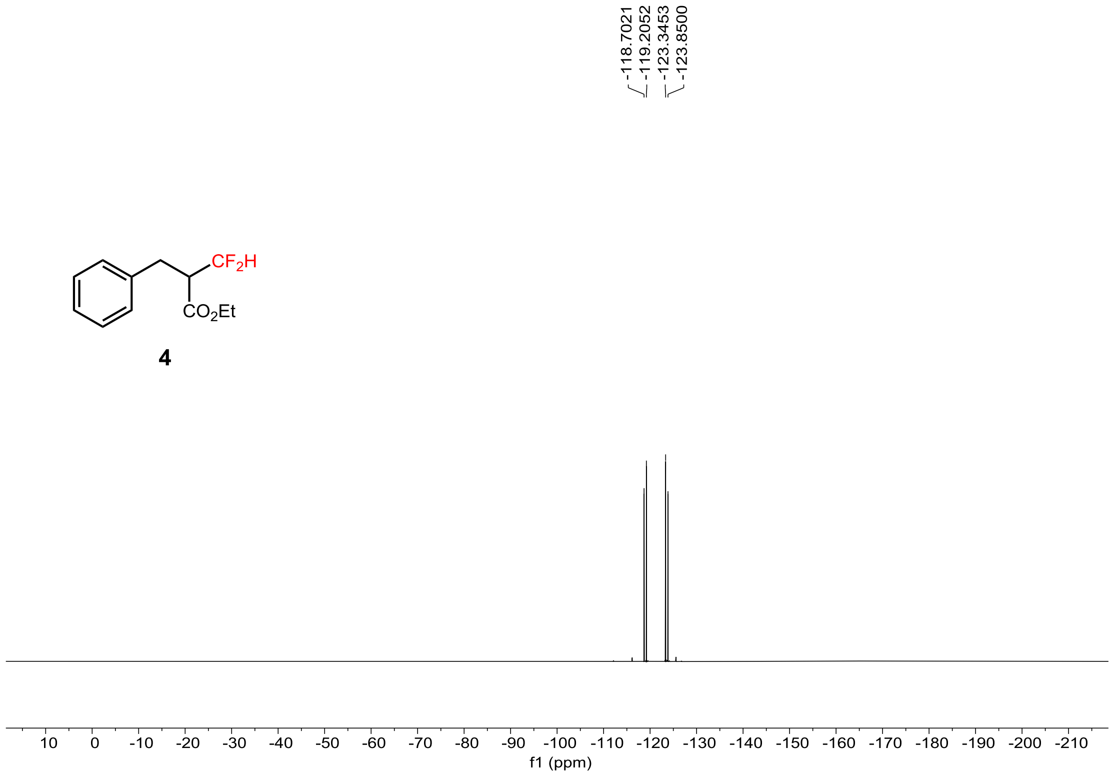

Supplementary Figure 12 ^{19}F NMR (565 MHz, $CDCl_3$) spectrum of compound 4.

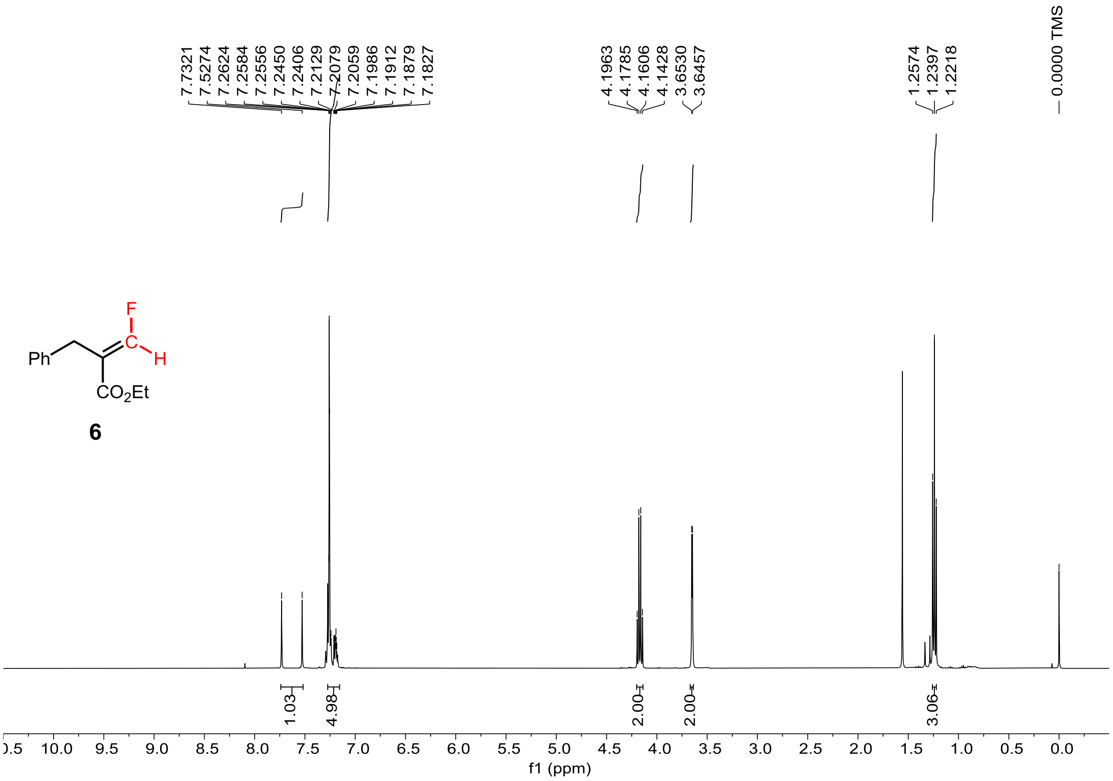

Supplementary Figure 13 ^{1}H NMR (400 MHz, $CDCl_3$) spectrum of compound 6.

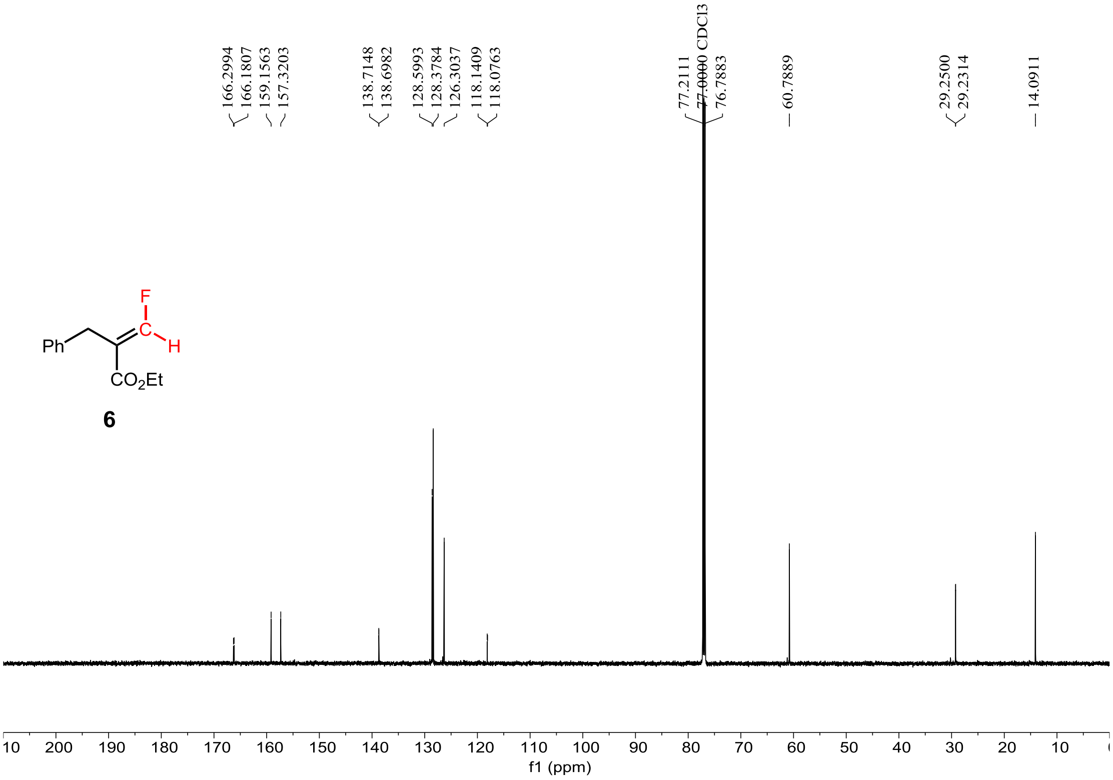

Supplementary Figure 14 ^{13}C NMR (151 MHz, $CDCl_3$) spectrum of compound 6.

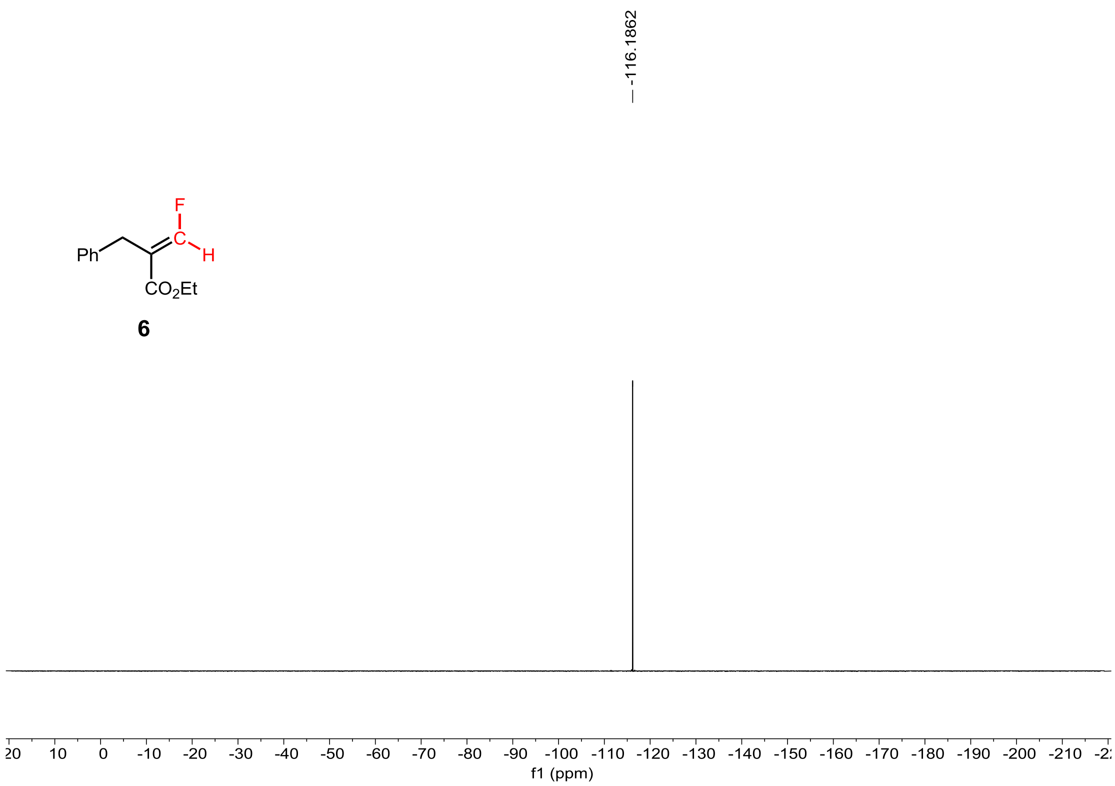

Supplementary Figure 15 ^{19}F NMR (377 MHz, $CDCl_3$) spectrum of compound 6.

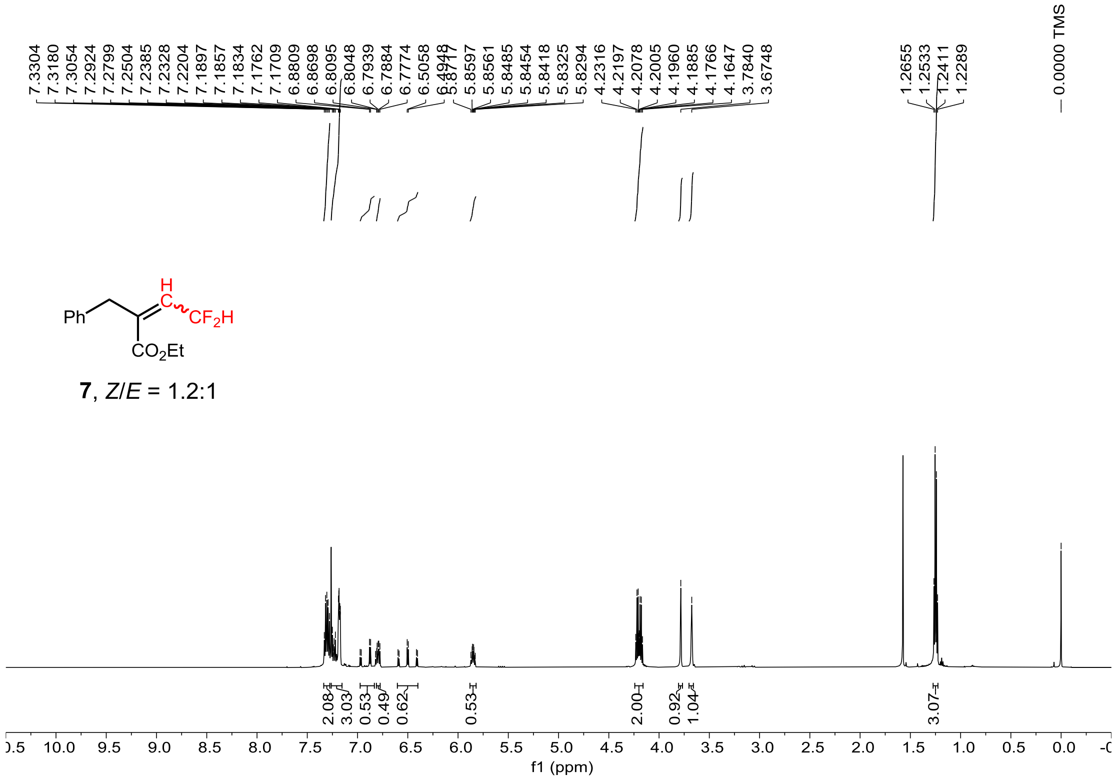

Supplementary Figure 16 ^{1}H NMR (600 MHz, $CDCl_3$) spectrum of compound 7.

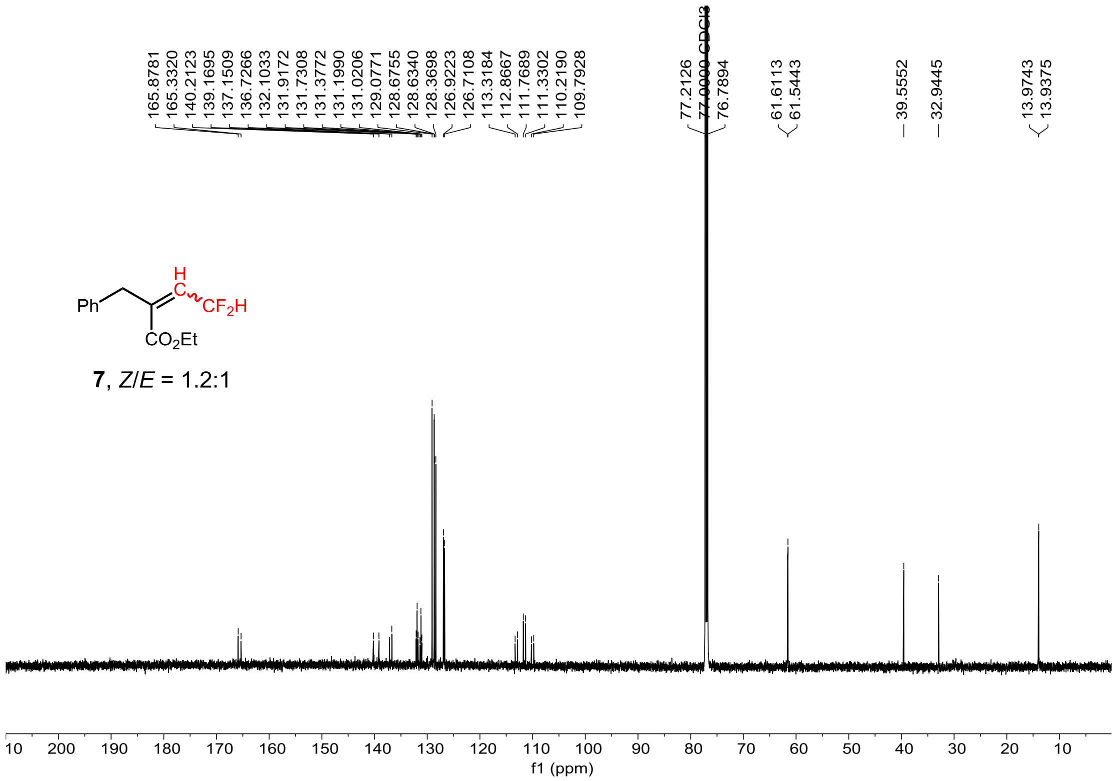

Supplementary Figure 17 ^{13}C NMR (151 MHz, $CDCl_3$) spectrum of compound 7.

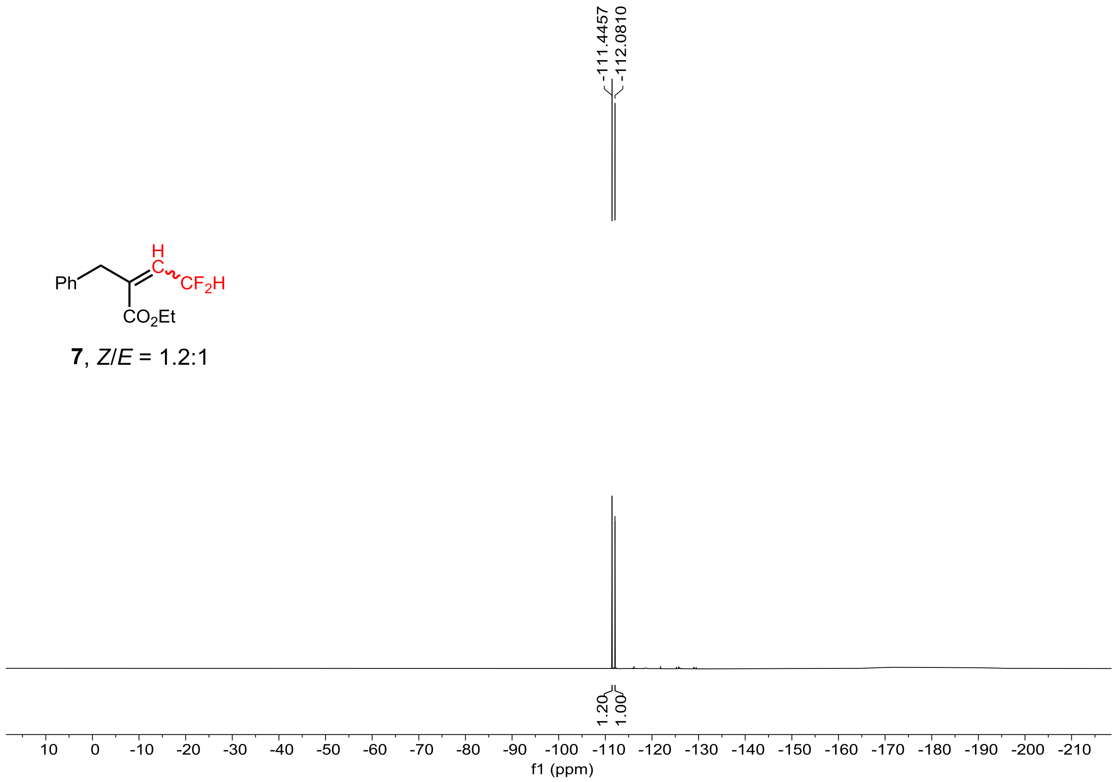

Supplementary Figure 18 ^{19}F NMR (565 MHz, $CDCl_3$) spectrum of compound 7.

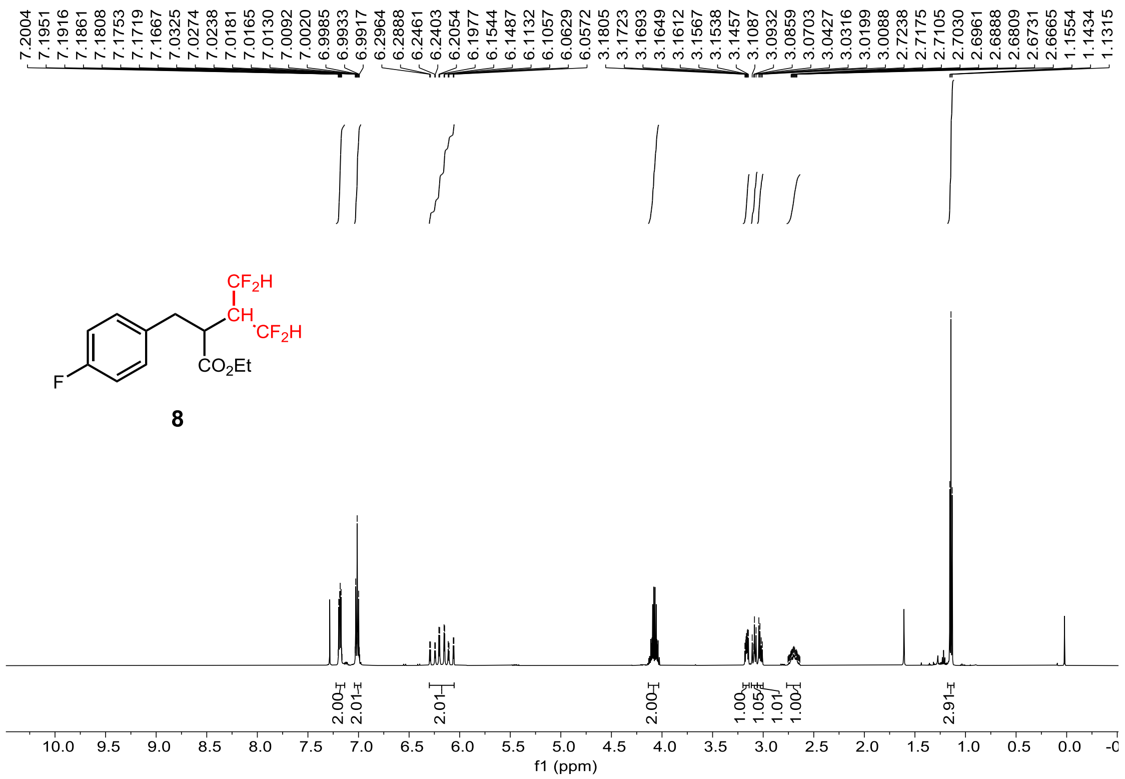

Supplementary Figure 19 ^{1}H NMR (600 MHz, $CDCl_3$) spectrum of compound 8.

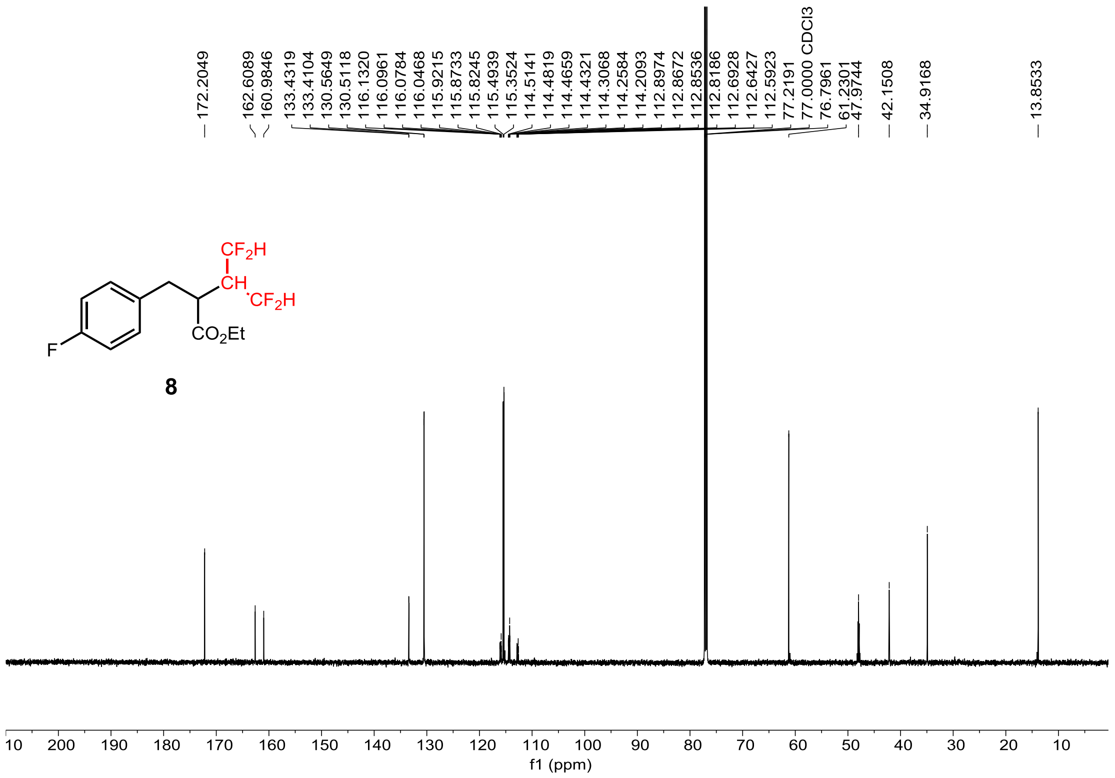

Supplementary Figure 20 ^{13}C NMR (151 MHz, $CDCl_3$) spectrum of compound 8.

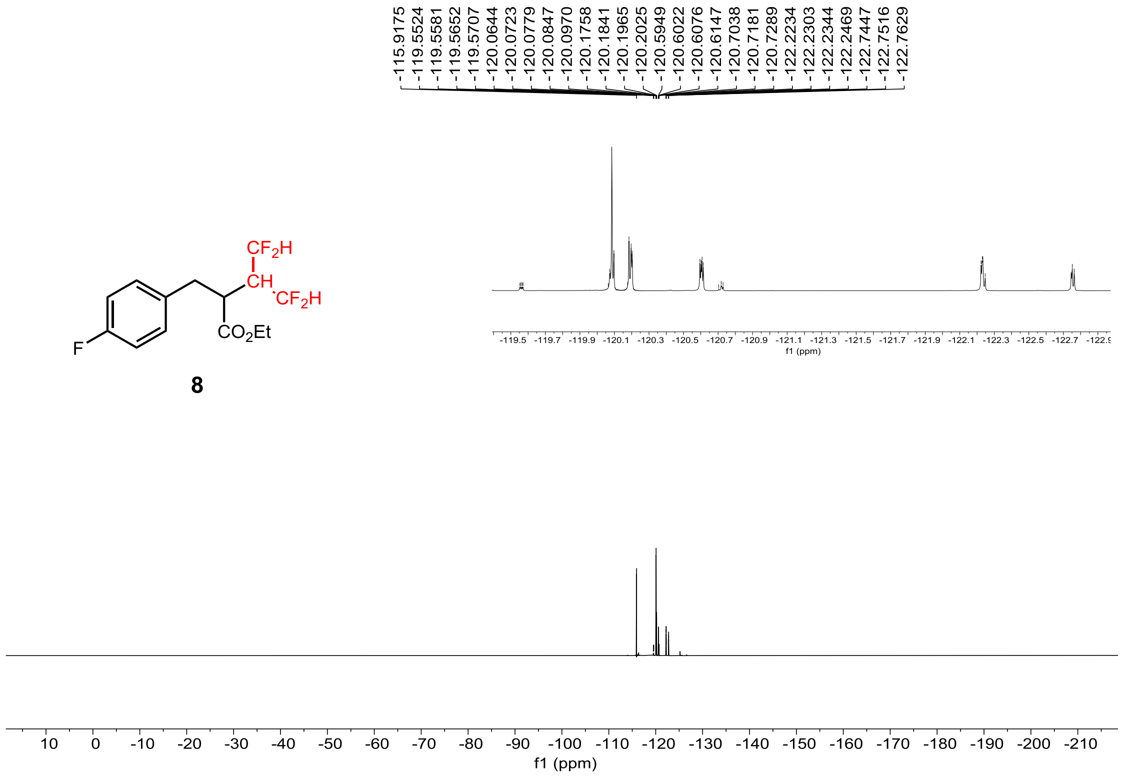

Supplementary Figure 21 ^{19}F NMR (565 MHz, $CDCl_3$) spectrum of compound 8.

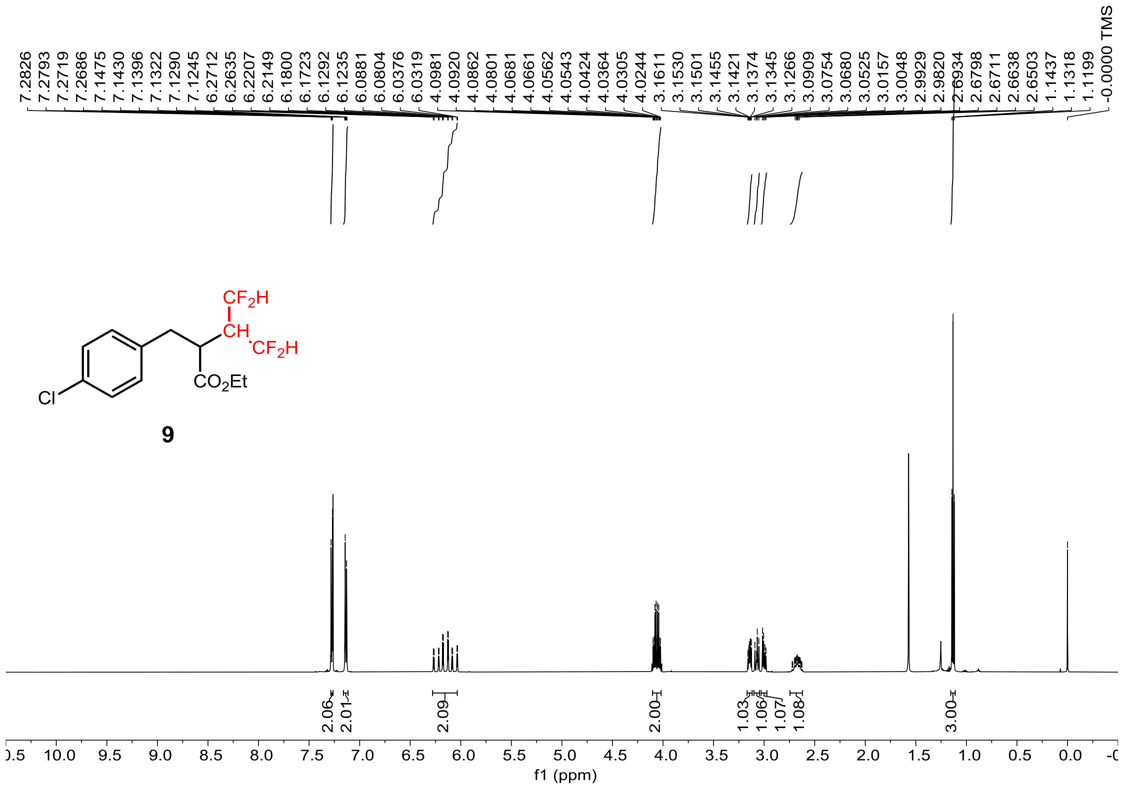

Supplementary Figure 22 ^{1}H NMR (600 MHz, $CDCl_3$) spectrum of compound 9.

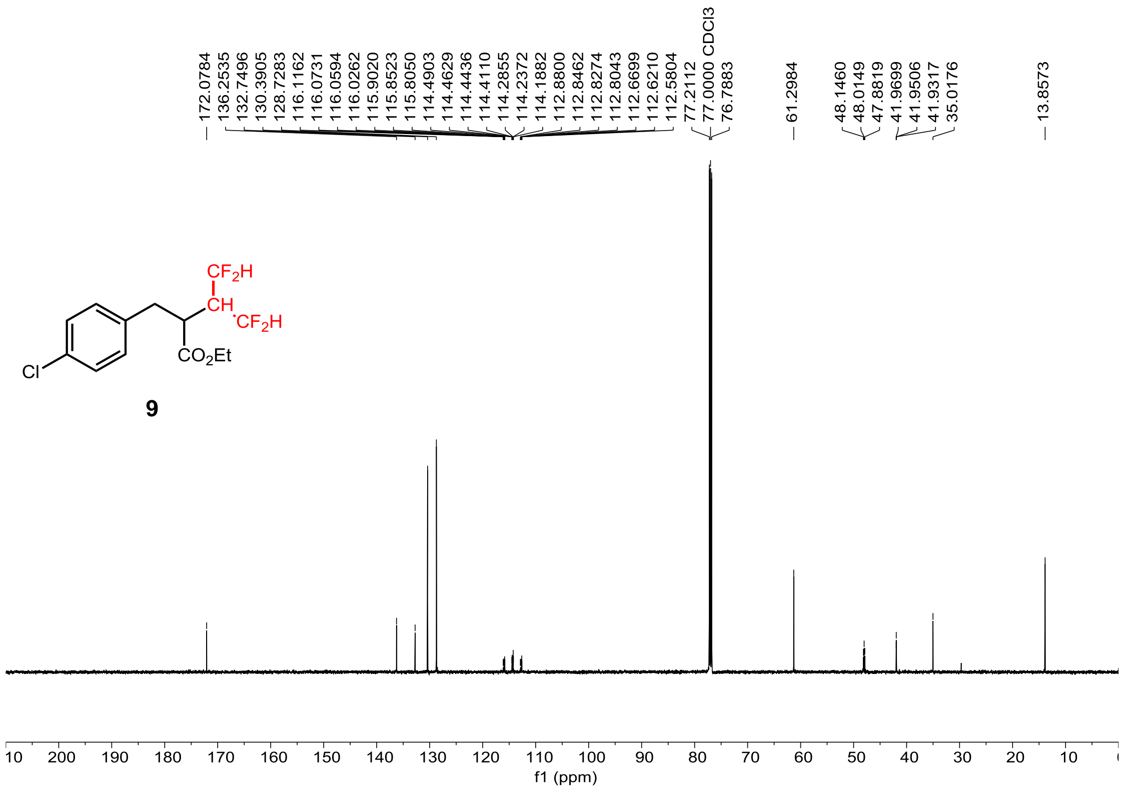

Supplementary Figure 23 ^{13}**C NMR (151 MHz, CDCl**$_3$**) spectrum of compound 9.**

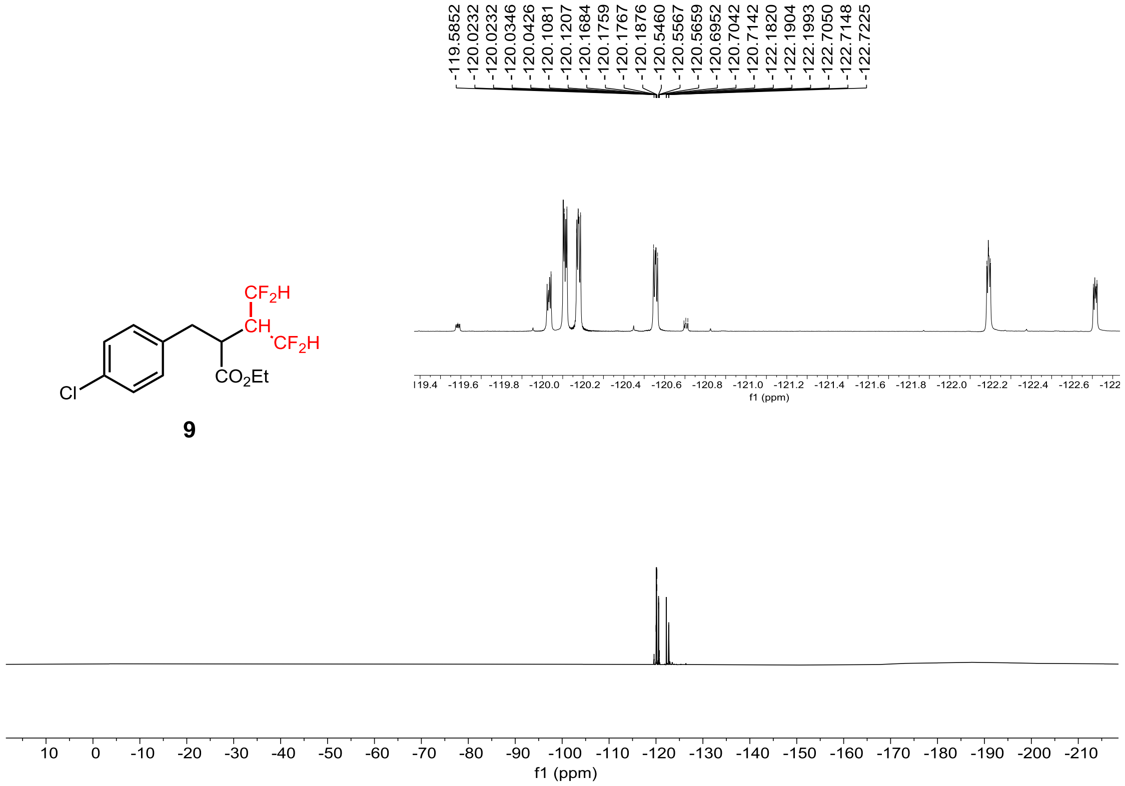

Supplementary Figure 24 ^{19}**F NMR (565 MHz, CDCl**$_3$**) spectrum of compound 9.**

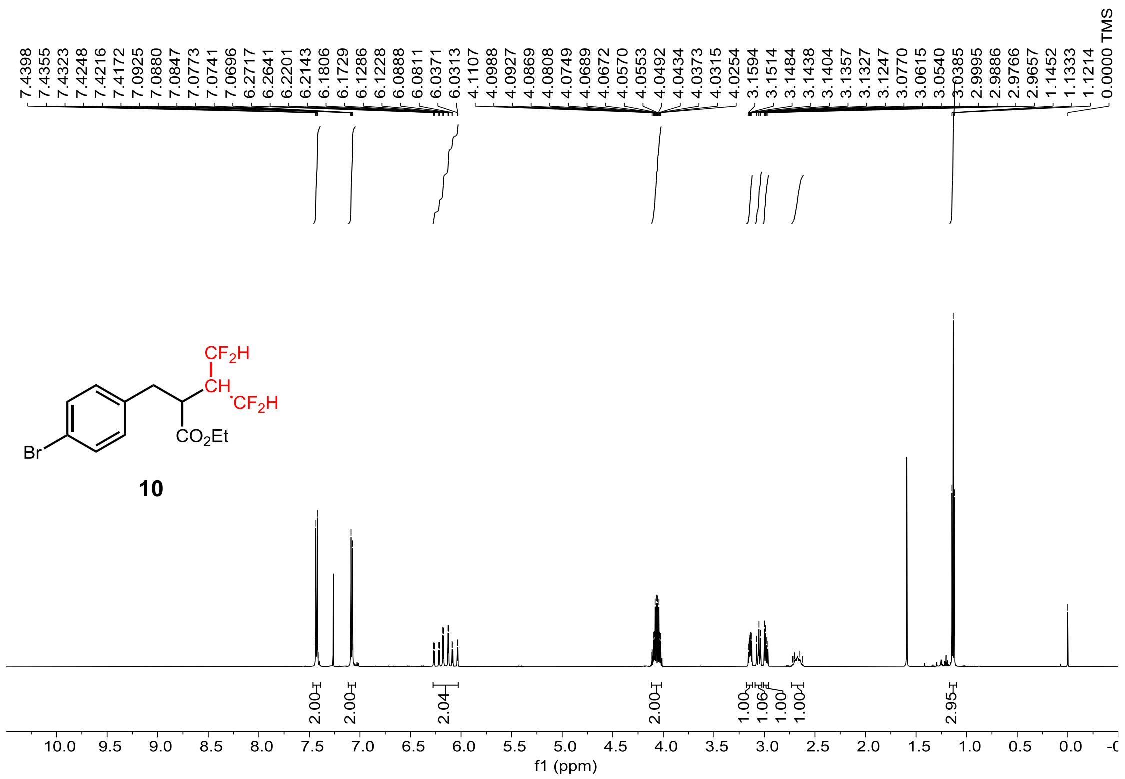

Supplementary Figure 25 ^{1}H NMR (600 MHz, $CDCl_3$) spectrum of compound 10.

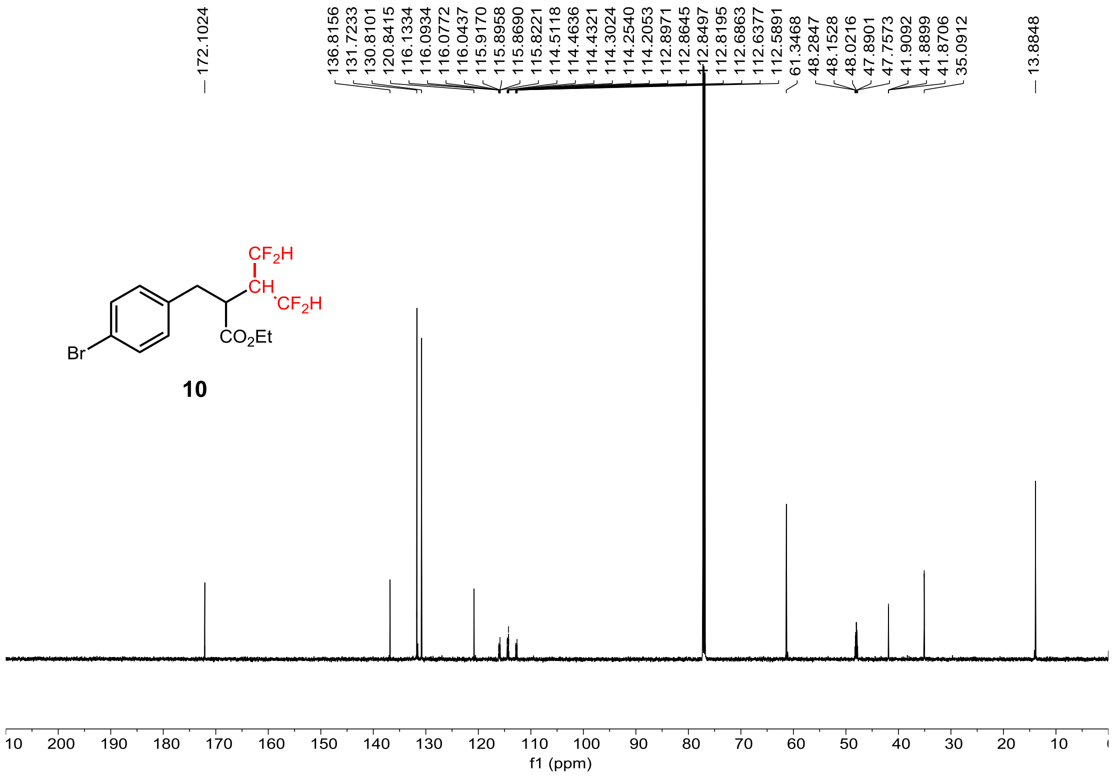

Supplementary Figure 26 ^{13}C NMR (151 MHz, $CDCl_3$) spectrum of compound 10.

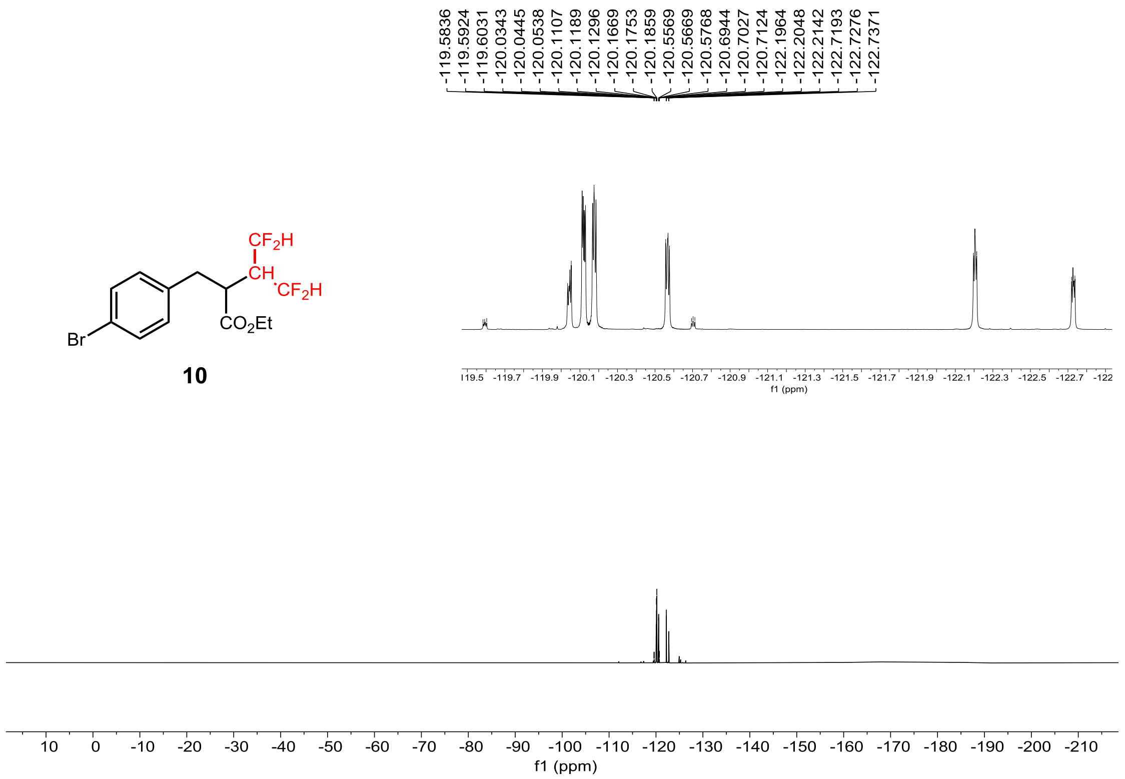

10

Supplementary Figure 27 ^{19}F NMR (565 MHz, $CDCl_3$) spectrum of compound 10.

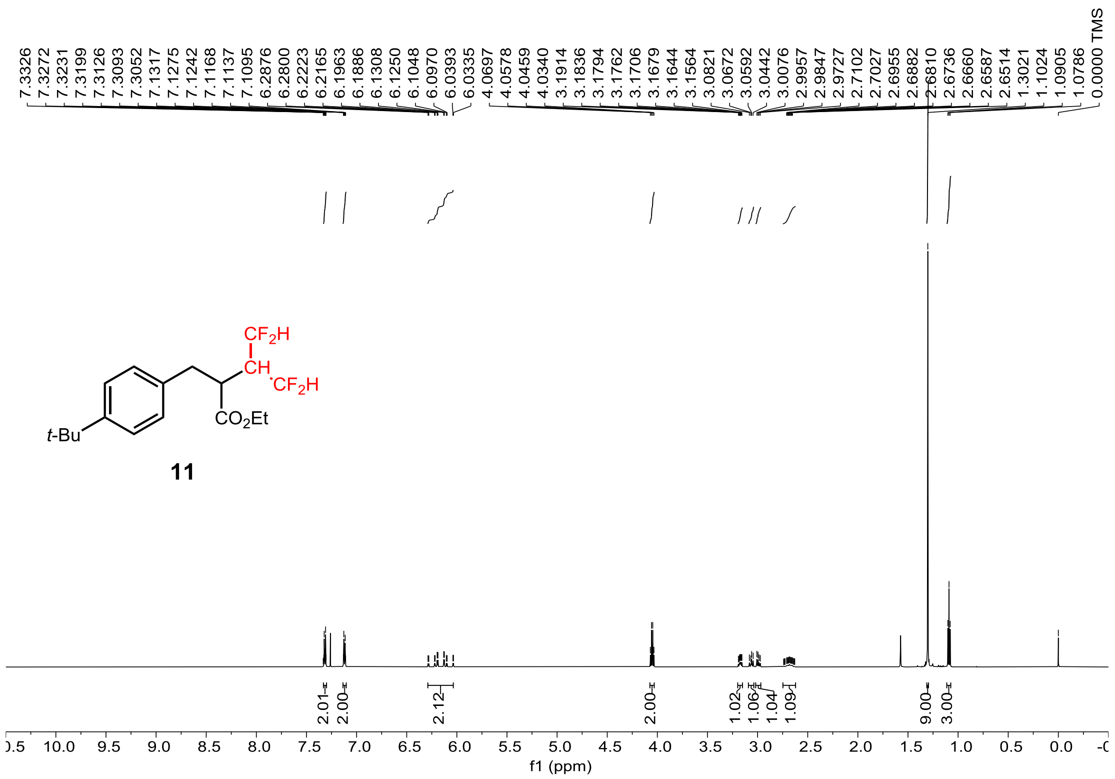

11

Supplementary Figure 28 ^{1}H NMR (600 MHz, $CDCl_3$) spectrum of compound 11.

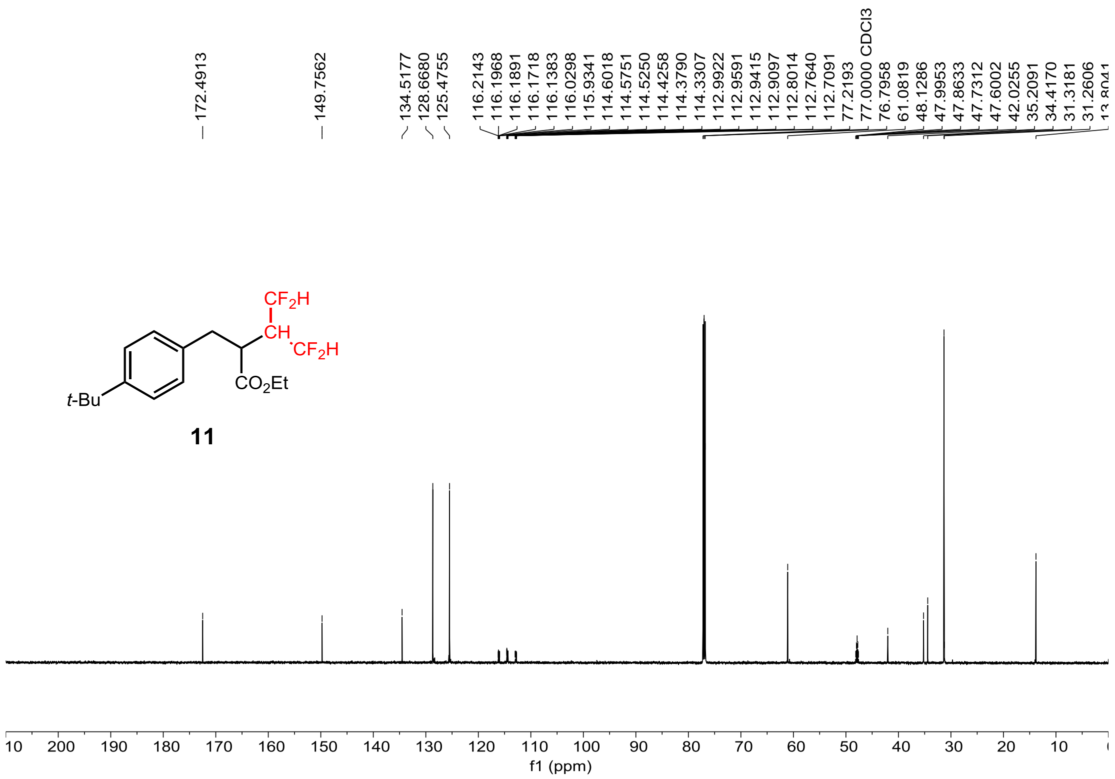

Supplementary Figure 29 ^{13}C NMR (151 MHz, $CDCl_3$) spectrum of compound 11.

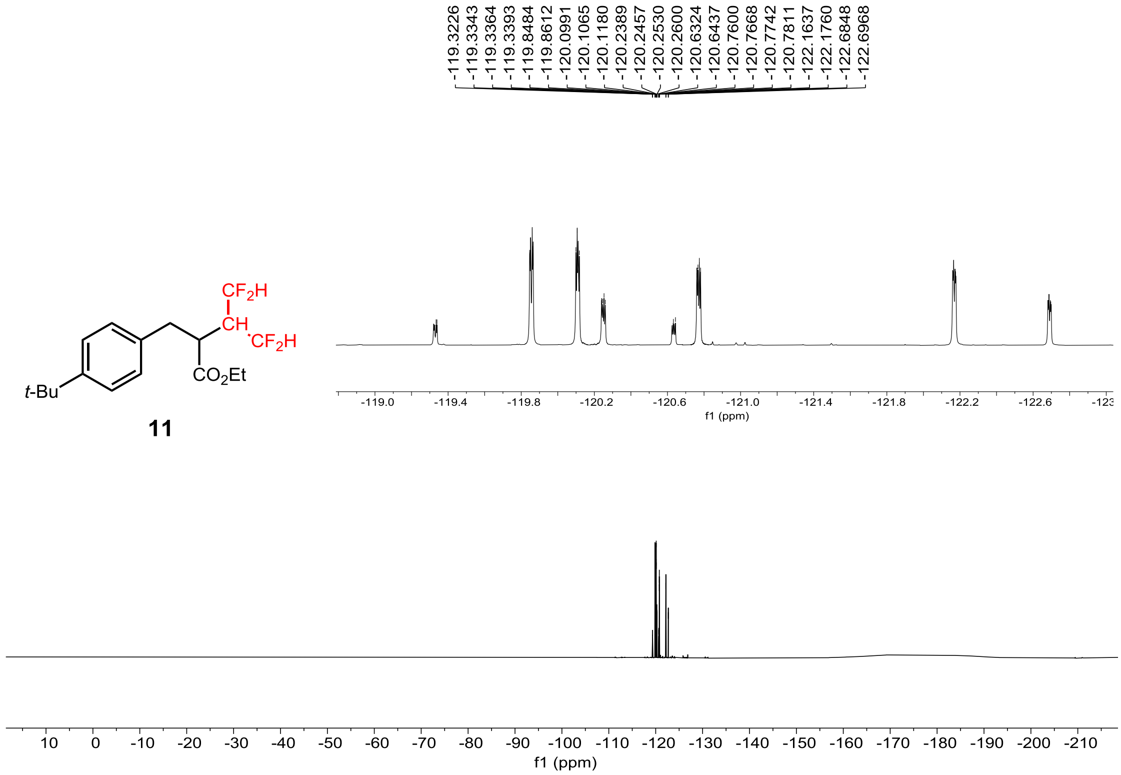

Supplementary Figure 30 ^{19}F NMR (565 MHz, $CDCl_3$) spectrum of compound 11.

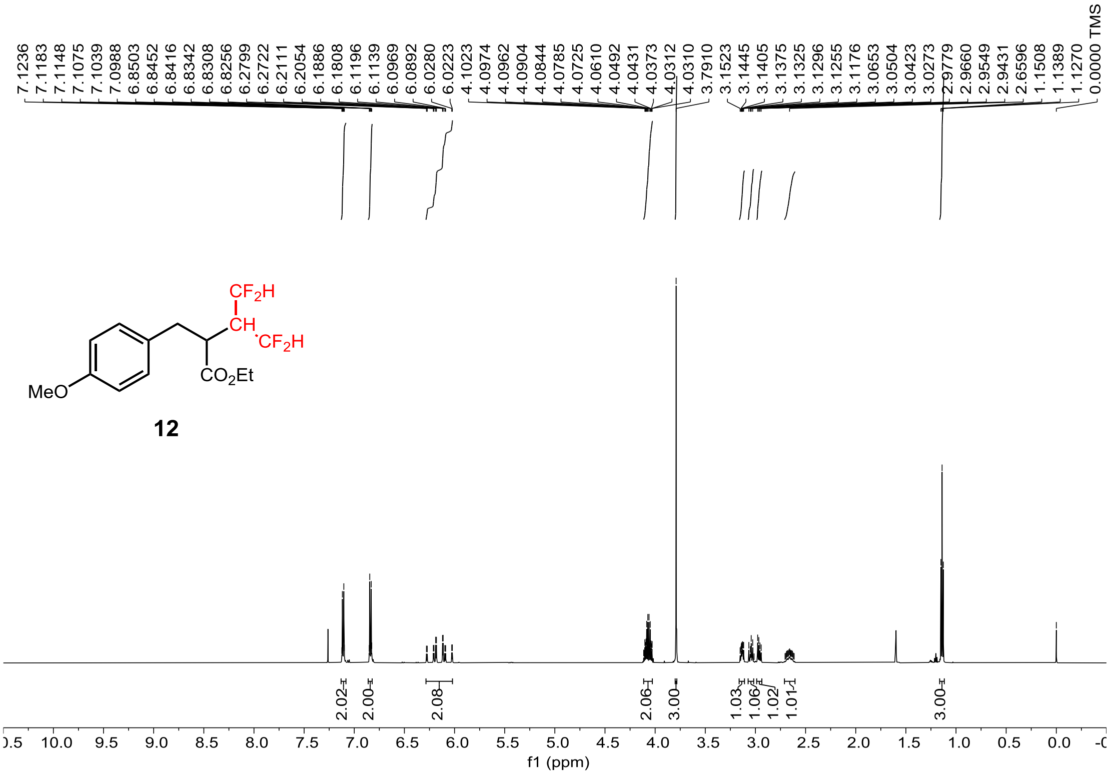

Supplementary Figure 31 ^{1}H NMR (600 MHz, $CDCl_3$) spectrum of compound 12.

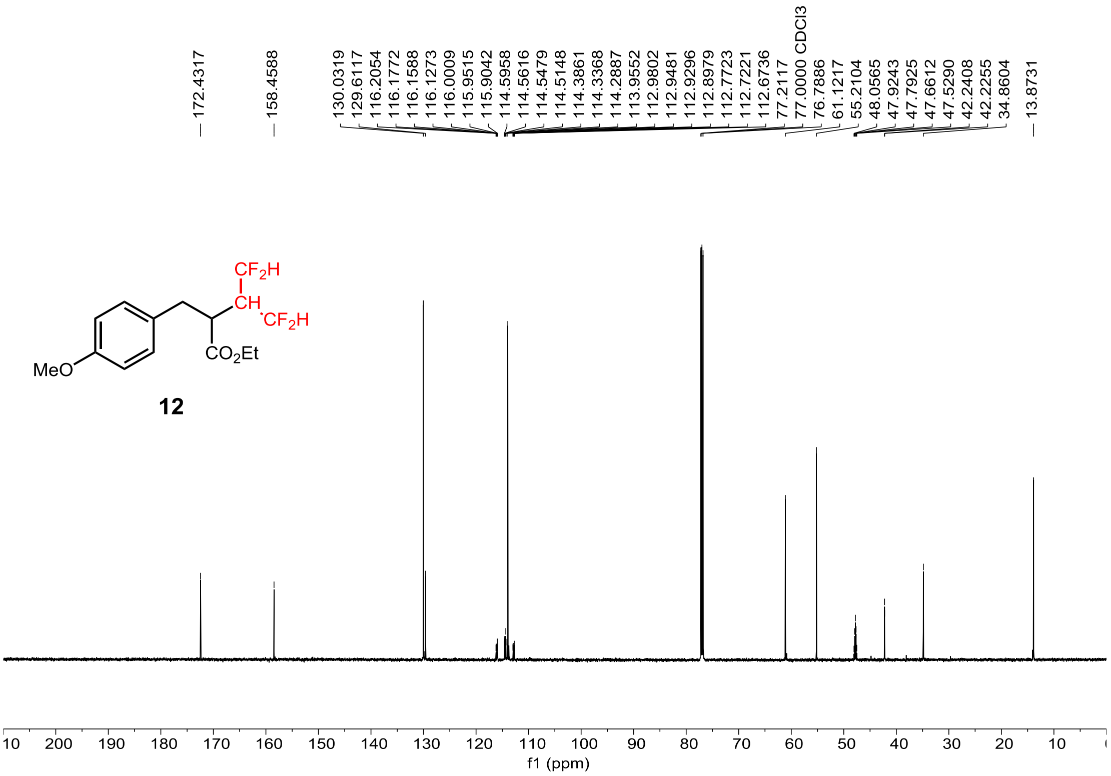

Supplementary Figure 32 ^{13}C NMR (151 MHz, $CDCl_3$) spectrum of compound 12.

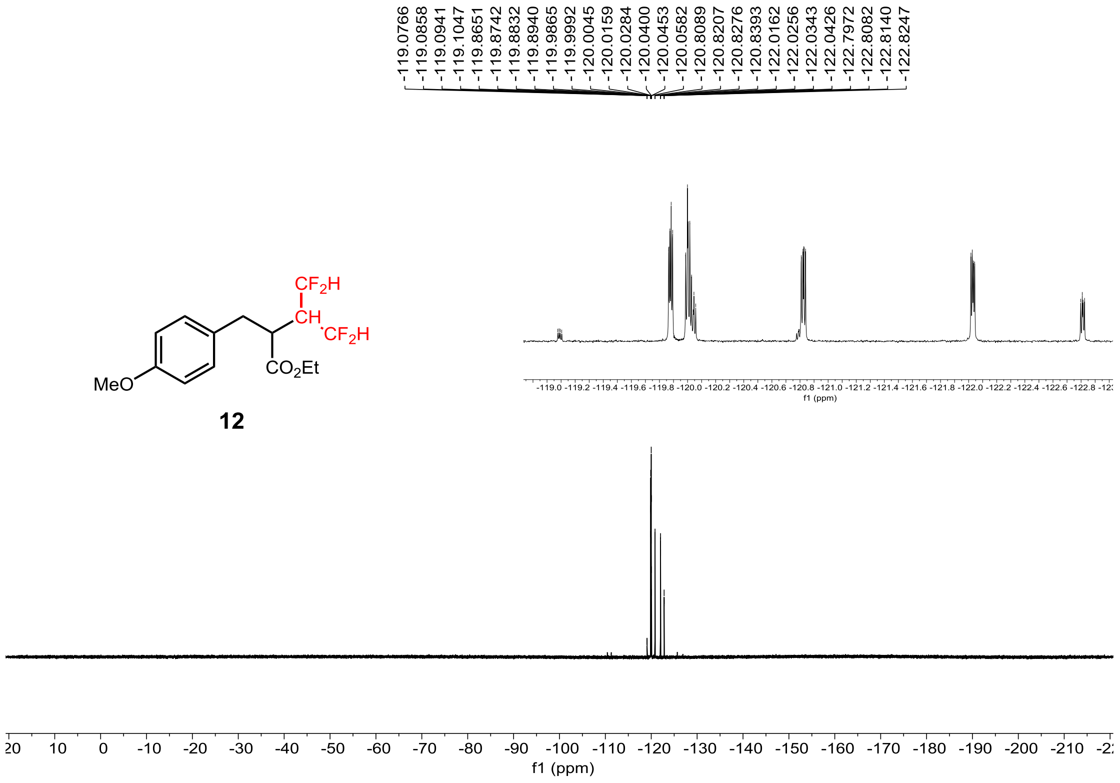

Supplementary Figure 33 ^{19}F NMR (377 MHz, $CDCl_3$) spectrum of compound 12.

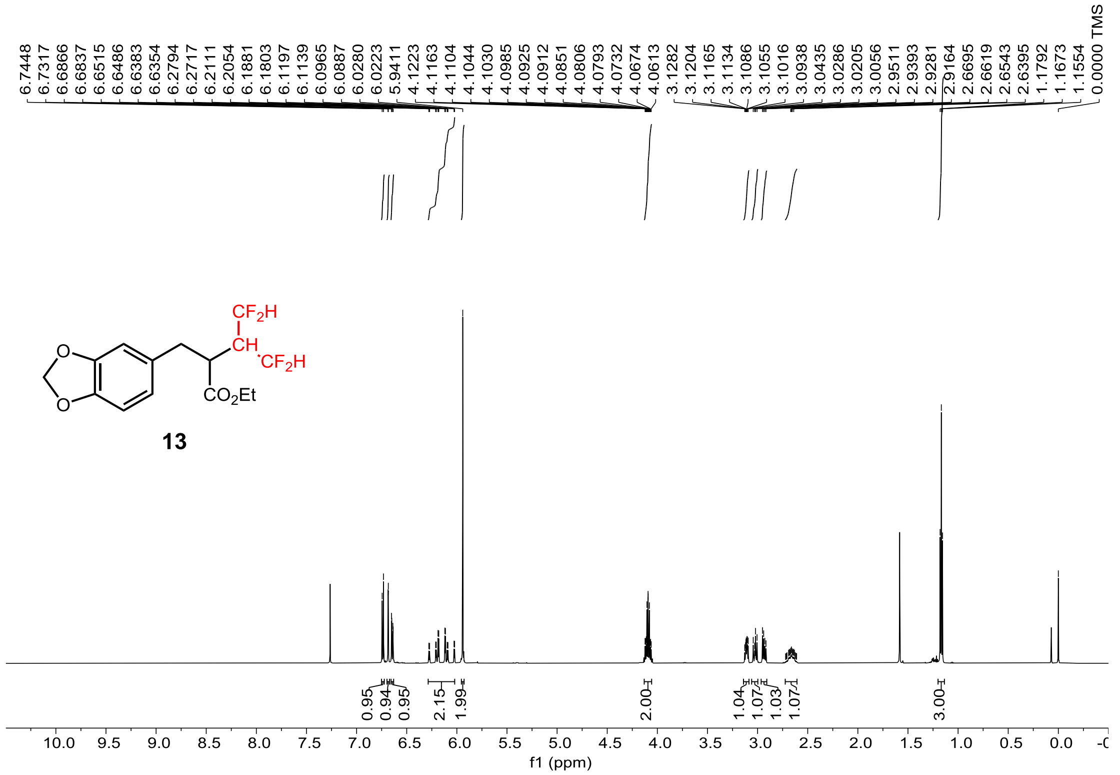

Supplementary Figure 34 ^{1}H NMR (600 MHz, $CDCl_3$) spectrum of compound 13.

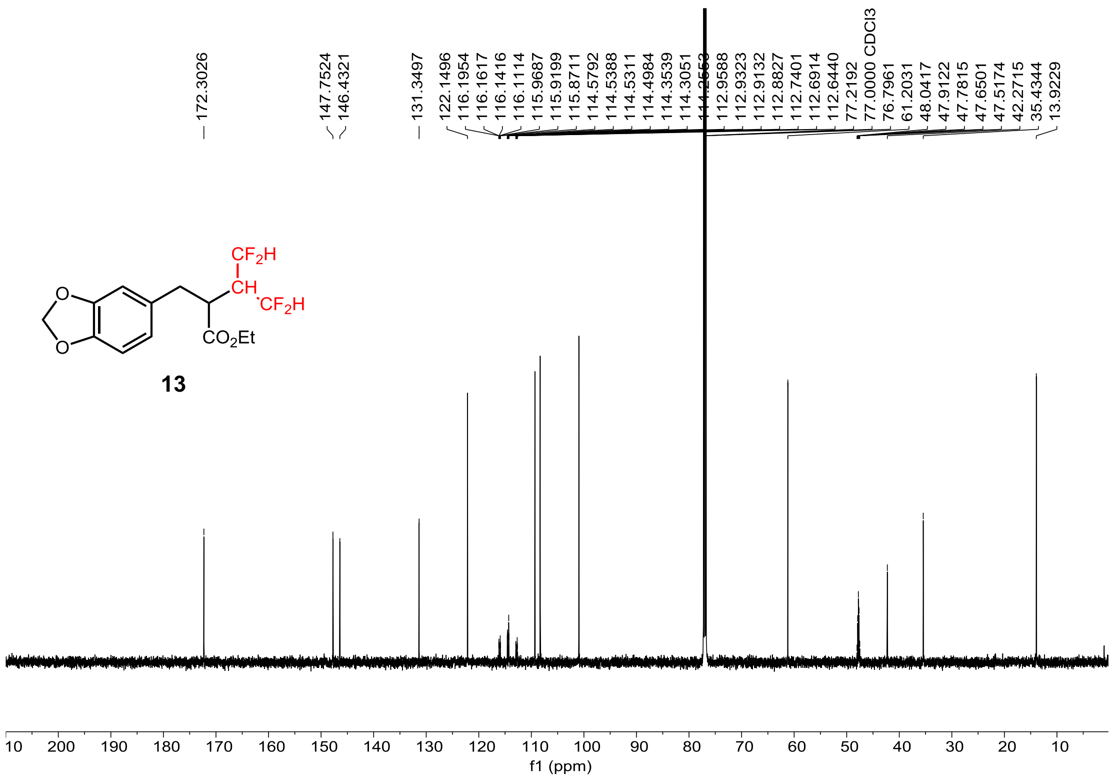

Supplementary Figure 35 ^{13}C NMR (151 MHz, $CDCl_3$) spectrum of compound 13.

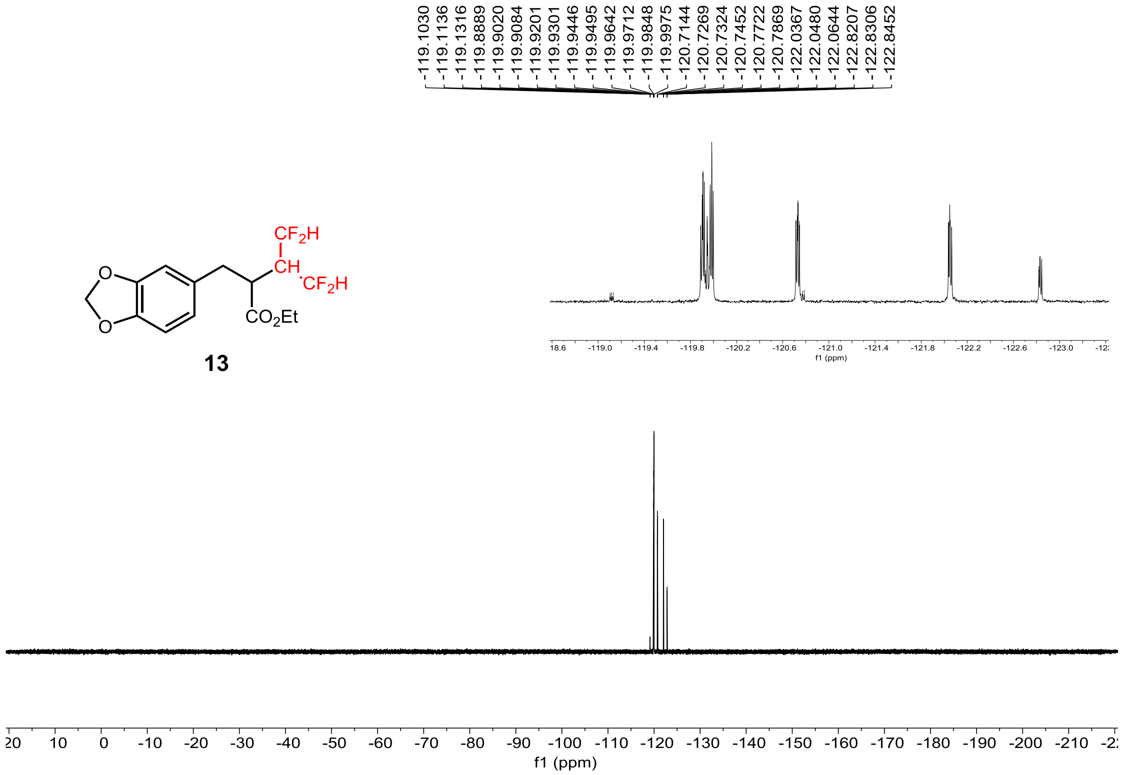

Supplementary Figure 36 ^{19}F NMR (377 MHz, $CDCl_3$) spectrum of compound 13.

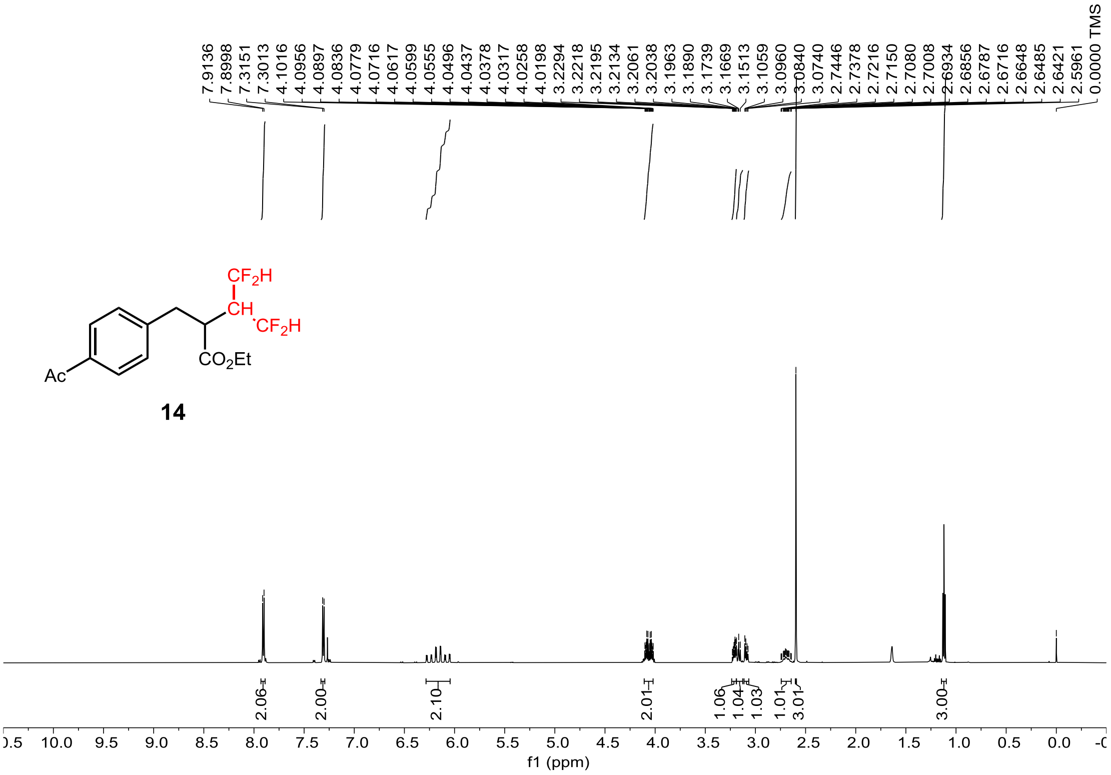

Supplementary Figure 37 ^{1}H NMR (600 MHz, $CDCl_3$) spectrum of compound 14.

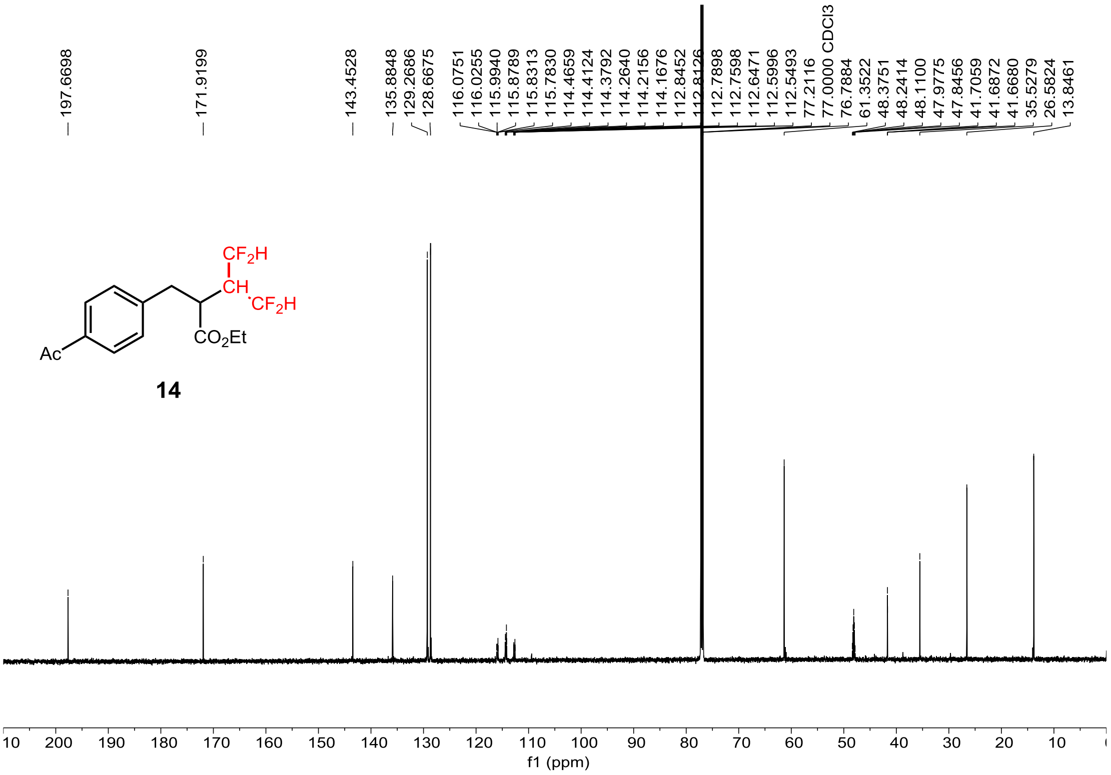

Supplementary Figure 38 ^{13}C NMR (151 MHz, $CDCl_3$) spectrum of compound 14.

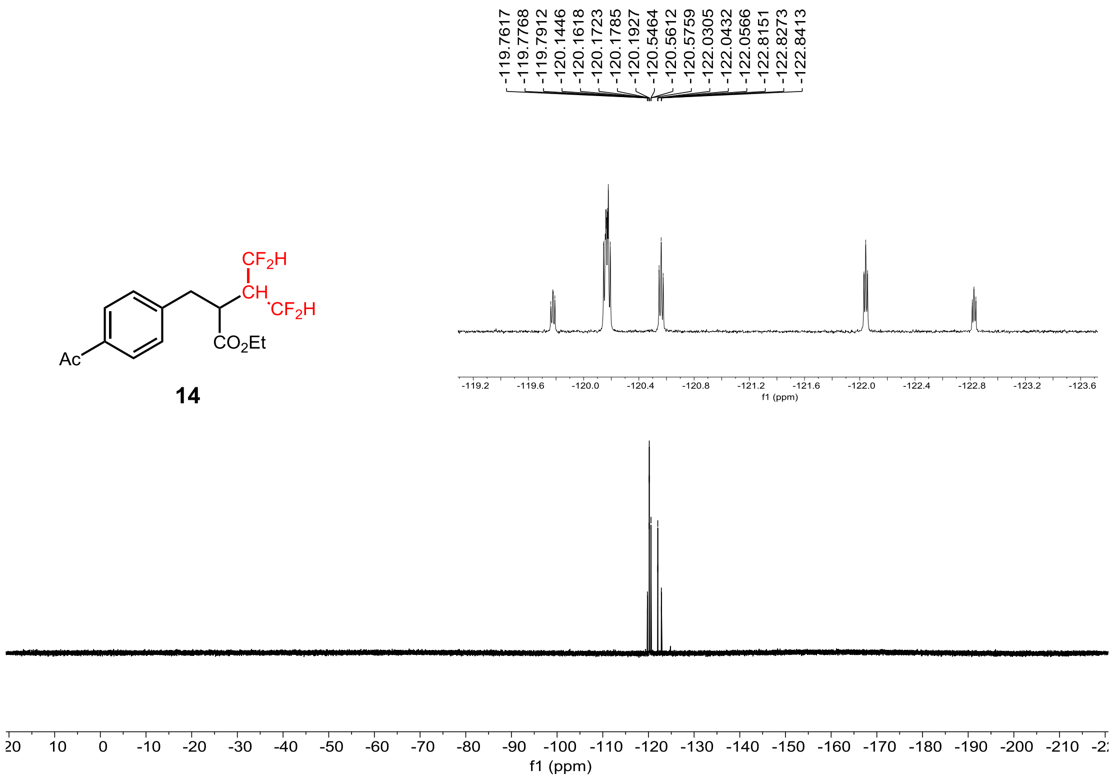

Supplementary Figure 39 ^{19}F NMR (377 MHz, CDCl$_3$) spectrum of compound 14.

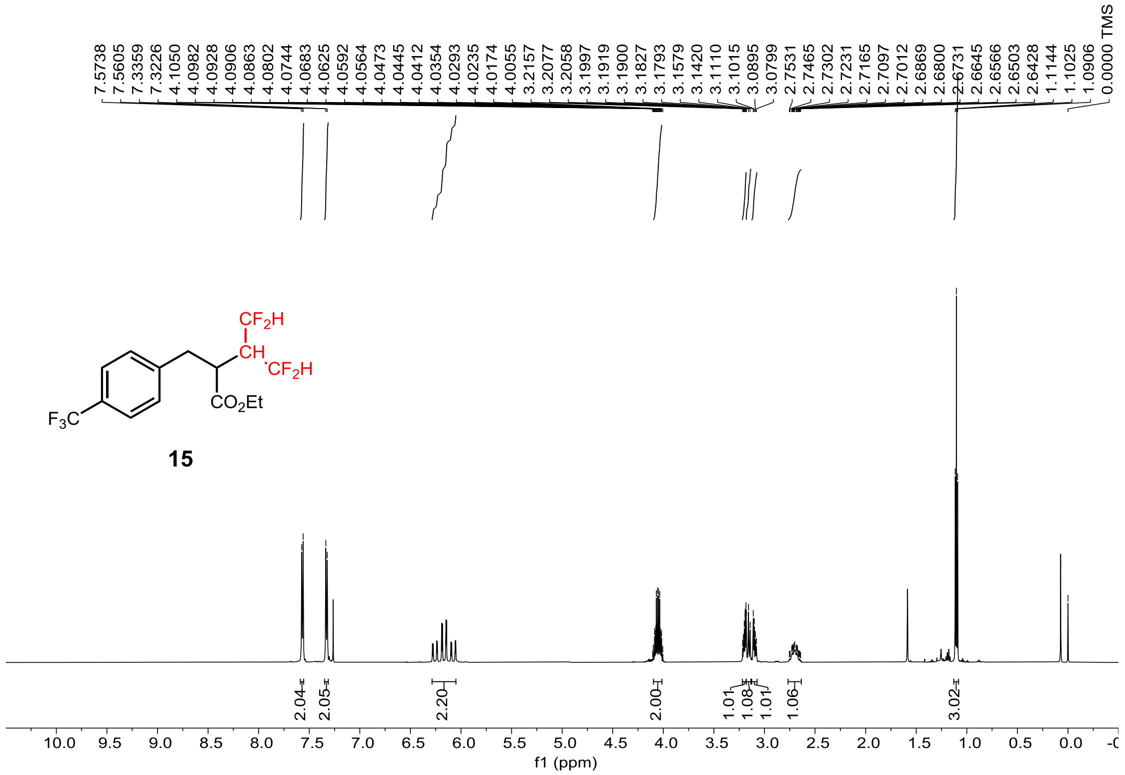

Supplementary Figure 40 ^{1}H NMR (600 MHz, CDCl$_3$) spectrum of compound 15.

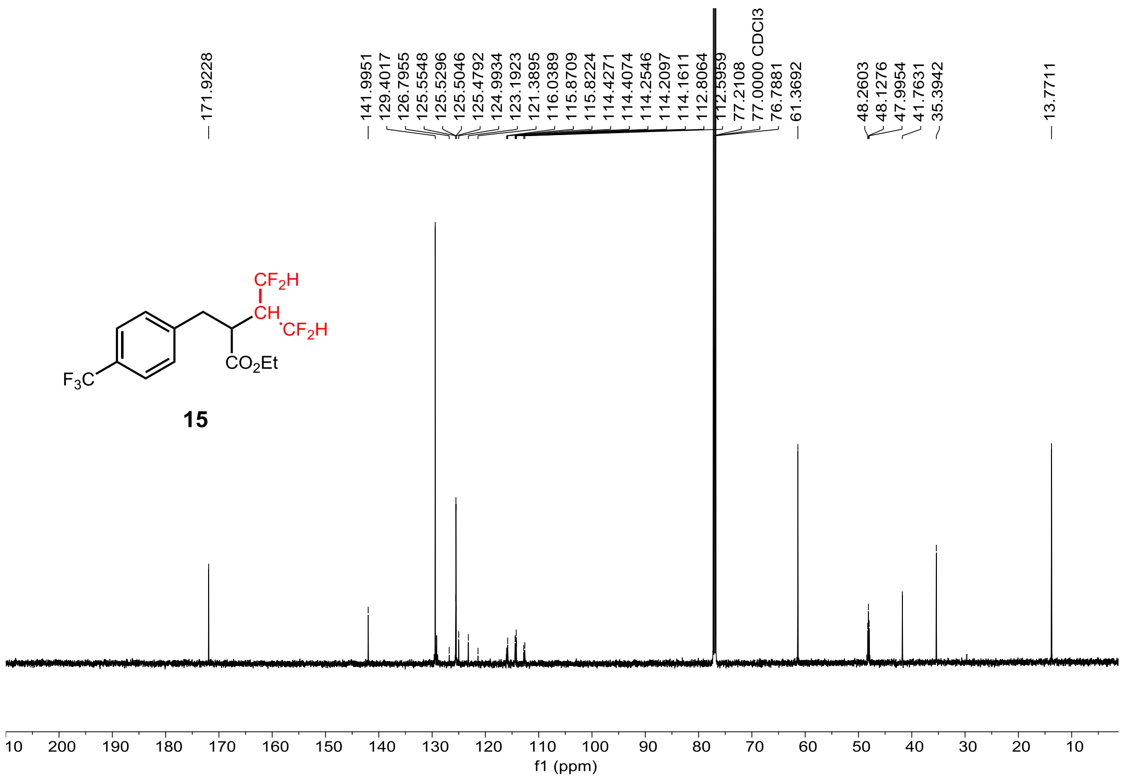

Supplementary Figure 41 ^{13}C NMR (151 MHz, $CDCl_3$) spectrum of compound 15.

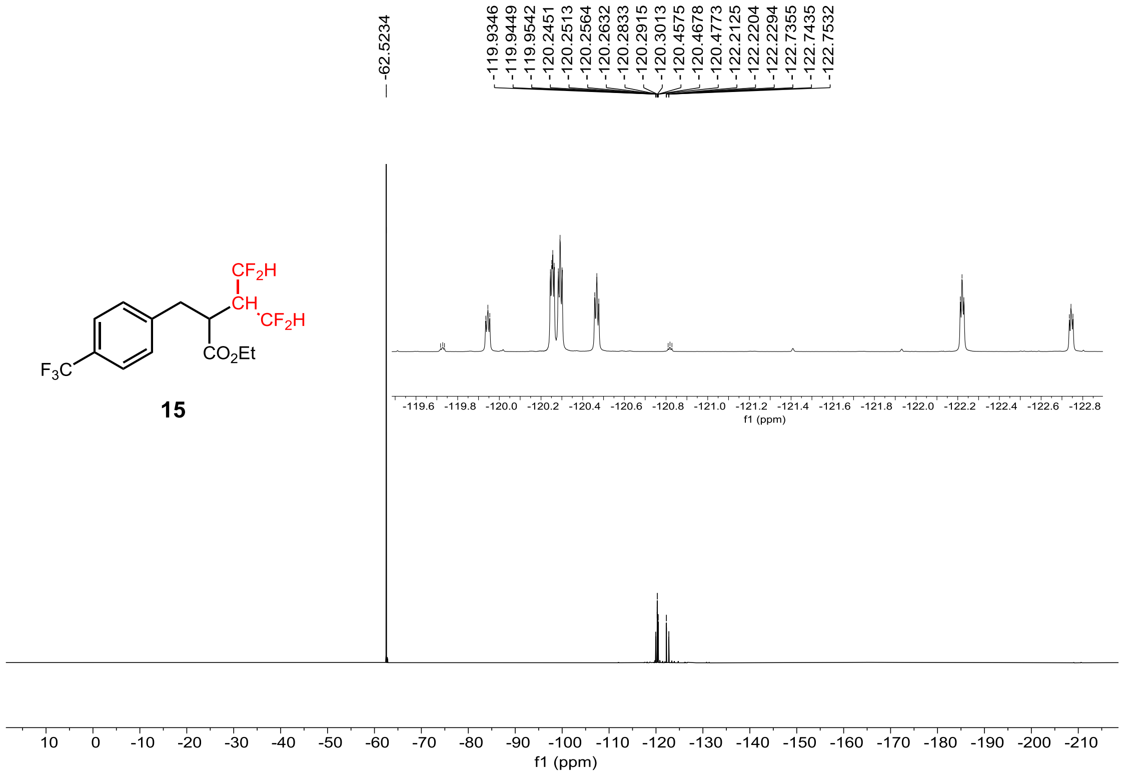

Supplementary Figure 42 ^{19}F NMR (565 MHz, $CDCl_3$) spectrum of compound 15.

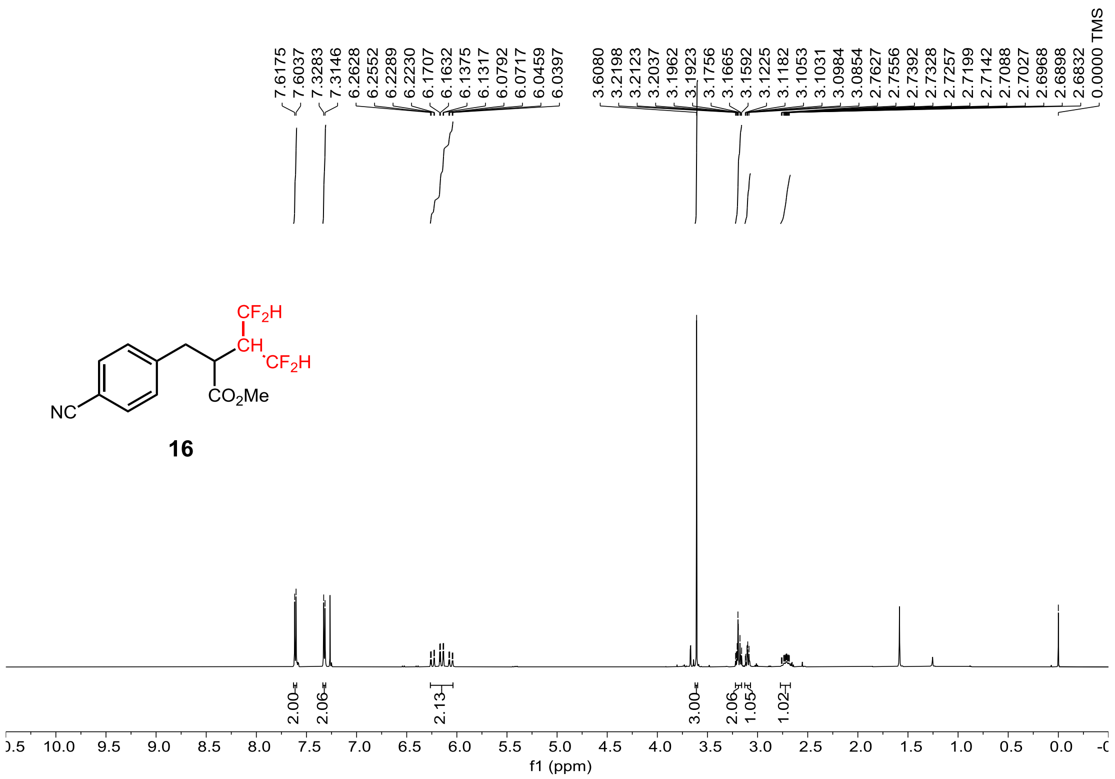

Supplementary Figure 43 ^{1}H NMR (600 MHz, $CDCl_3$) spectrum of compound 16.

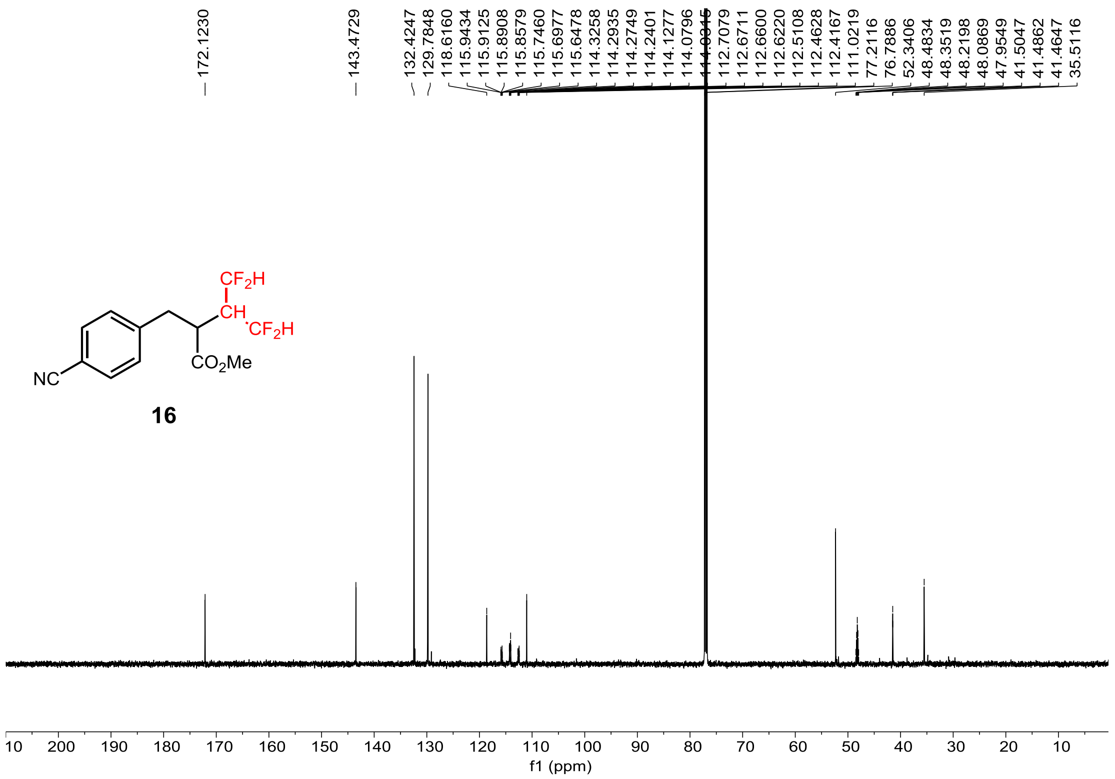

Supplementary Figure 44 ^{13}C NMR (151 MHz, $CDCl_3$) spectrum of compound 16.

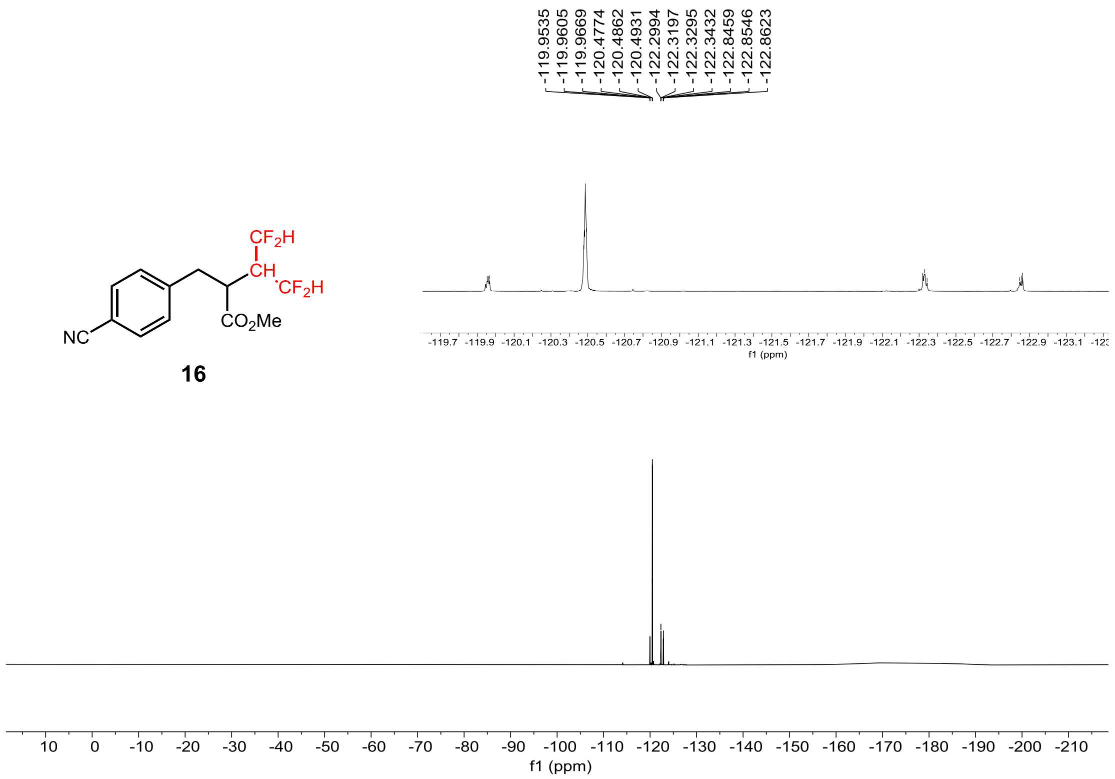

Supplementary Figure 45 ^{19}F NMR (565 MHz, $CDCl_3$) spectrum of compound 16.

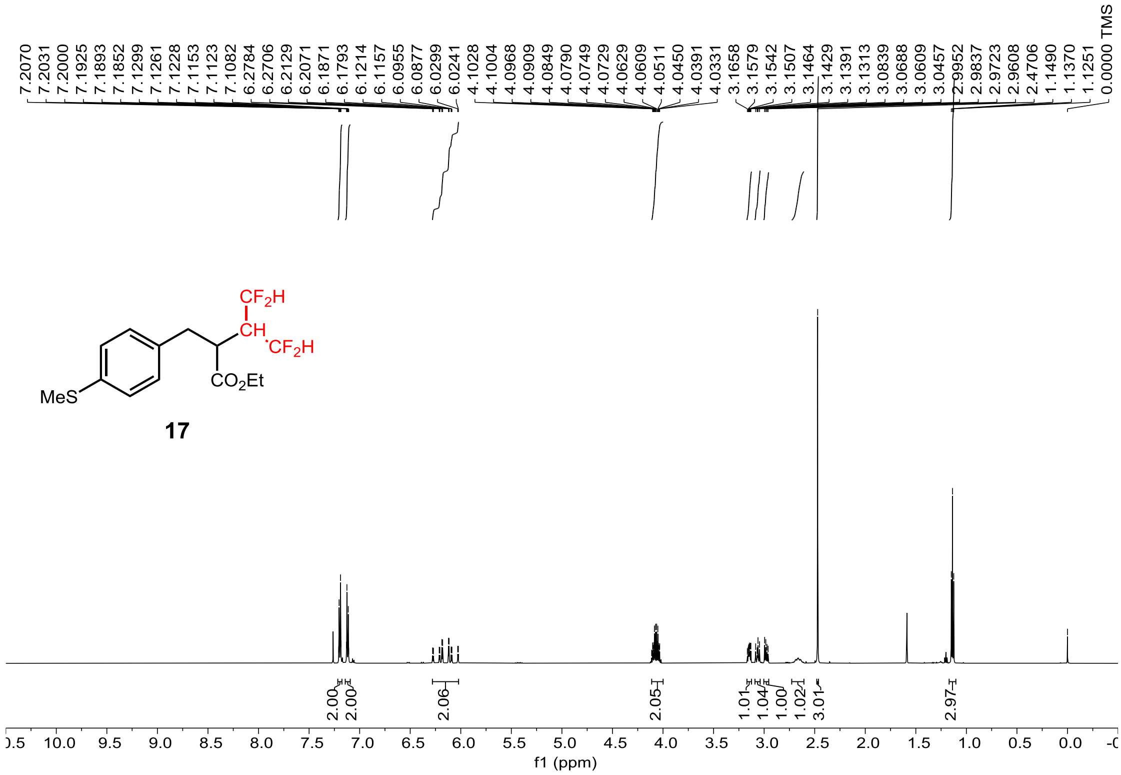

Supplementary Figure 46 ^{1}H NMR (600 MHz, $CDCl_3$) spectrum of compound 17.

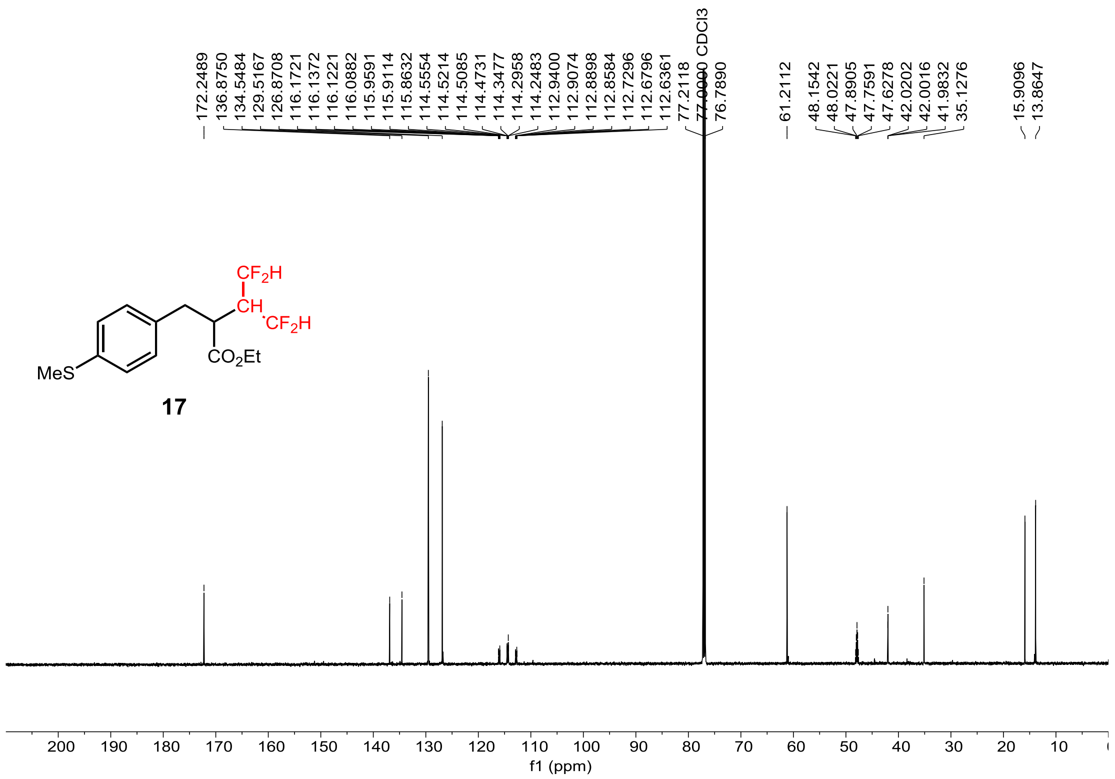

Supplementary Figure 47 ^{13}C NMR (151 MHz, $CDCl_3$) spectrum of compound 17.

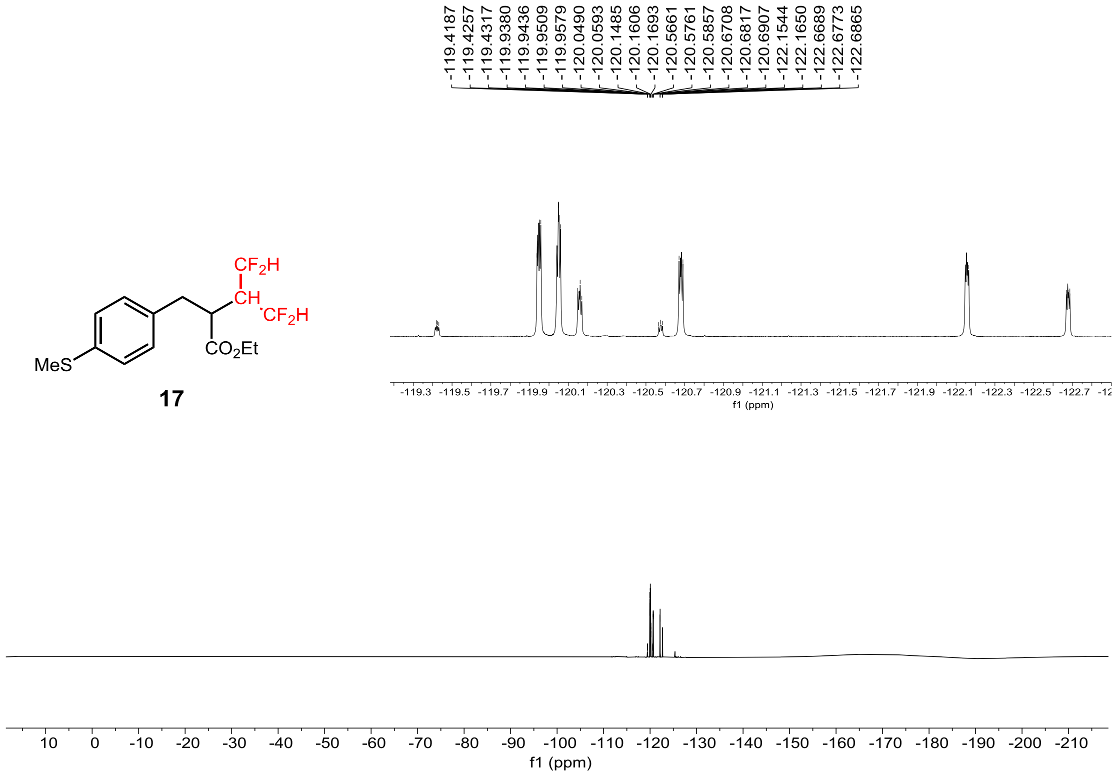

Supplementary Figure 48 ^{19}F NMR (565 MHz, $CDCl_3$) spectrum of compound 17.

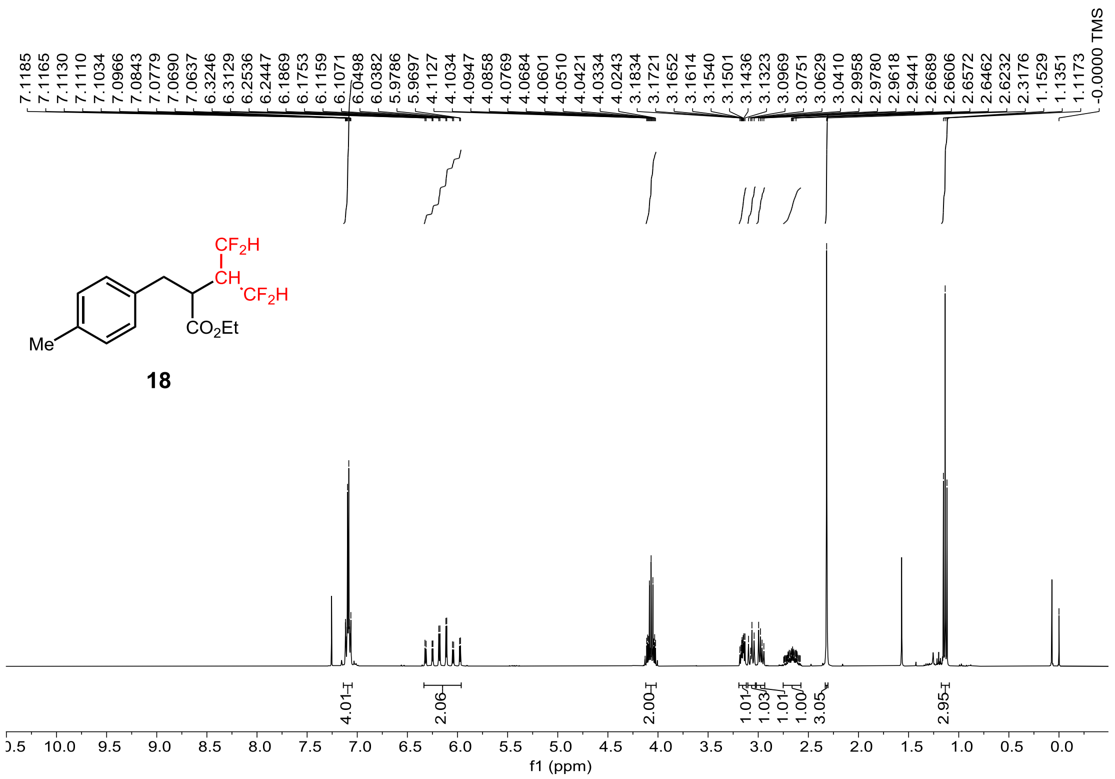

Supplementary Figure 49 ^{1}H NMR (600 MHz, $CDCl_3$) spectrum of compound 18.

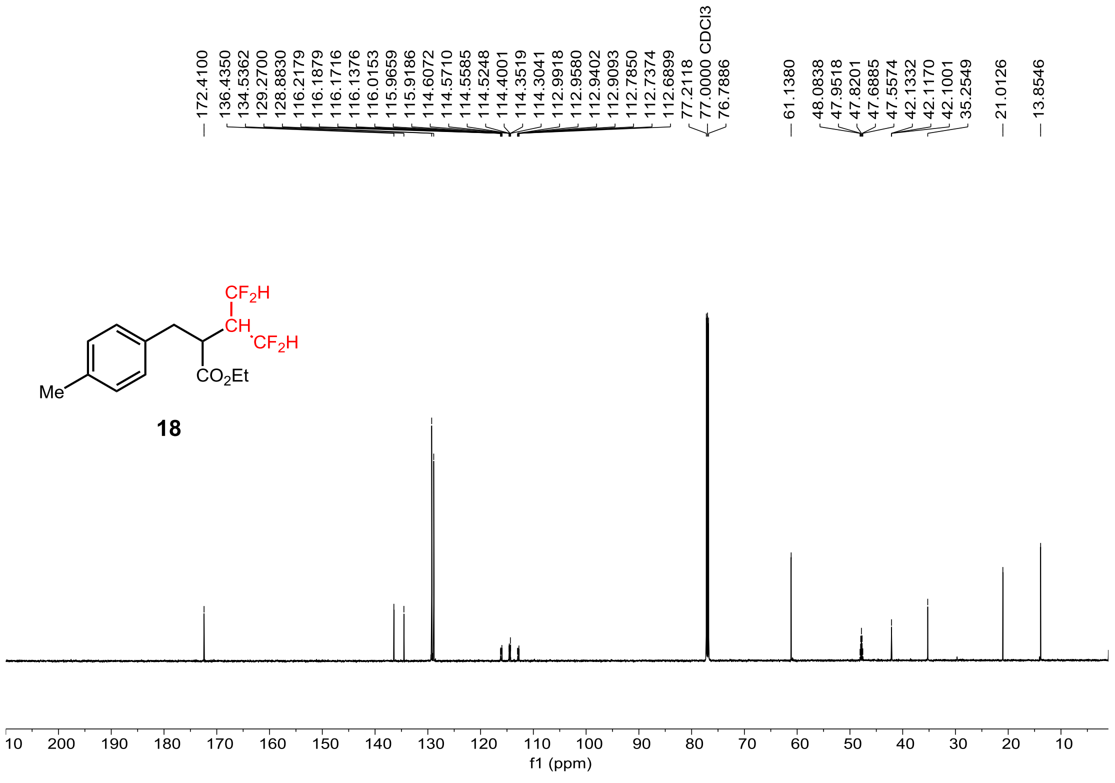

Supplementary Figure 50 ^{13}C NMR (151 MHz, $CDCl_3$) spectrum of compound 18.

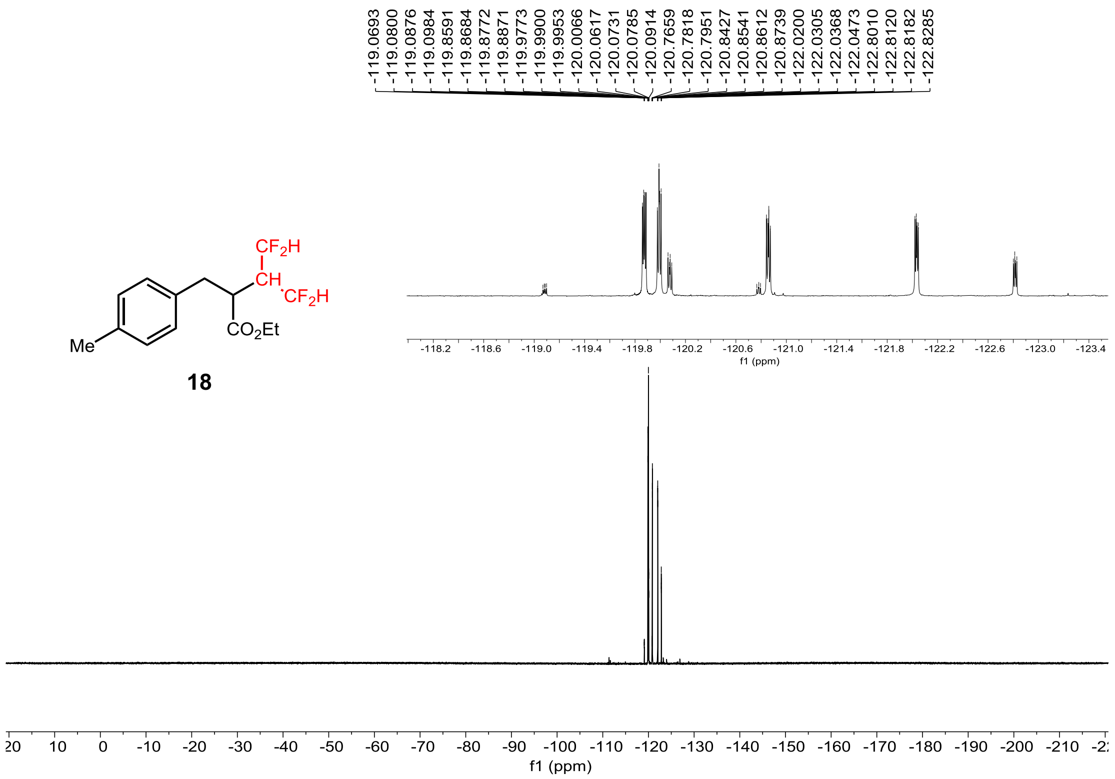

Supplementary Figure 51 ^{19}F NMR (377 MHz, CDCl$_3$) spectrum of compound 18.

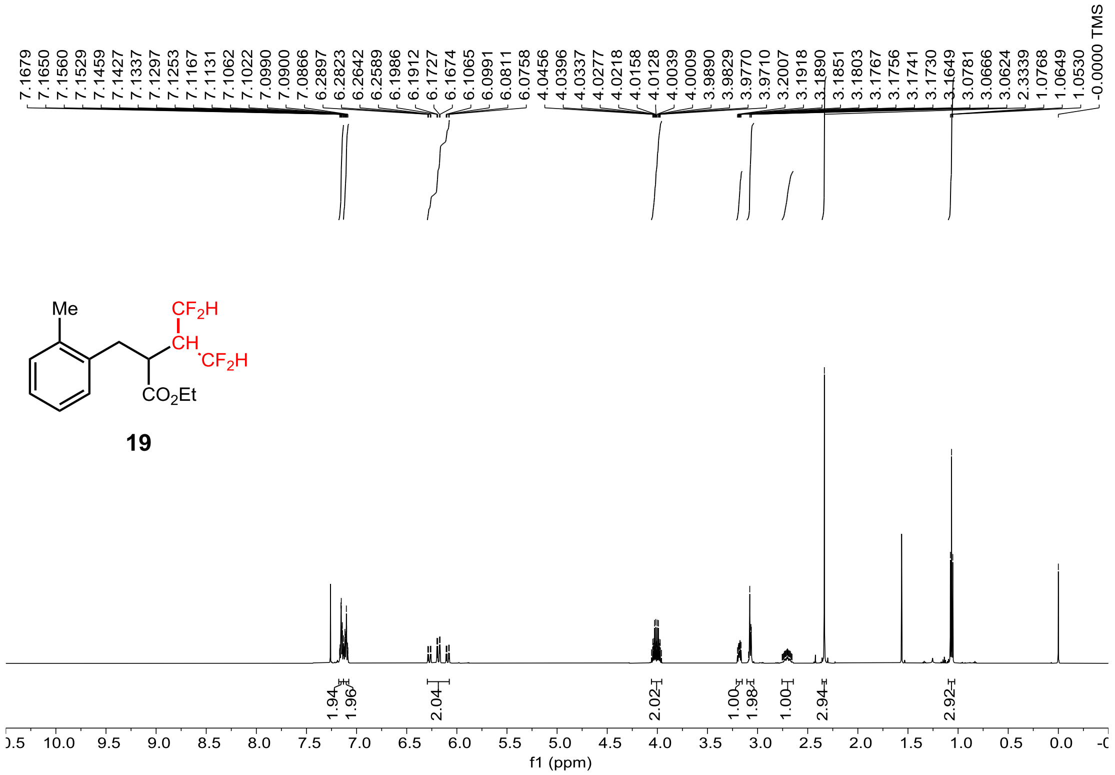

Supplementary Figure 52 ^{1}H NMR (600 MHz, CDCl$_3$) spectrum of compound 19.

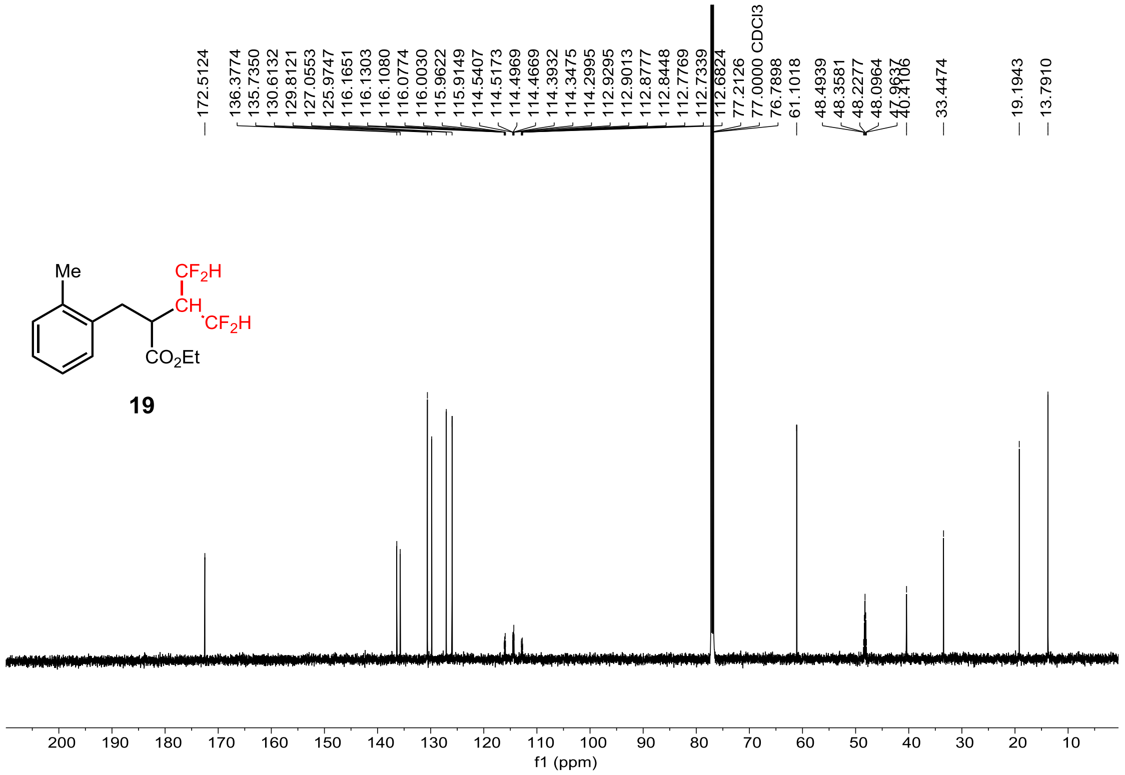

Supplementary Figure 53 ^{13}C NMR (151 MHz, $CDCl_3$) spectrum of compound 19.

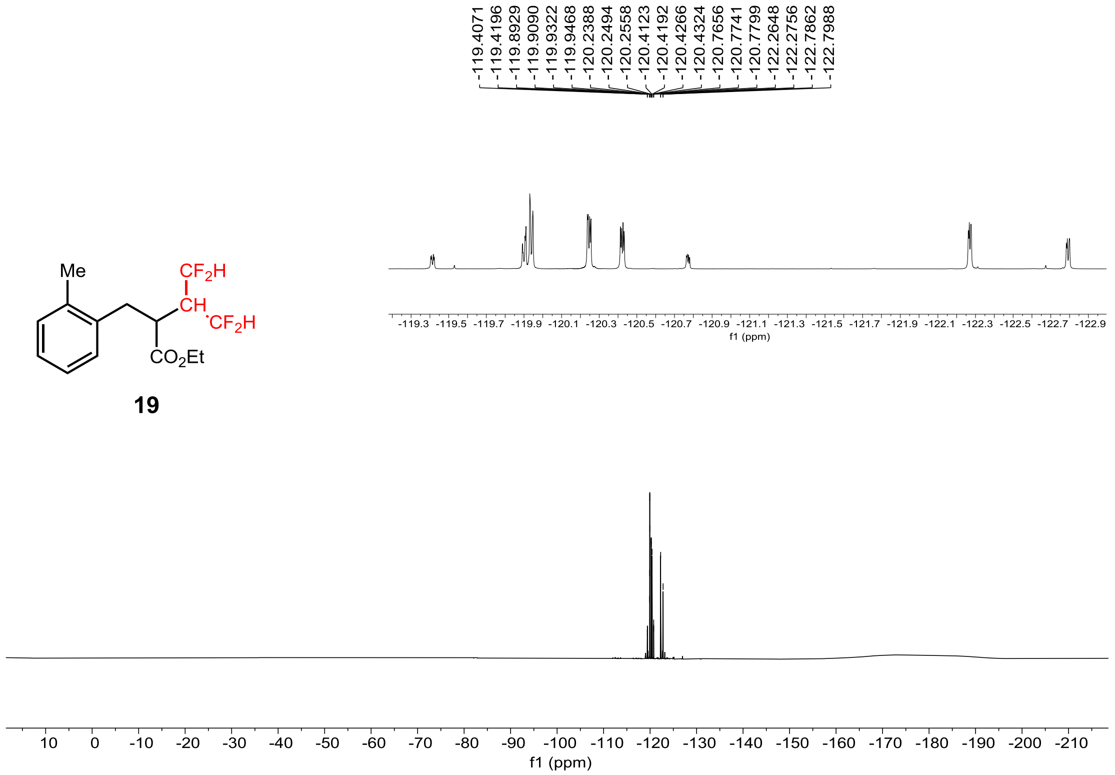

Supplementary Figure 54 ^{19}F NMR (565 MHz, $CDCl_3$) spectrum of compound 19.

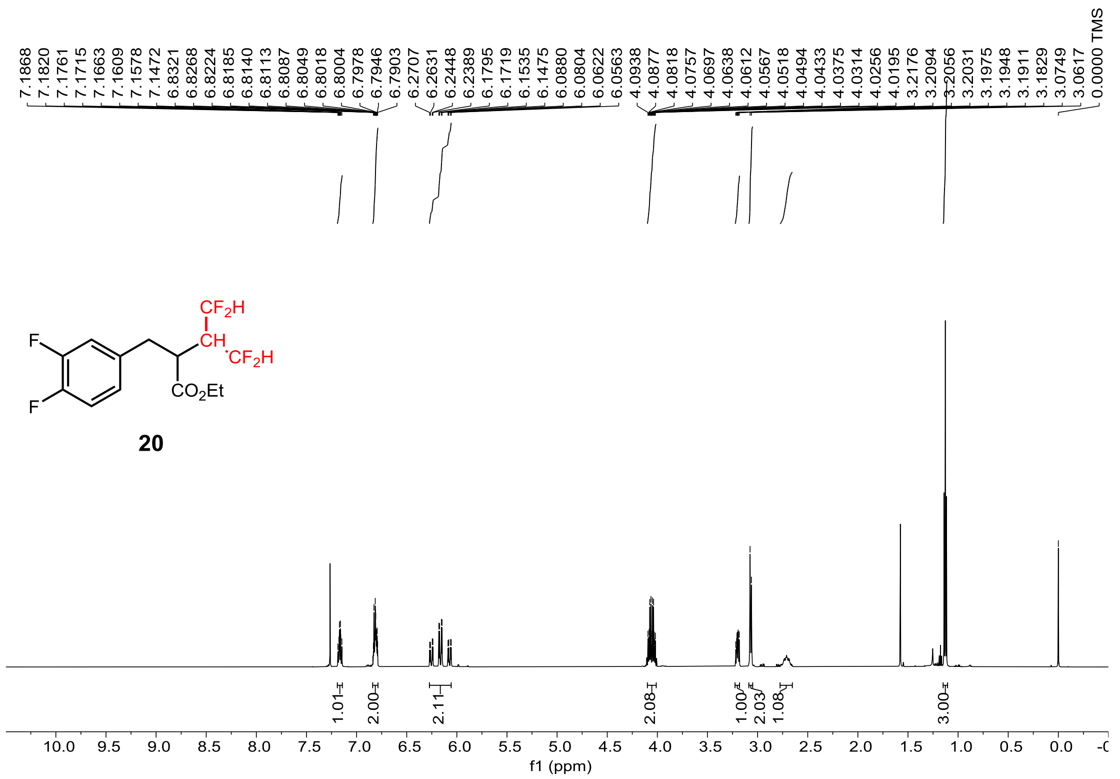

Supplementary Figure 55 ^{1}H NMR (600 MHz, $CDCl_3$) spectrum of compound 20.

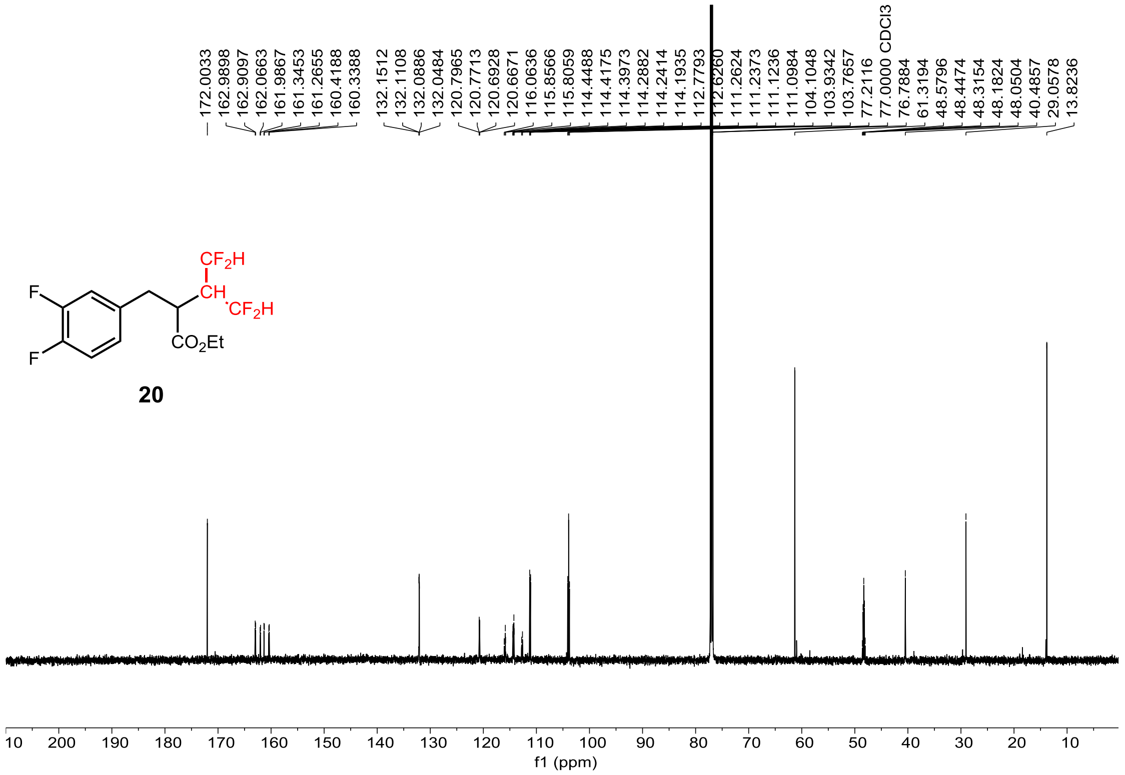

Supplementary Figure 56 ^{13}C NMR (151 MHz, $CDCl_3$) spectrum of compound 20.

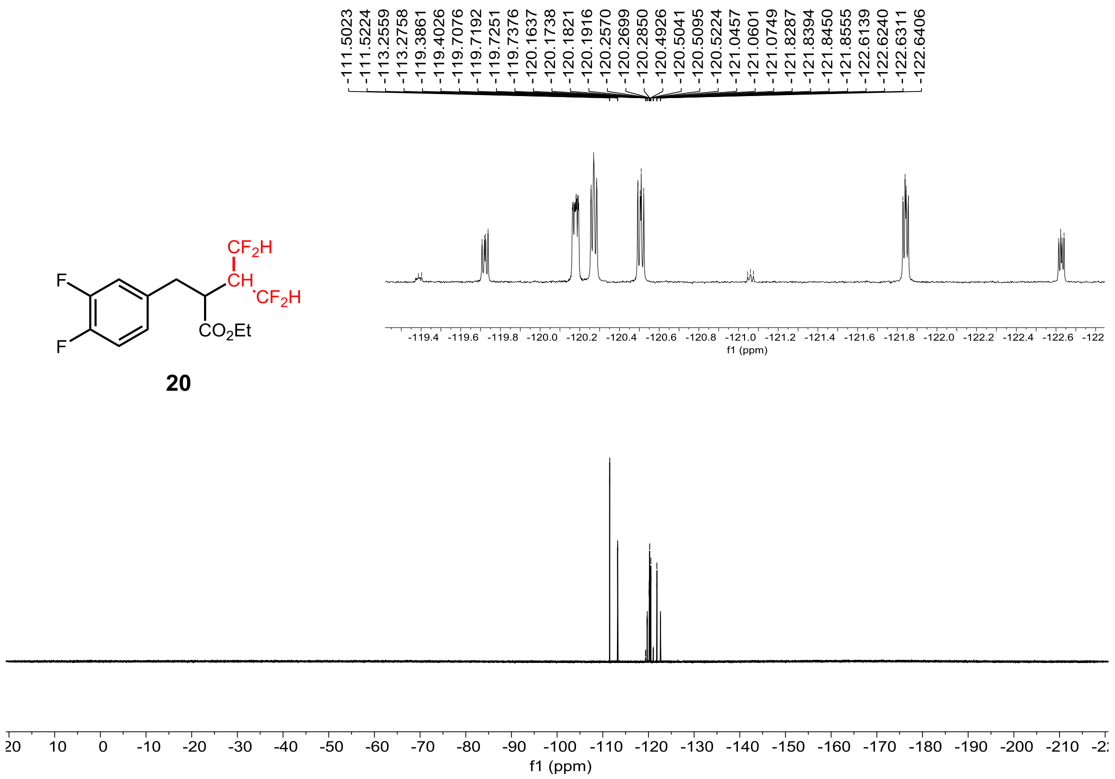

Supplementary Figure 57 ^{19}F NMR (377 MHz, $CDCl_3$) spectrum of compound 20.

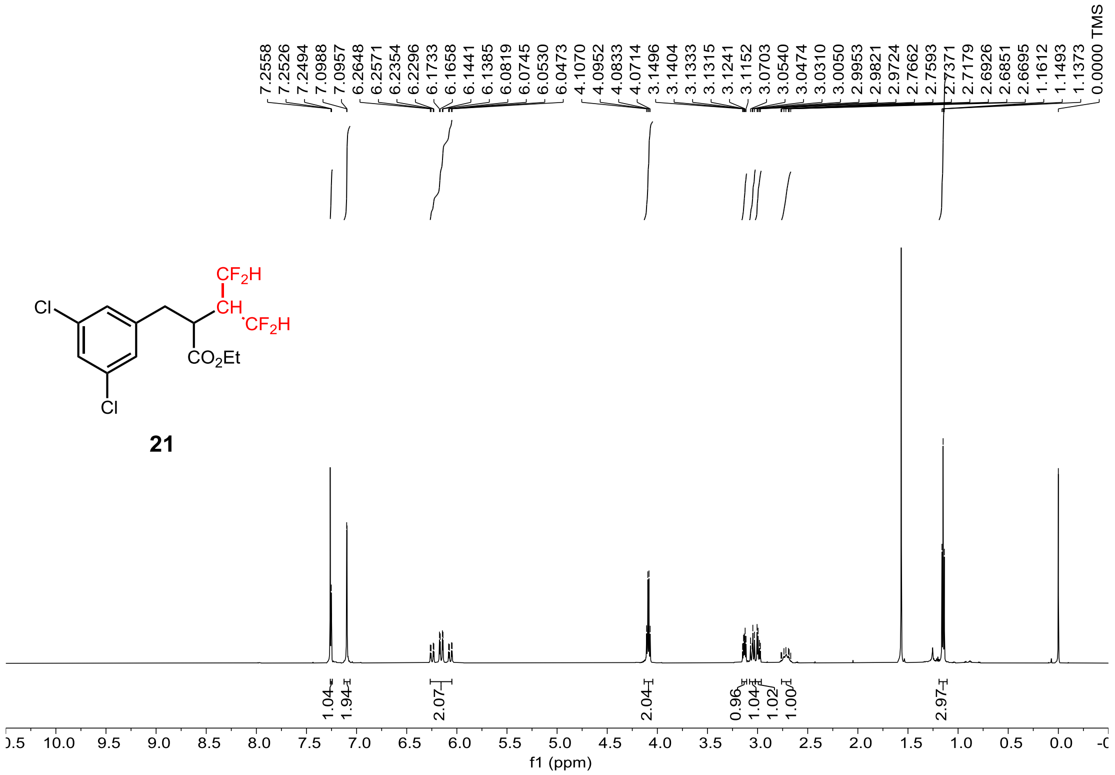

Supplementary Figure 58 ^{1}H NMR (600 MHz, $CDCl_3$) spectrum of compound 21.

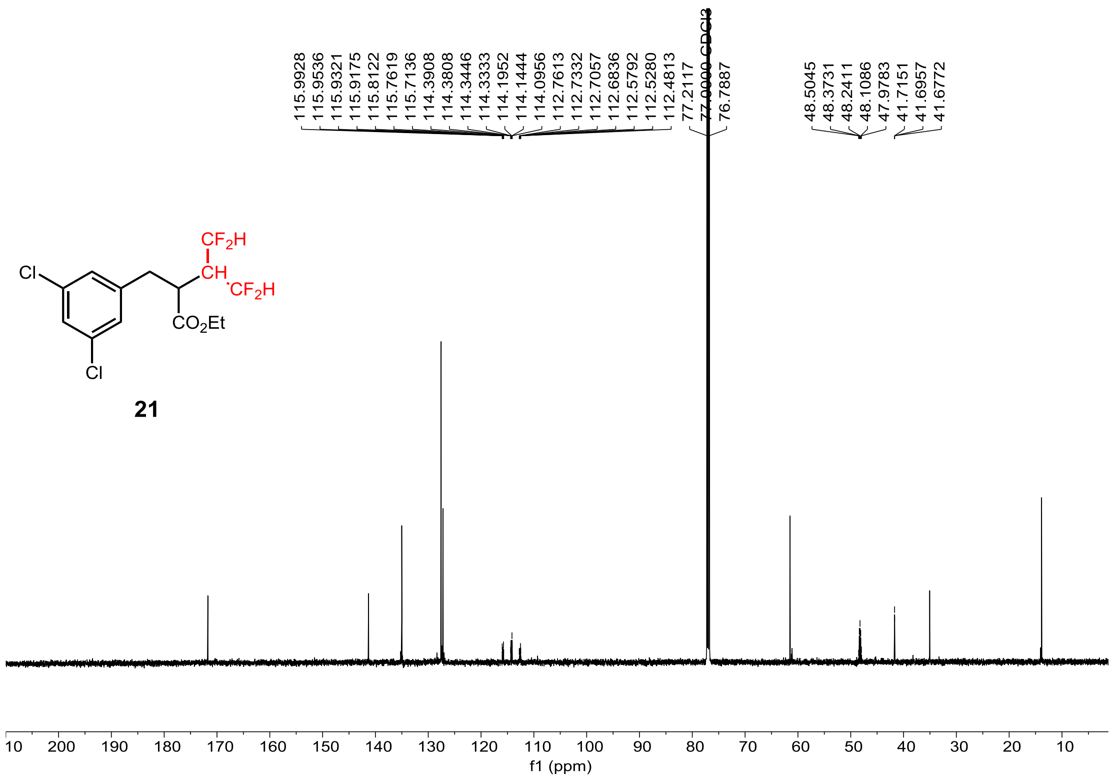

Supplementary Figure 59 ^{13}C NMR (151 MHz, $CDCl_3$) spectrum of compound 21.

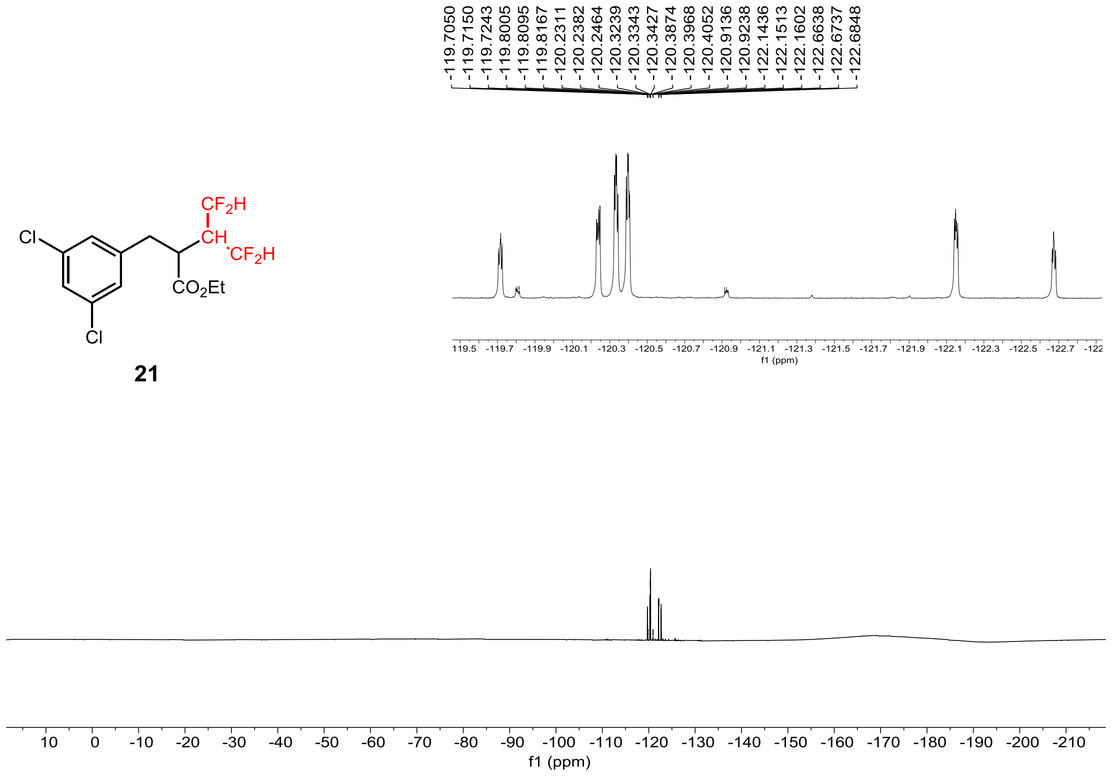

Supplementary Figure 60 ^{19}F NMR (565 MHz, $CDCl_3$) spectrum of compound 21.

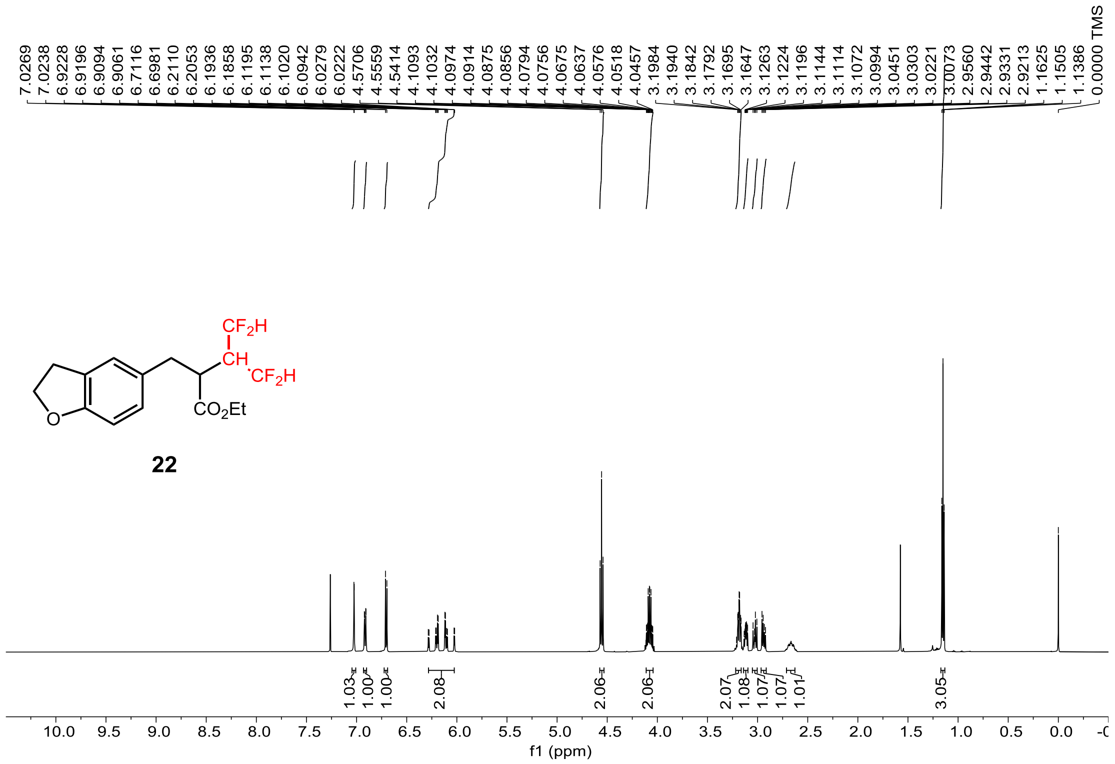

Supplementary Figure 61 ^{1}H NMR (600 MHz, $CDCl_3$) spectrum of compound 22.

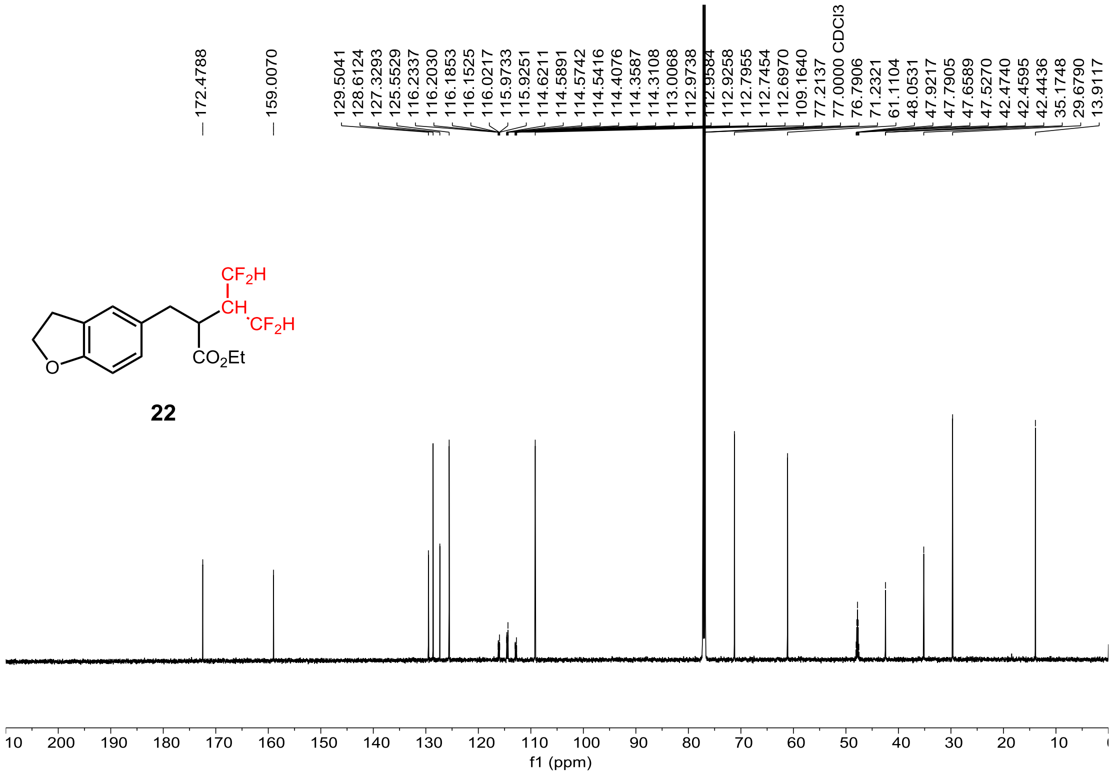

Supplementary Figure 62 ^{13}C NMR (151 MHz, $CDCl_3$) spectrum of compound 22.

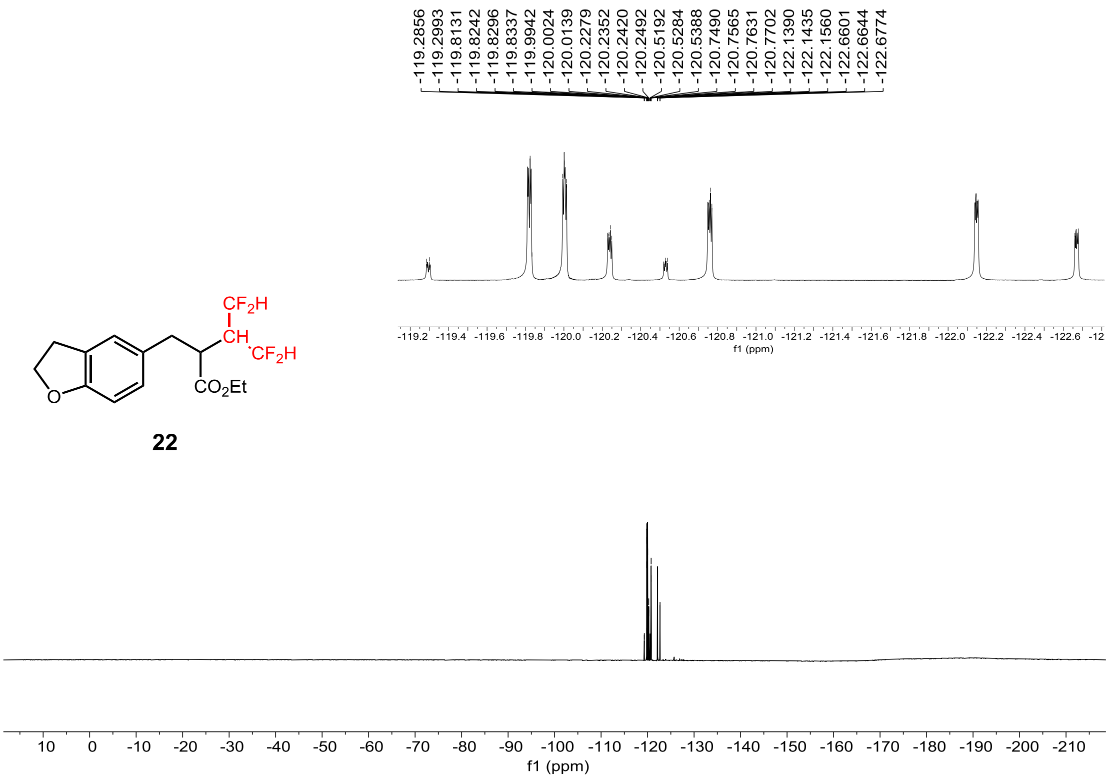

Supplementary Figure 63 ^{19}F NMR (565 MHz, $CDCl_3$) spectrum of compound 22.

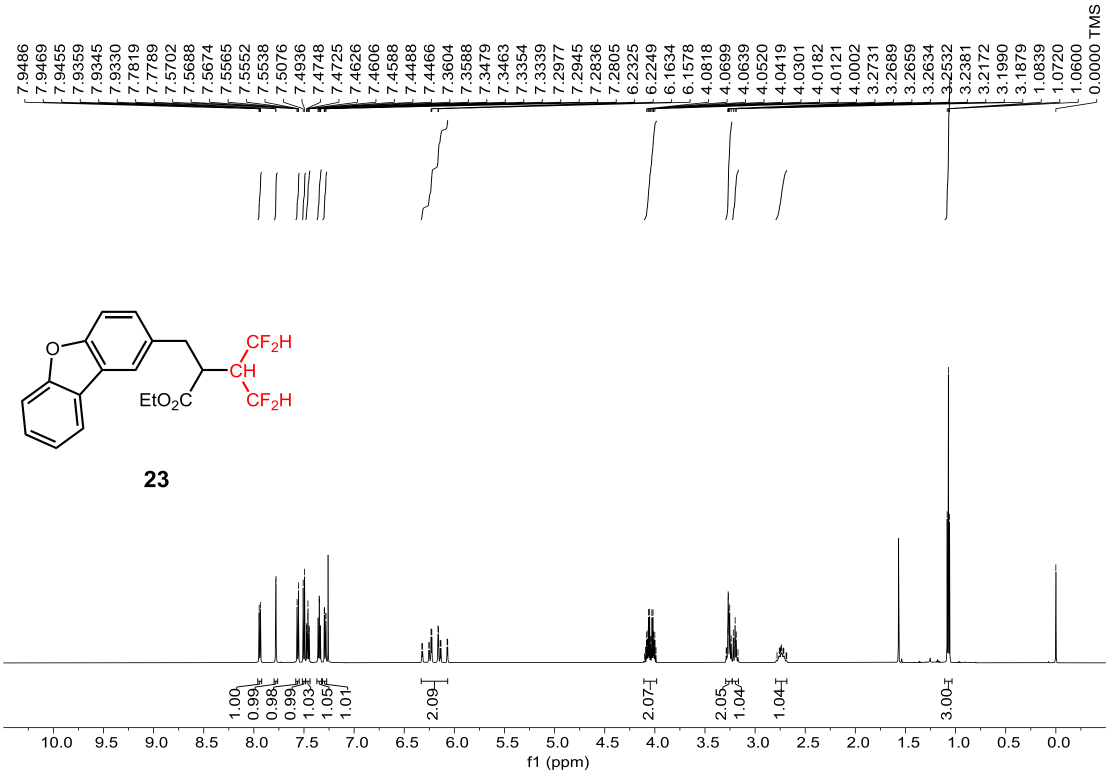

Supplementary Figure 64 ^{1}H NMR (600 MHz, $CDCl_3$) spectrum of compound 23.

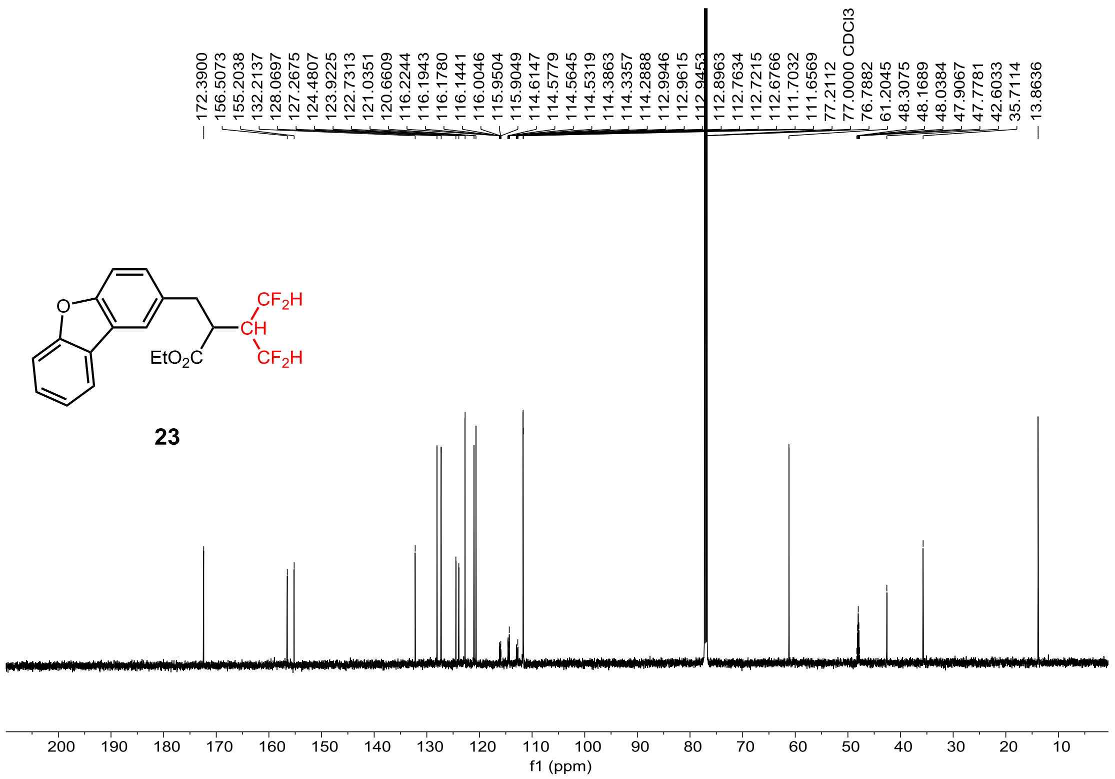

Supplementary Figure 65 ^{13}C NMR (151 MHz, CDCl$_3$) spectrum of compound 23.

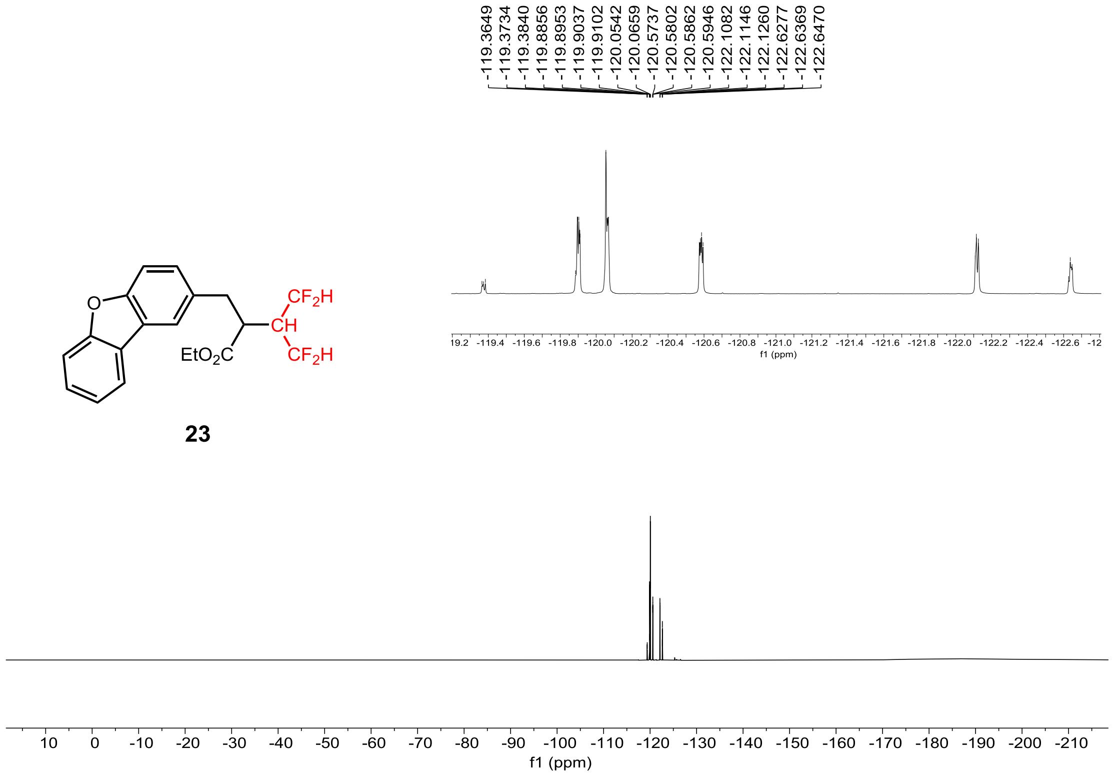

Supplementary Figure 66 ^{19}F NMR (565 MHz, CDCl$_3$) spectrum of compound 23.

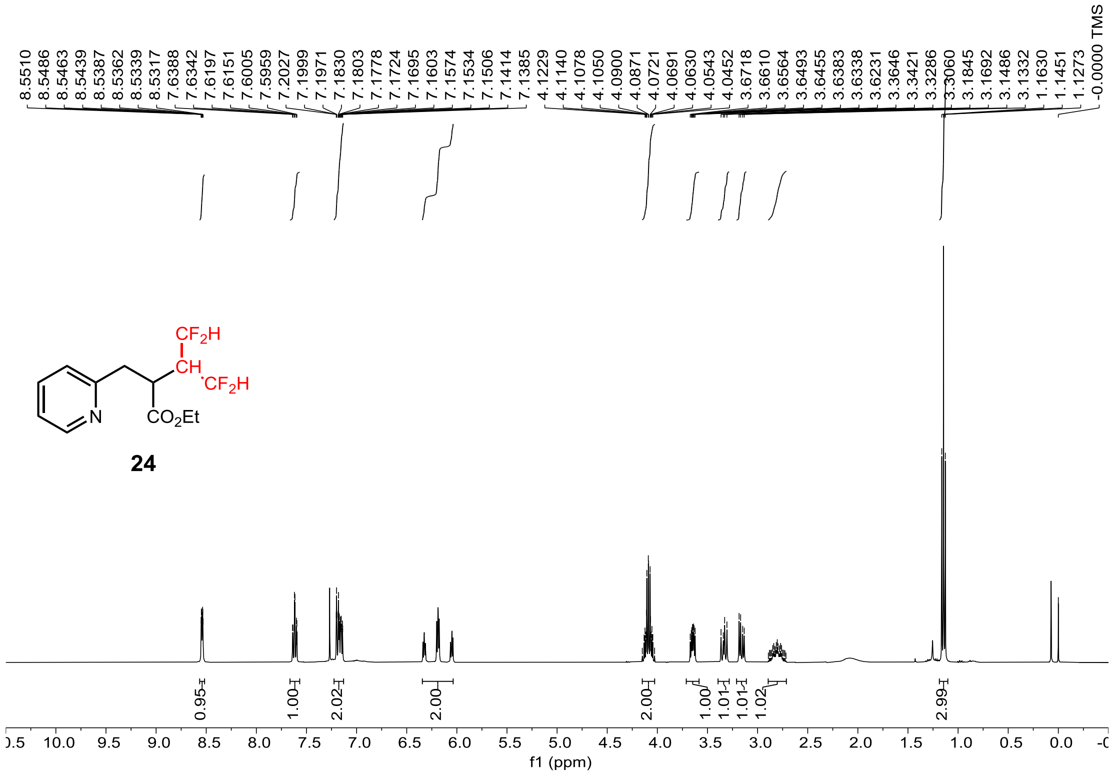

Supplementary Figure 67 ^{1}H NMR (400 MHz, $CDCl_3$) spectrum of compound 24.

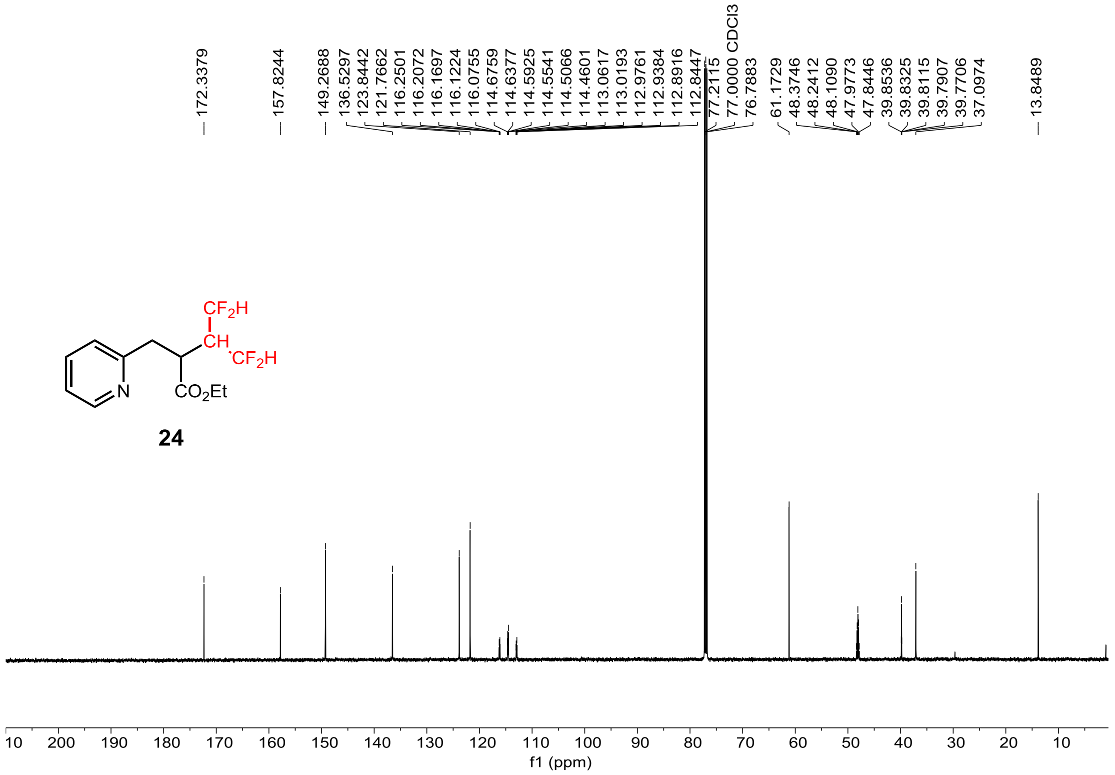

Supplementary Figure 68 ^{13}C NMR (151 MHz, $CDCl_3$) spectrum of compound 24.

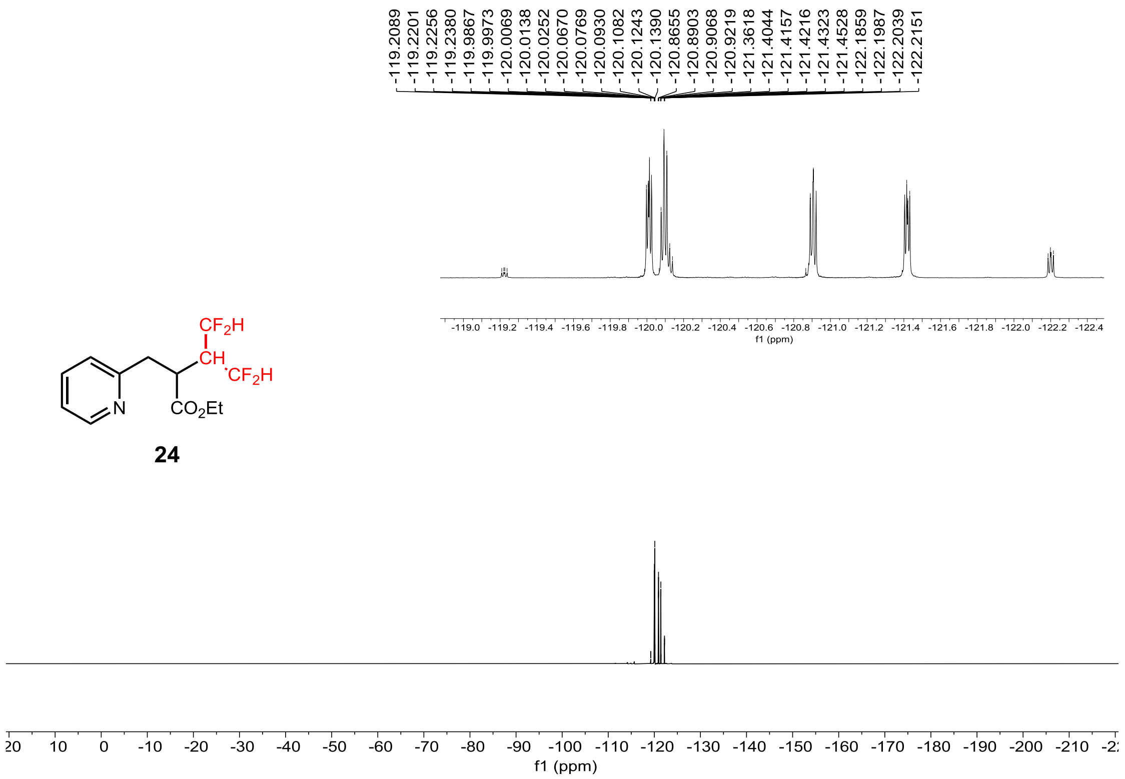

Supplementary Figure 69 ^{19}F NMR (565 MHz, $CDCl_3$) spectrum of compound 24.

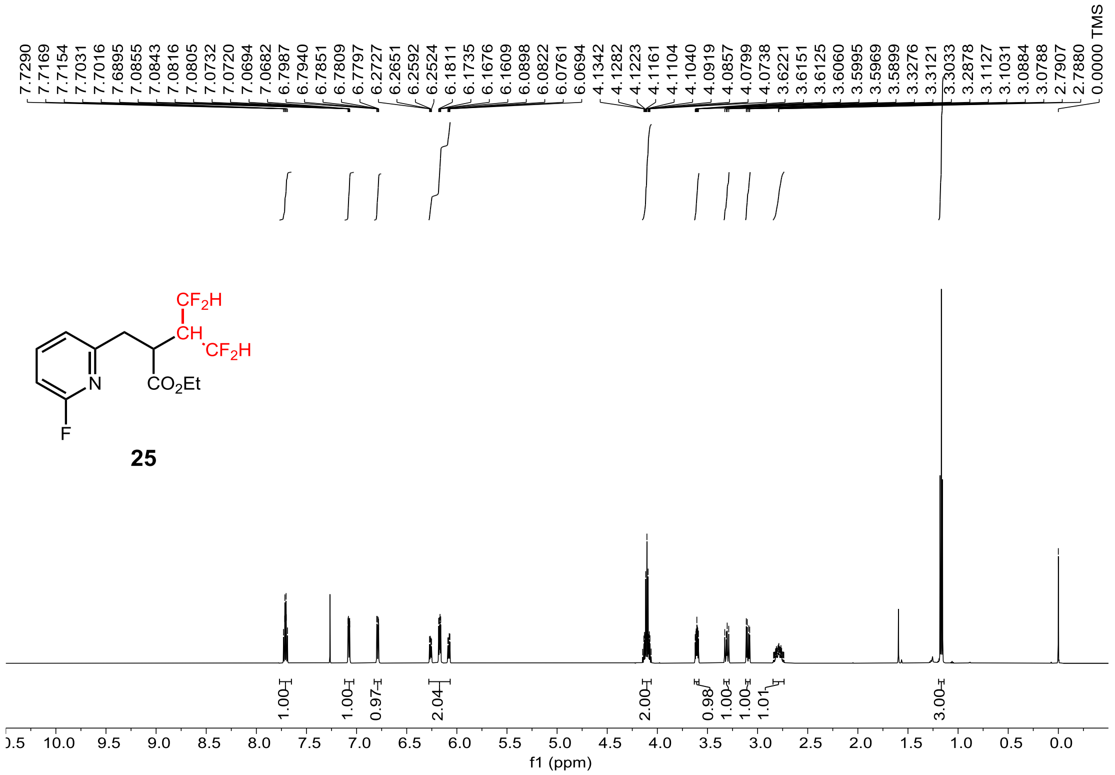

Supplementary Figure 70 ^{1}H NMR (600 MHz, $CDCl_3$) spectrum of compound 25.

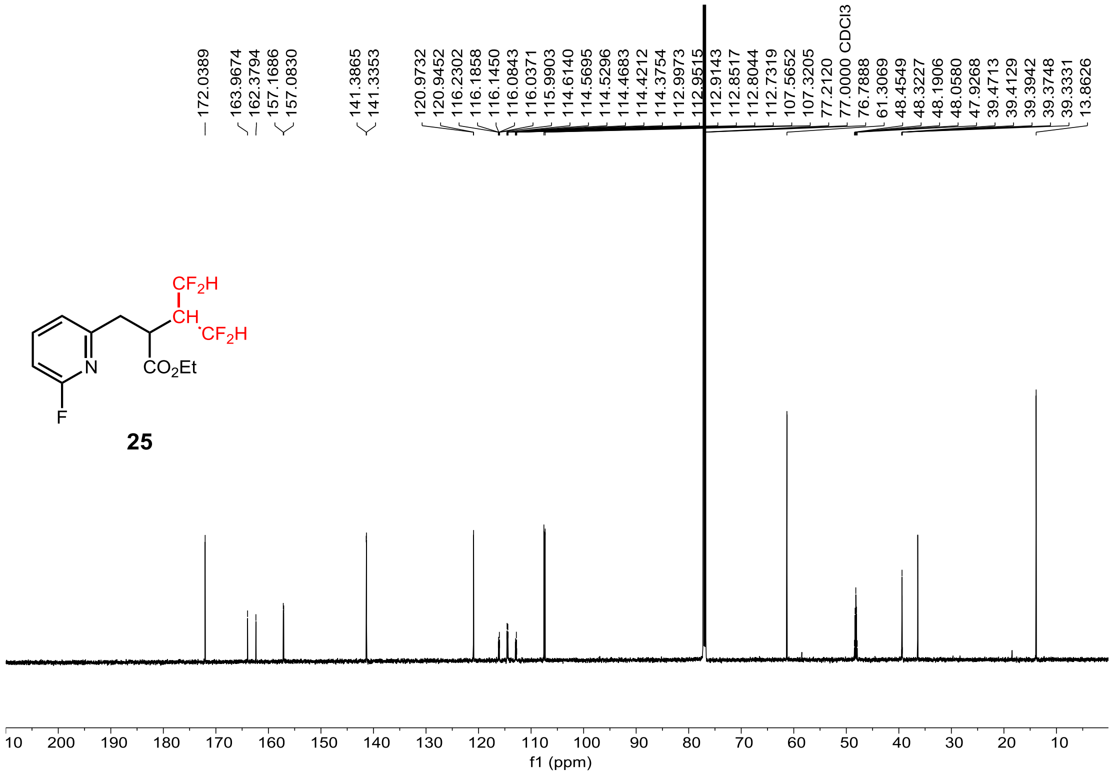

Supplementary Figure 71 ^{13}C NMR (151 MHz, CDCl$_3$) spectrum of compound 25.

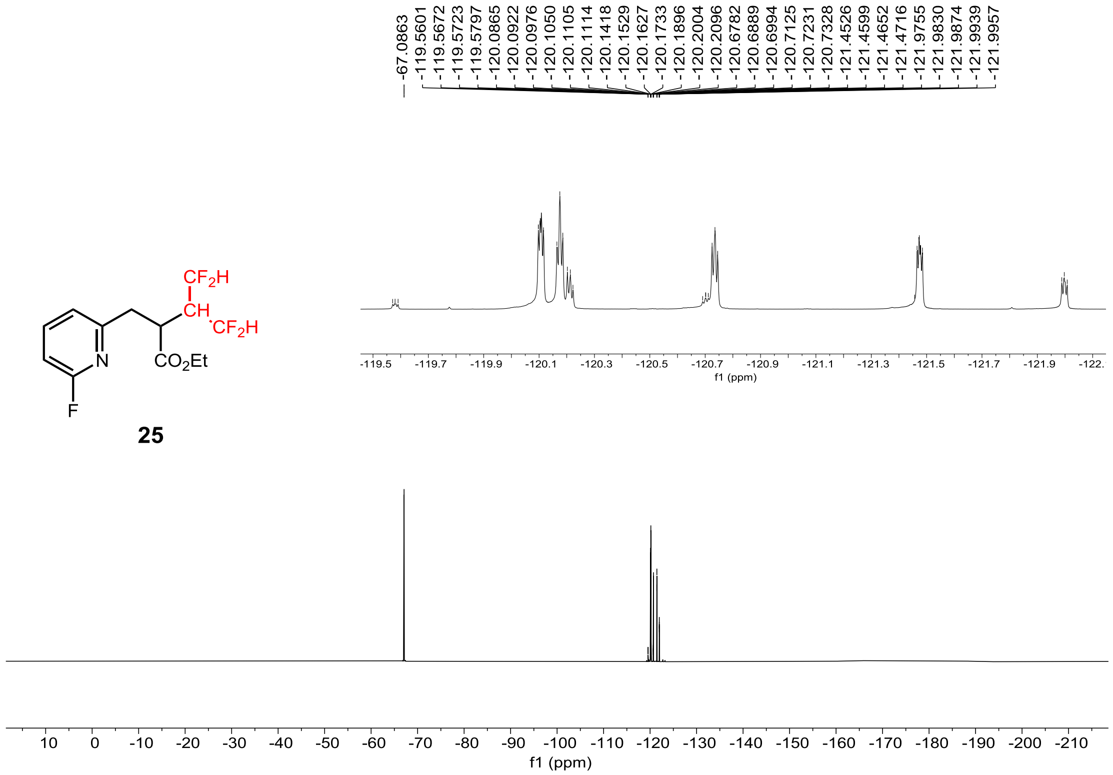

Supplementary Figure 72 ^{19}F NMR (565 MHz, CDCl$_3$) spectrum of compound 25.

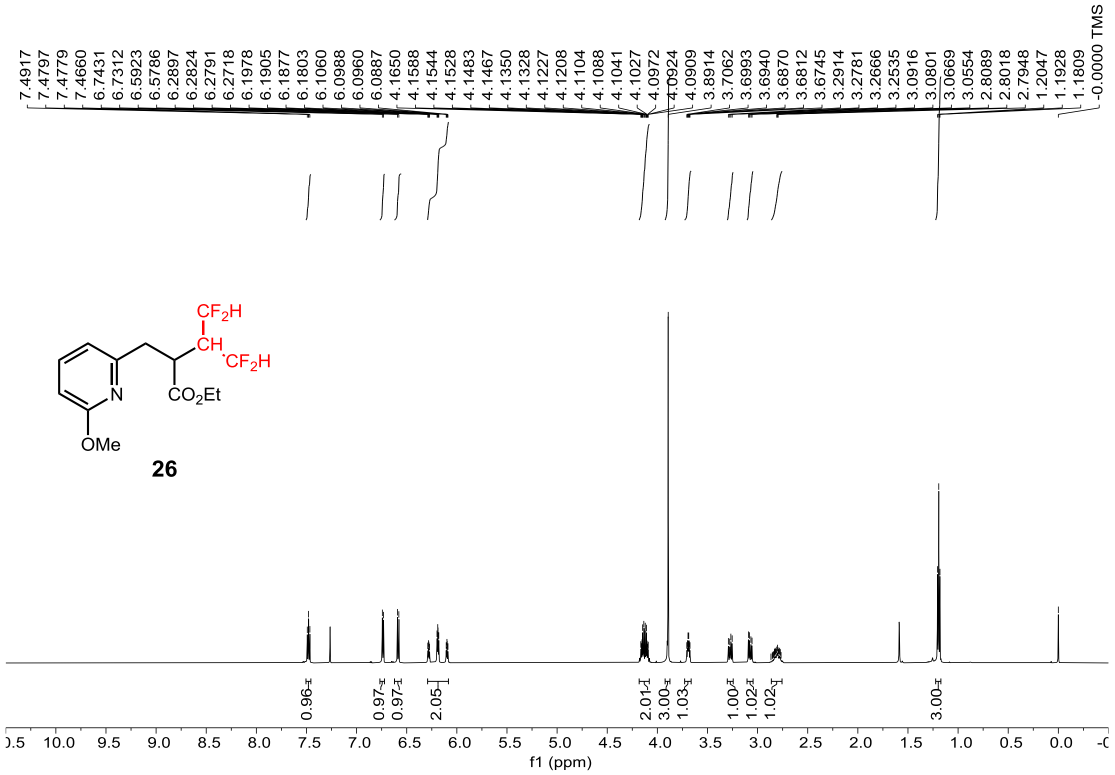

Supplementary Figure 73 ^{1}H NMR (600 MHz, $CDCl_3$) spectrum of compound 26.

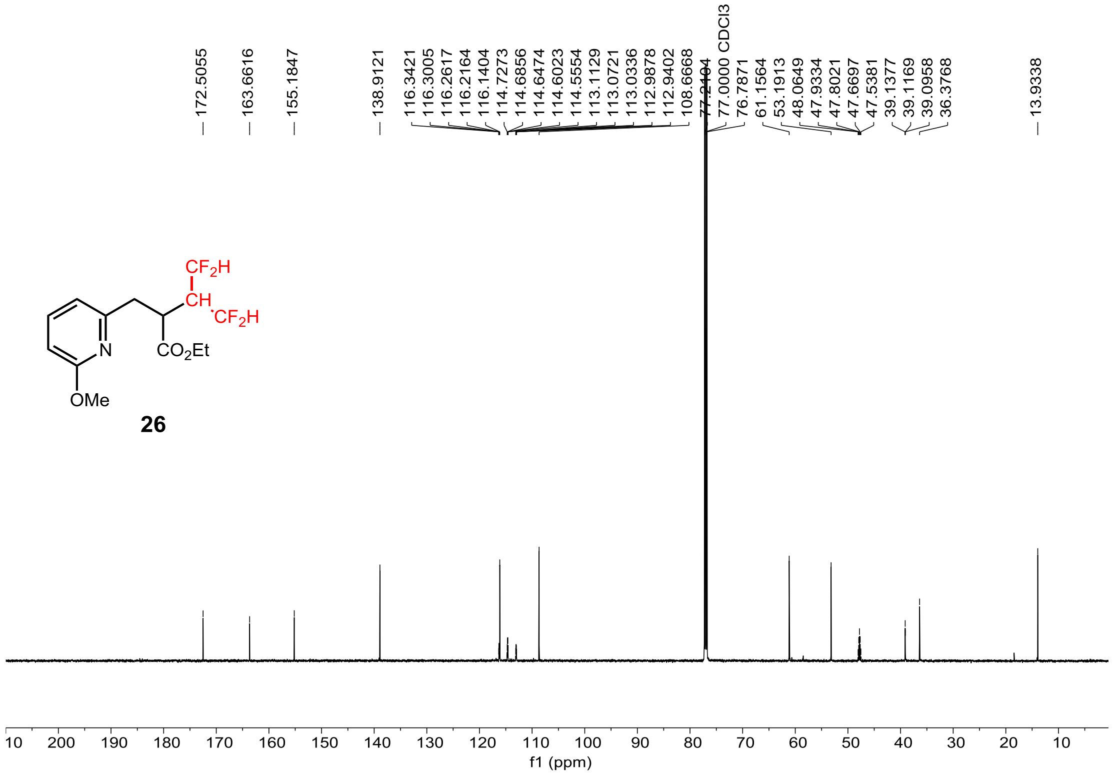

Supplementary Figure 74 ^{13}C NMR (151 MHz, $CDCl_3$) spectrum of compound 26.

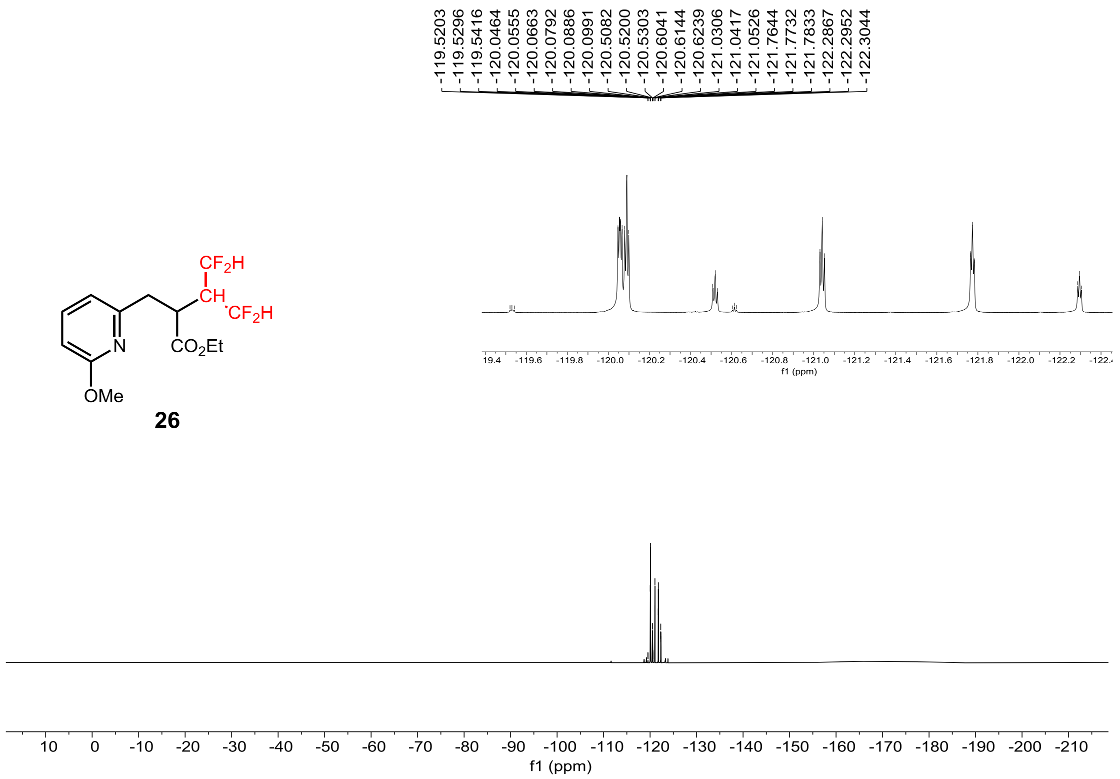

Supplementary Figure 75 ^{19}F NMR (565 MHz, $CDCl_3$) spectrum of compound 26.

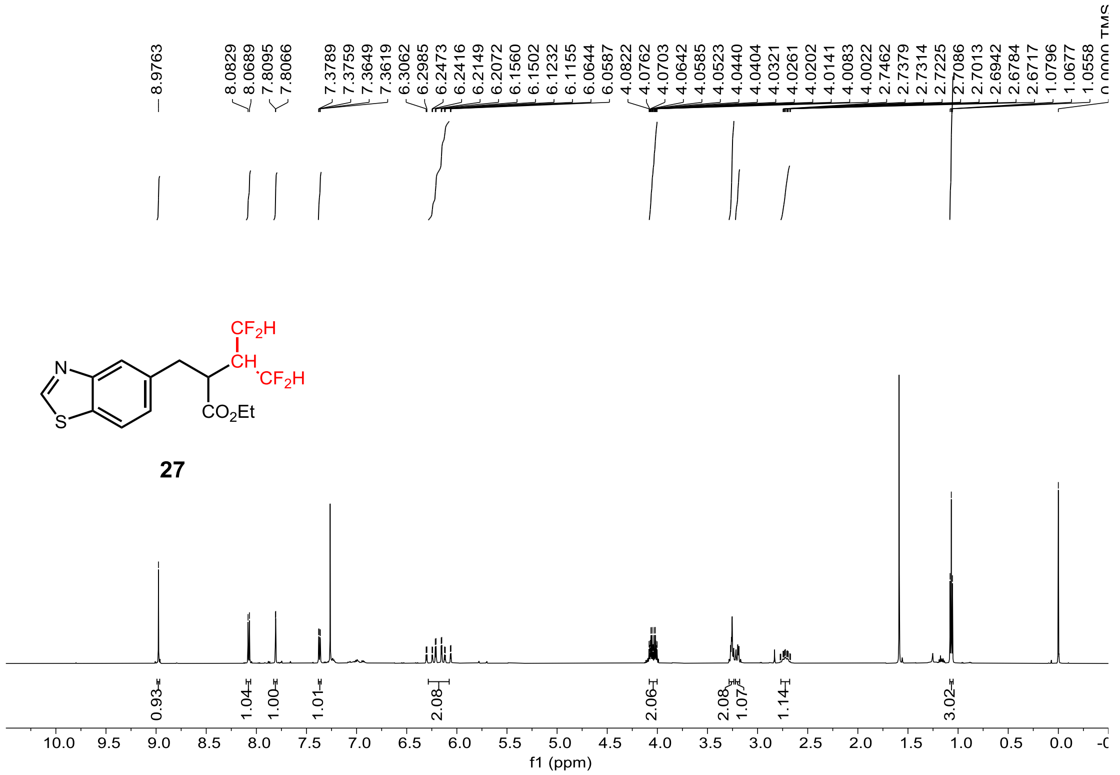

Supplementary Figure 76 ^{1}H NMR (600 MHz, $CDCl_3$) spectrum of compound 27.

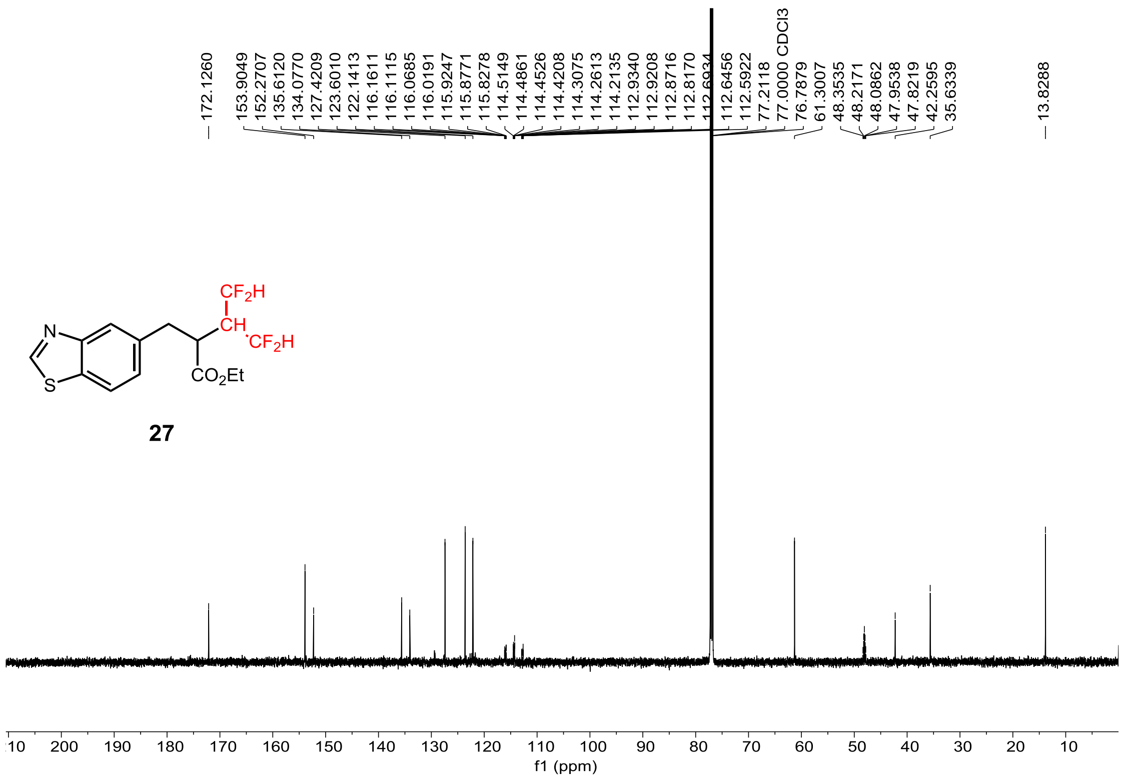

Supplementary Figure 77 ^{13}C NMR (151 MHz, $CDCl_3$) spectrum of compound 27.

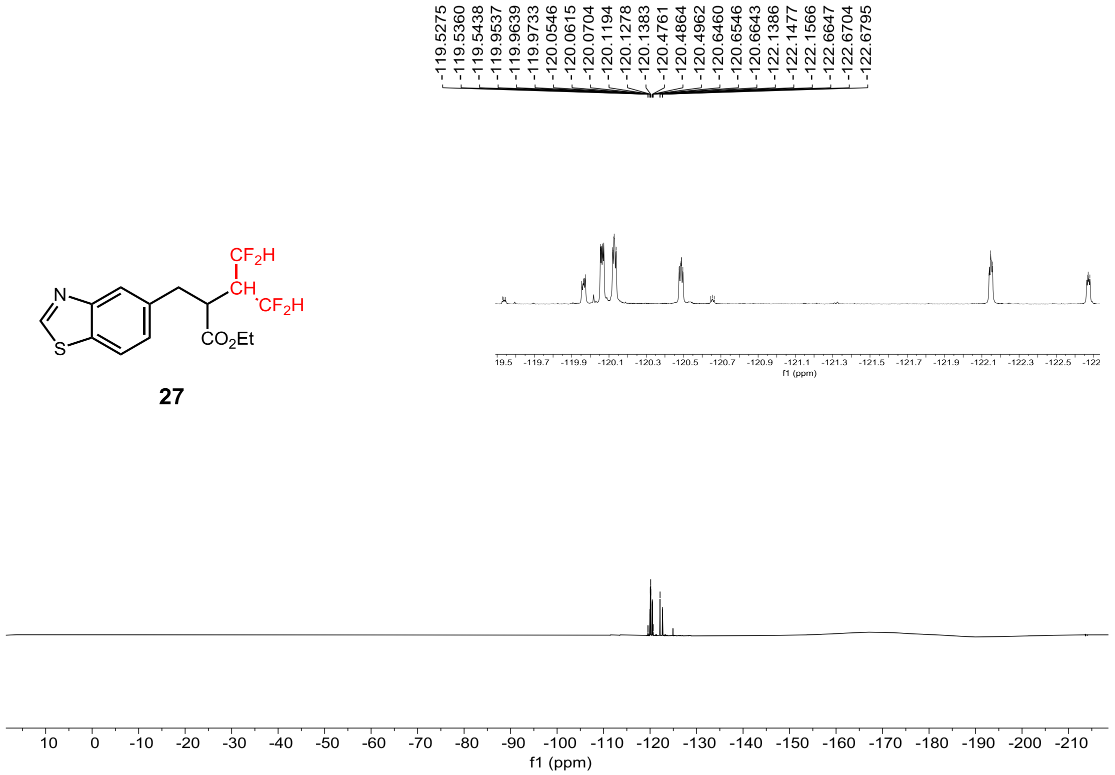

Supplementary Figure 78 ^{19}F NMR (565 MHz, $CDCl_3$) spectrum of compound 27.

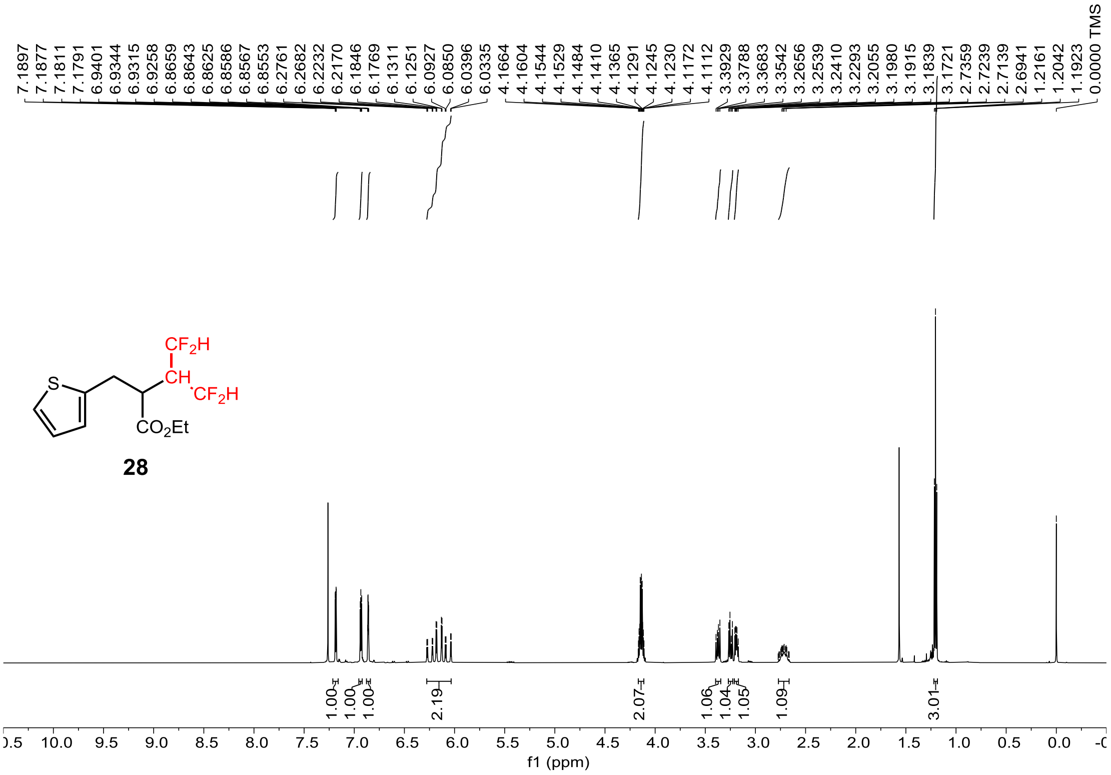

Supplementary Figure 79 ^{1}H NMR (600 MHz, $CDCl_3$) spectrum of compound 28.

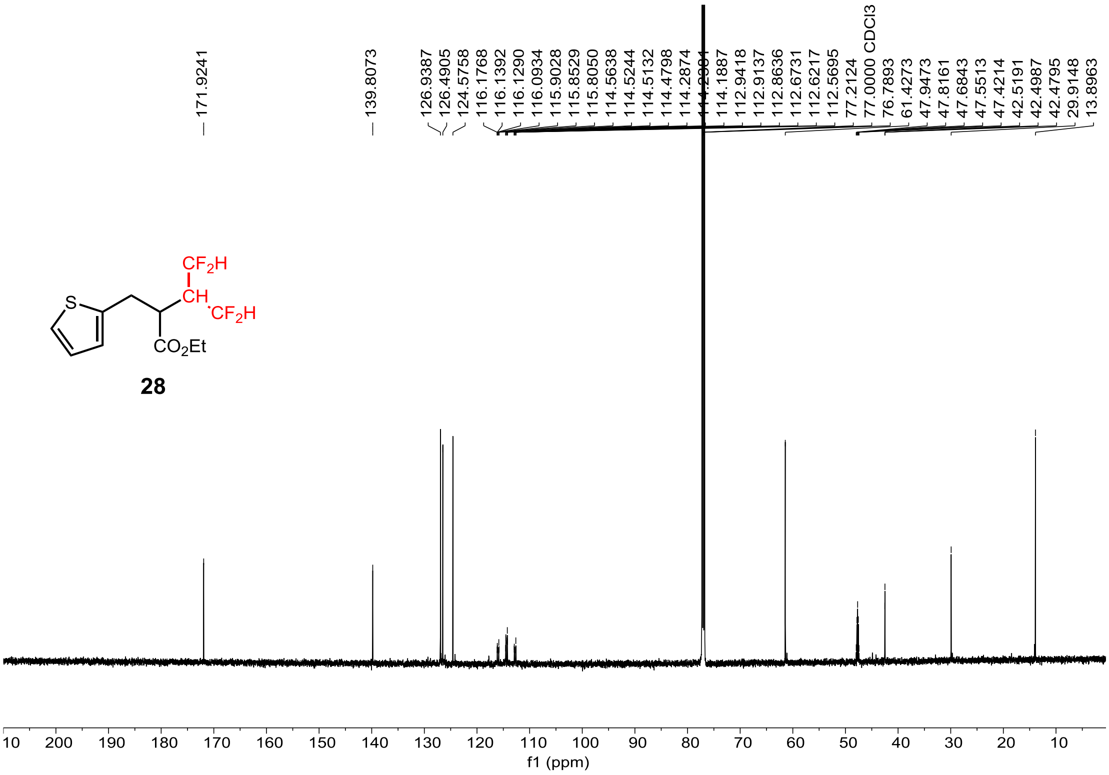

Supplementary Figure 80 ^{13}C NMR (151 MHz, $CDCl_3$) spectrum of compound 28.

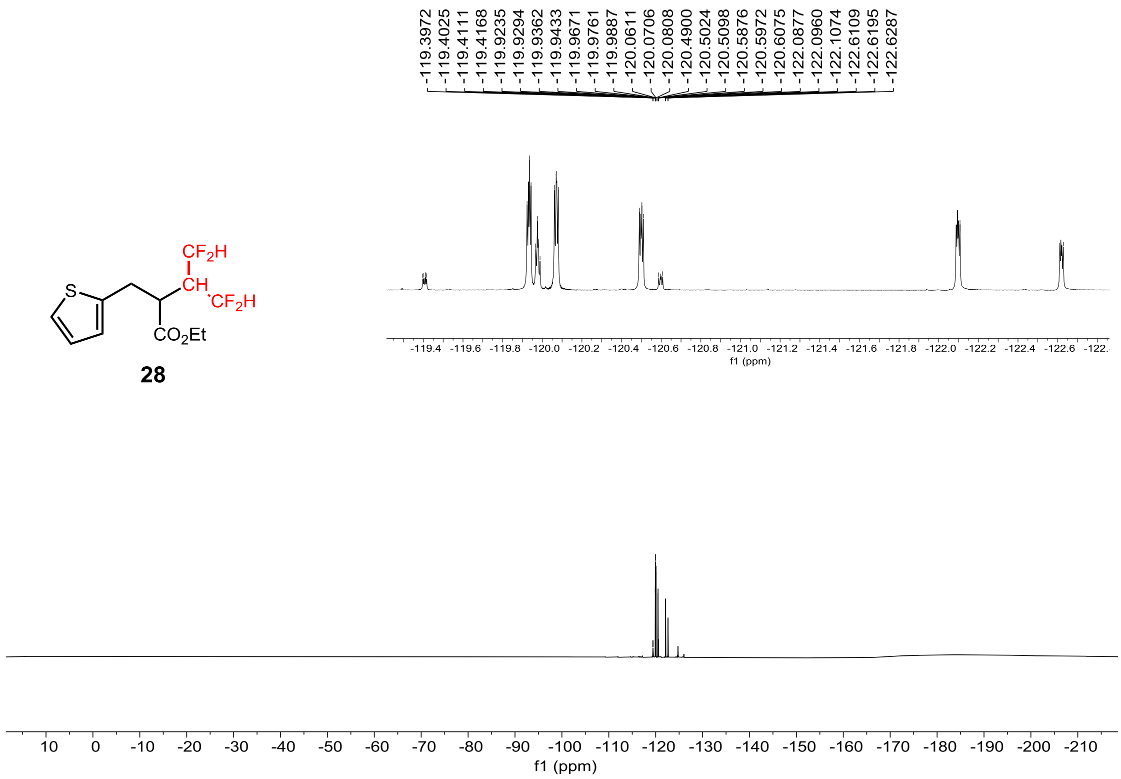

Supplementary Figure 81 ^{19}F NMR (565 MHz, $CDCl_3$) spectrum of compound 28.

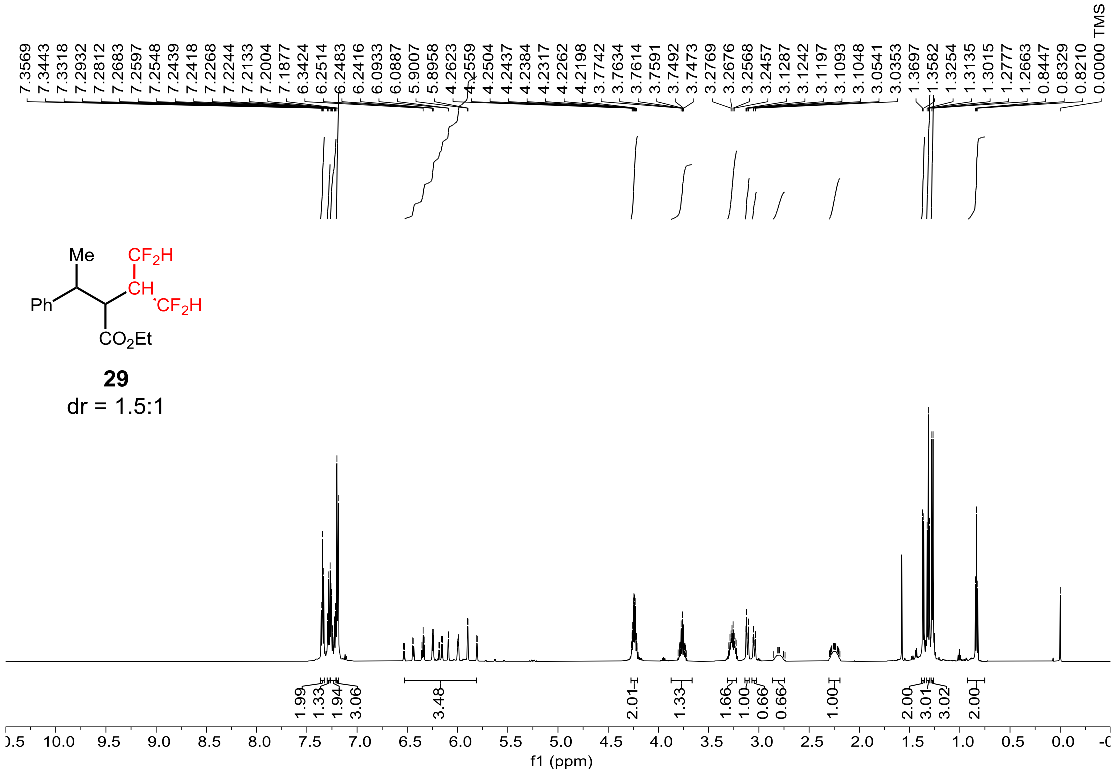

Supplementary Figure 82 ^{1}H NMR (600 MHz, $CDCl_3$) spectrum of compound 29.

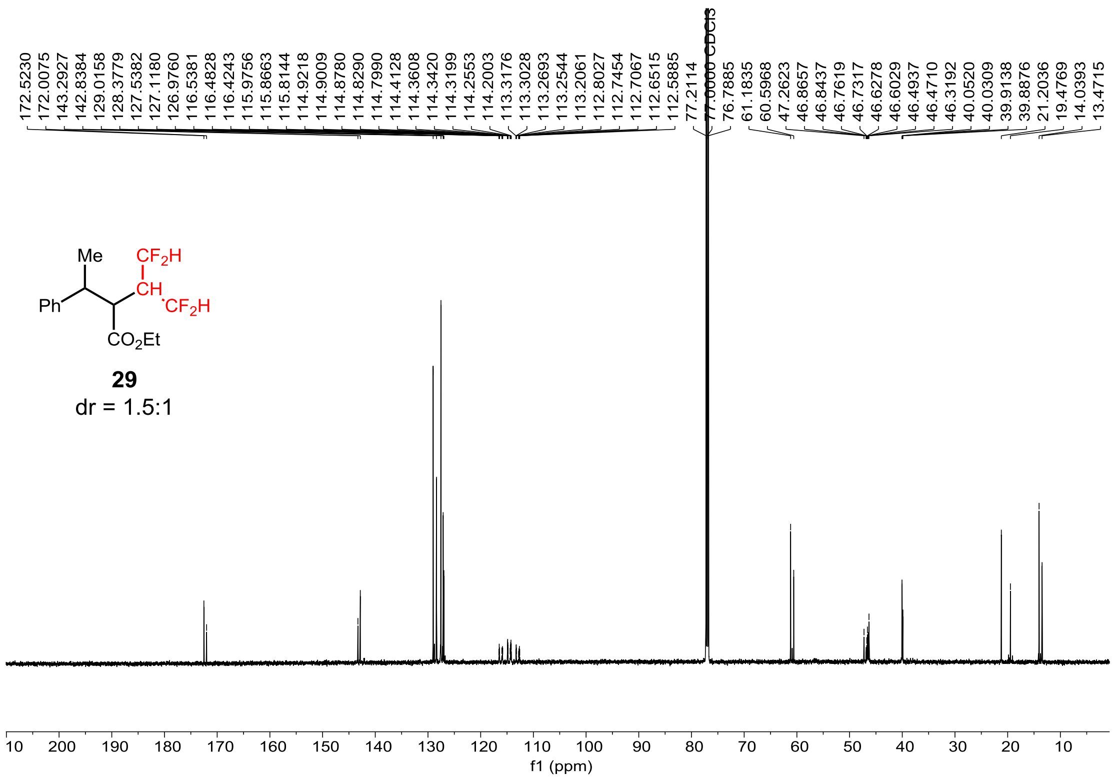

Supplementary Figure 83 ^{13}C NMR (151 MHz, $CDCl_3$) spectrum of compound 29.

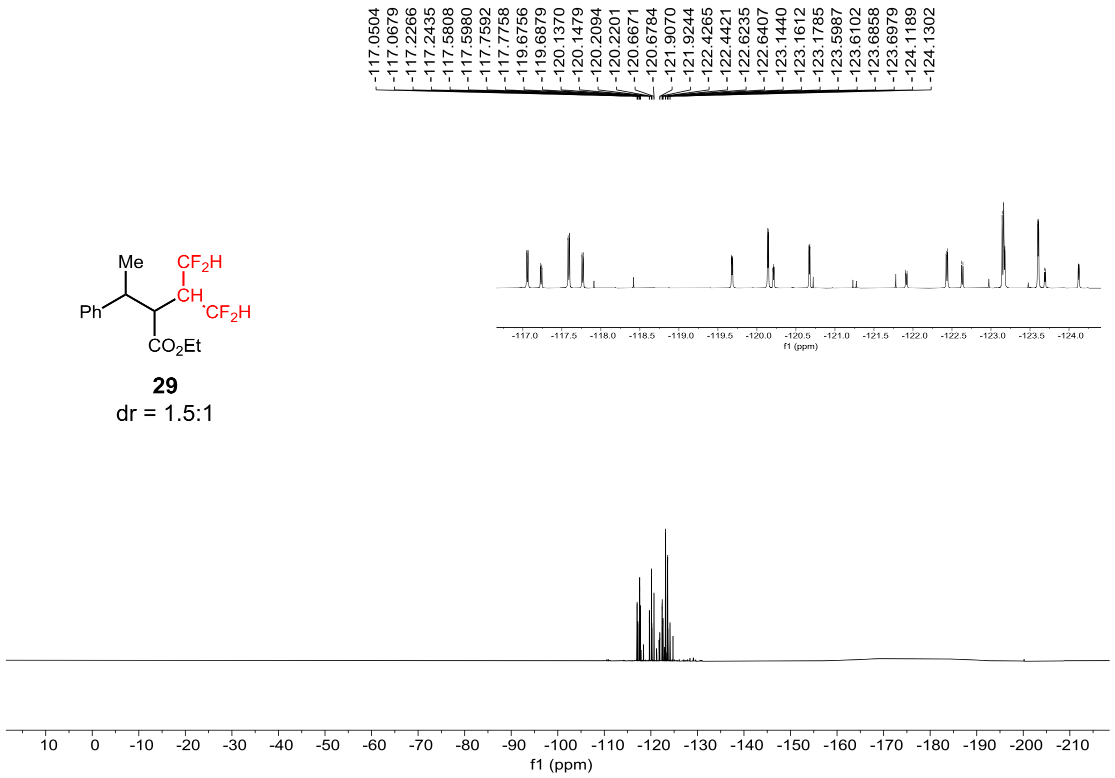

Supplementary Figure 84 ^{19}F NMR (565 MHz, $CDCl_3$) spectrum of compound 29.

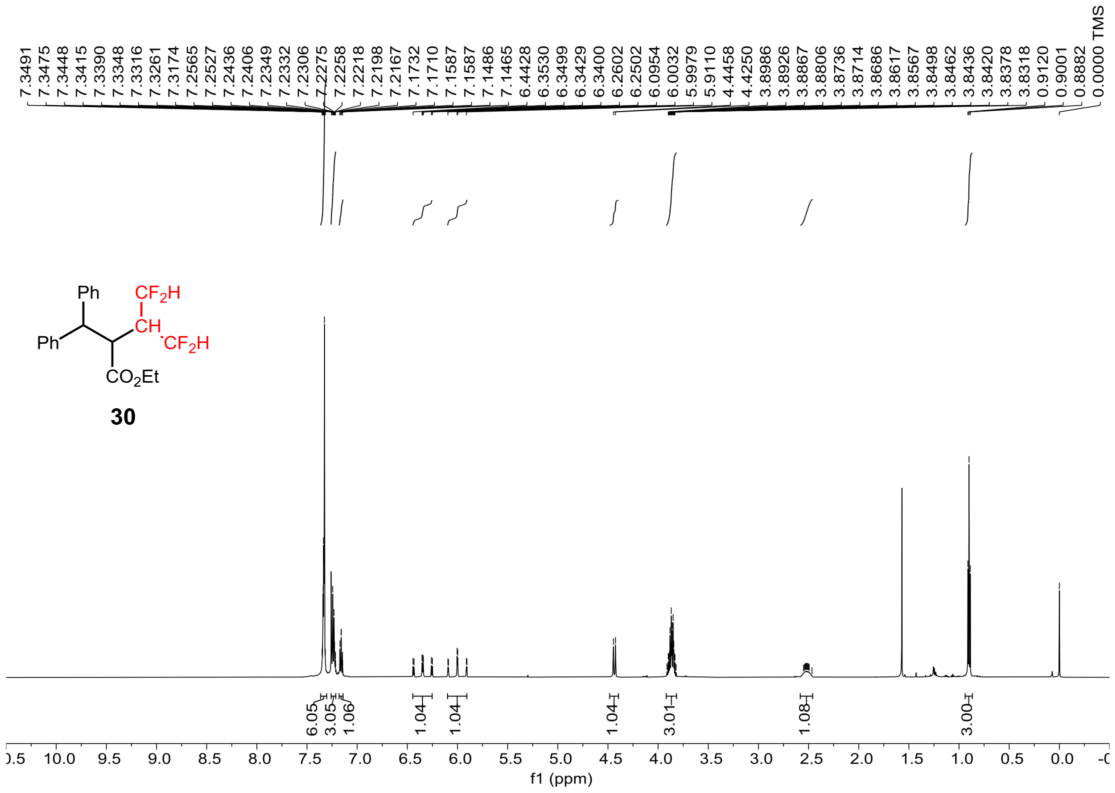

Supplementary Figure 85 ^{1}H NMR (600 MHz, $CDCl_3$) spectrum of compound 30.

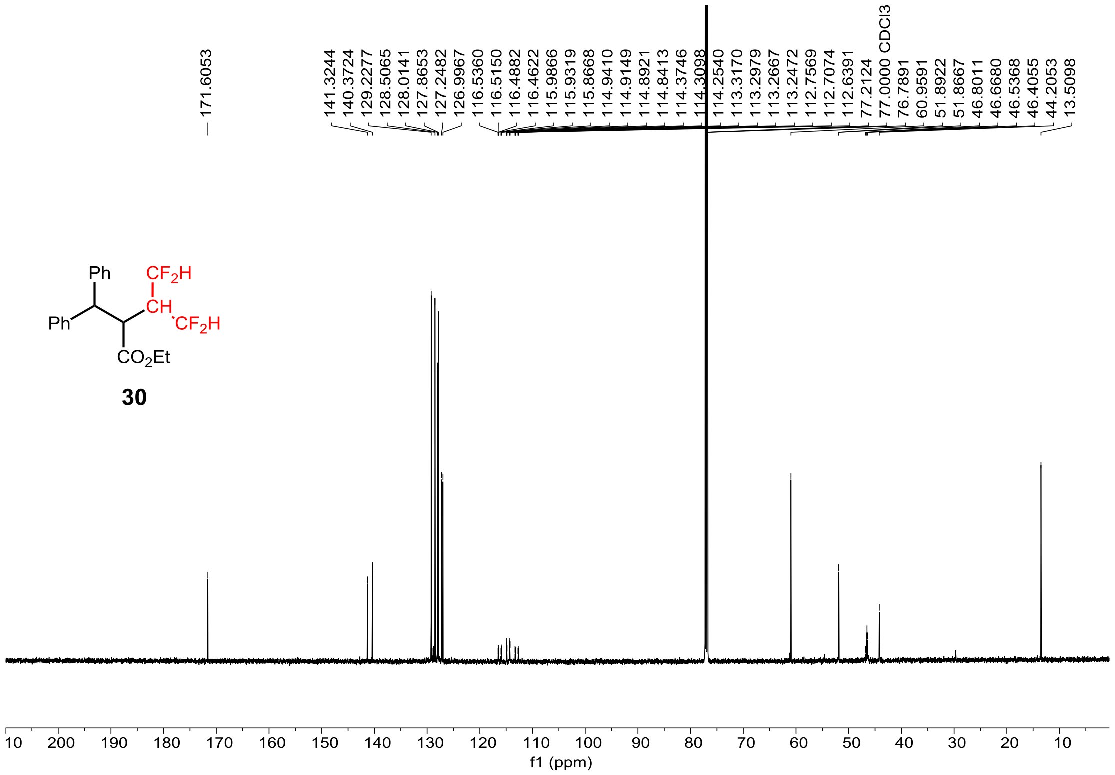

Supplementary Figure 86 ^{13}C NMR (151 MHz, $CDCl_3$) spectrum of compound 30.

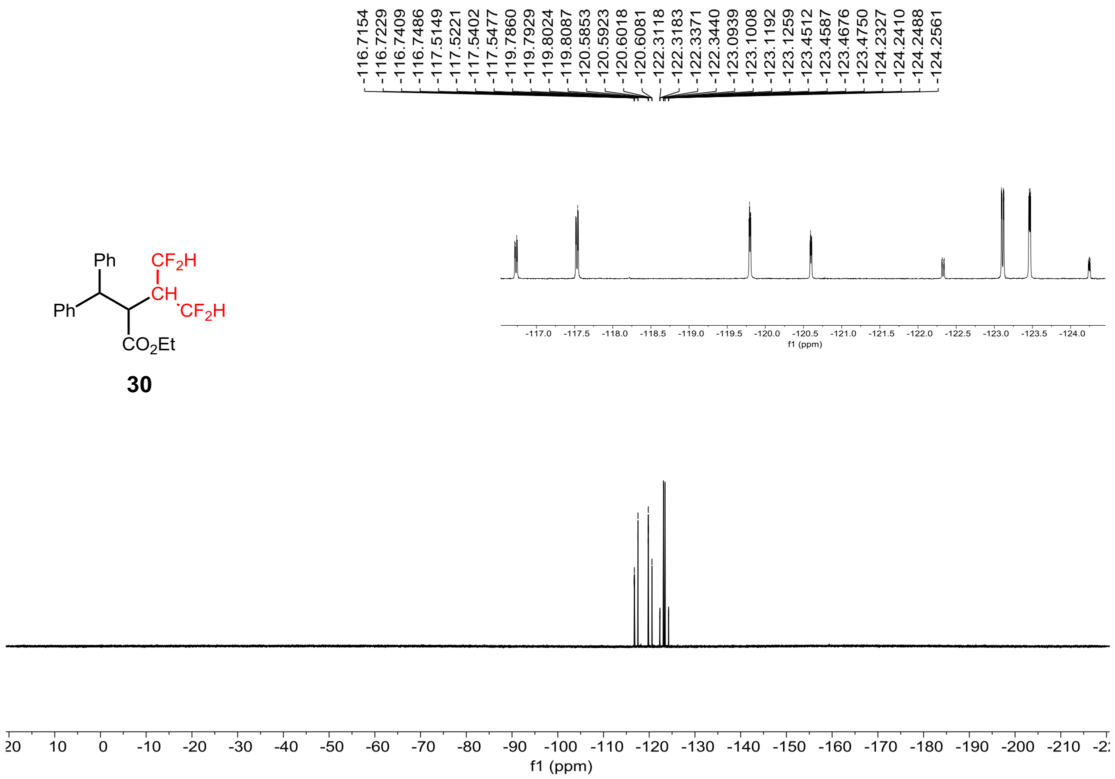

Supplementary Figure 87 ^{19}**F NMR (377 MHz, CDCl**$_3$**) spectrum of compound 30.**

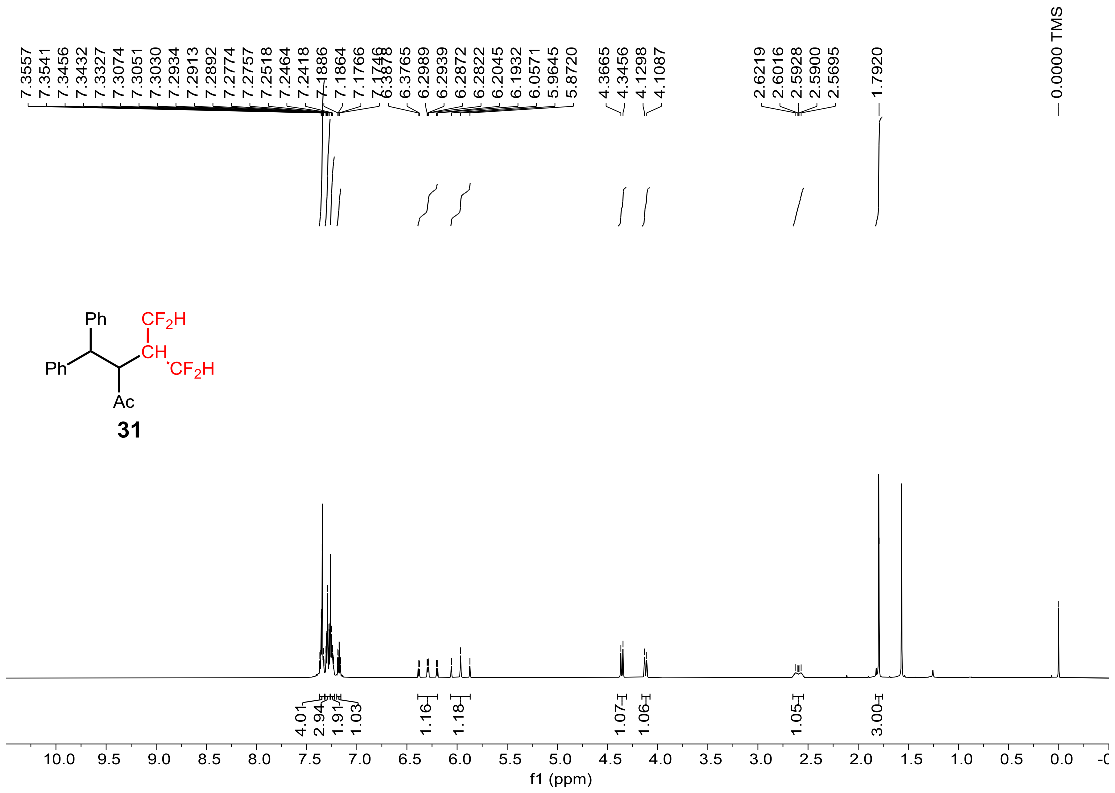

Supplementary Figure 88 ^{1}**H NMR (600 MHz, CDCl**$_3$**) spectrum of compound 31.**

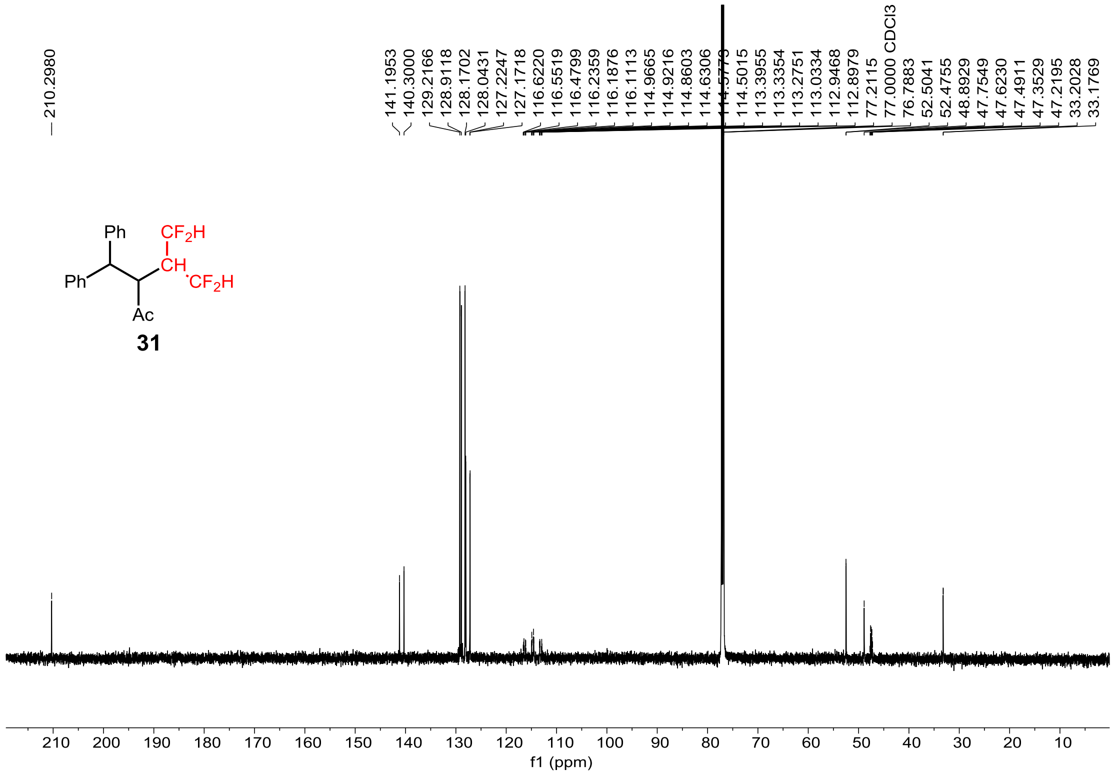

Supplementary Figure 89 ^{13}C NMR (151 MHz, $CDCl_3$) spectrum of compound 31.

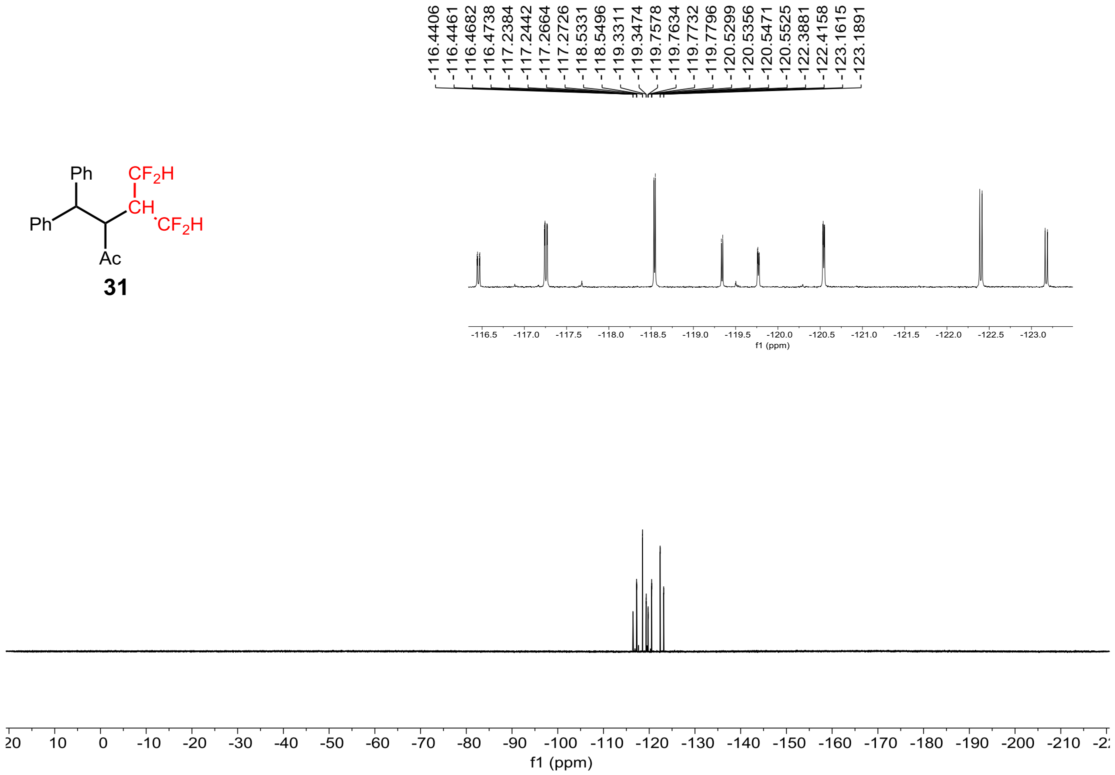

Supplementary Figure 90 ^{19}F NMR (377 MHz, $CDCl_3$) spectrum of compound 31.

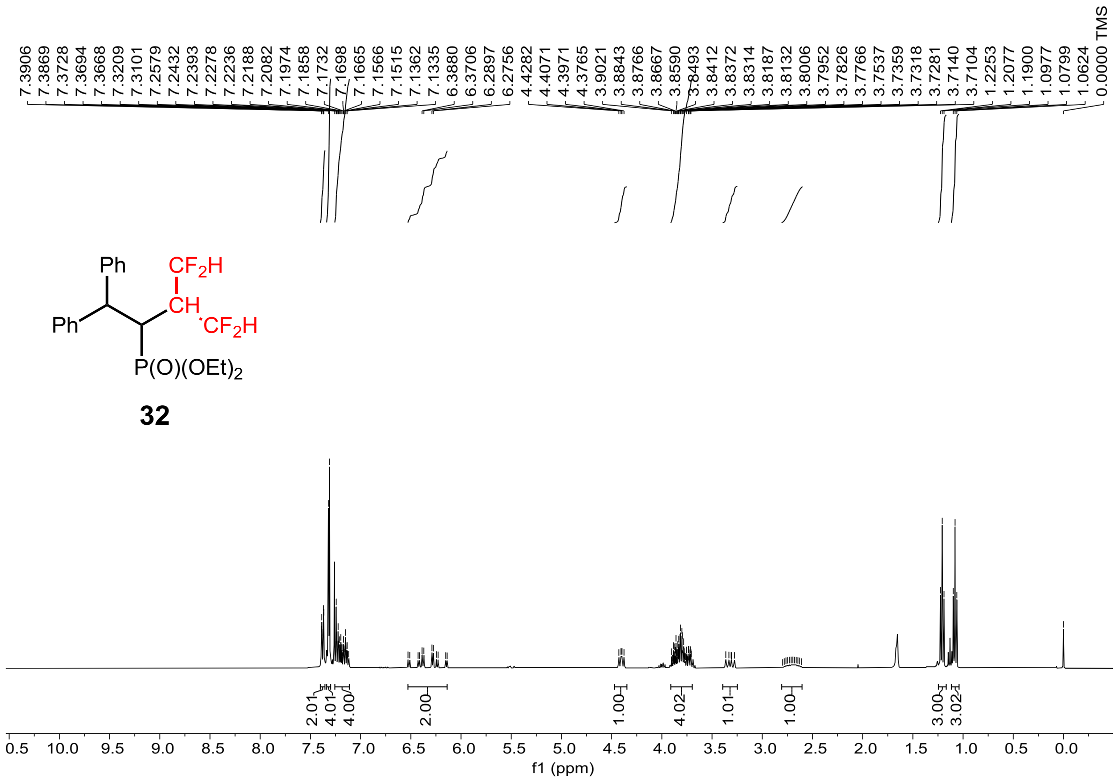

Supplementary Figure 91 ^{1}H NMR (400 MHz, $CDCl_3$) spectrum of compound 32.

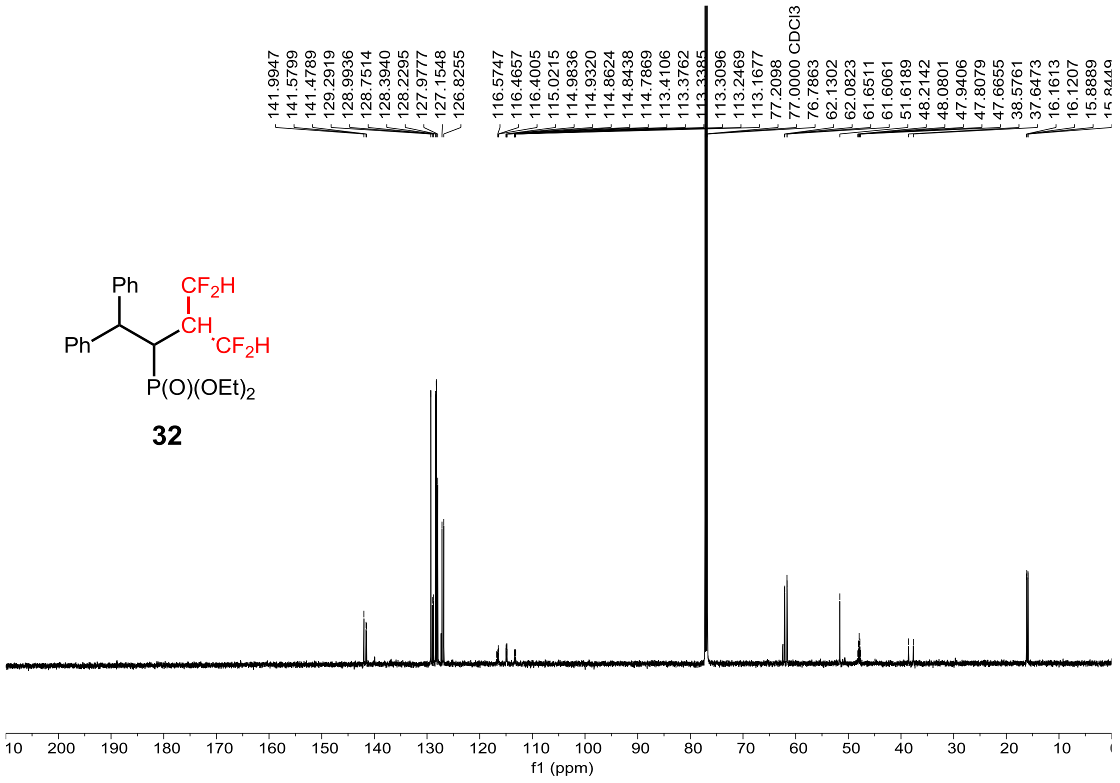

Supplementary Figure 92 ^{13}C NMR (151 MHz, $CDCl_3$) spectrum of compound 32.

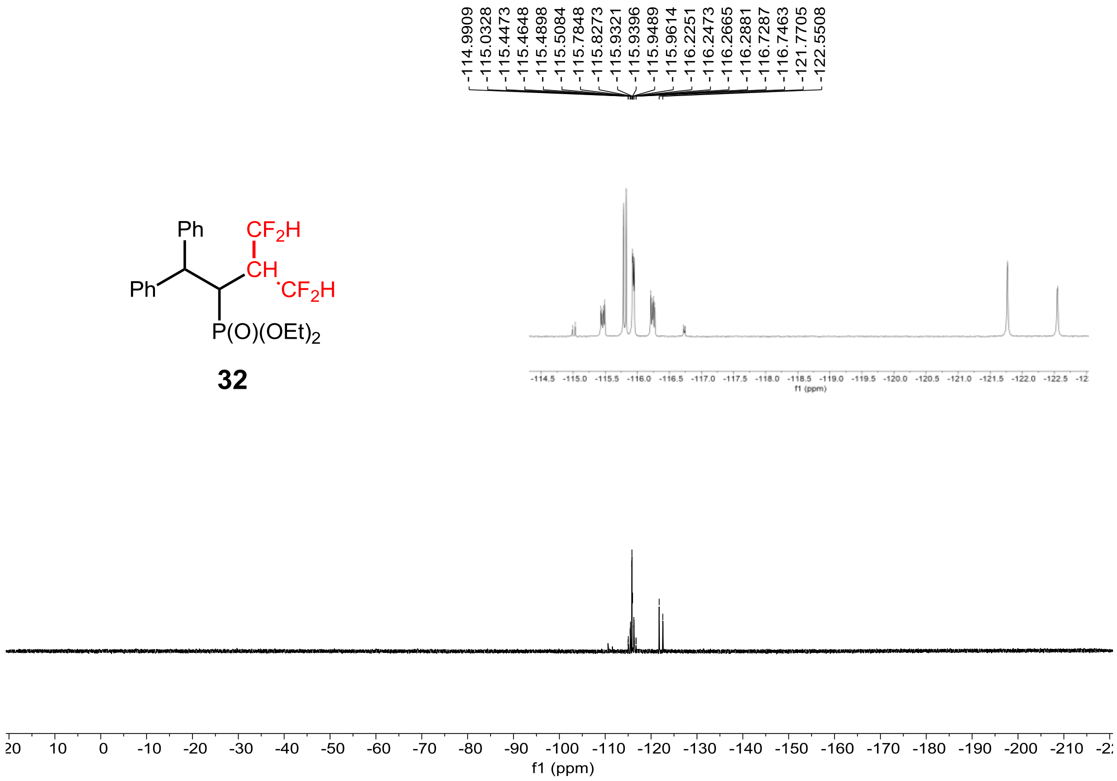

Supplementary Figure 93 ^{19}F NMR (377 MHz, $CDCl_3$) spectrum of compound 32.

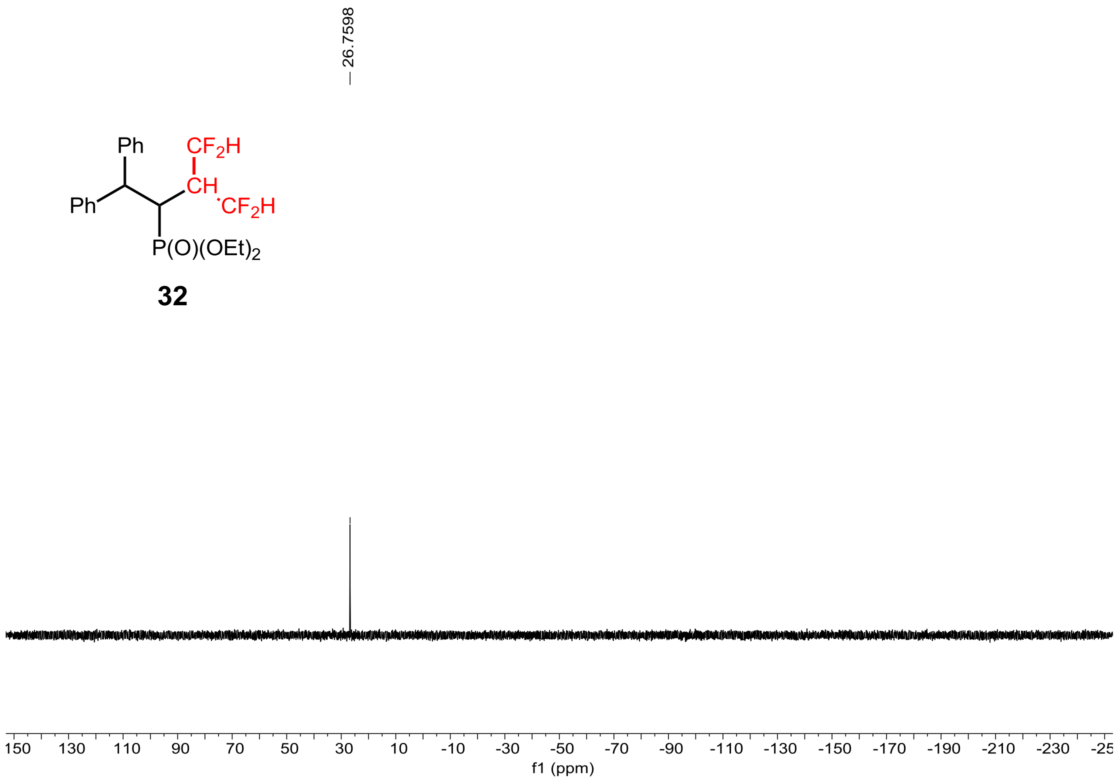

Supplementary Figure 94 ^{31}P NMR (162 MHz, $CDCl_3$) spectrum of compound 32.

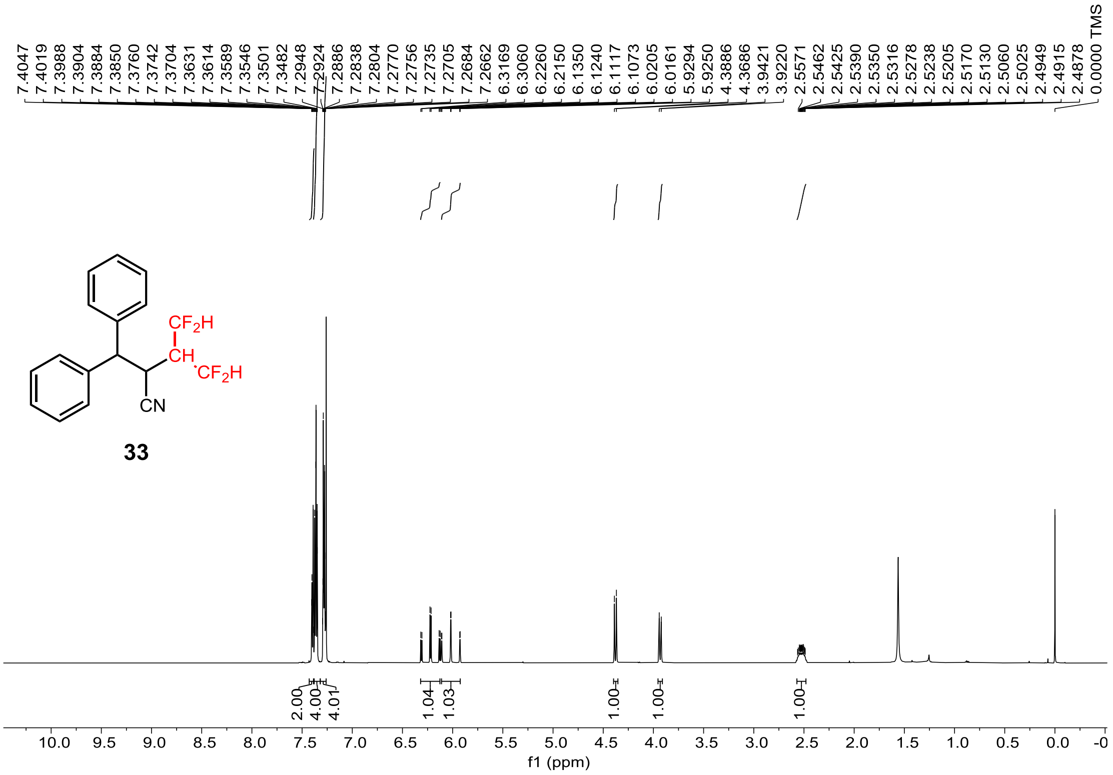

Supplementary Figure 95 ^{1}H NMR (600 MHz, $CDCl_3$) spectrum of compound 33.

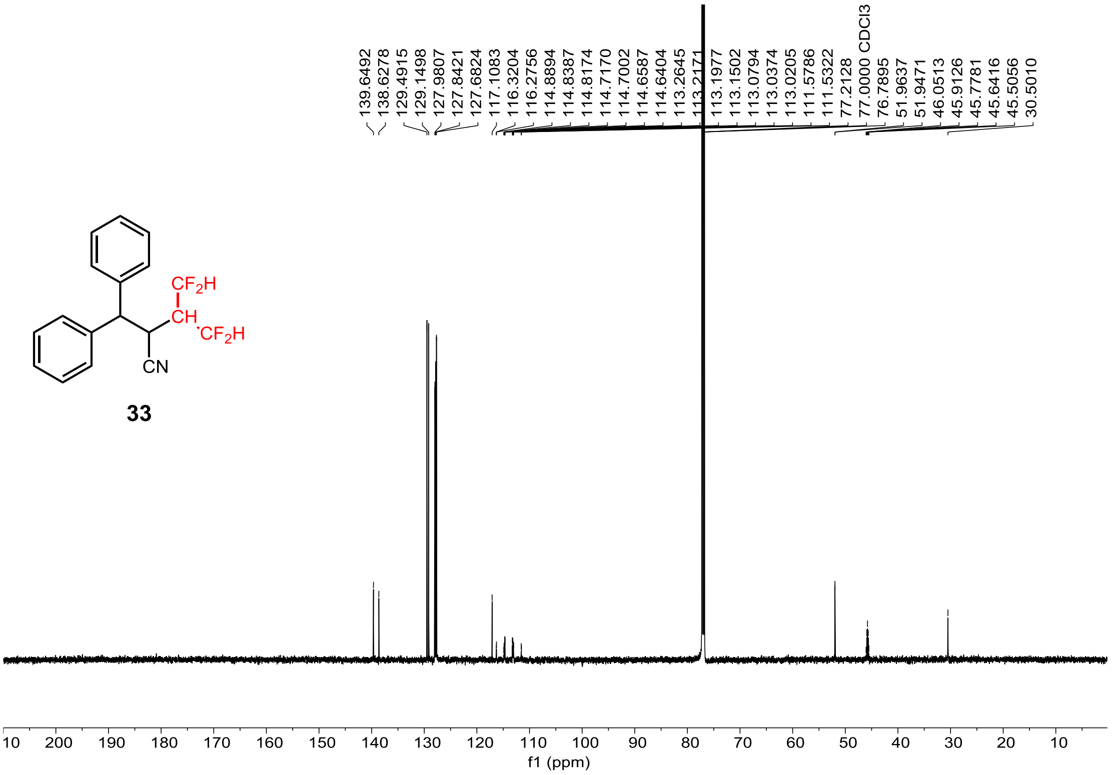

Supplementary Figure 96 ^{13}C NMR (151 MHz, $CDCl_3$) spectrum of compound 33.

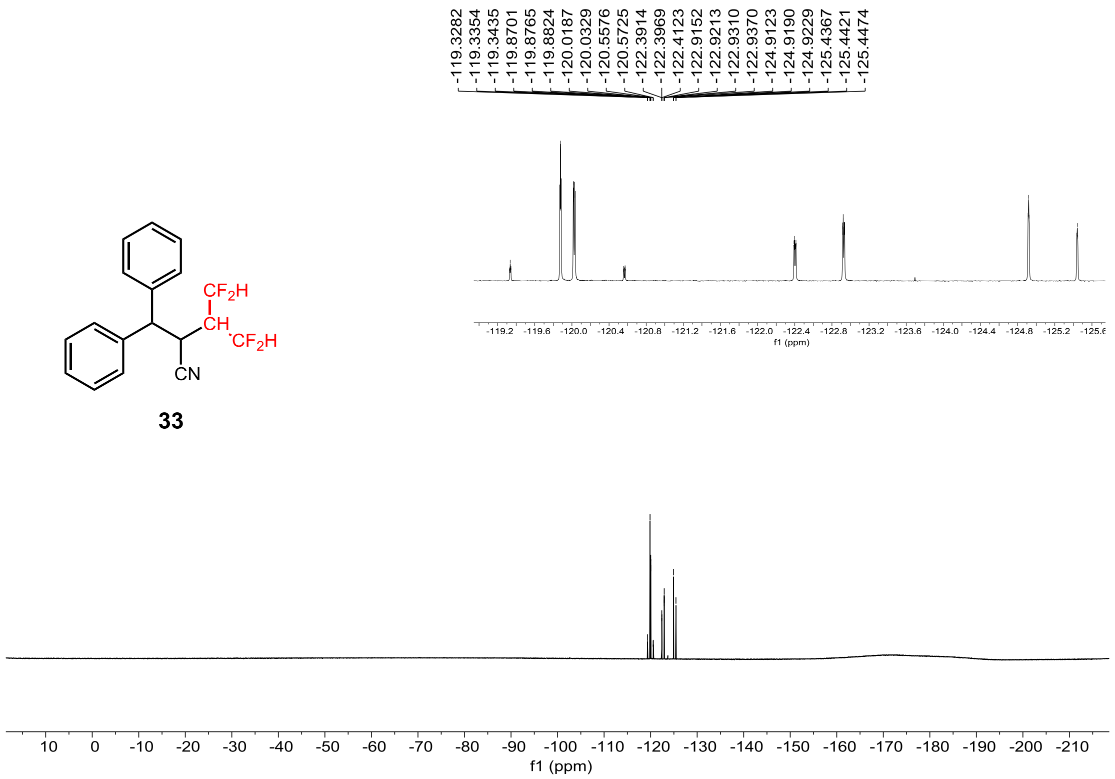

Supplementary Figure 97 ^{19}F NMR (565 MHz, $CDCl_3$) spectrum of compound 33.

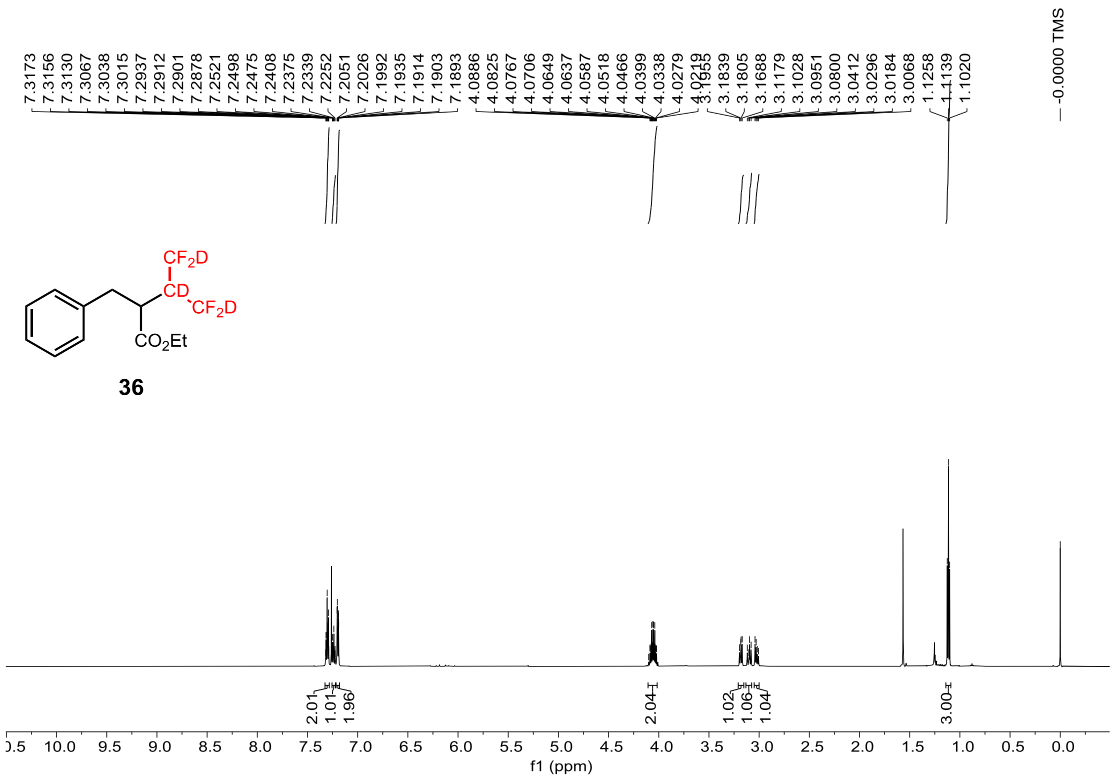

Supplementary Figure 98 ^{1}H NMR (600 MHz, $CDCl_3$) spectrum of compound 36.

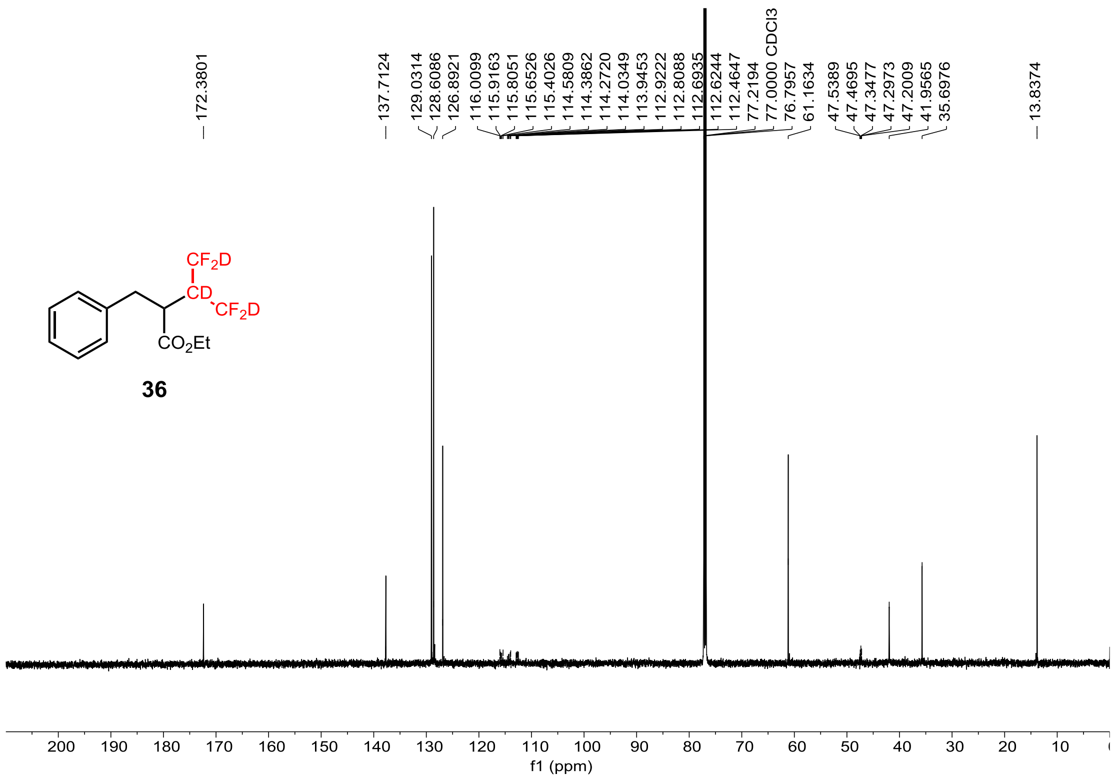

Supplementary Figure 99 ^{13}C NMR (151 MHz, $CDCl_3$) spectrum of compound 36.

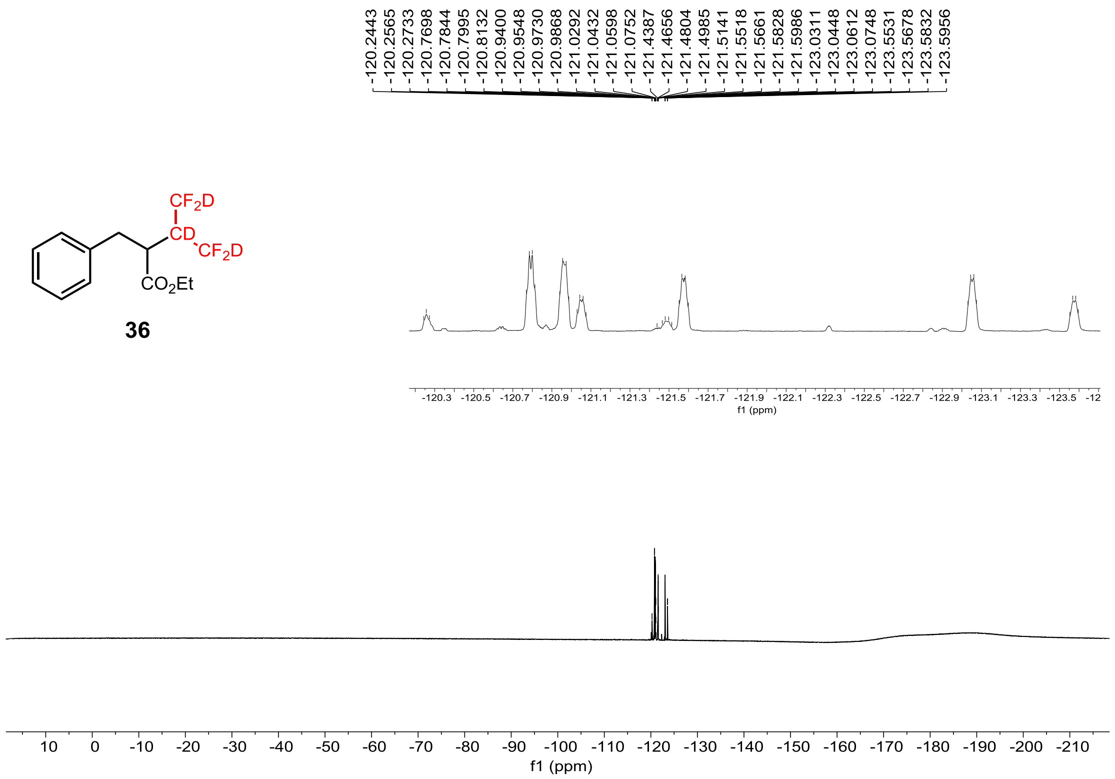

Supplementary Figure 100 ^{19}F NMR (565 MHz, $CDCl_3$) spectrum of compound 36.

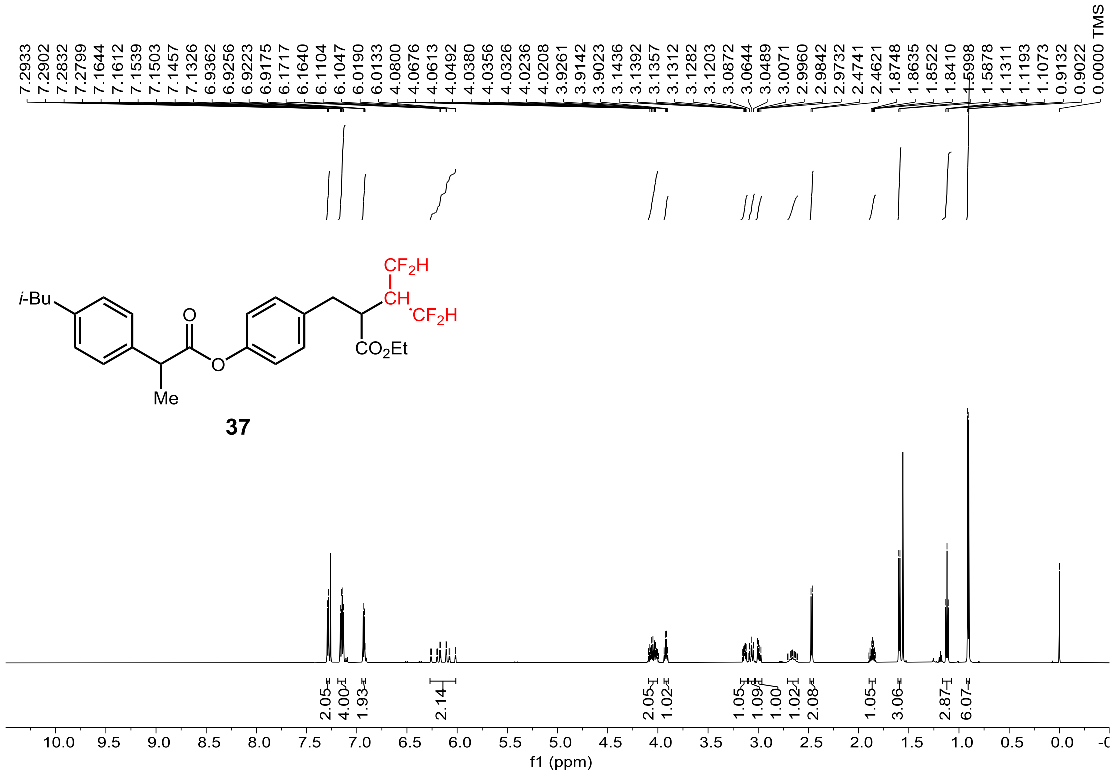

Supplementary Figure 101 ^{1}H NMR (600 MHz, $CDCl_3$) spectrum of compound 37.

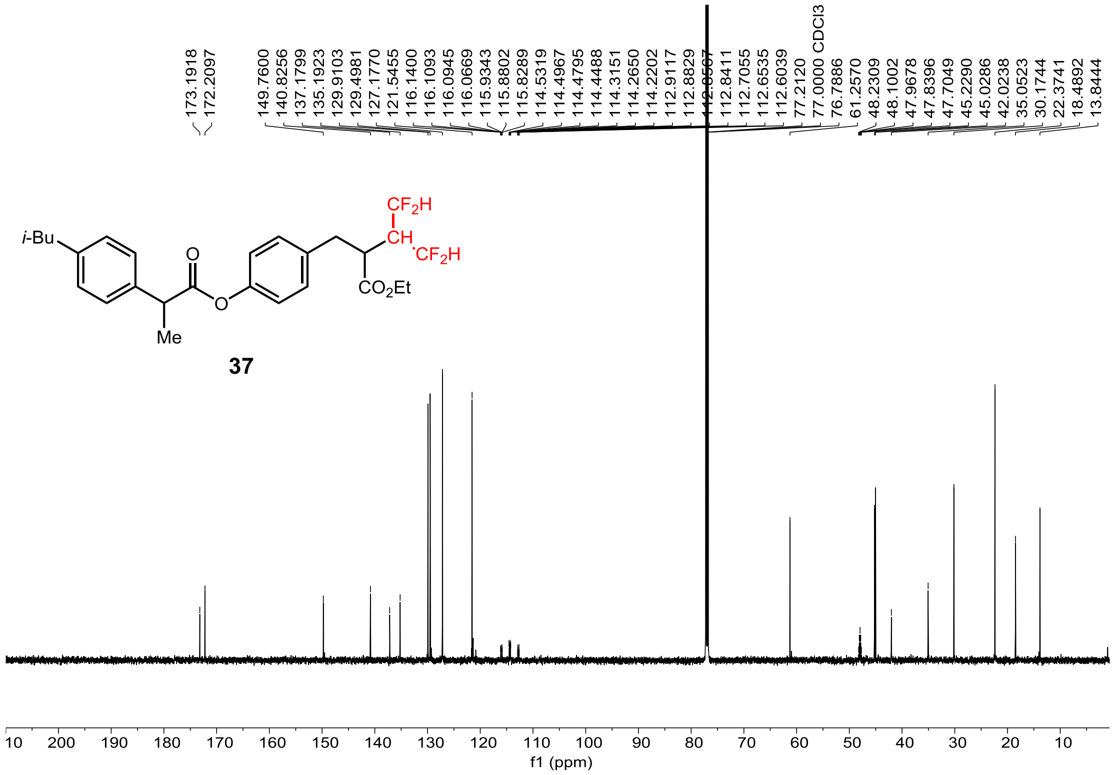

Supplementary Figure 102 ^{13}C NMR (151 MHz, $CDCl_3$) spectrum of compound 37.

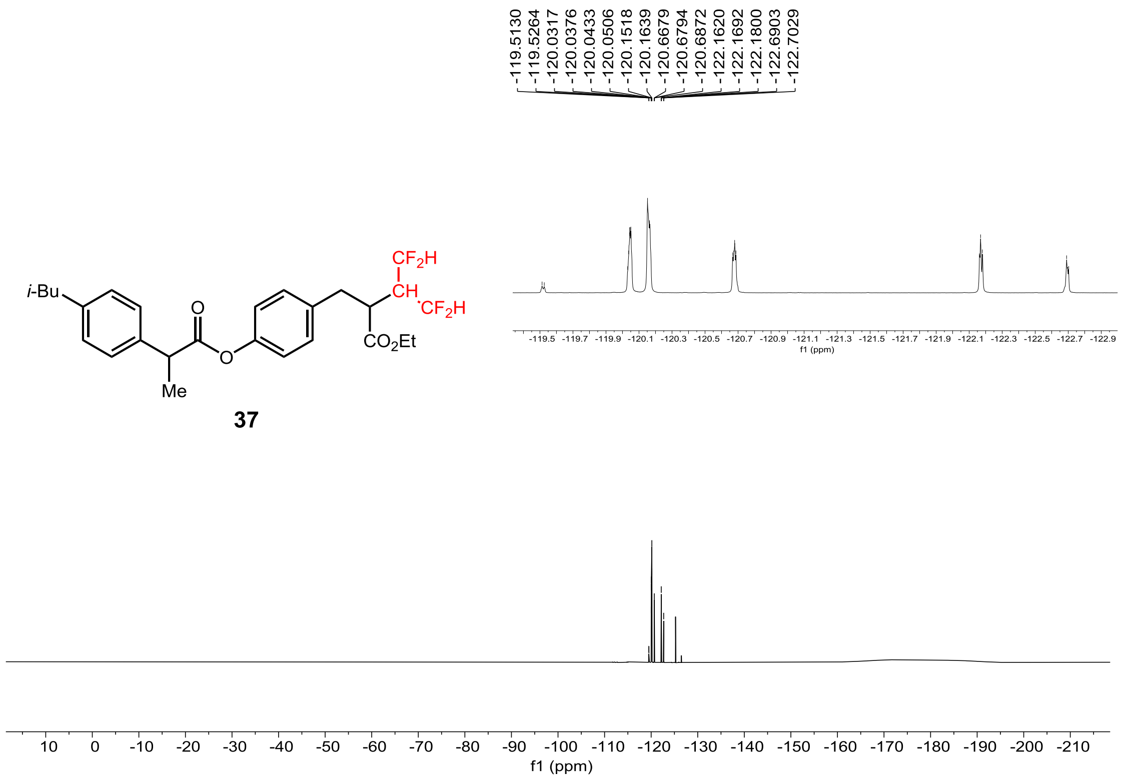

Supplementary Figure 103 ^{19}F NMR (565 MHz, $CDCl_3$) spectrum of compound 37.

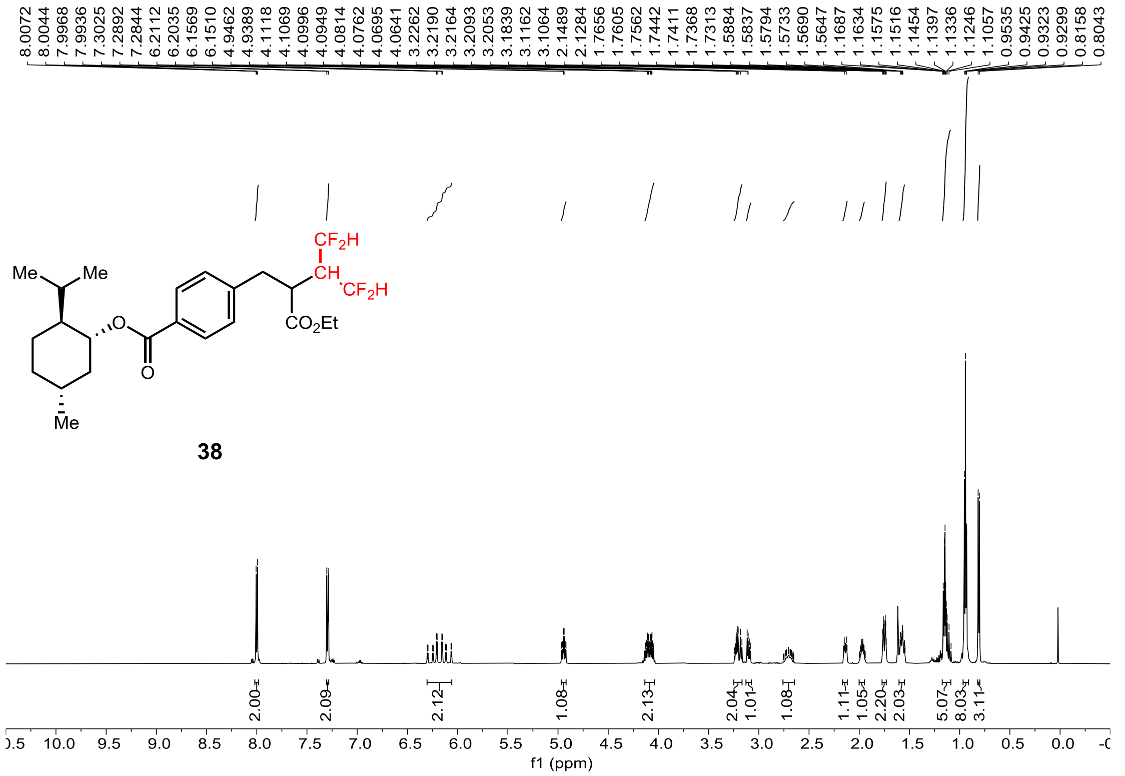

Supplementary Figure 104 ^{1}H NMR (600 MHz, $CDCl_3$) spectrum of compound 38.

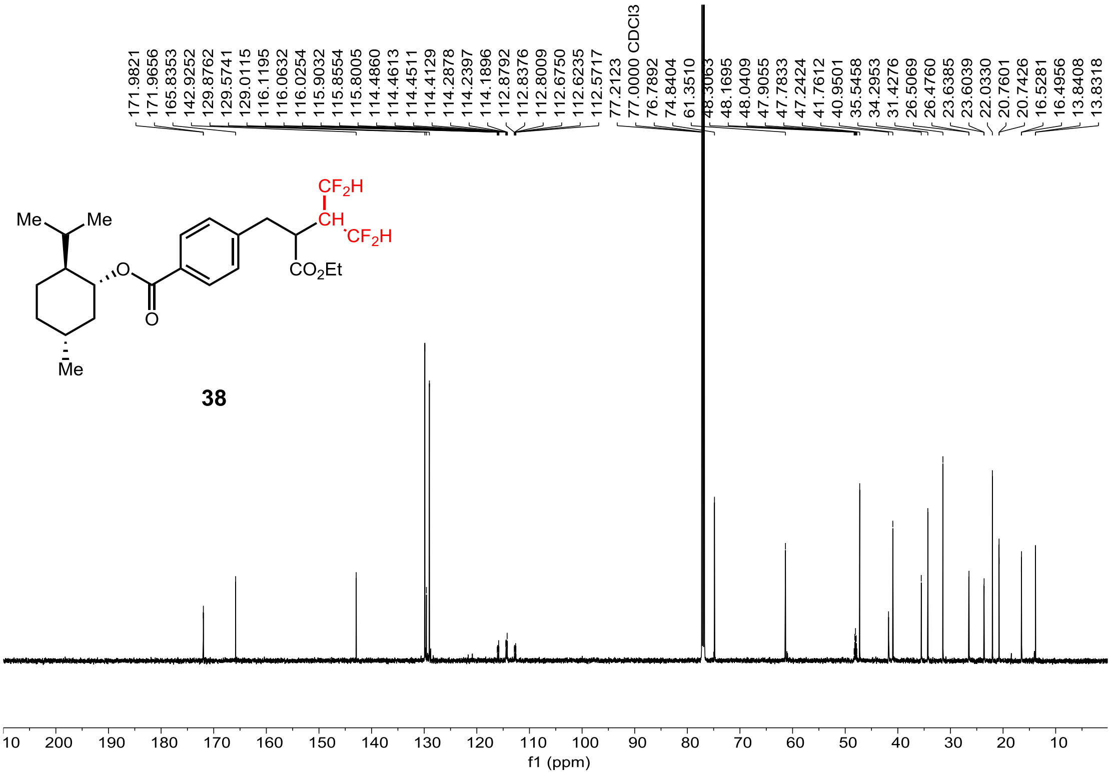

Supplementary Figure 105 ^{13}C NMR (151 MHz, $CDCl_3$) spectrum of compound 38.

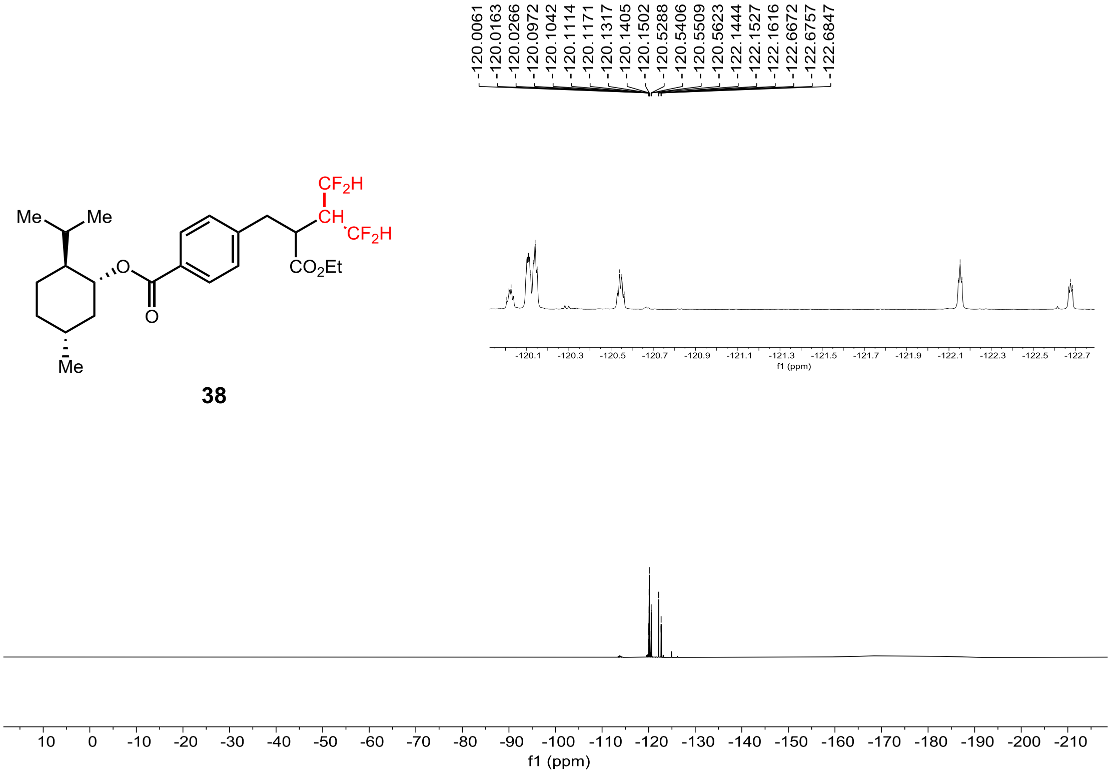

Supplementary Figure 106 ^{19}F NMR (565 MHz, $CDCl_3$) spectrum of compound 38.

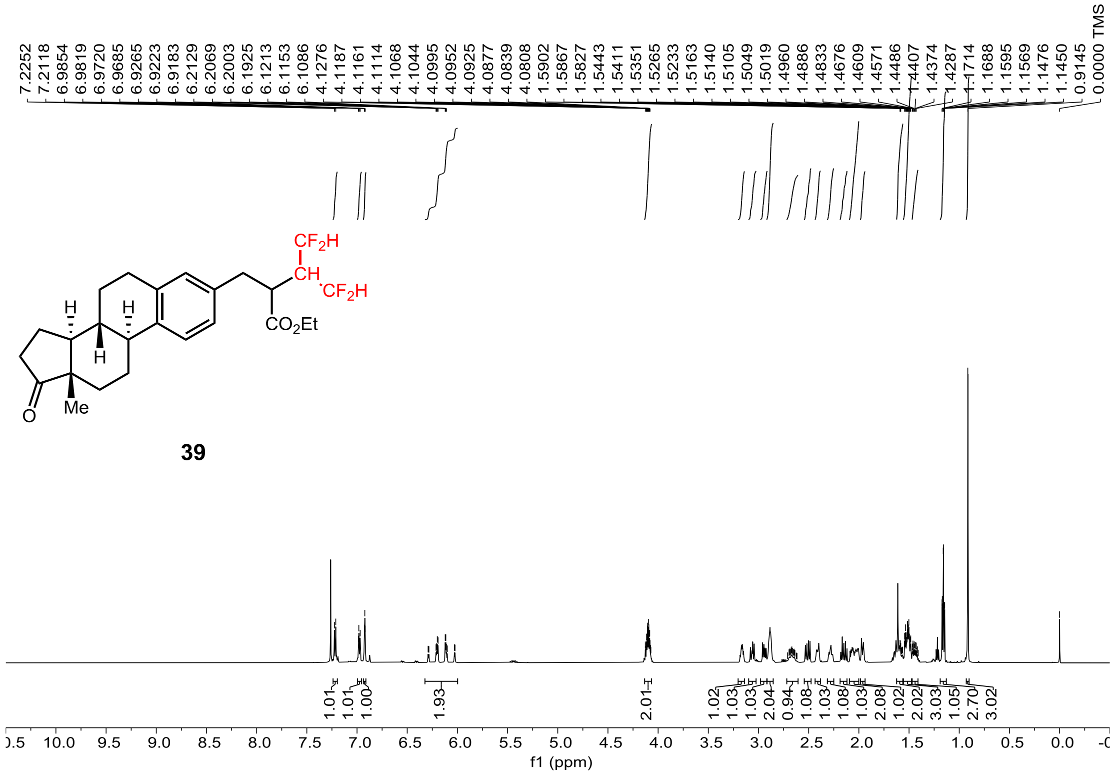

Supplementary Figure 107 ^{1}H NMR (600 MHz, $CDCl_3$) spectrum of compound 39.

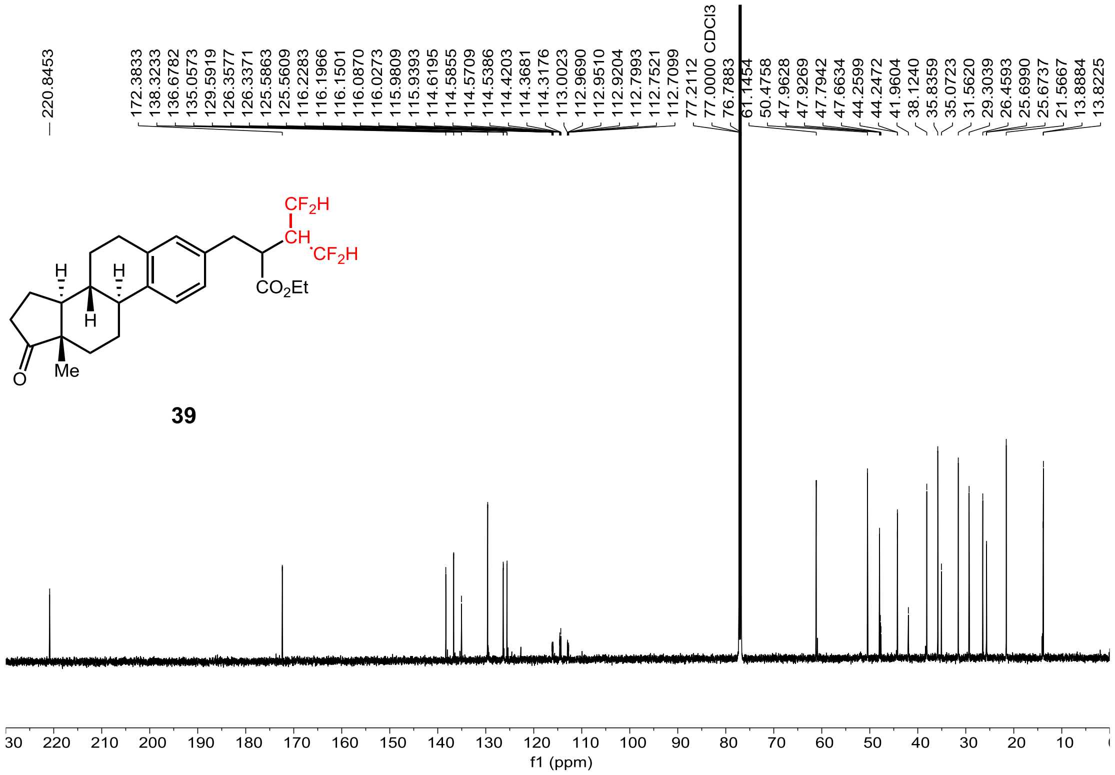

Supplementary Figure 108 ^{13}C NMR (151 MHz, $CDCl_3$) spectrum of compound 39.

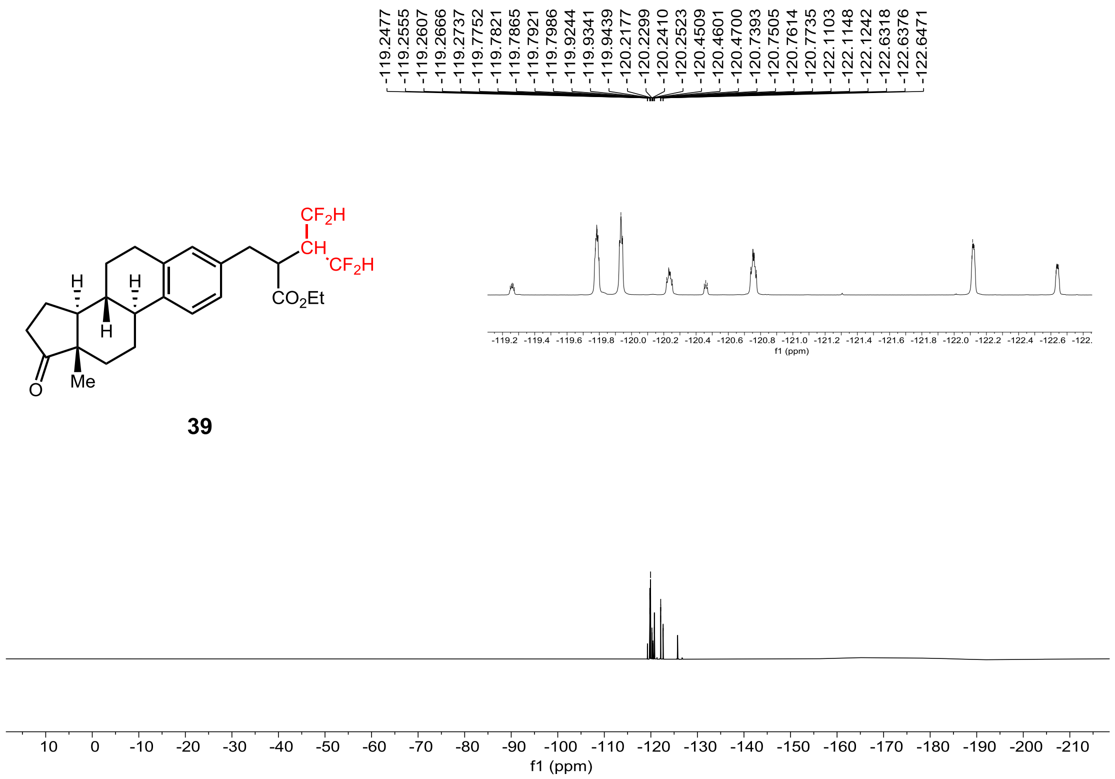

Supplementary Figure 109 ^{19}F NMR (565 MHz, $CDCl_3$) spectrum of compound 39.

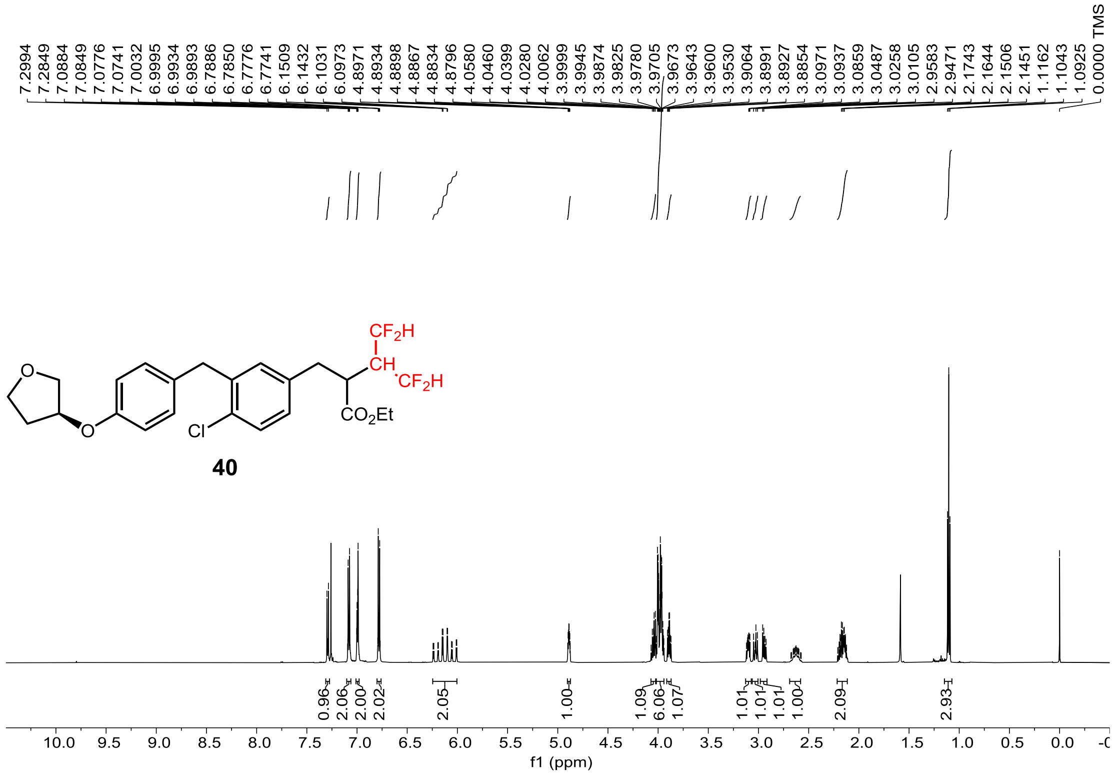

Supplementary Figure 110 ^{1}H NMR (600 MHz, $CDCl_3$) spectrum of compound 40.

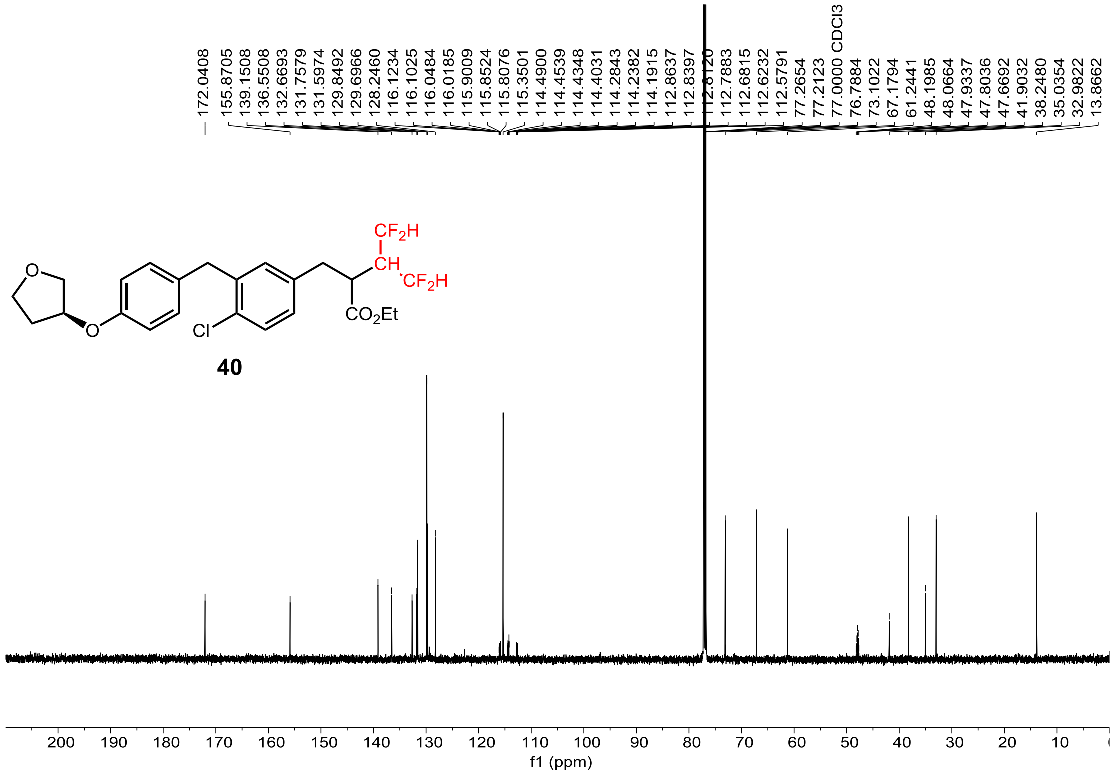

Supplementary Figure 111 ^{13}C NMR (151 MHz, $CDCl_3$) spectrum of compound 40.

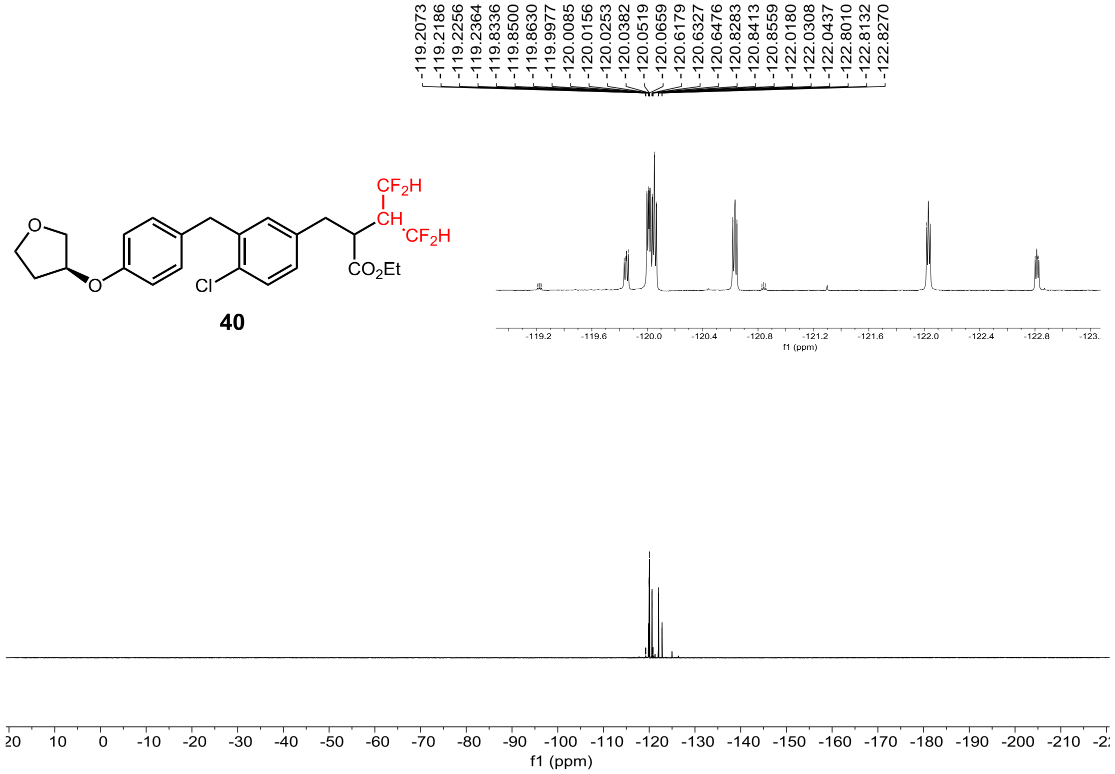

Supplementary Figure 112 ^{19}F NMR (565 MHz, $CDCl_3$) spectrum of compound 40.

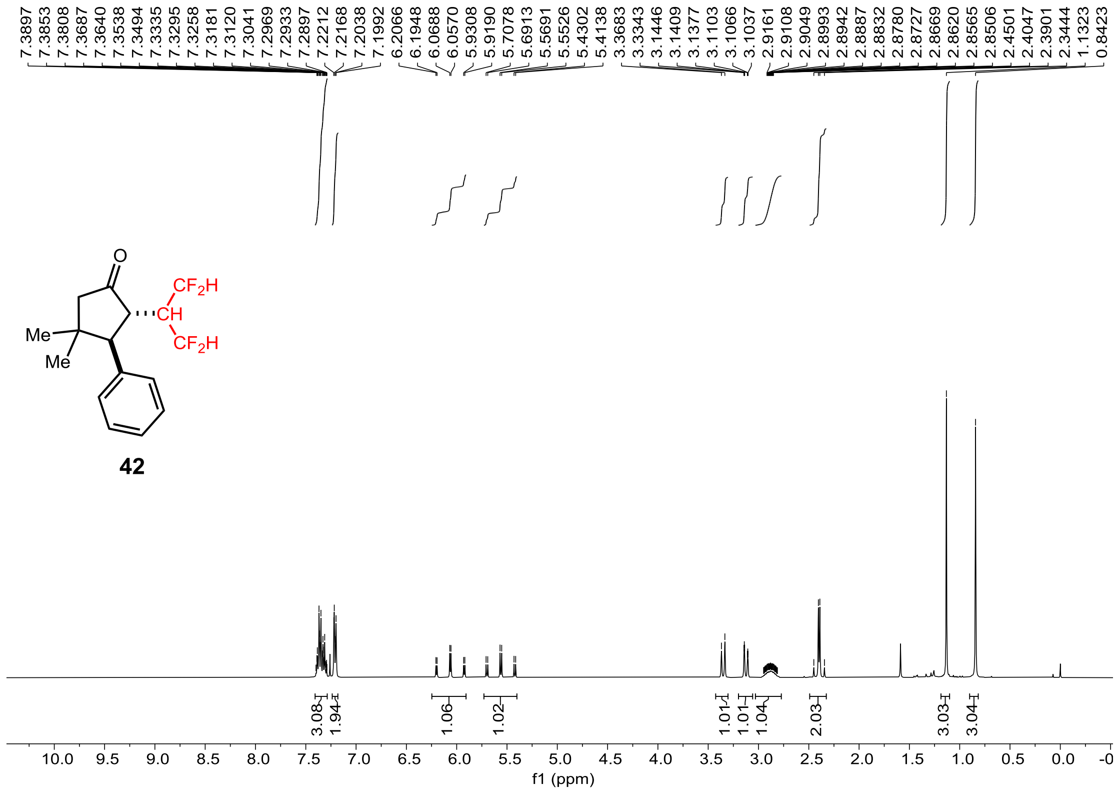

Supplementary Figure 113 ^{1}H NMR (400 MHz, $CDCl_3$) spectrum of compound 42.

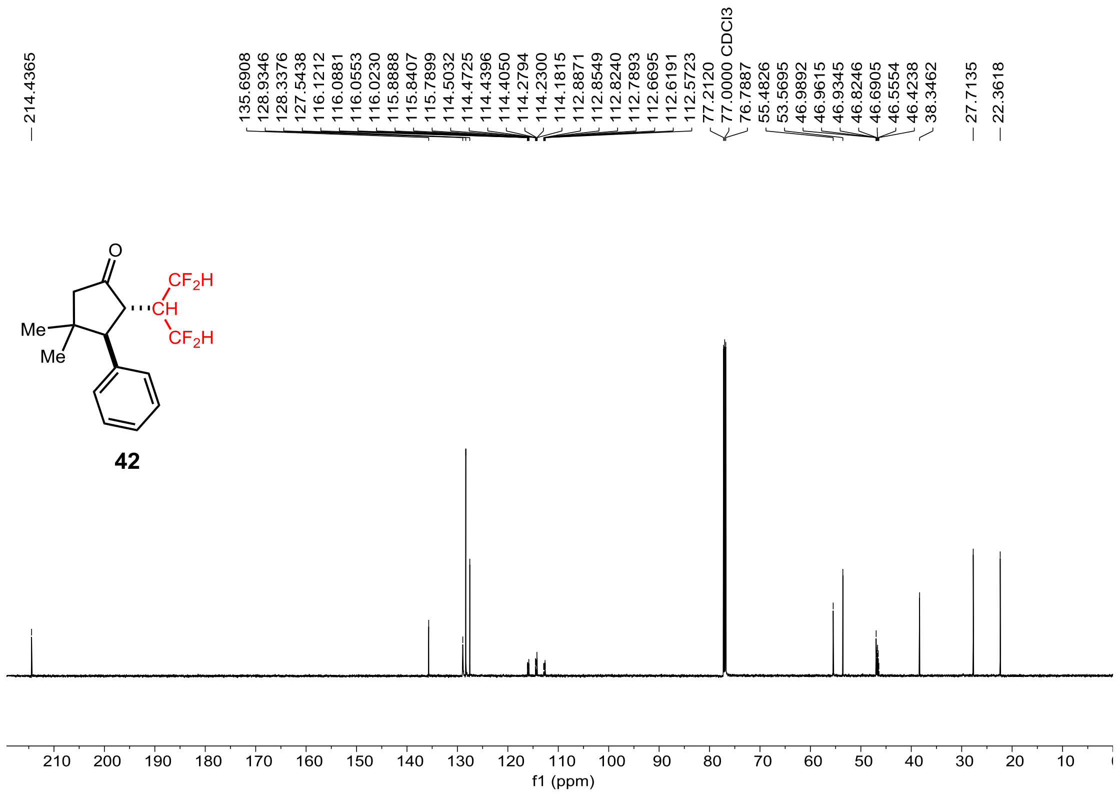

Supplementary Figure 114 ^{13}C NMR (151 MHz, $CDCl_3$) spectrum of compound 42.

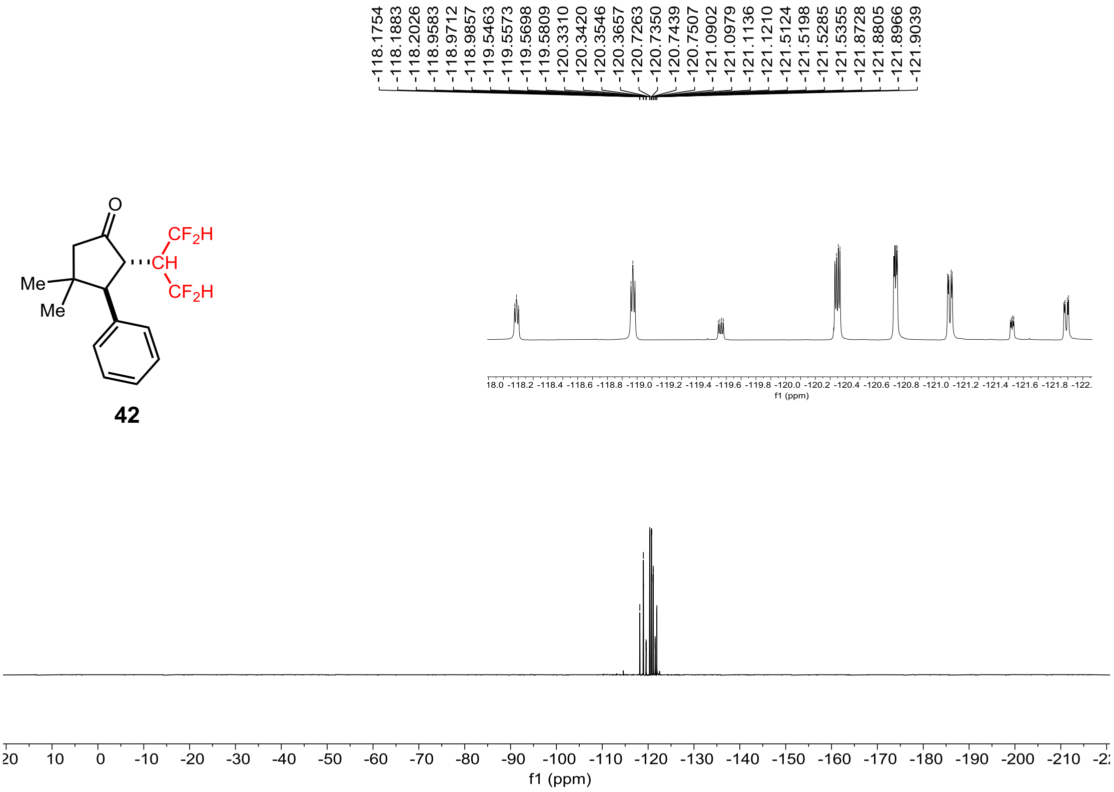

Supplementary Figure 115 ^{19}F NMR (377 MHz, CDCl$_3$) spectrum of compound 42.

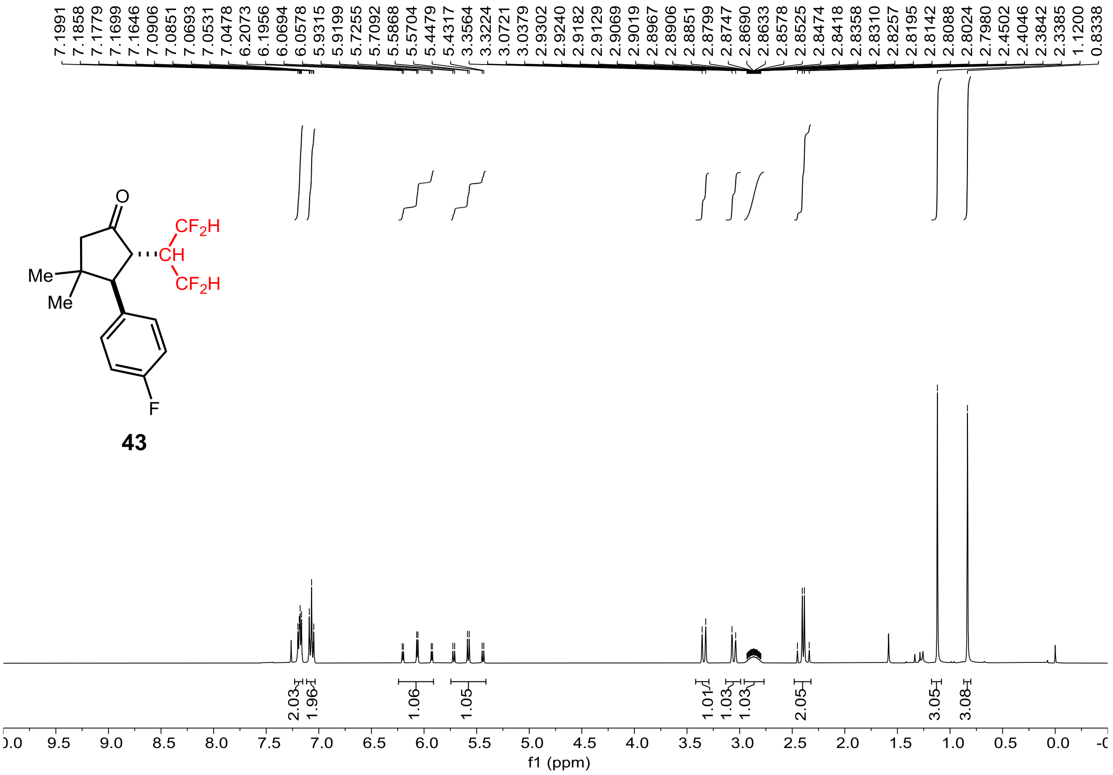

Supplementary Figure 116 ^{1}H NMR (400 MHz, CDCl$_3$) spectrum of compound 43.

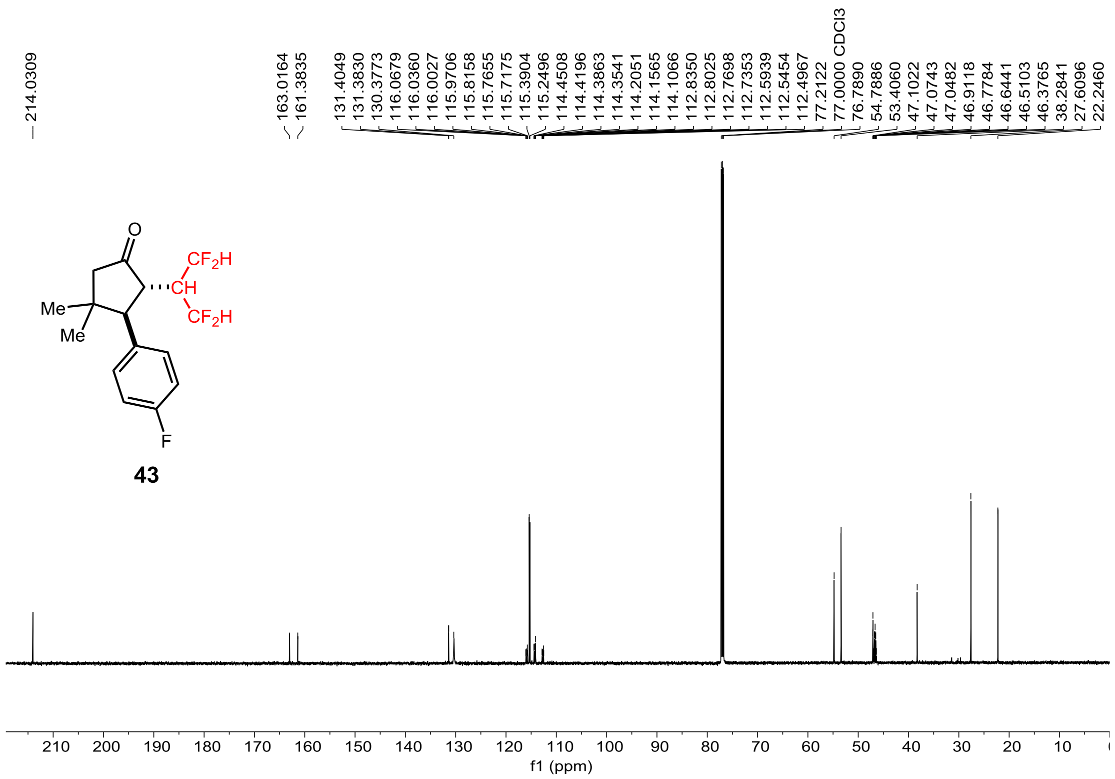

Supplementary Figure 117 ^{13}C NMR (151 MHz, $CDCl_3$) spectrum of compound 43.

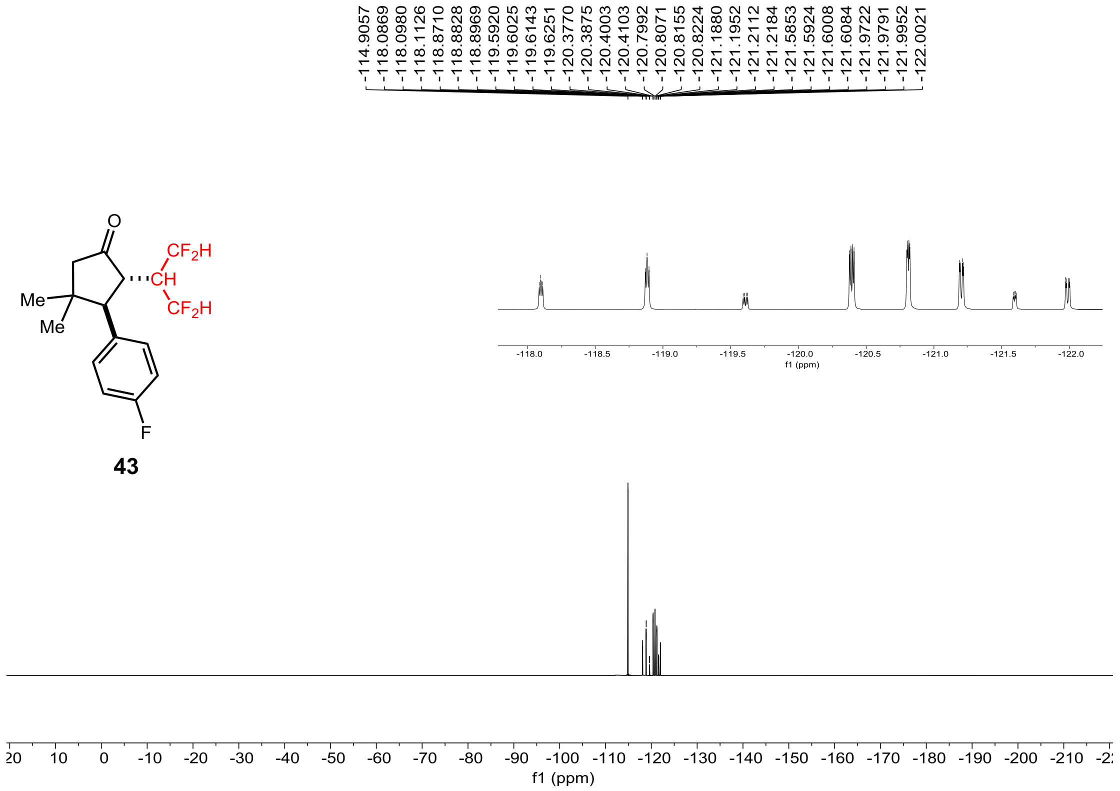

Supplementary Figure 118 ^{19}F NMR (377 MHz, $CDCl_3$) spectrum of compound 43.

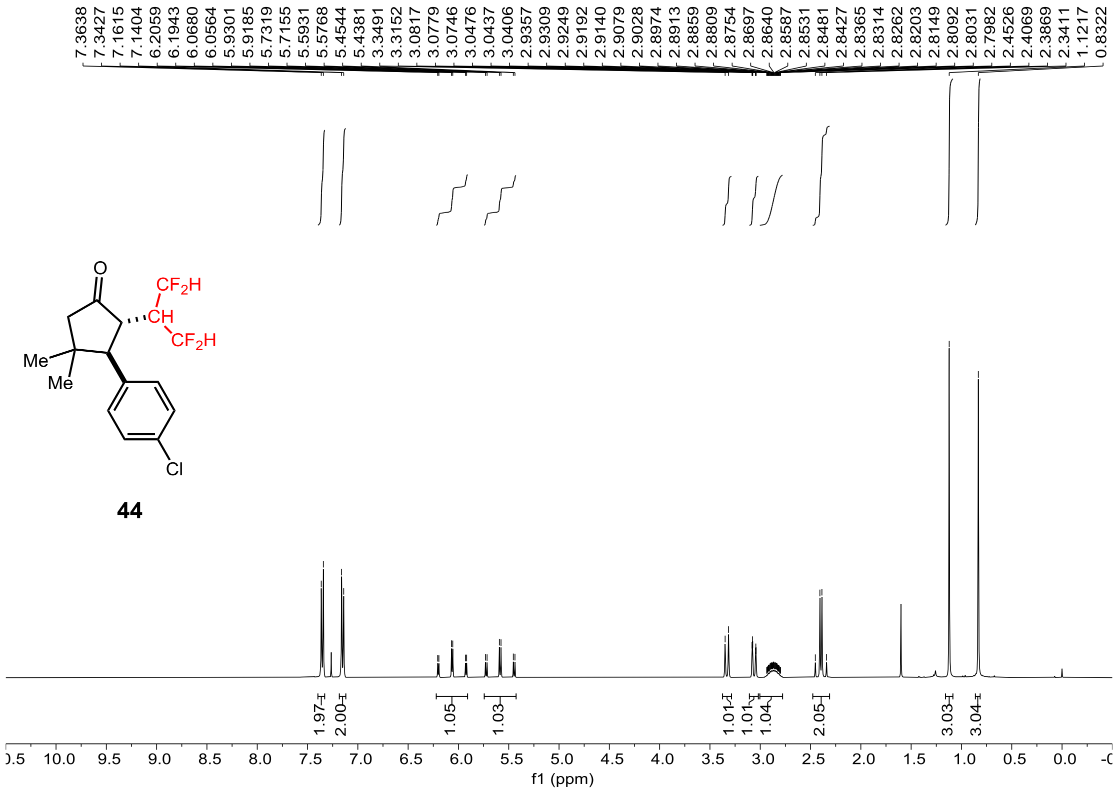

Supplementary Figure 119 ^{1}H NMR (400 MHz, $CDCl_3$) spectrum of compound 44.

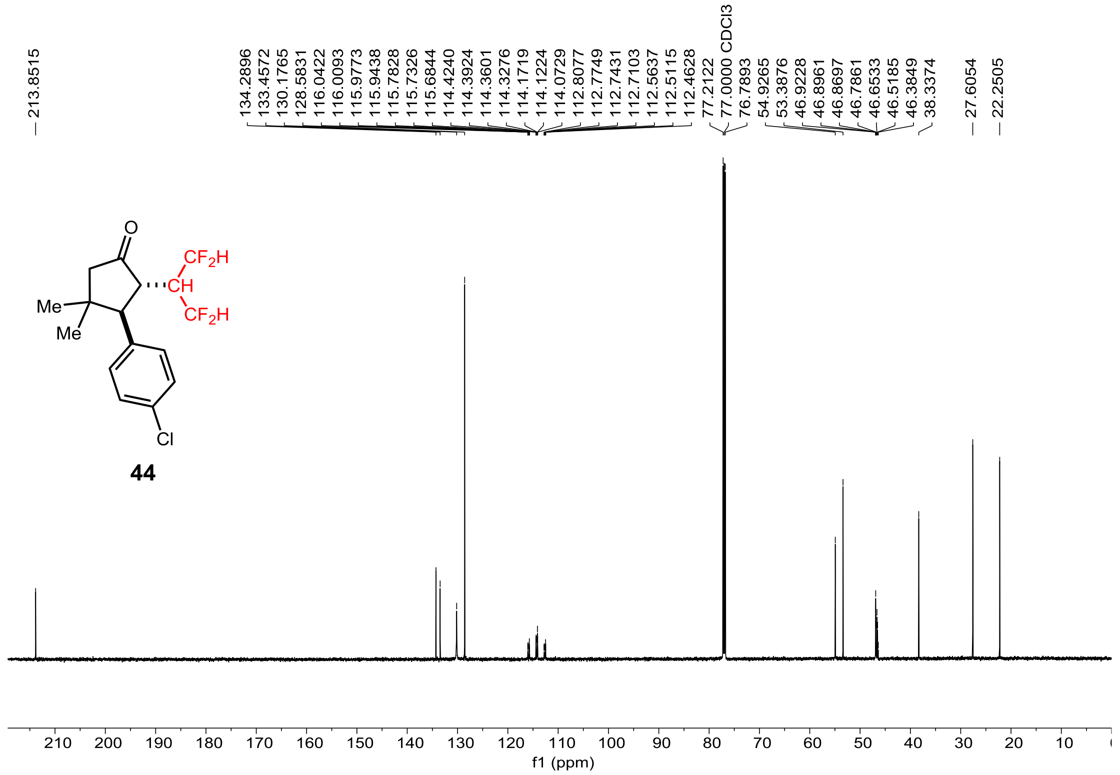

Supplementary Figure 120 ^{13}C NMR (151 MHz, $CDCl_3$) spectrum of compound 44.

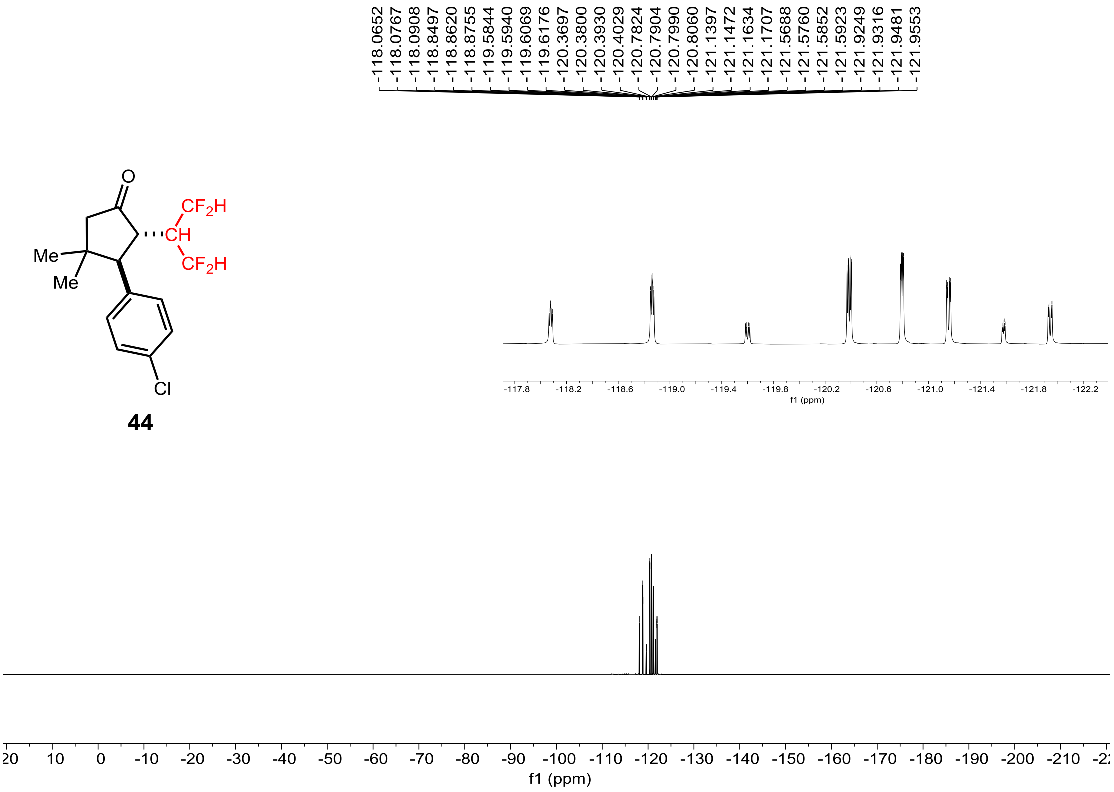

Supplementary Figure 121 ^{19}F NMR (377 MHz, CDCl$_3$) spectrum of compound 44.

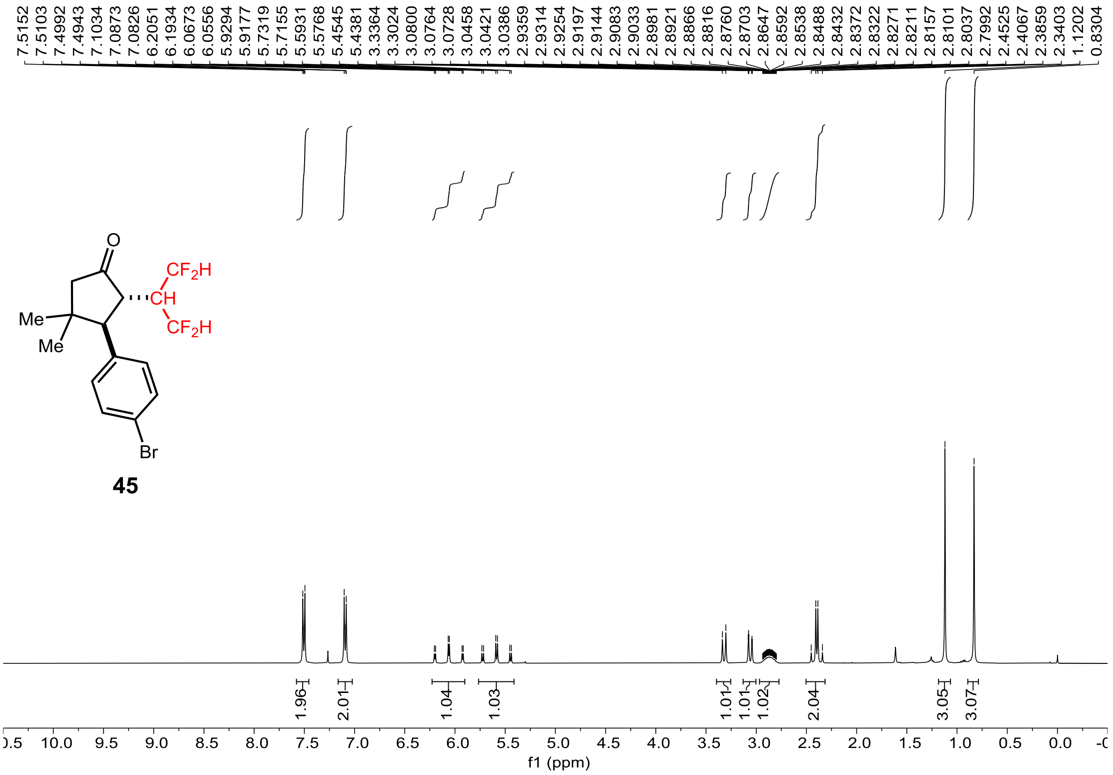

Supplementary Figure 122 ^{1}H NMR (400 MHz, CDCl$_3$) spectrum of compound 45.

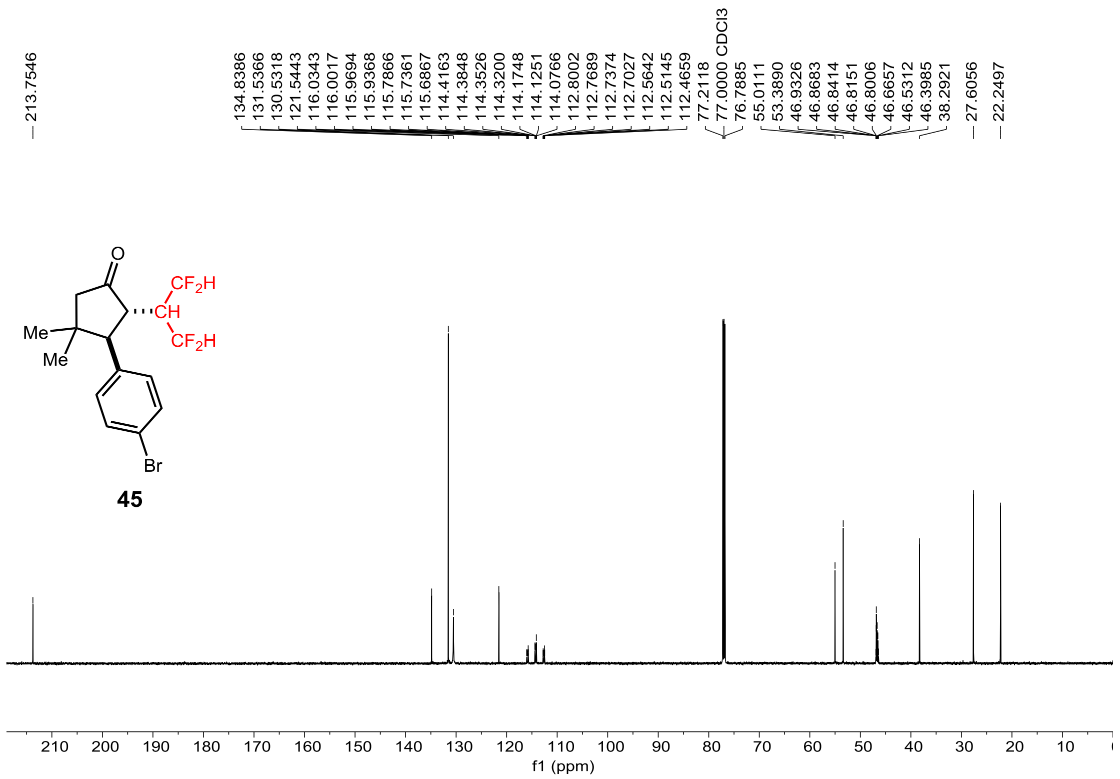

Supplementary Figure 123 ^{13}C NMR (151 MHz, $CDCl_3$) spectrum of compound 45.

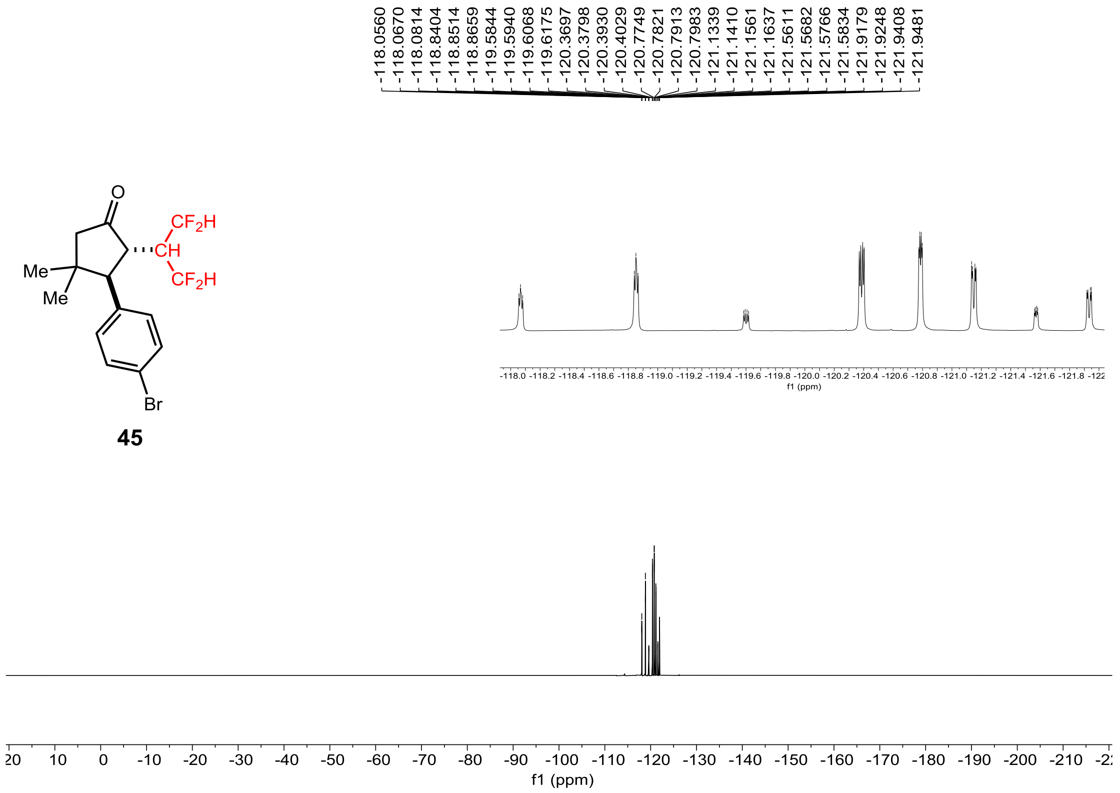

Supplementary Figure 124 ^{19}F NMR (377 MHz, $CDCl_3$) spectrum of compound 45.

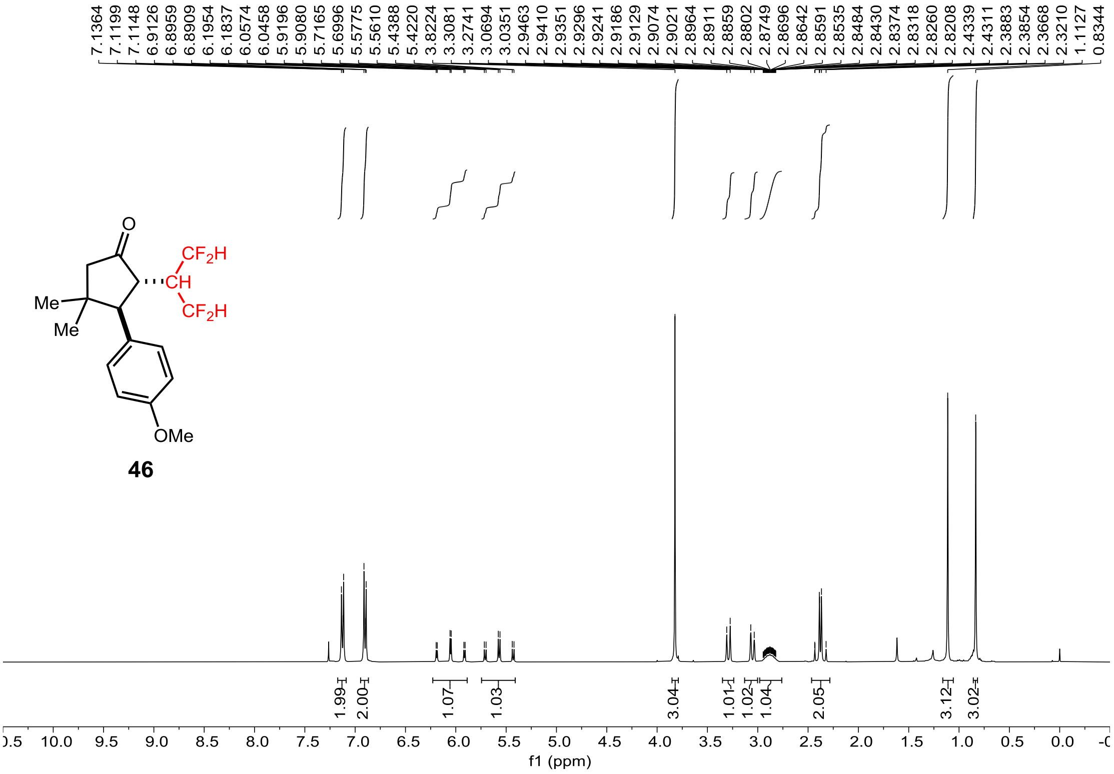

Supplementary Figure 125 ^{1}H NMR (400 MHz, $CDCl_3$) spectrum of compound 46.

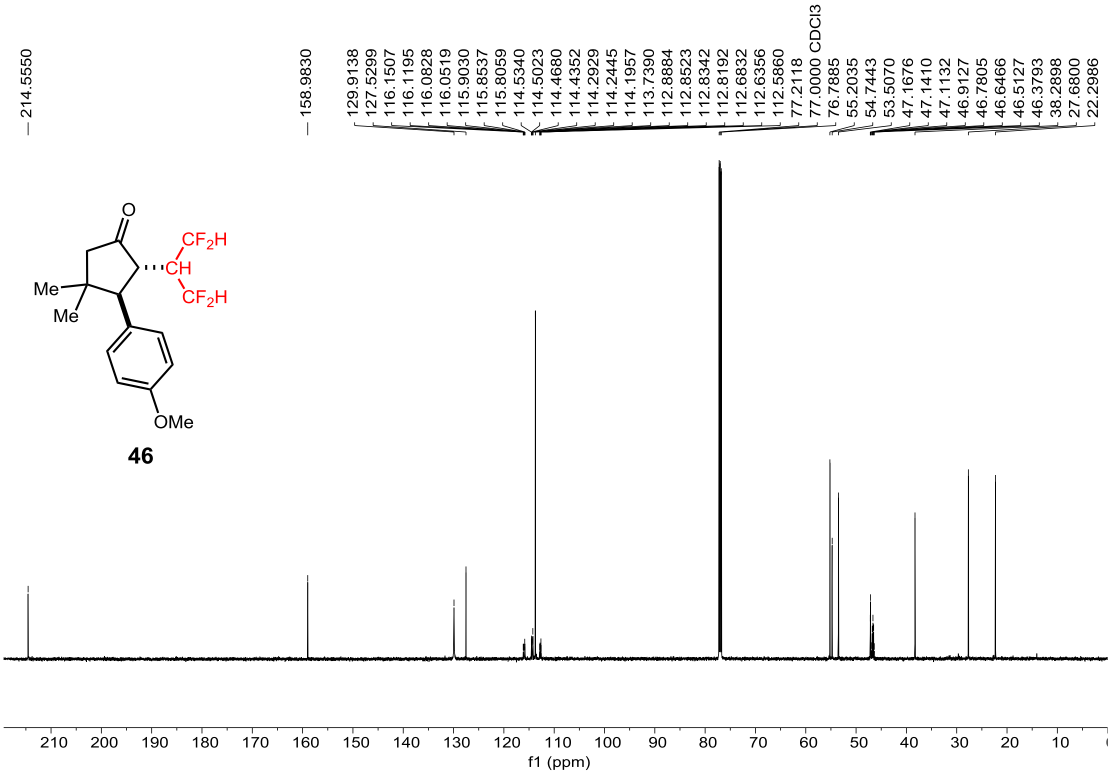

Supplementary Figure 126 ^{13}C NMR (151 MHz, $CDCl_3$) spectrum of compound 46.

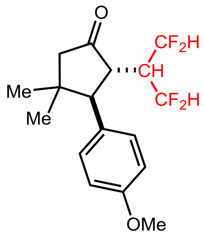

46

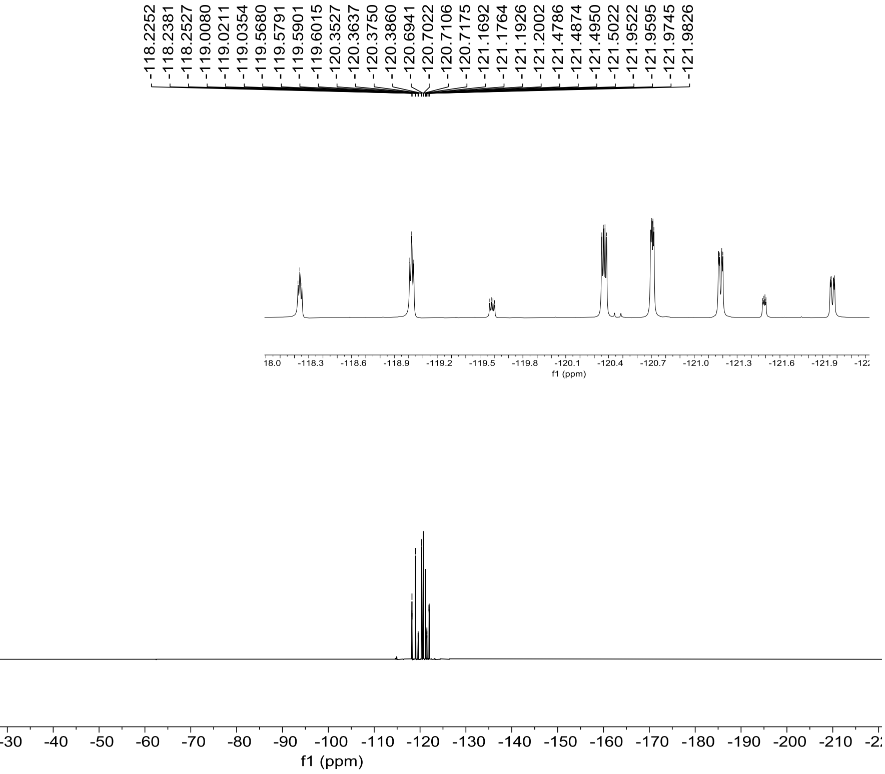

Supplementary Figure 127 ^{19}F NMR (377 MHz, CDCl$_3$) spectrum of compound 46.

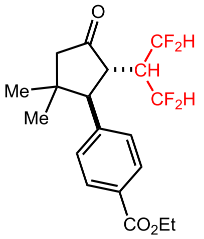

47

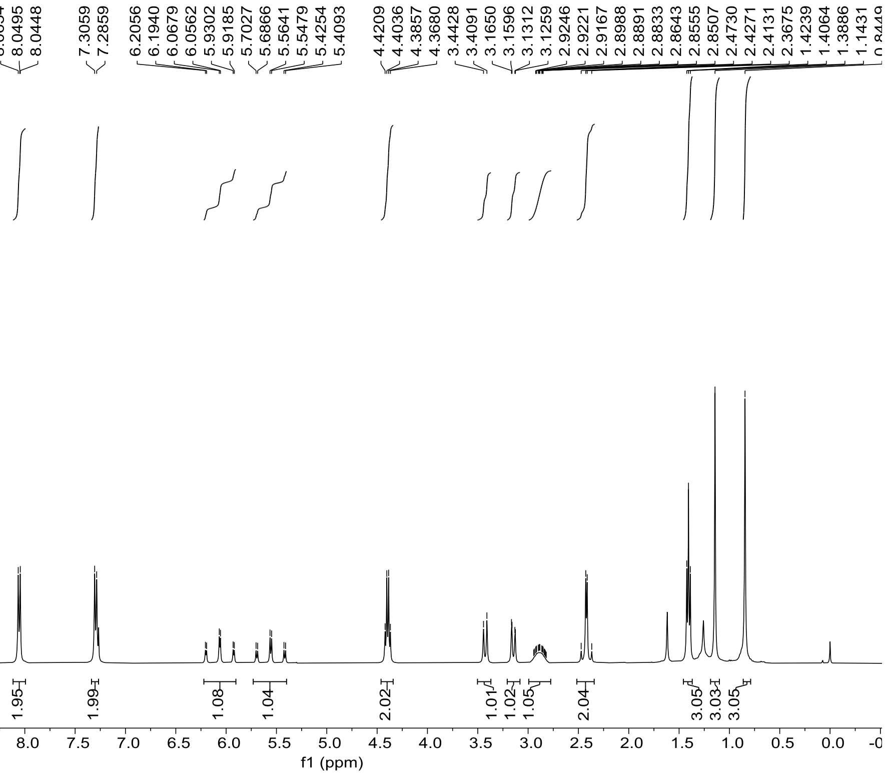

Supplementary Figure 128 ^{1}H NMR (400 MHz, CDCl$_3$) spectrum of compound 47.

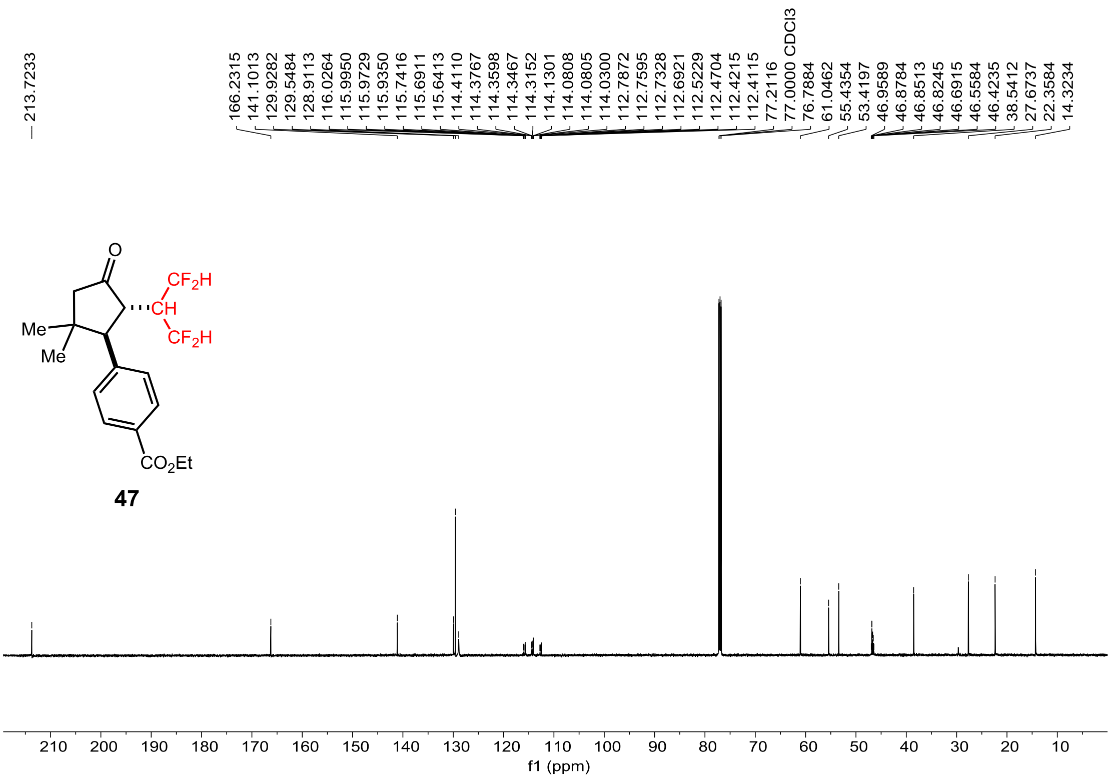

Supplementary Figure 129 ^{13}C NMR (151 MHz, $CDCl_3$) spectrum of compound 47.

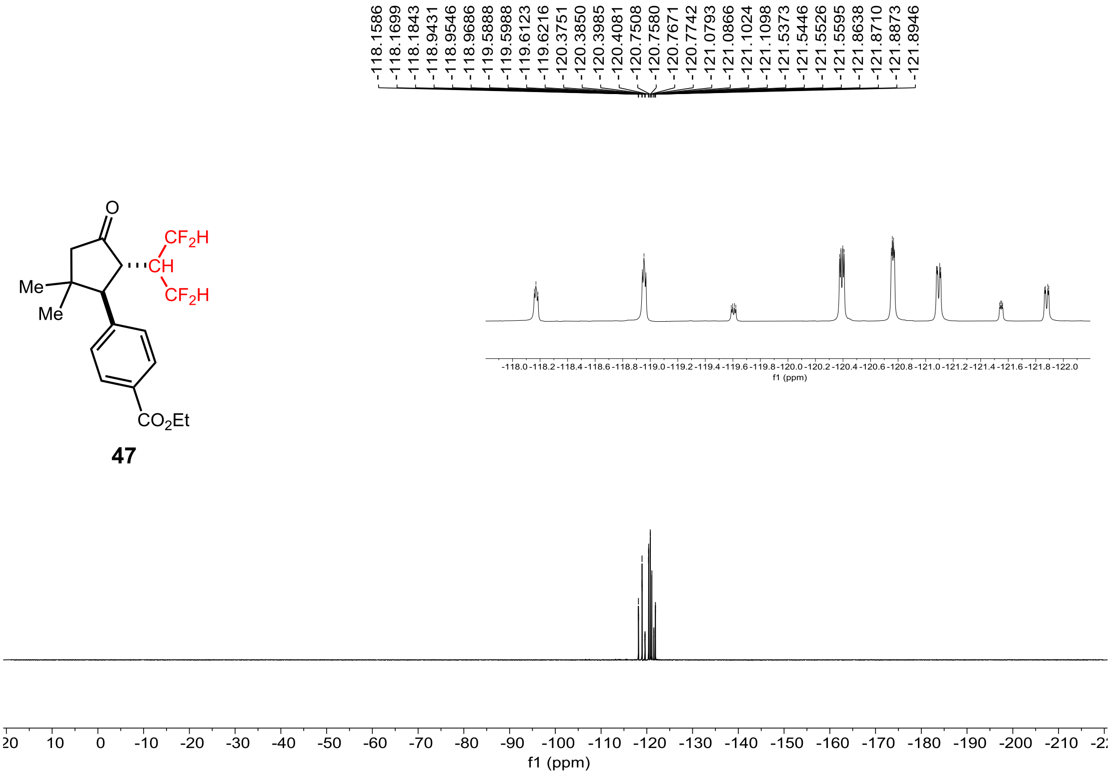

Supplementary Figure 130 ^{19}F NMR (377 MHz, $CDCl_3$) spectrum of compound 47.

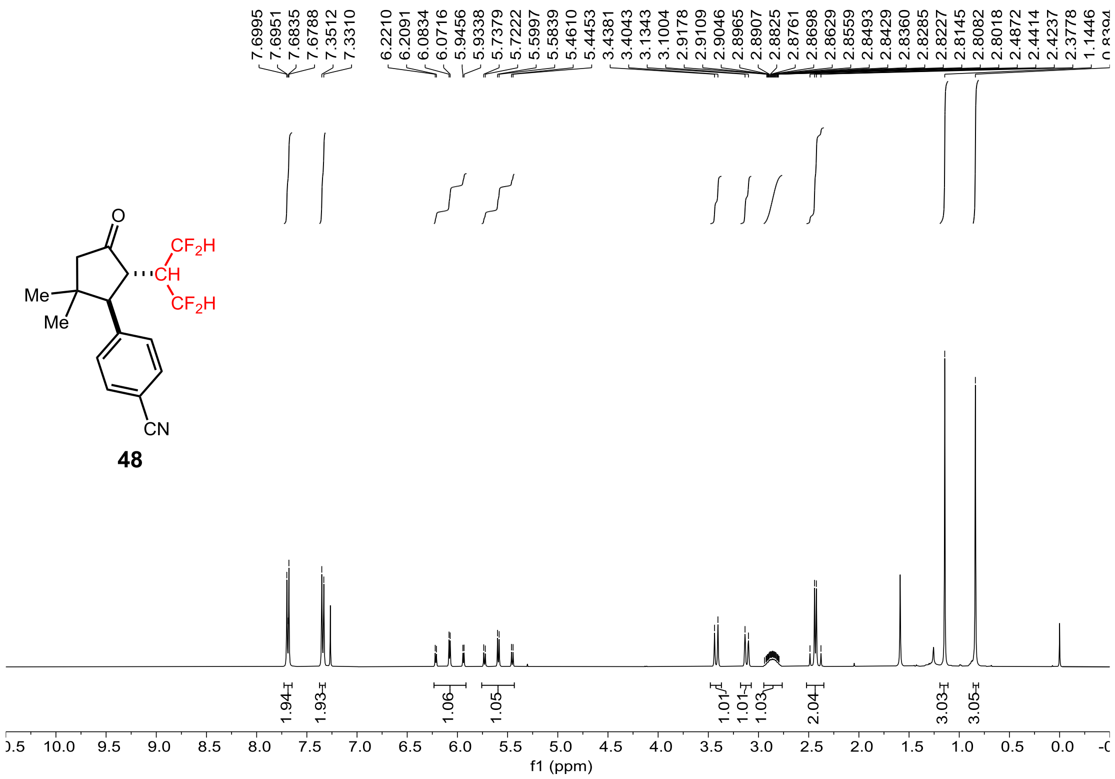

Supplementary Figure 131 ^{1}H NMR (400 MHz, CDCl$_3$) spectrum of compound 48.

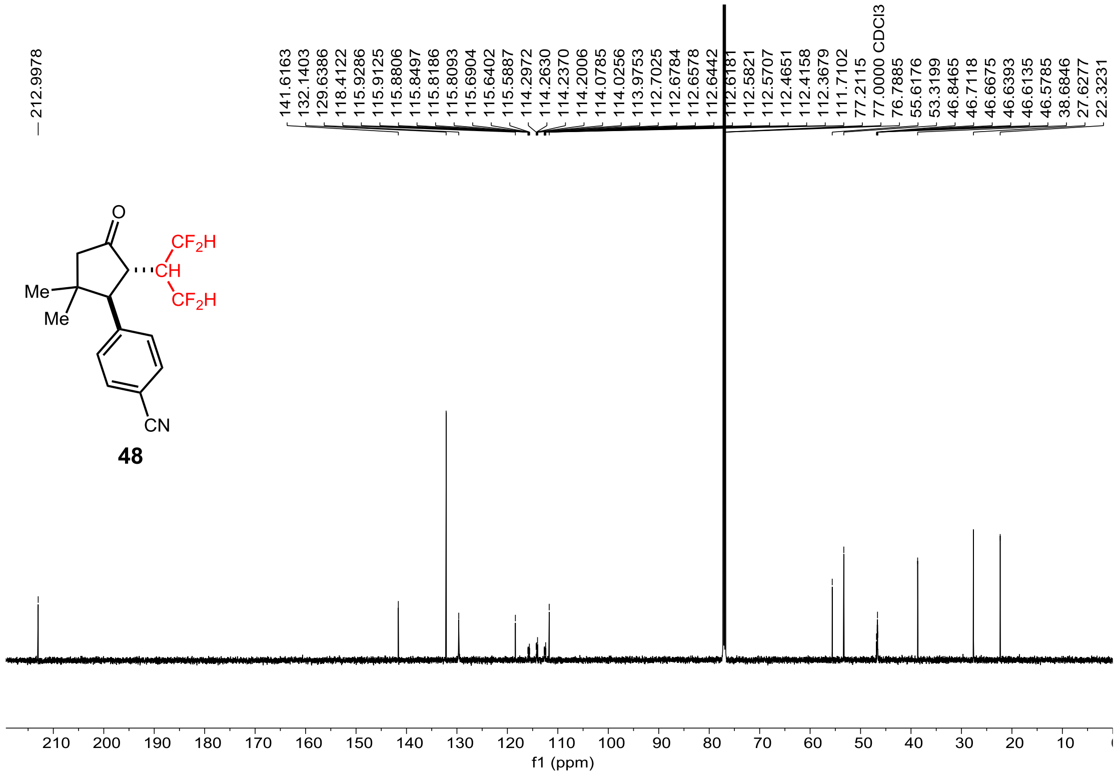

Supplementary Figure 132 ^{13}C NMR (151 MHz, CDCl$_3$) spectrum of compound 48.

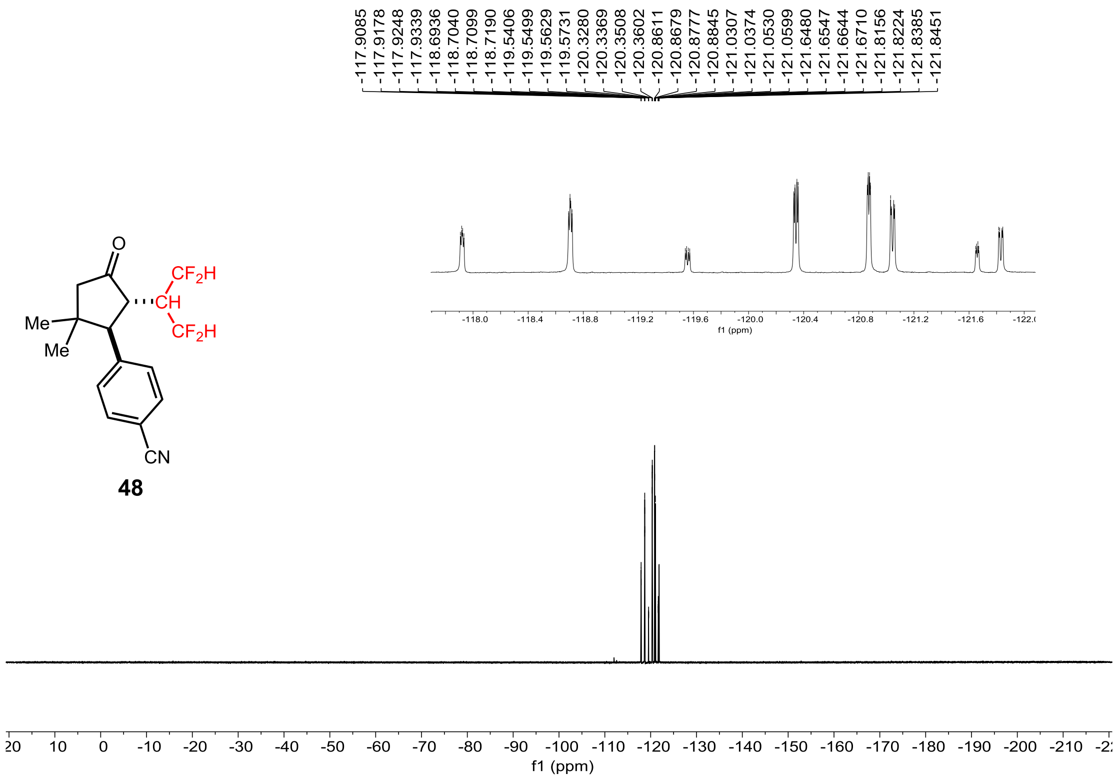

Supplementary Figure 133 ^{19}F NMR (377 MHz, $CDCl_3$) spectrum of compound 48.

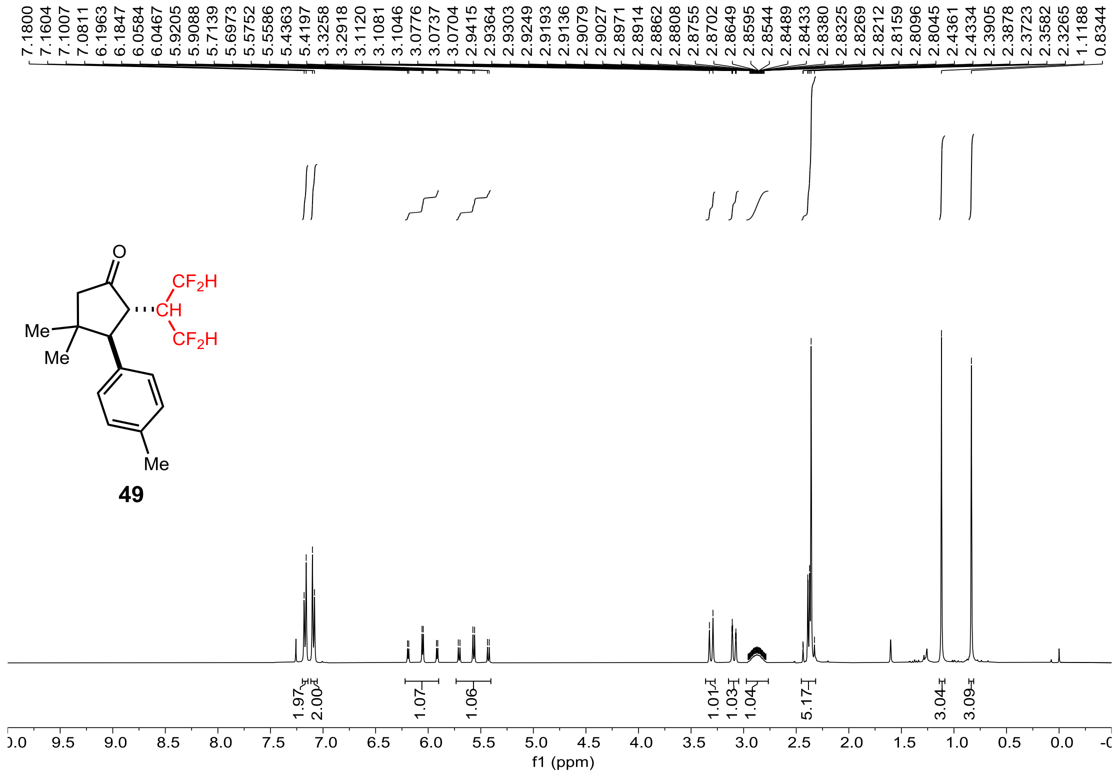

Supplementary Figure 134 ^{1}H NMR (400 MHz, $CDCl_3$) spectrum of compound 49.

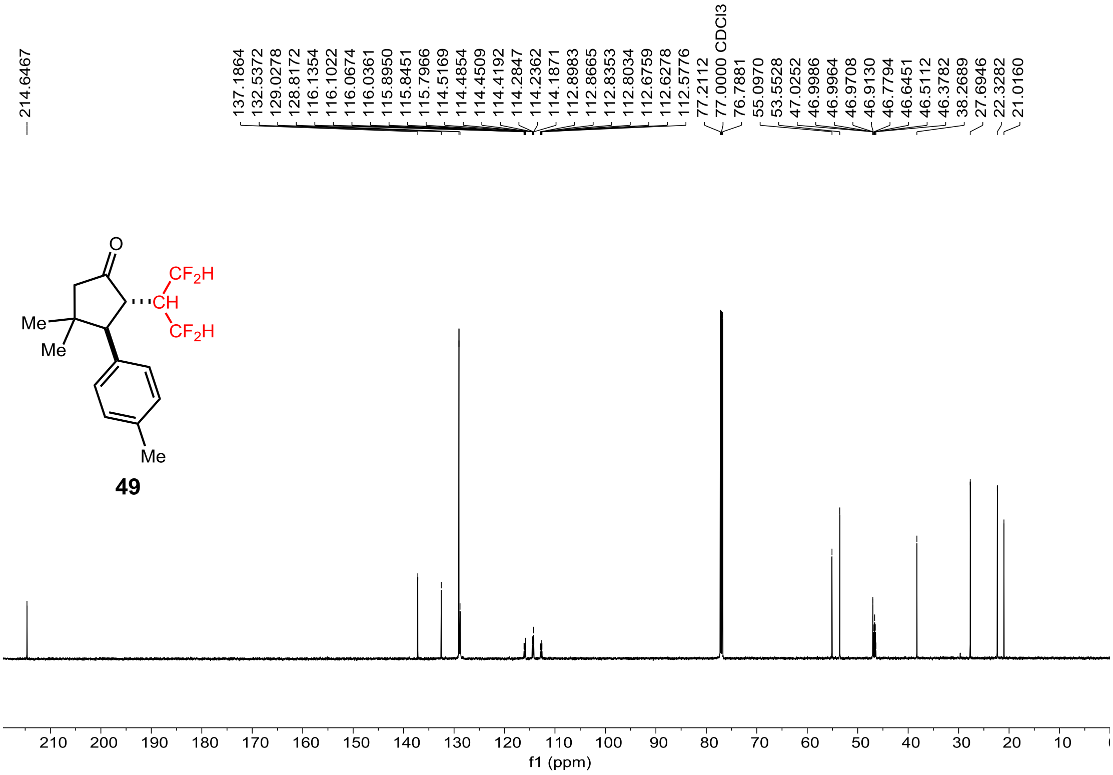

Supplementary Figure 135 ^{13}C NMR (151 MHz, $CDCl_3$) spectrum of compound 49.

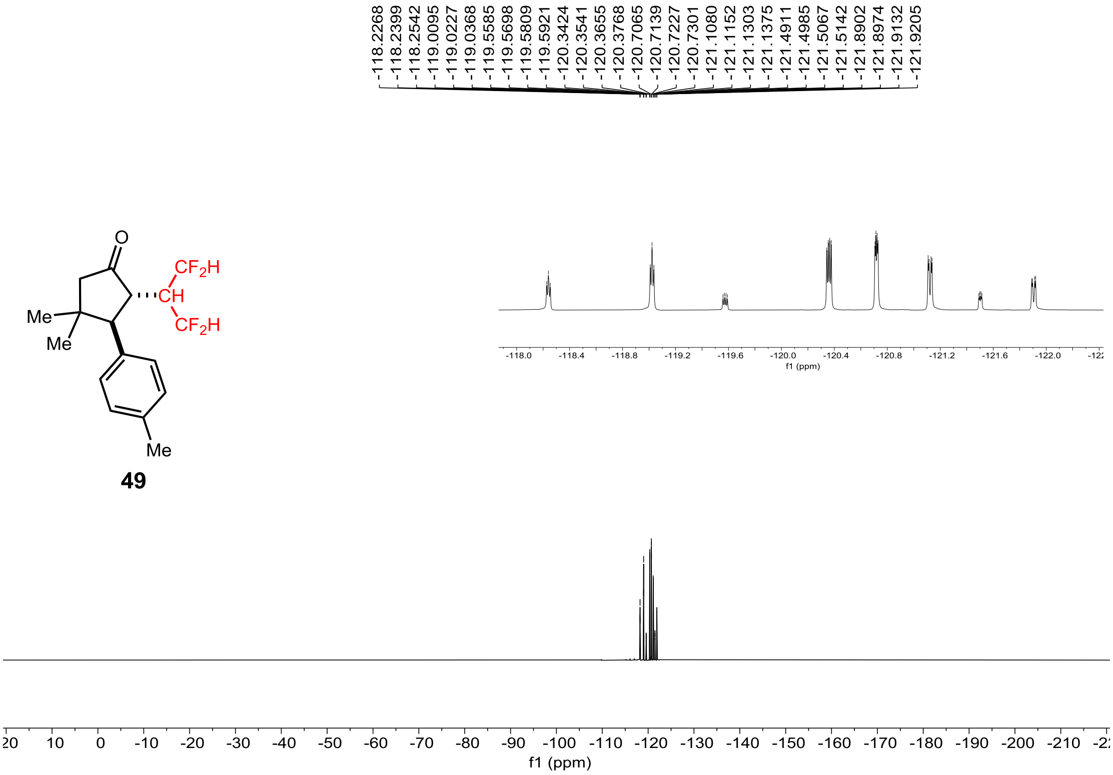

Supplementary Figure 136 ^{19}F NMR (377 MHz, $CDCl_3$) spectrum of compound 49.

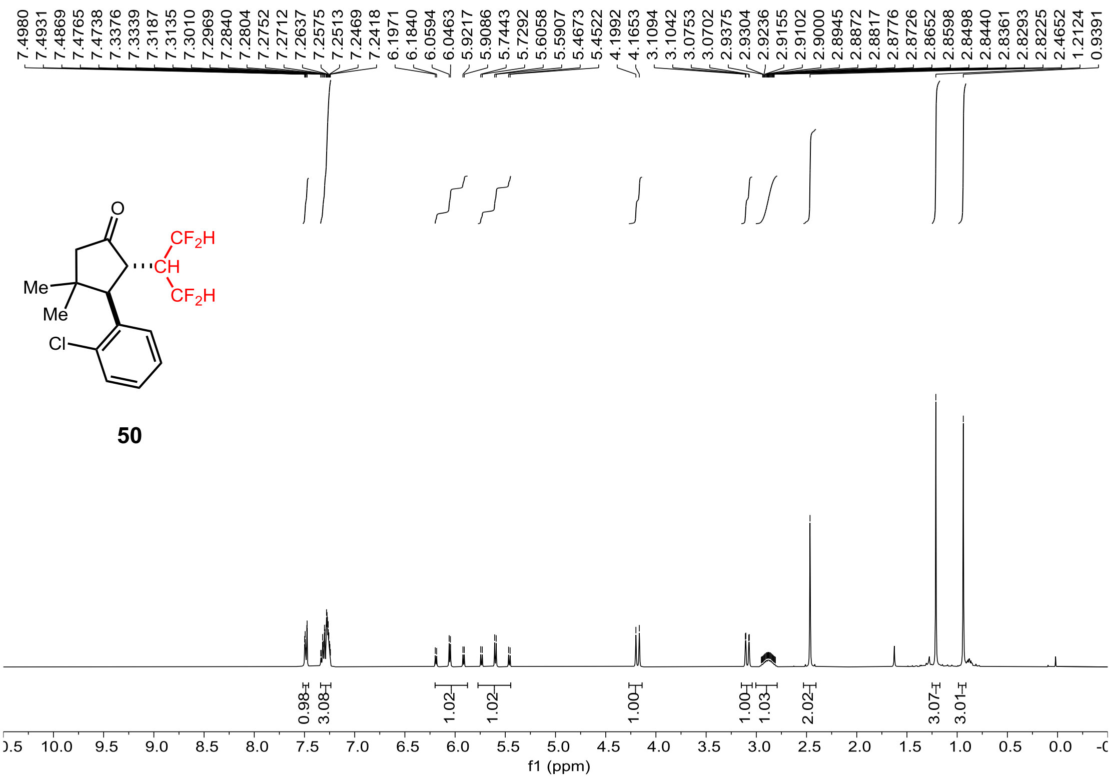

Supplementary Figure 137 ^{1}H NMR (400 MHz, $CDCl_3$) spectrum of compound 50.

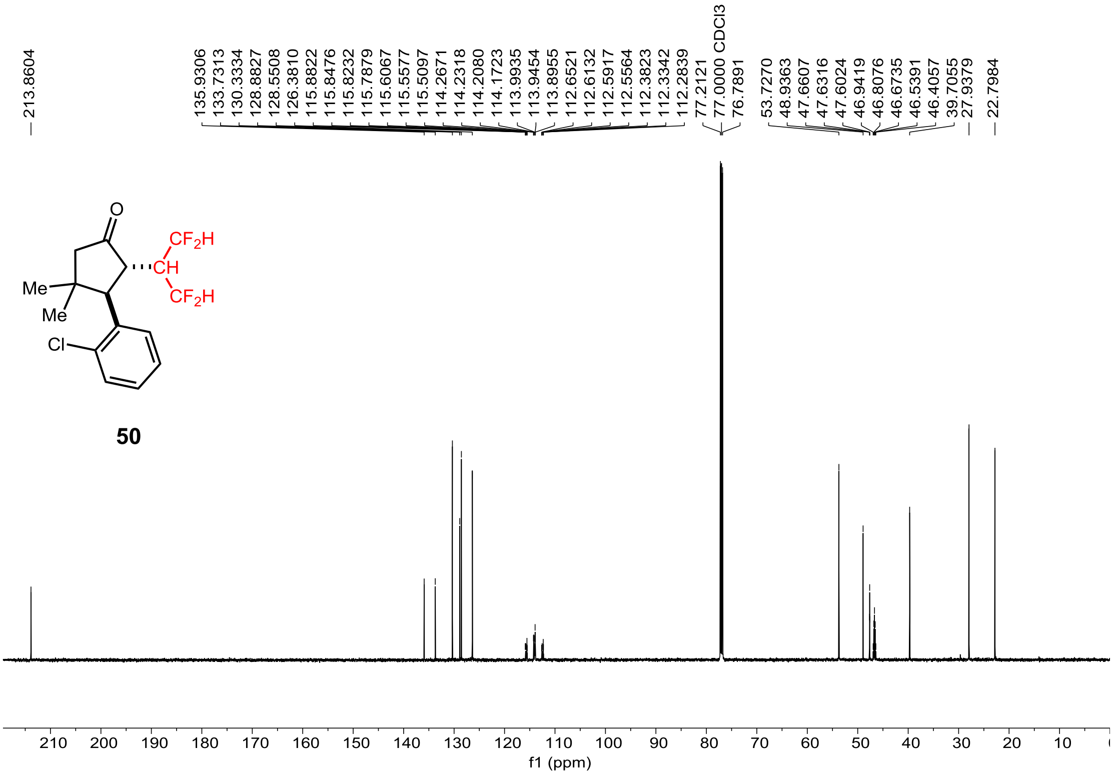

Supplementary Figure 138 ^{13}C NMR (151 MHz, $CDCl_3$) spectrum of compound 50.

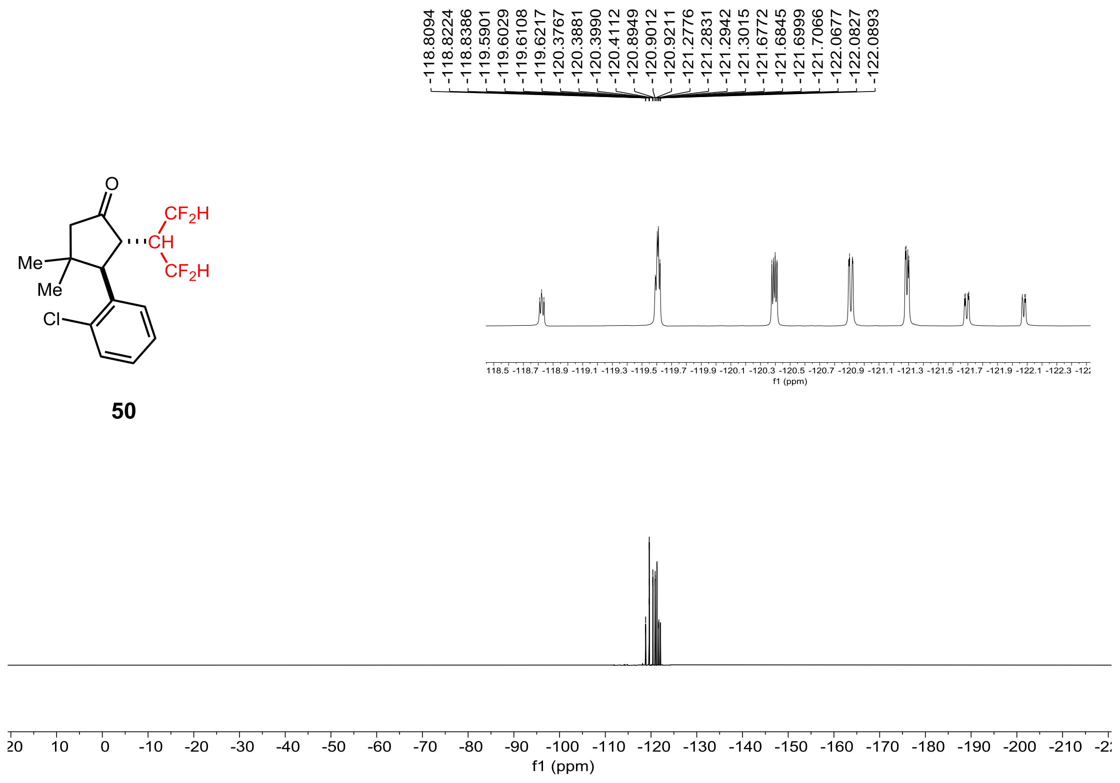

Supplementary Figure 139 ^{19}F NMR (377 MHz, $CDCl_3$) spectrum of compound 50.

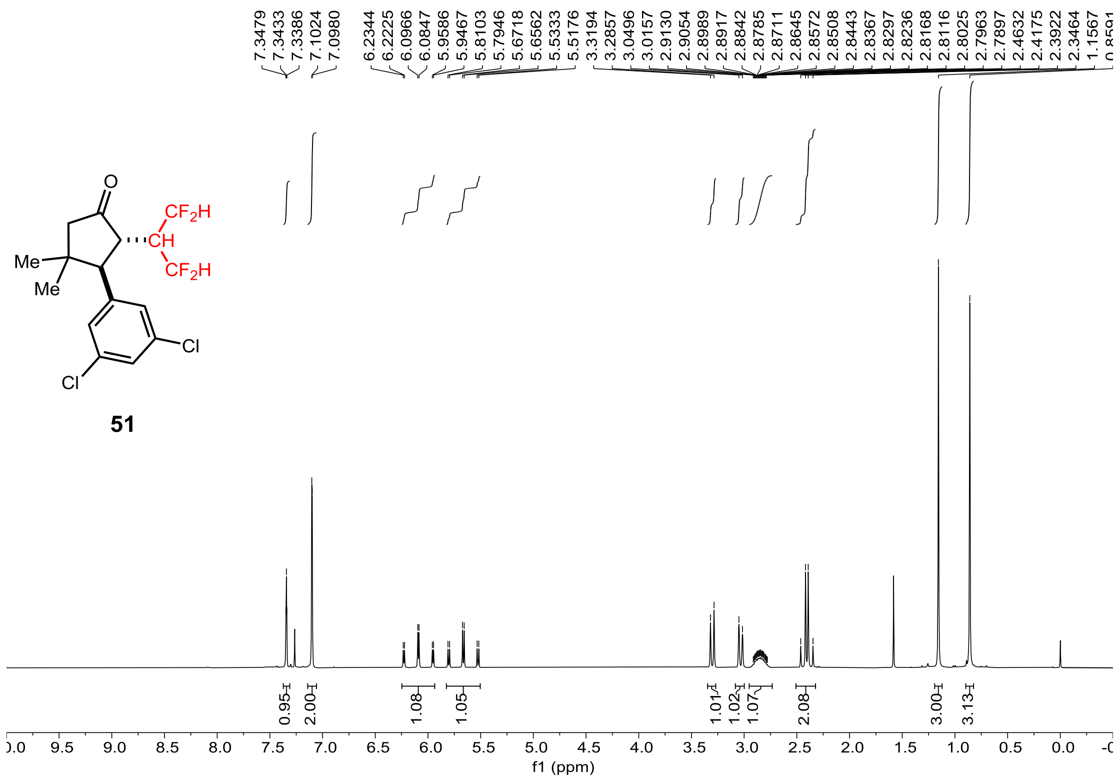

Supplementary Figure 140 ^{1}H NMR (400 MHz, $CDCl_3$) spectrum of compound 51.

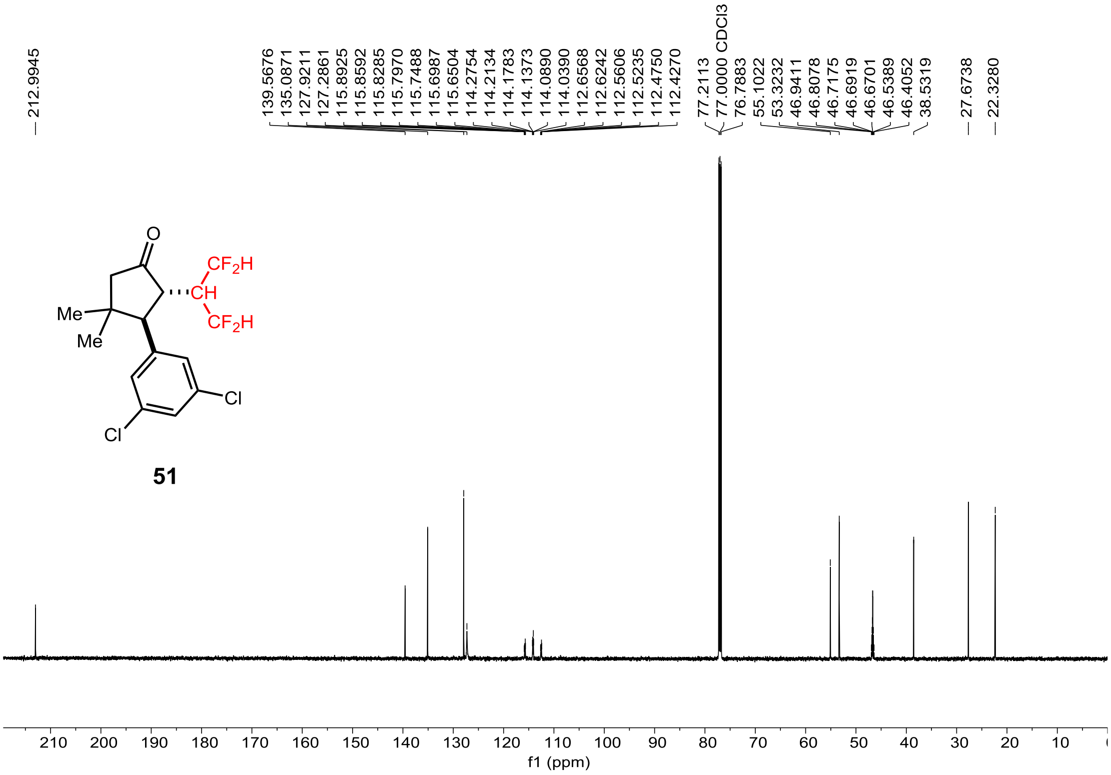

Supplementary Figure 141 ^{13}C NMR (151 MHz, CDCl$_3$) spectrum of compound 51.

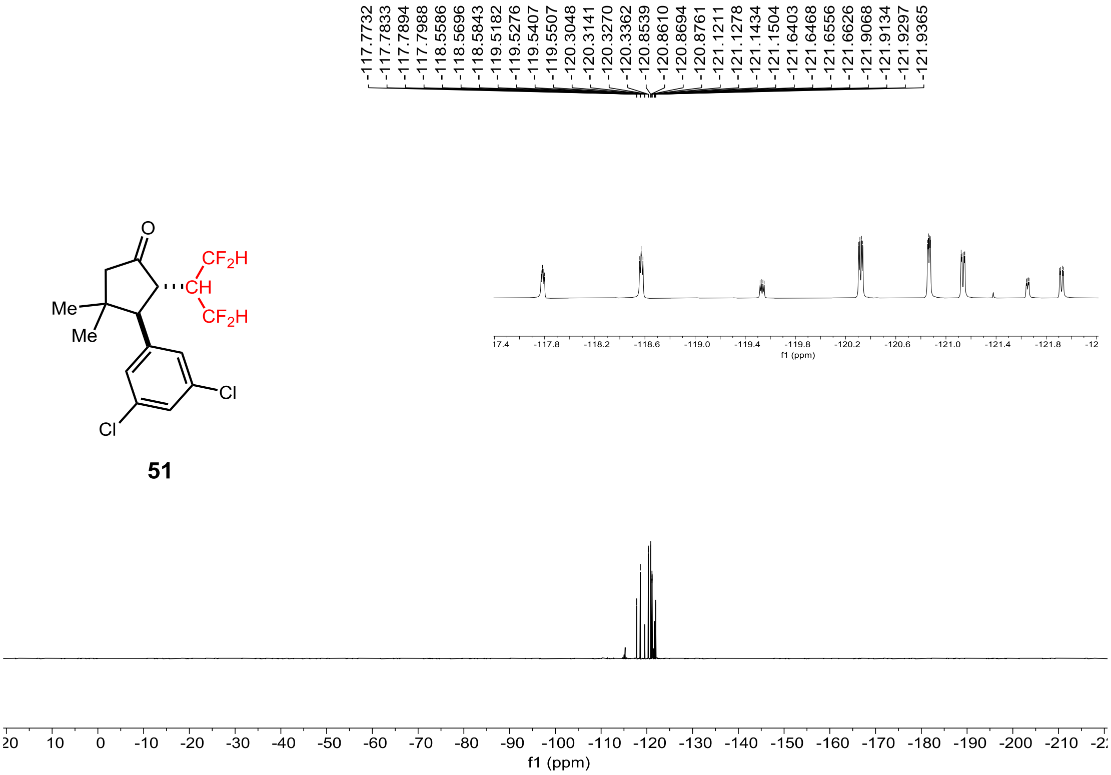

Supplementary Figure 142 ^{19}F NMR (377 MHz, CDCl$_3$) spectrum of compound 51.

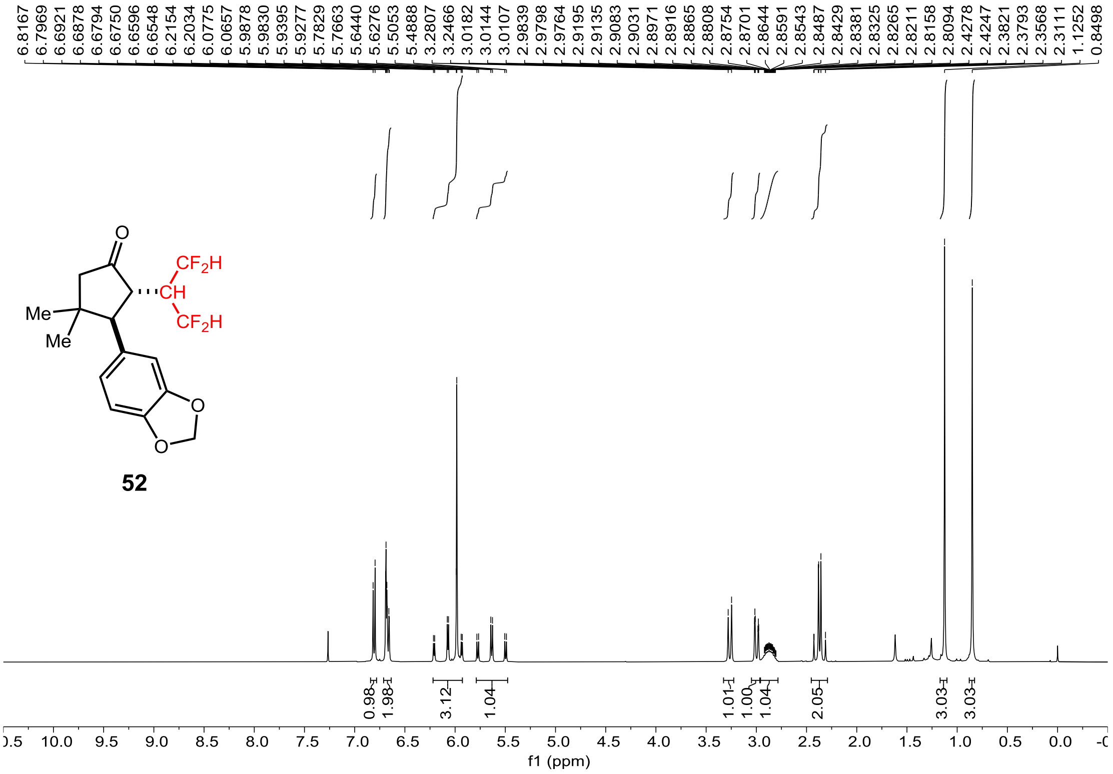

Supplementary Figure 143 ^{1}H NMR (400 MHz, $CDCl_3$) spectrum of compound 52.

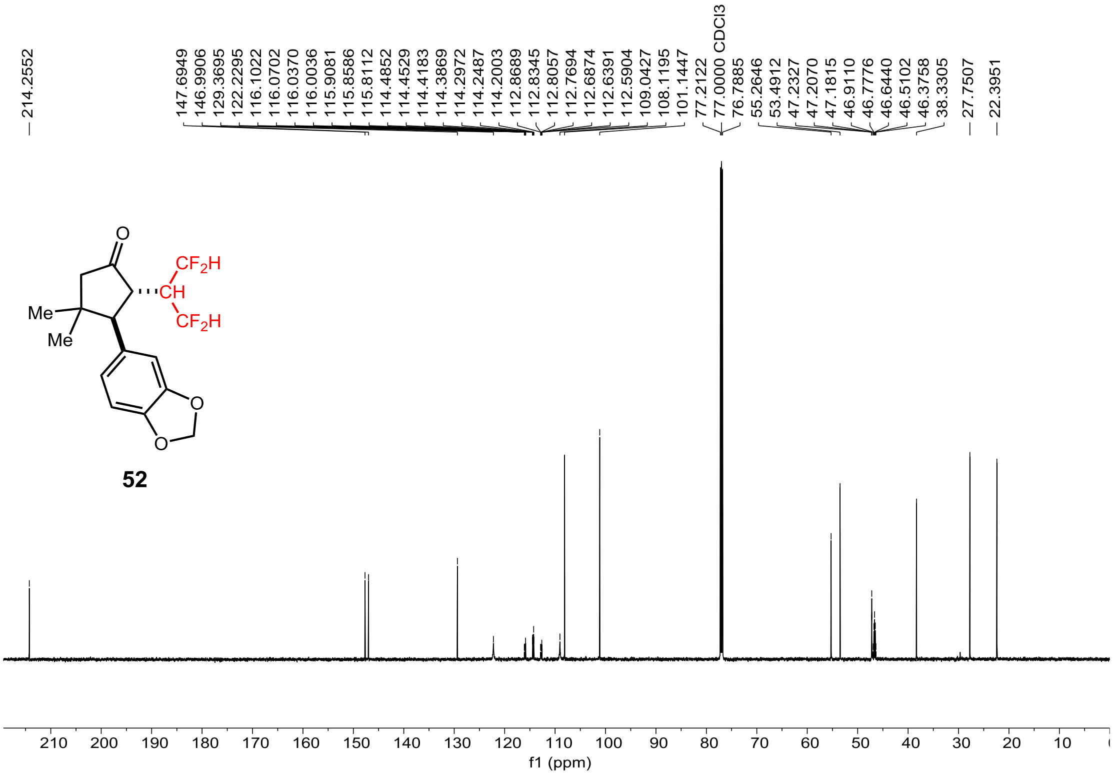

Supplementary Figure 144 ^{13}C NMR (151 MHz, $CDCl_3$) spectrum of compound 52.

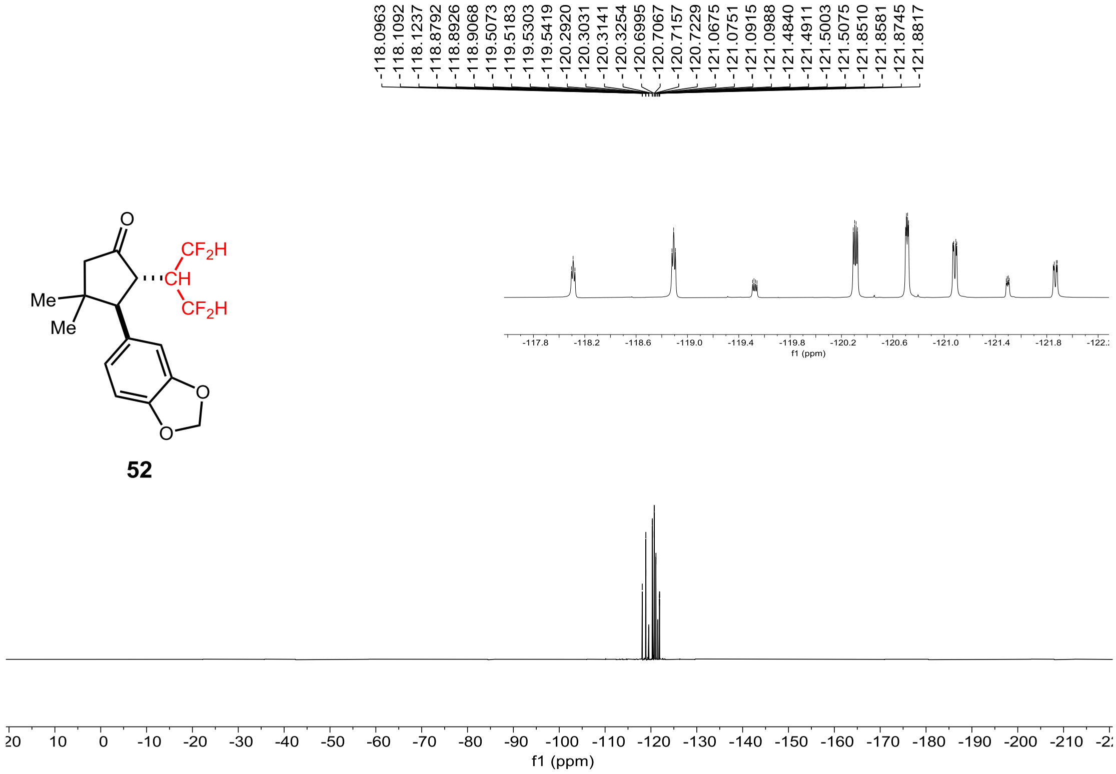

Supplementary Figure 145 ^{19}F NMR (377 MHz, $CDCl_3$) spectrum of compound 52.

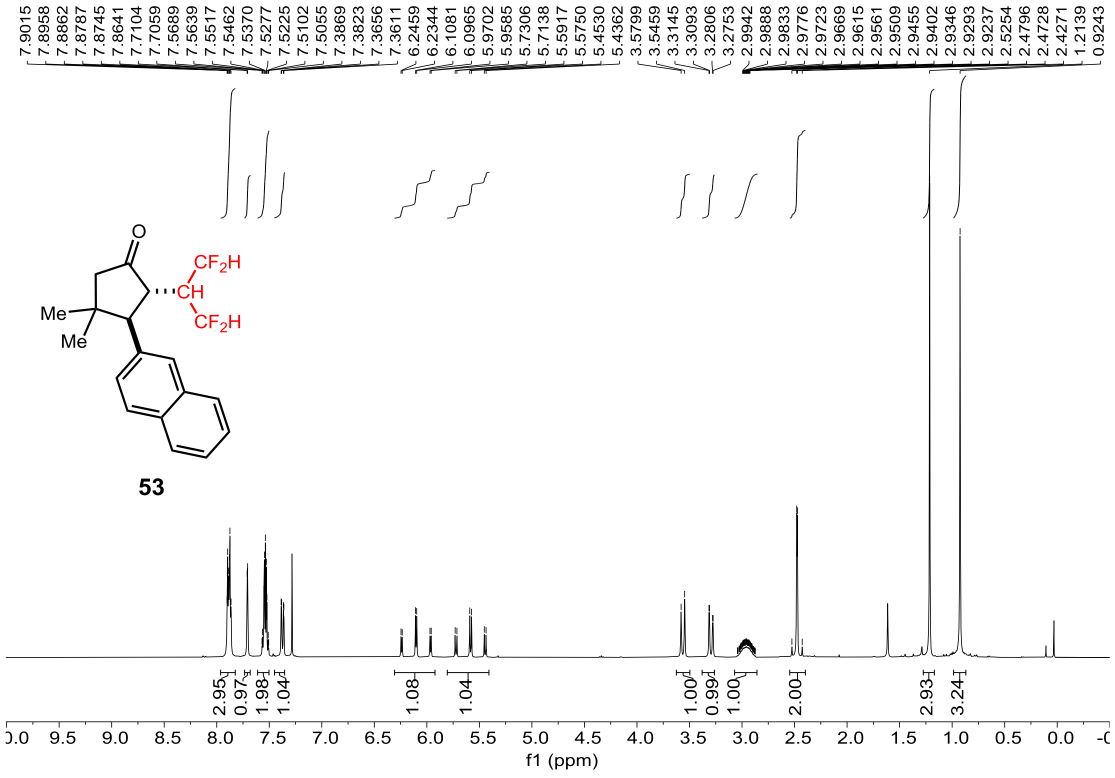

Supplementary Figure 146 ^{1}H NMR (400 MHz, $CDCl_3$) spectrum of compound 53.

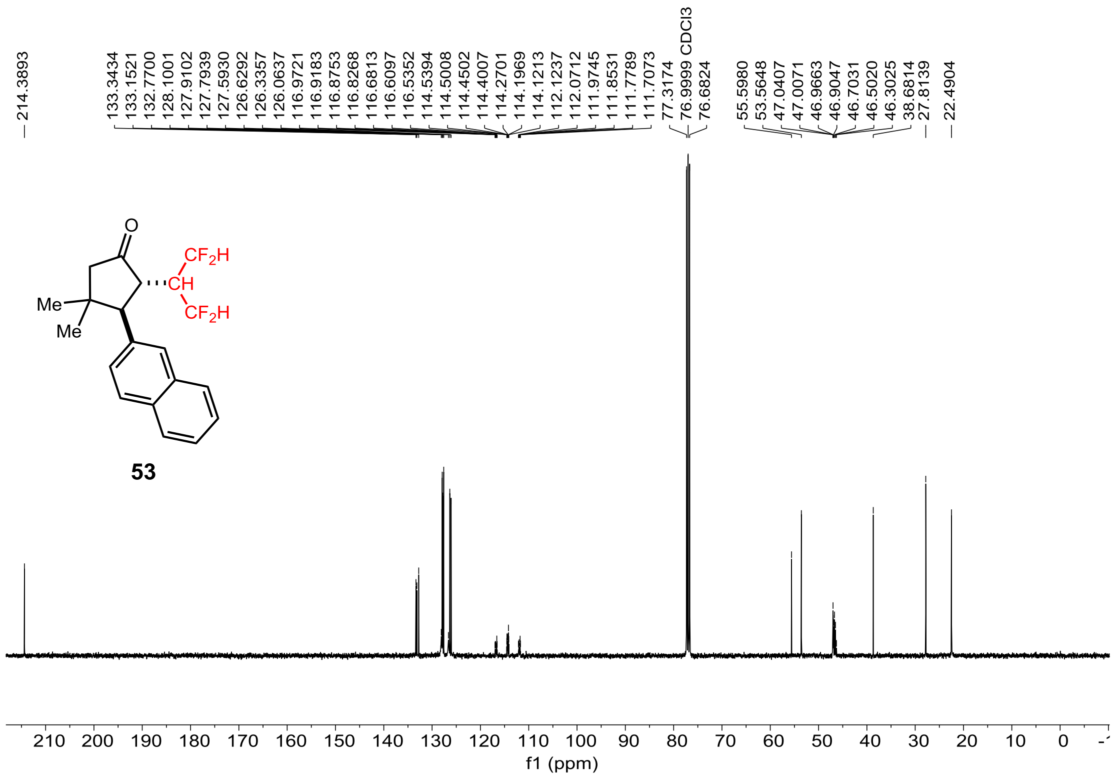

Supplementary Figure 147 ^{13}C NMR (101 MHz, $CDCl_3$) spectrum of compound 53.

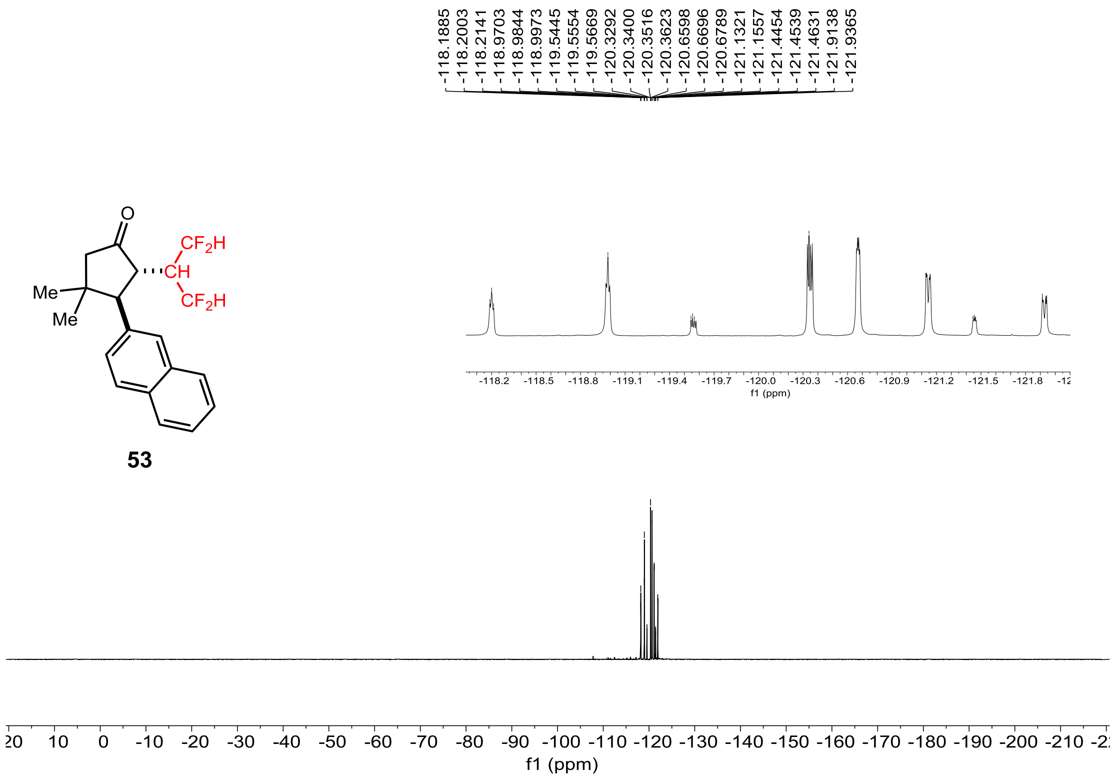

Supplementary Figure 148 ^{19}F NMR (377 MHz, $CDCl_3$) spectrum of compound 53.

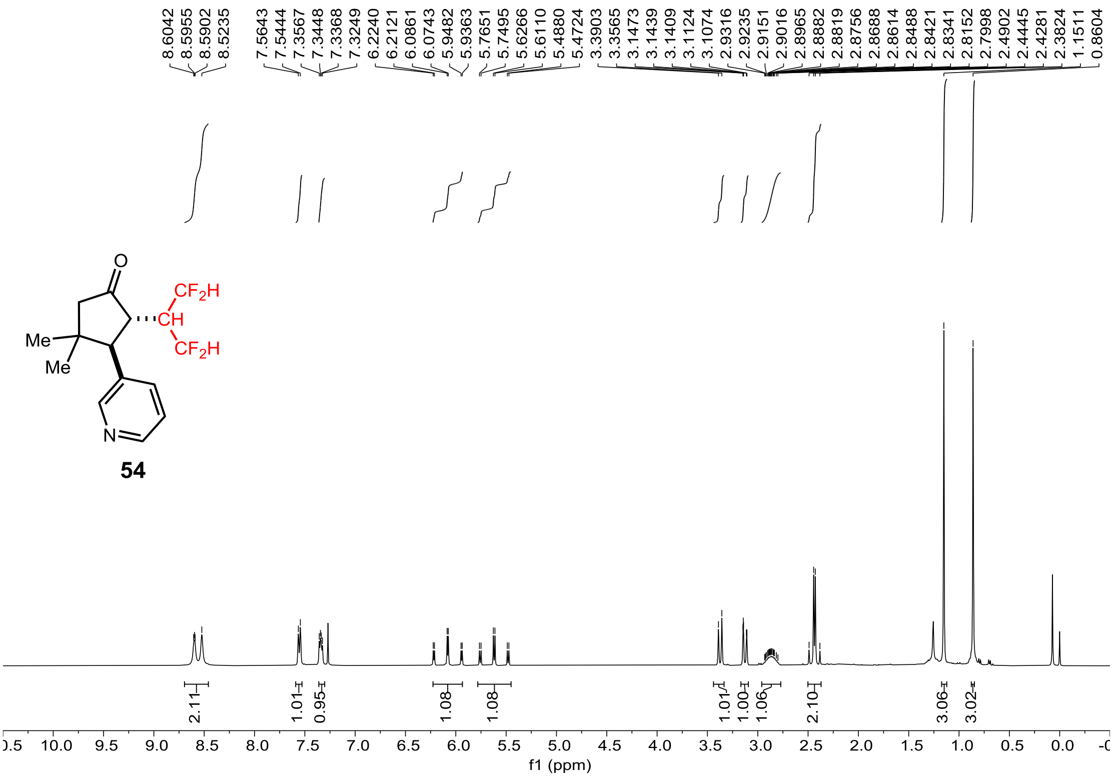

Supplementary Figure 149 ^{1}H NMR (400 MHz, $CDCl_3$) spectrum of compound 54.

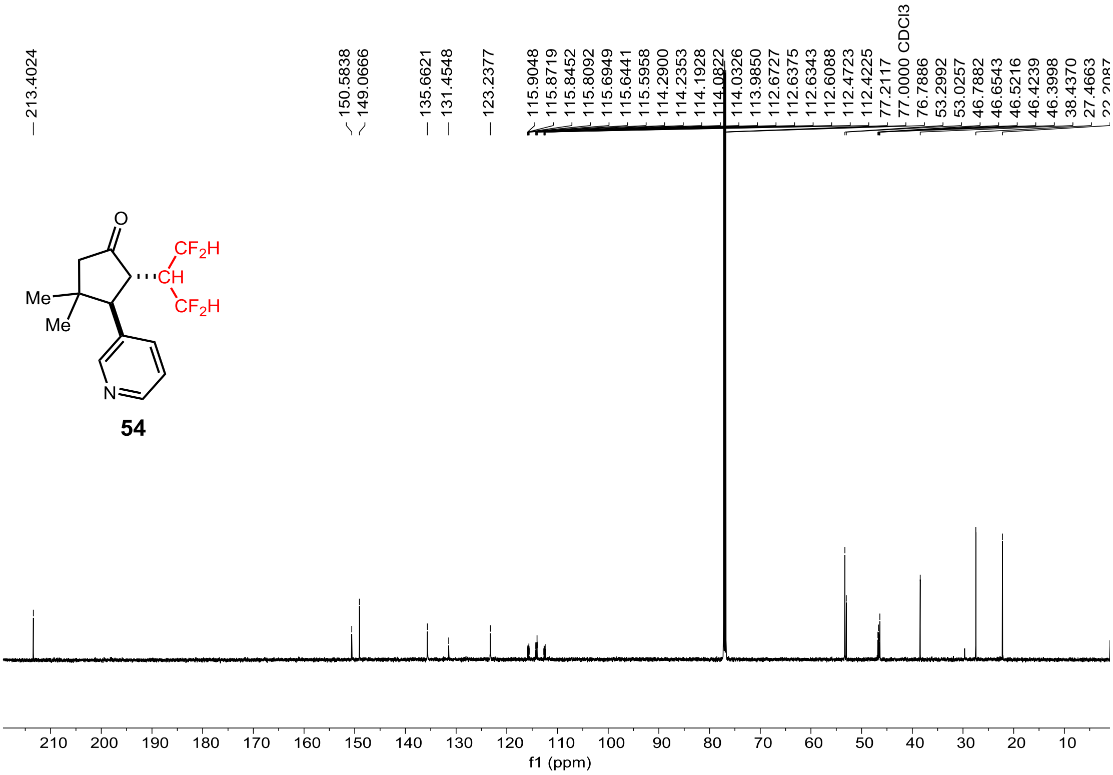

Supplementary Figure 150 ^{13}C NMR (151 MHz, $CDCl_3$) spectrum of compound 54.

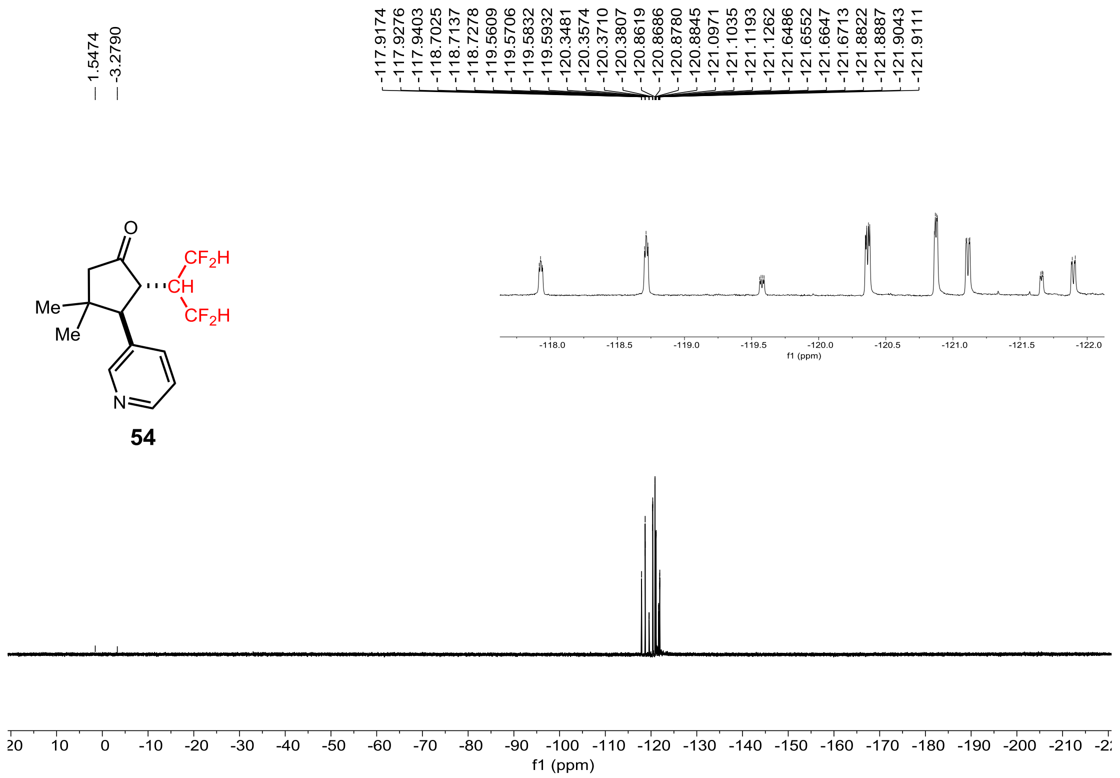

Supplementary Figure 151 ^{19}F NMR (377 MHz, $CDCl_3$) spectrum of compound 54.

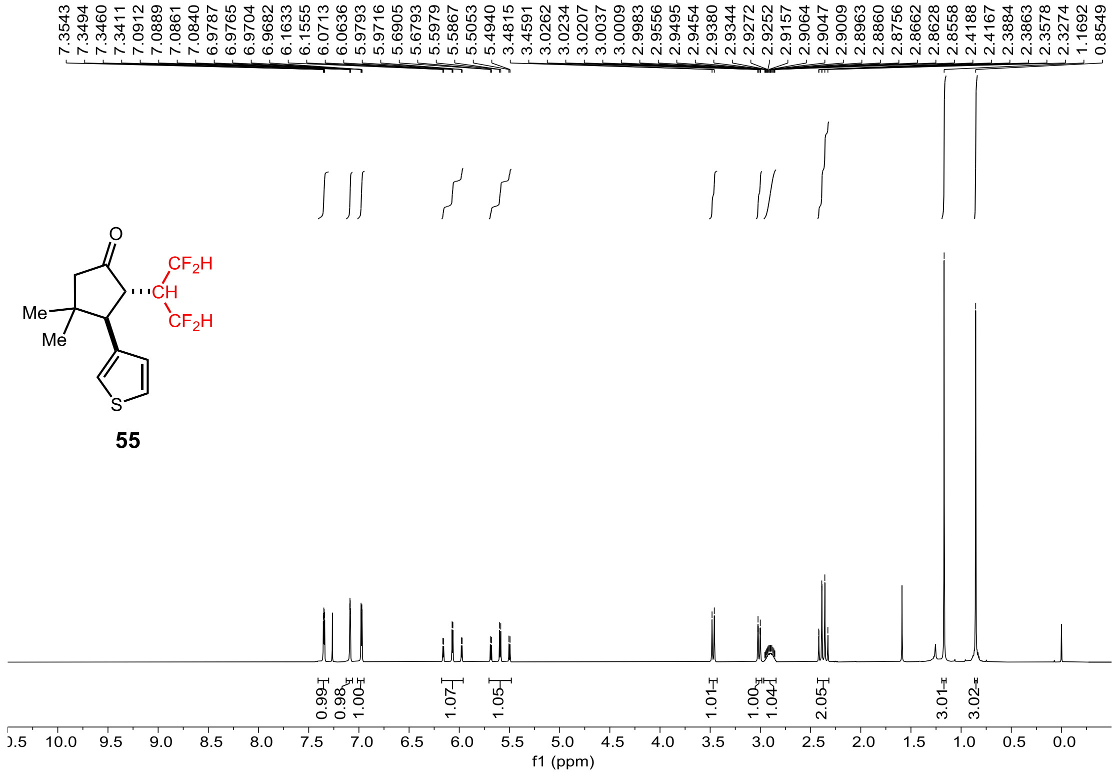

Supplementary Figure 152 ^{1}H NMR (600 MHz, $CDCl_3$) spectrum of compound 55.

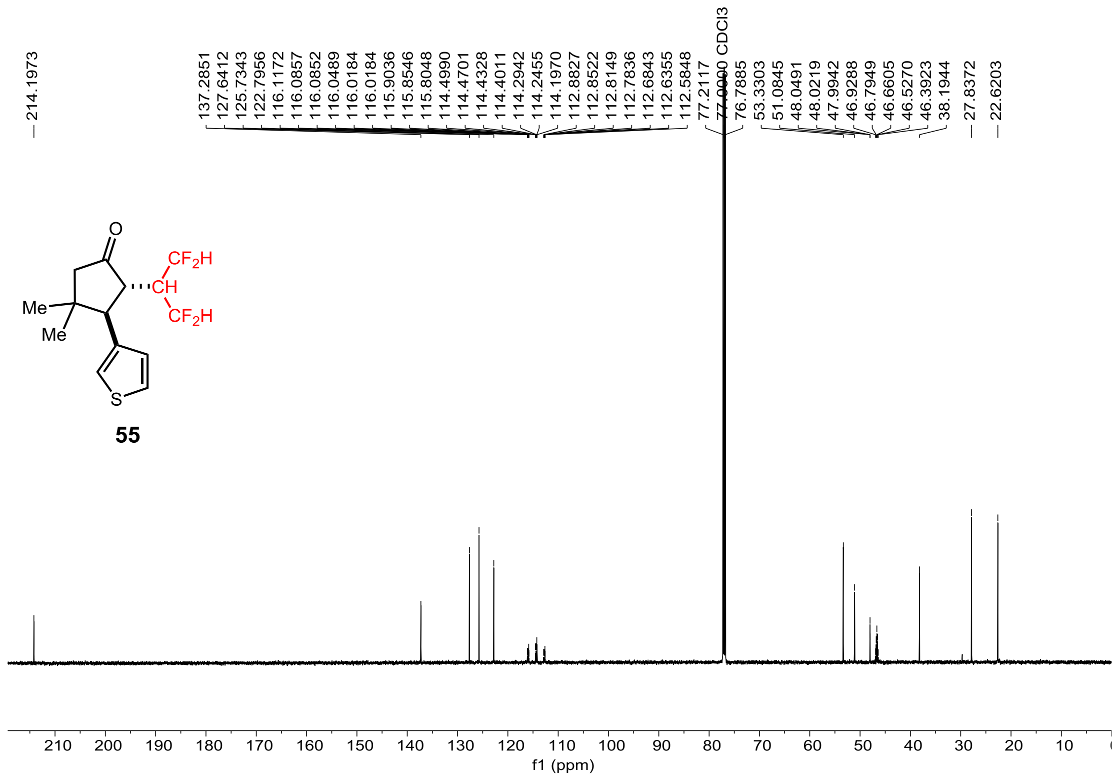

Supplementary Figure 153 ^{13}C NMR (151 MHz, $CDCl_3$) spectrum of compound 55.

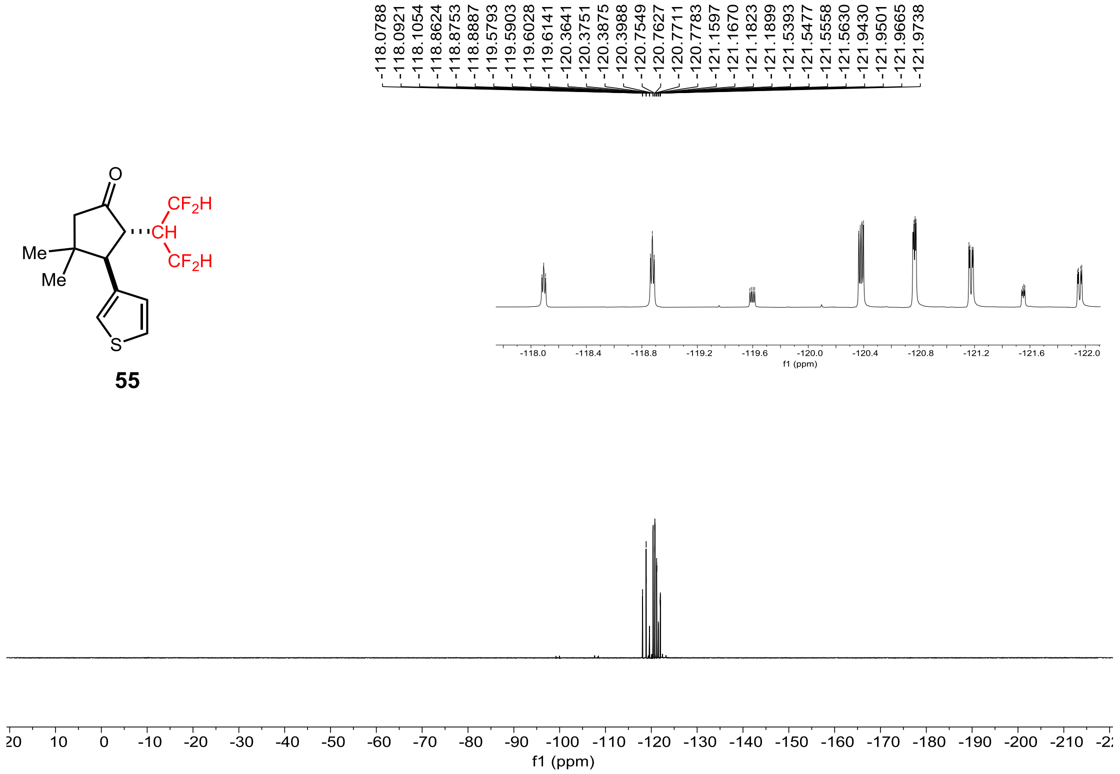

Supplementary Figure 154 ^{19}F NMR (377 MHz, $CDCl_3$) spectrum of compound 55.

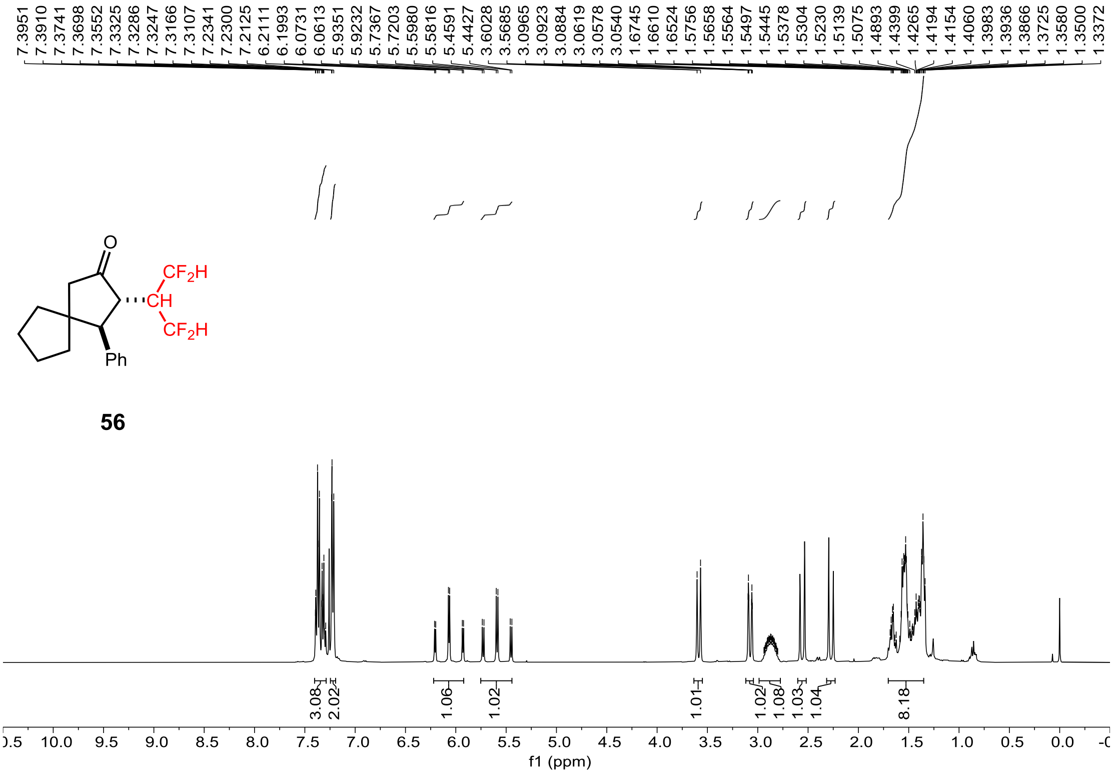

Supplementary Figure 155 ^{1}H NMR (400 MHz, $CDCl_3$) spectrum of compound 56.

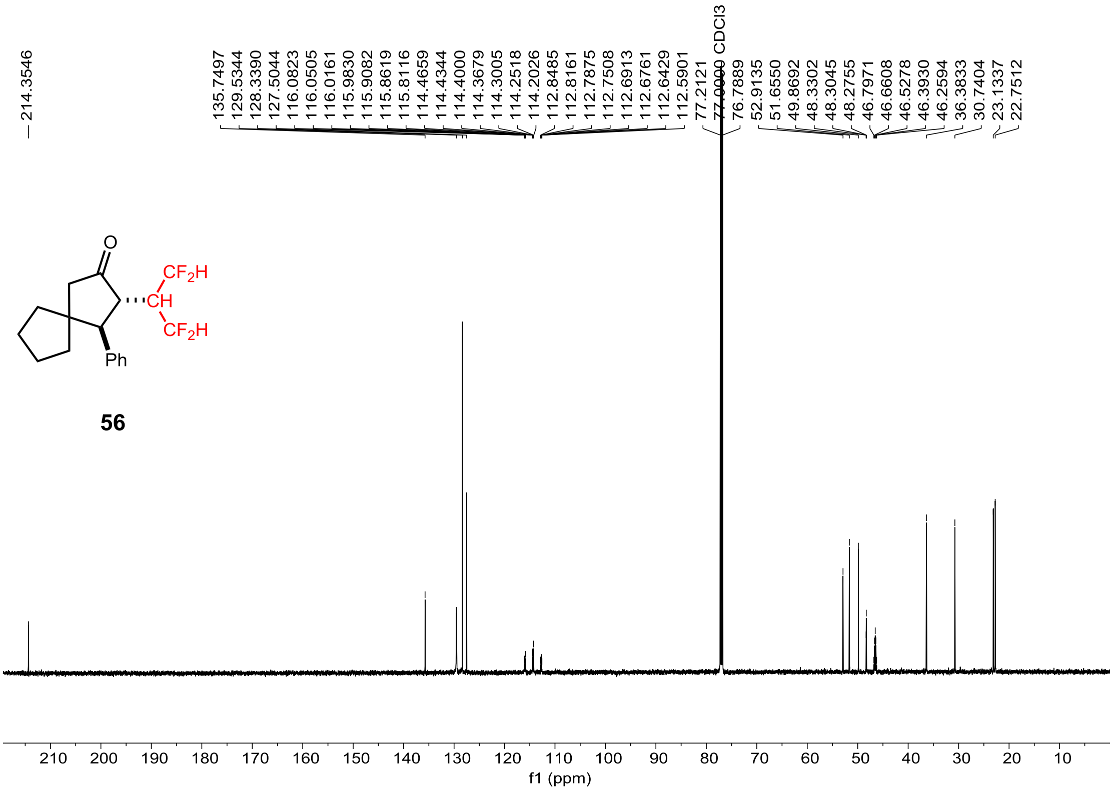

Supplementary Figure 156 ^{13}C NMR (151 MHz, $CDCl_3$) spectrum of compound 56.

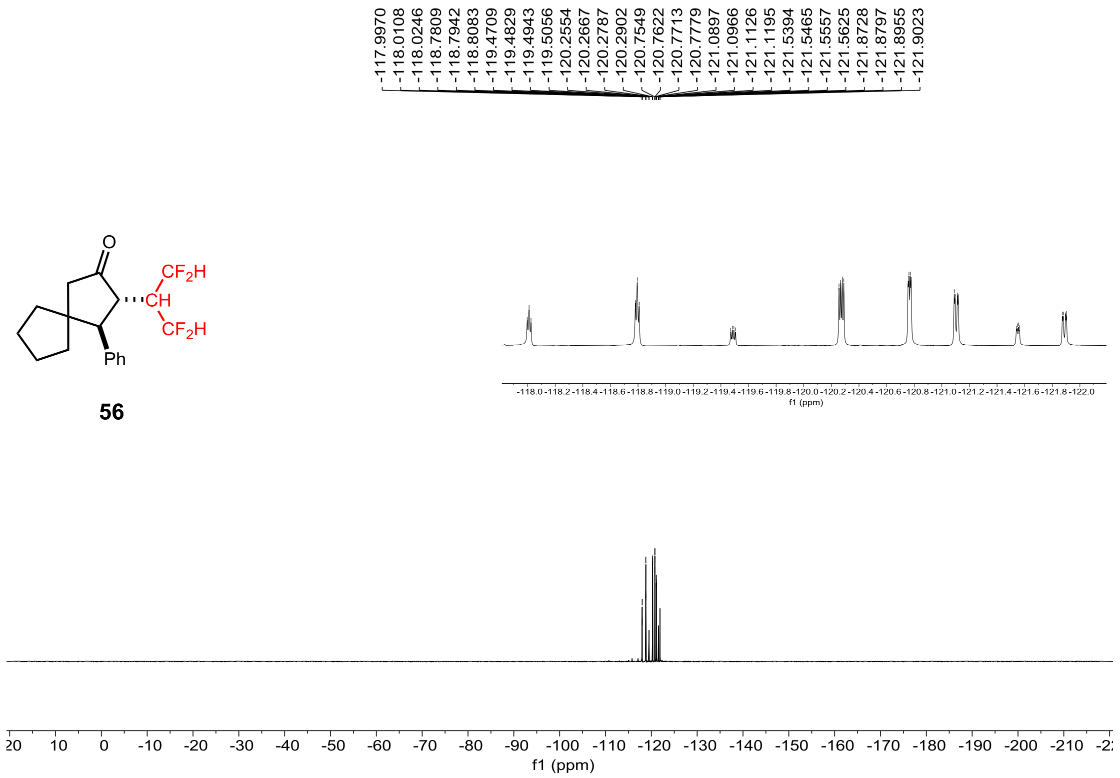

Supplementary Figure 157 ^{19}F NMR (377 MHz, $CDCl_3$) spectrum of compound 56.

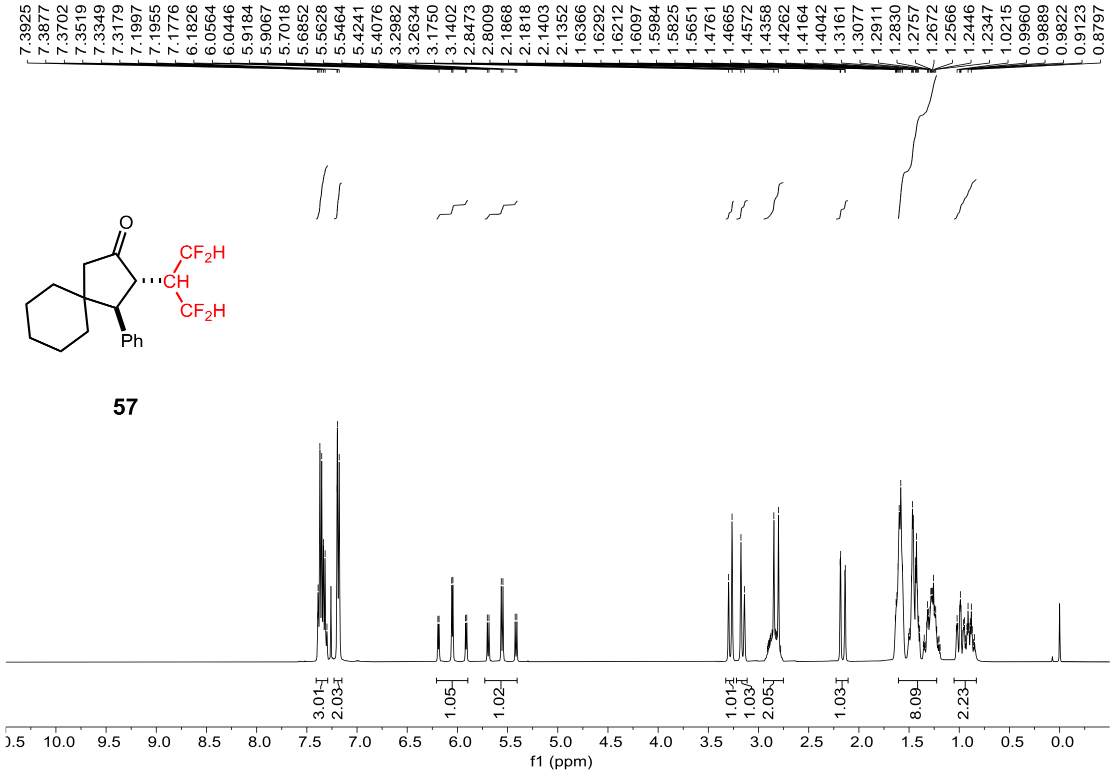

Supplementary Figure 158 ^{1}H NMR (400 MHz, $CDCl_3$) spectrum of compound 57.

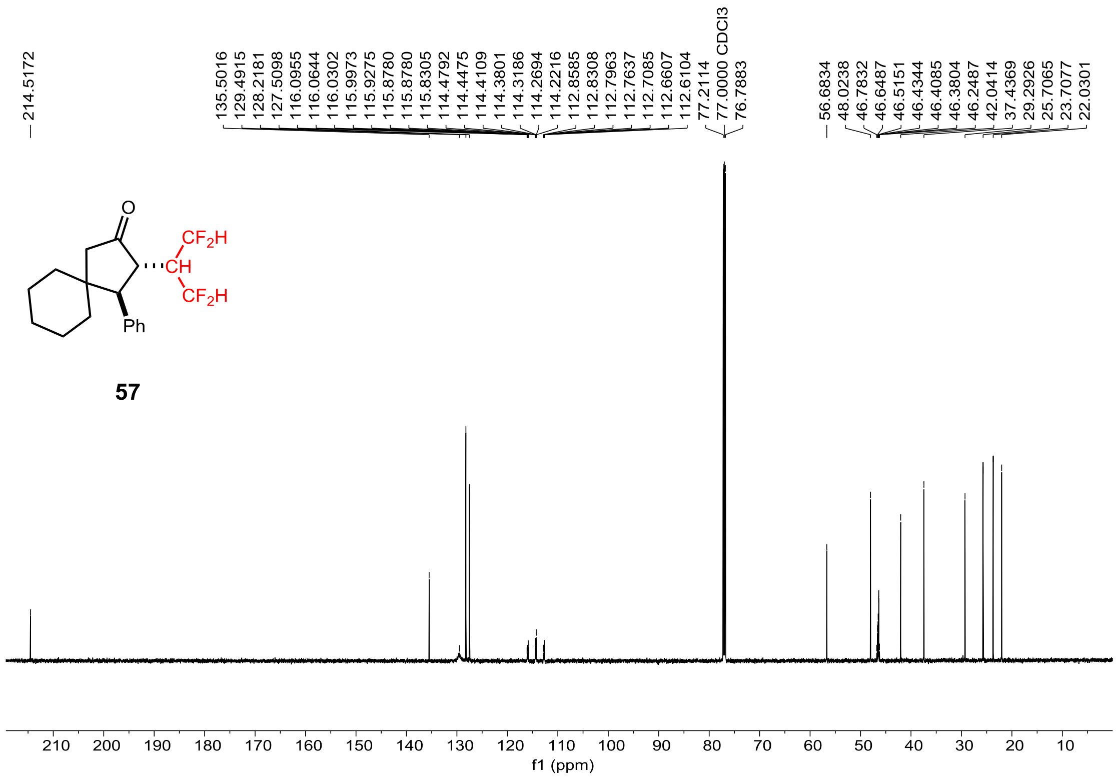

Supplementary Figure 159 ^{13}C NMR (151 MHz, $CDCl_3$) spectrum of compound 57.

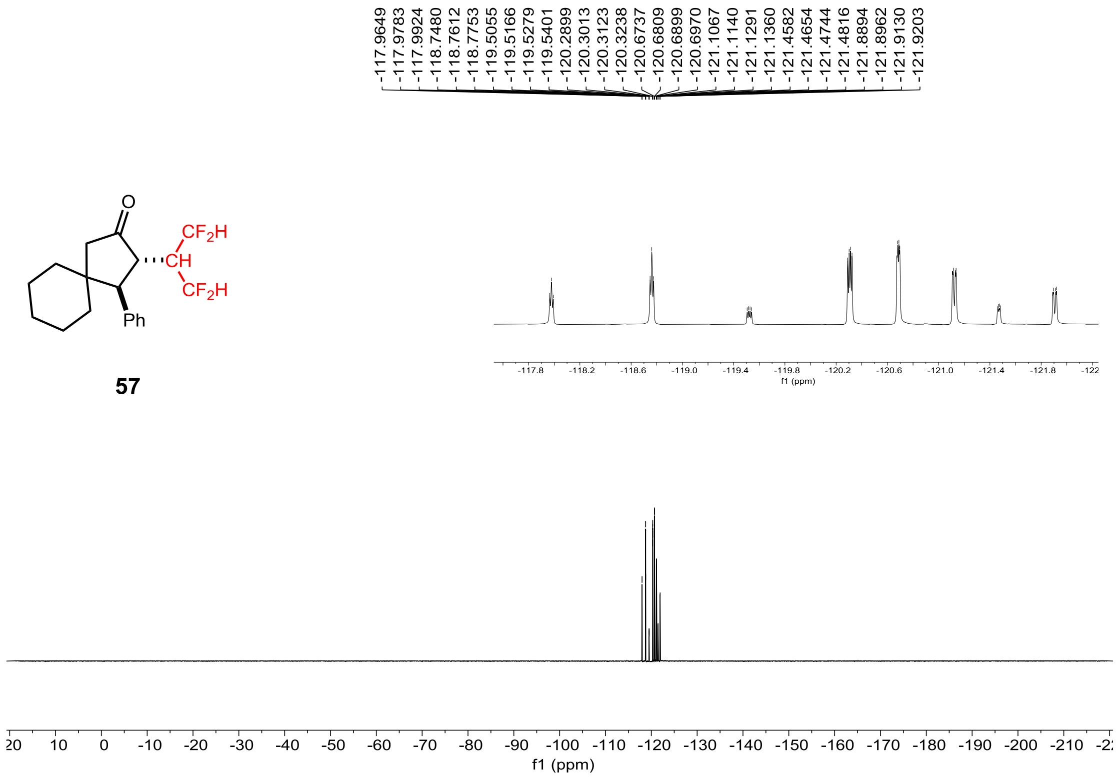

Supplementary Figure 160 ^{19}F NMR (377 MHz, $CDCl_3$) spectrum of compound 57.

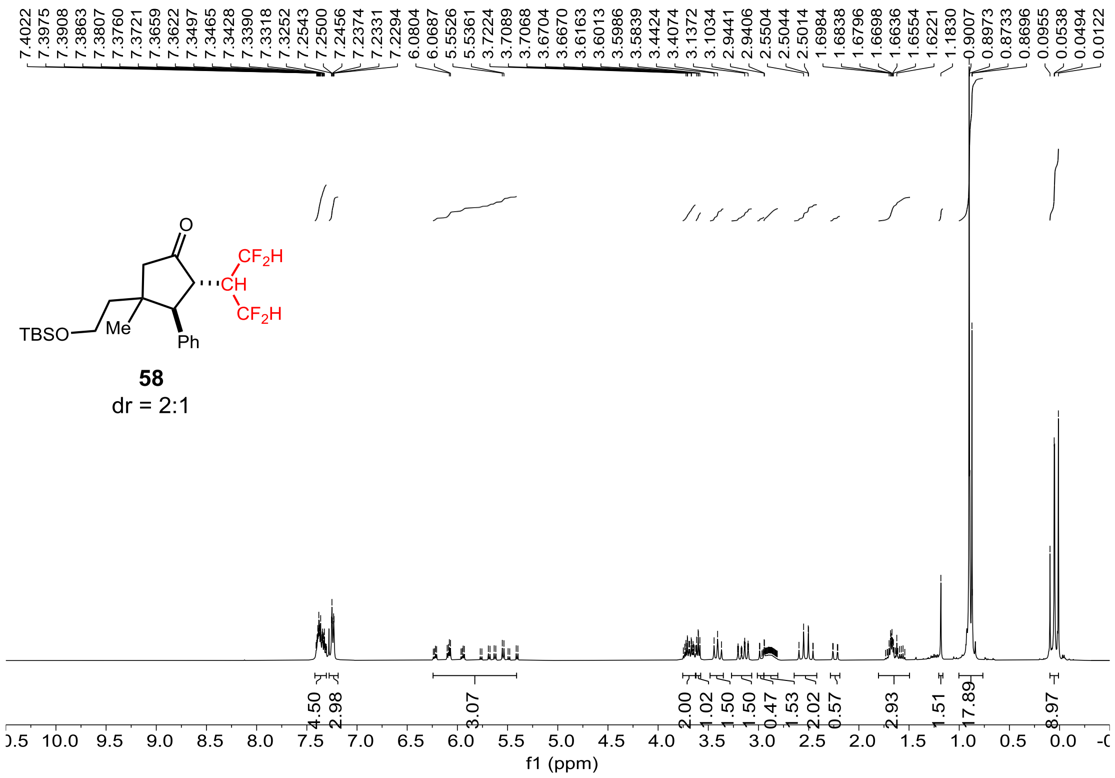

Supplementary Figure 161 ^{1}H NMR (400 MHz, $CDCl_3$) spectrum of compound 58.

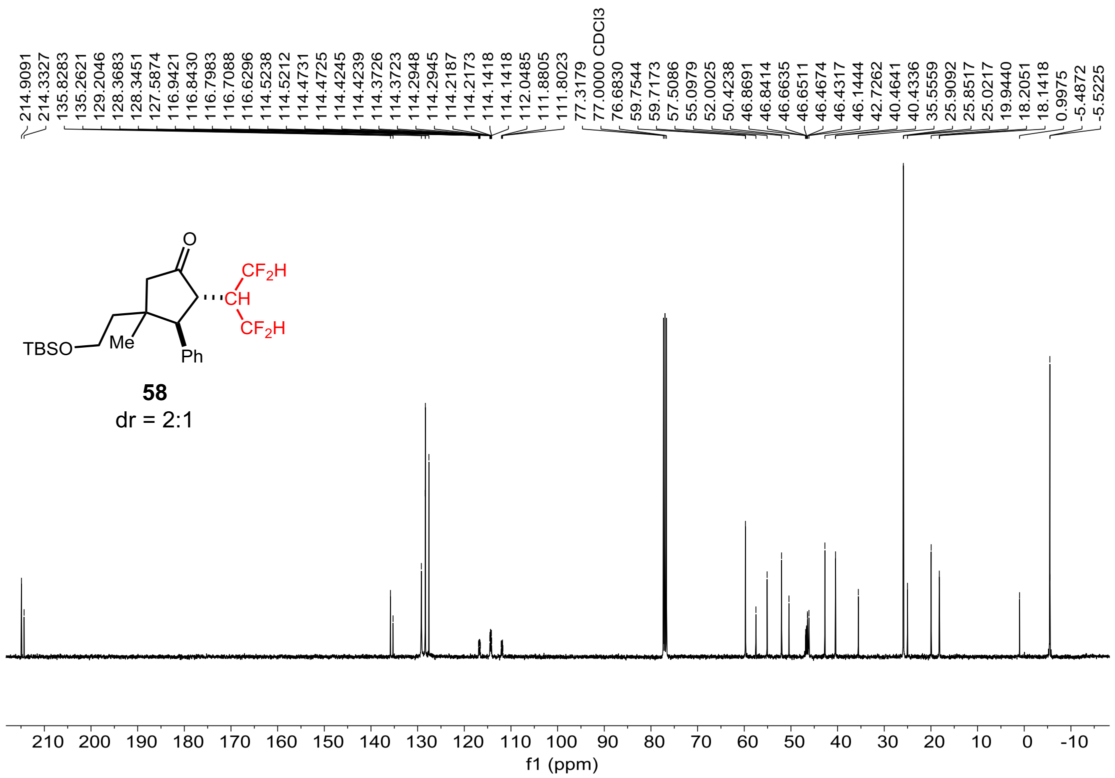

Supplementary Figure 162 ^{13}C NMR (151 MHz, $CDCl_3$) spectrum of compound 58.

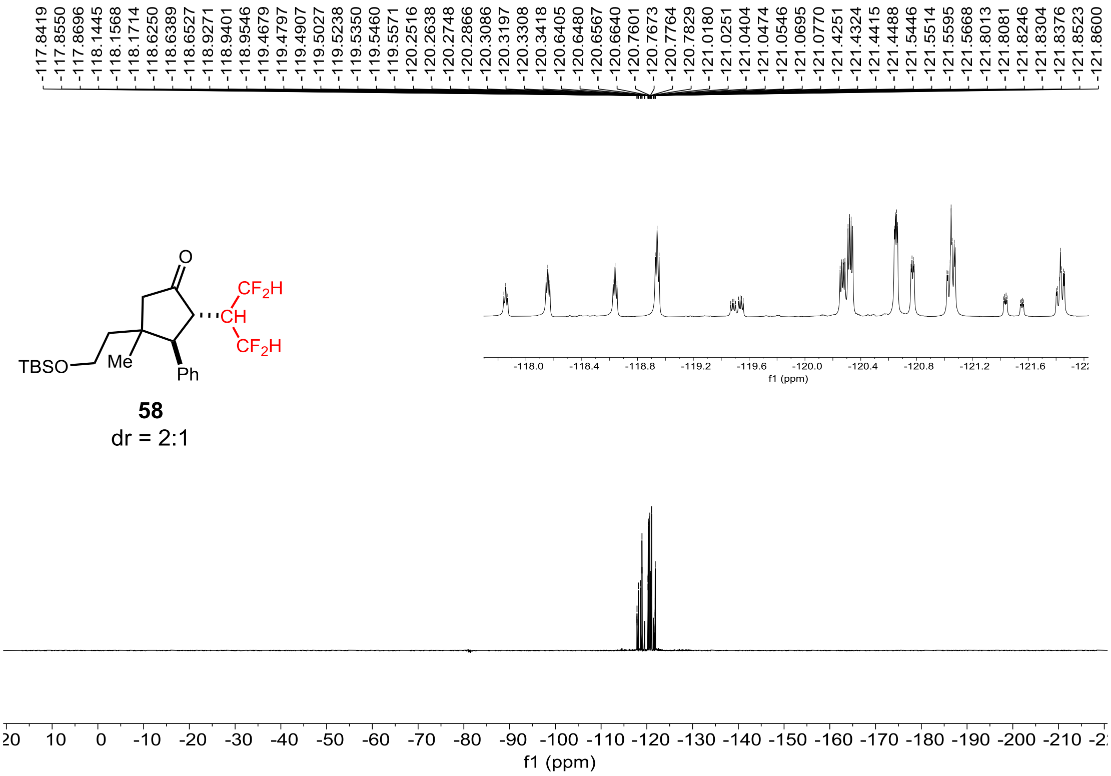

Supplementary Figure 163 ^{19}**F NMR (377 MHz, CDCl$_3$) spectrum of compound 58.**

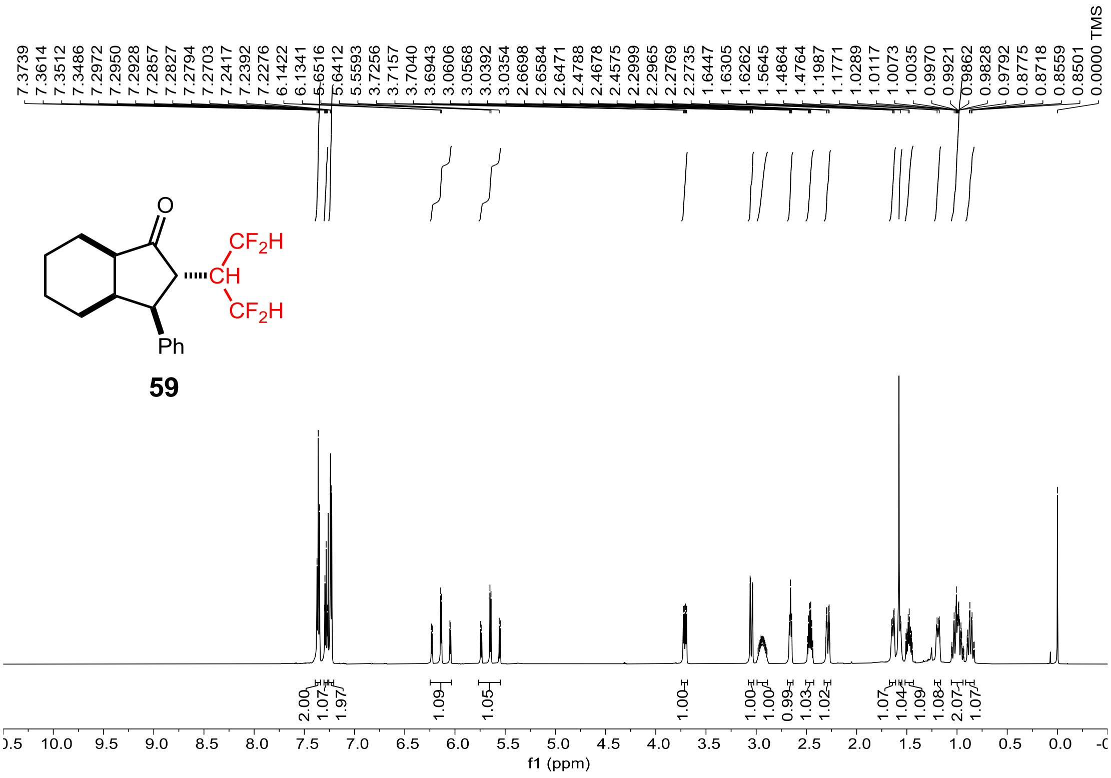

Supplementary Figure 164 ^{1}**H NMR (600 MHz, CDCl$_3$) spectrum of compound 59.**

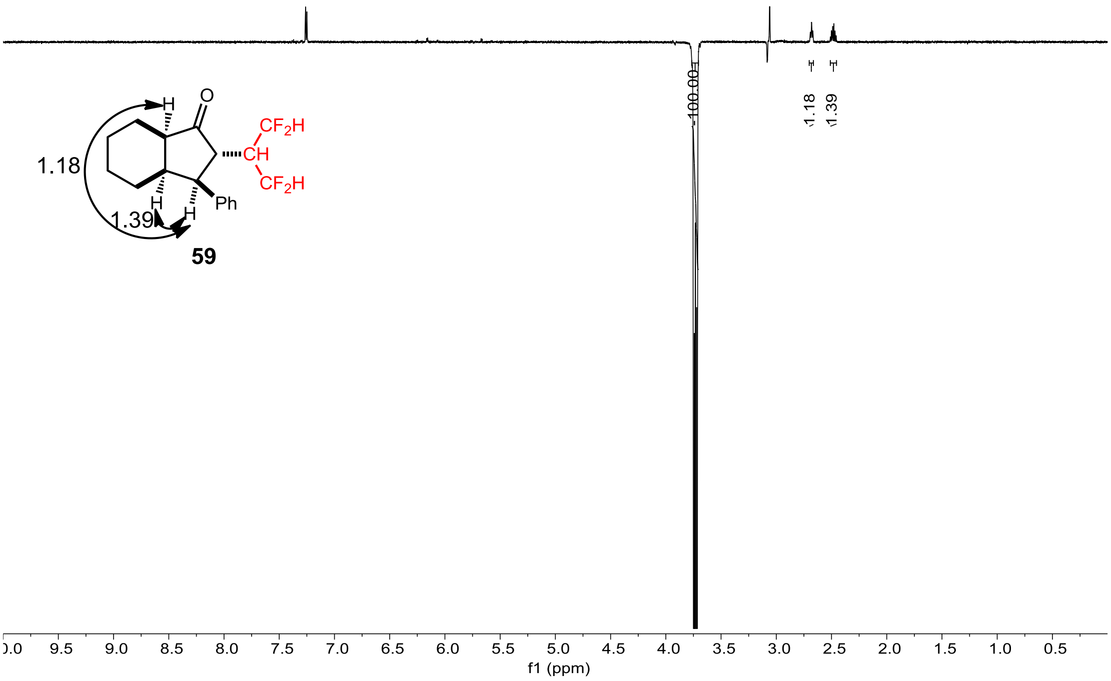

Supplementary Figure 165 NOE spectrum of compound 59.

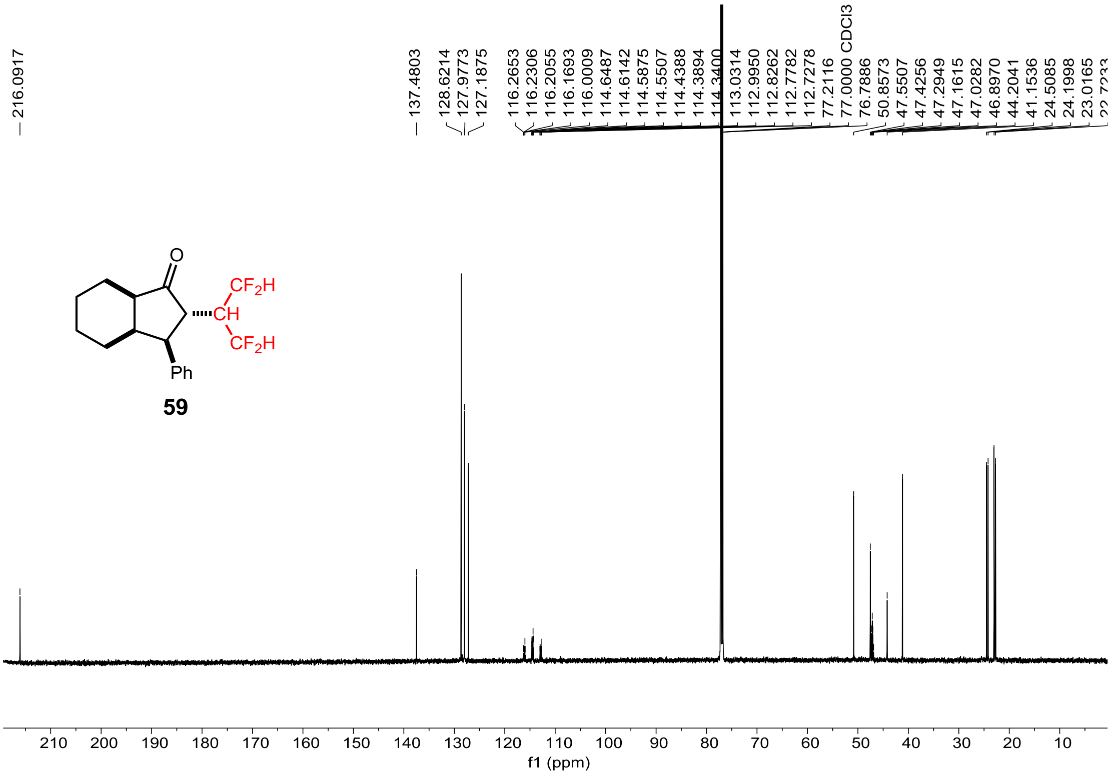

Supplementary Figure 166 ^{13}C NMR (151 MHz, $CDCl_3$) spectrum of compound 59.

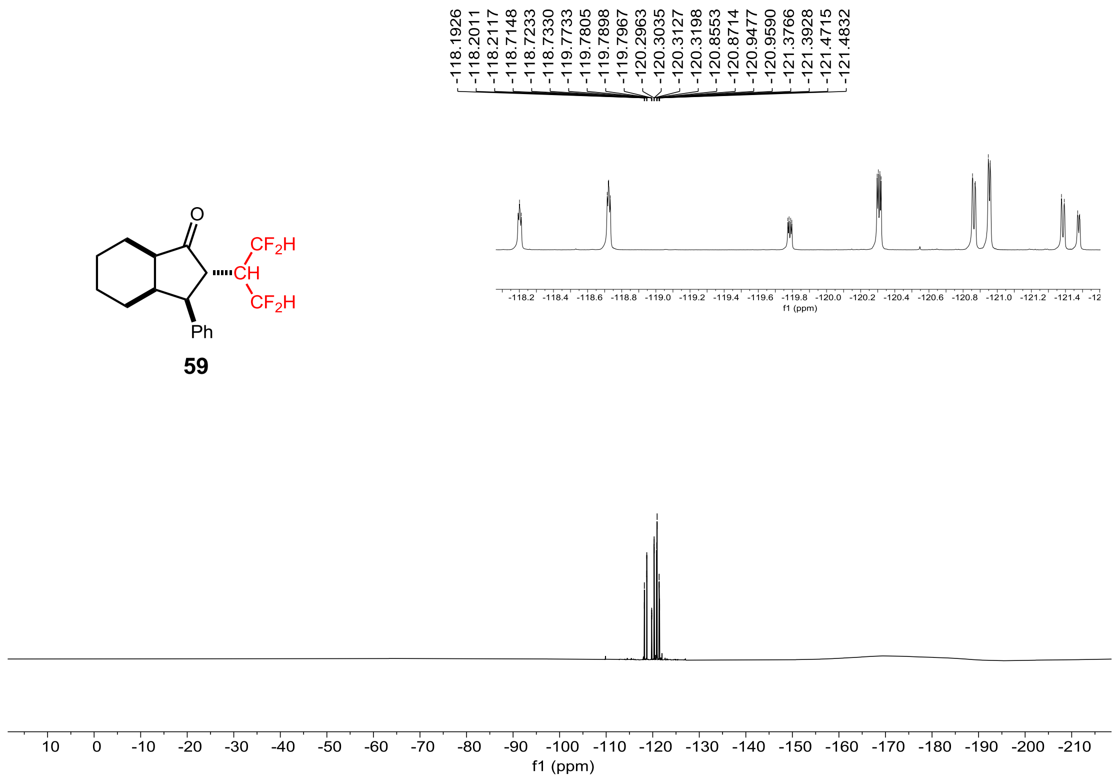

Supplementary Figure 167 ^{19}F NMR (377 MHz, $CDCl_3$) spectrum of compound 59.

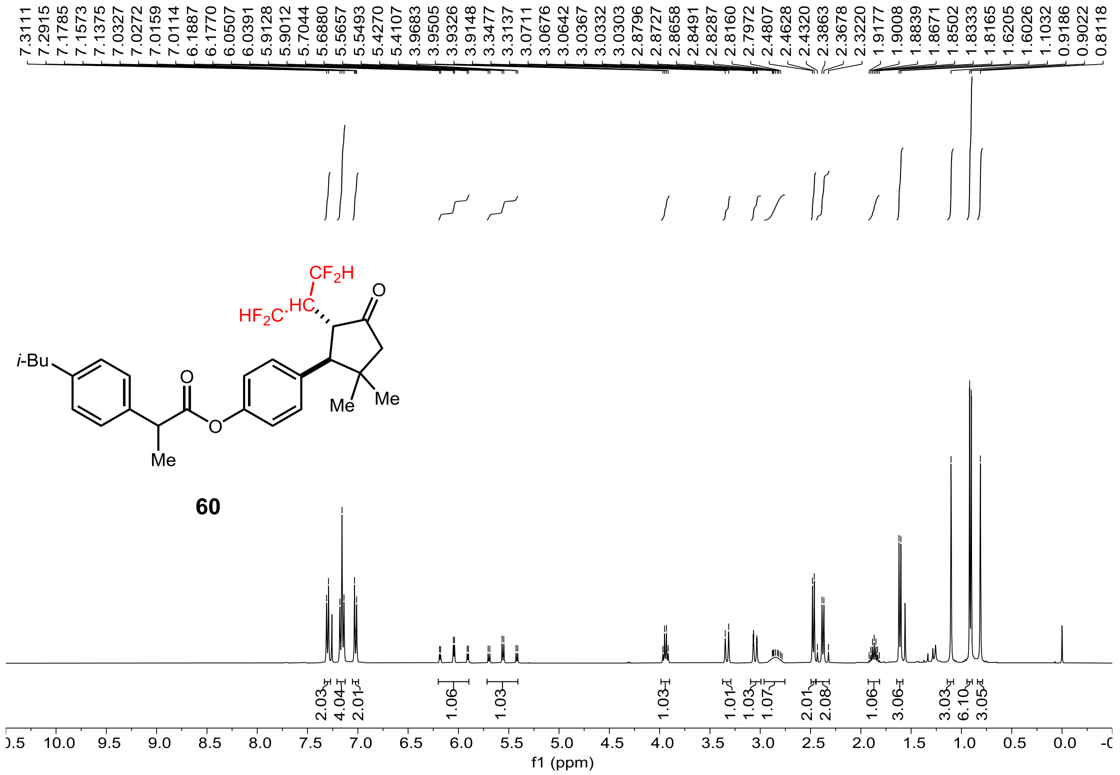

Supplementary Figure 168 ^{1}H NMR (400 MHz, $CDCl_3$) spectrum of compound 60.

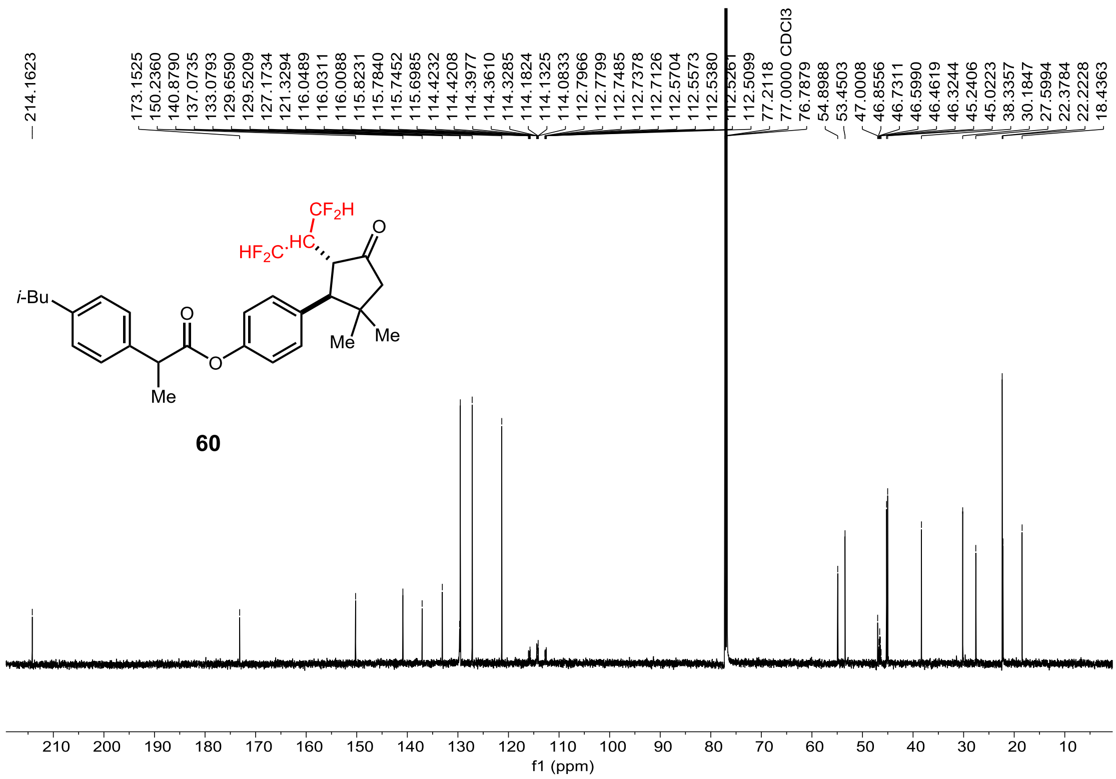

Supplementary Figure 169 ^{13}C NMR (151 MHz, CDCl$_3$) spectrum of compound 60.

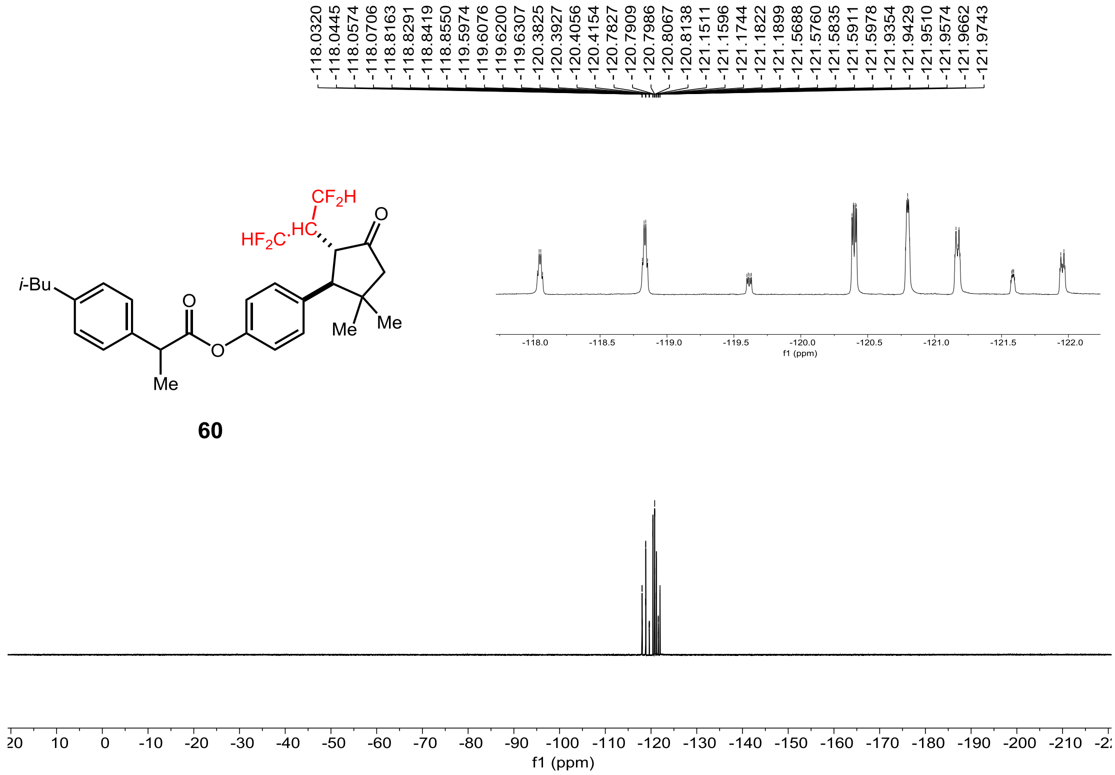

Supplementary Figure 170 ^{19}F NMR (377 MHz, CDCl$_3$) spectrum of compound 60.

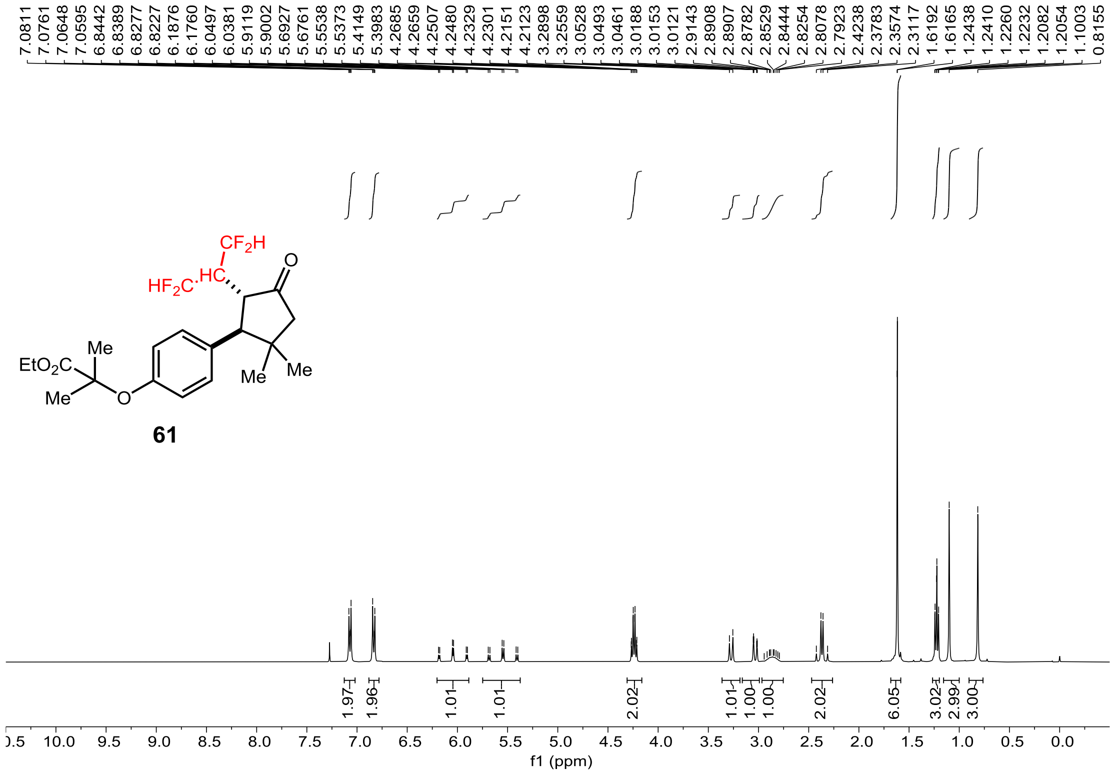

Supplementary Figure 171 ^{1}H NMR (400 MHz, $CDCl_3$) spectrum of compound 61.

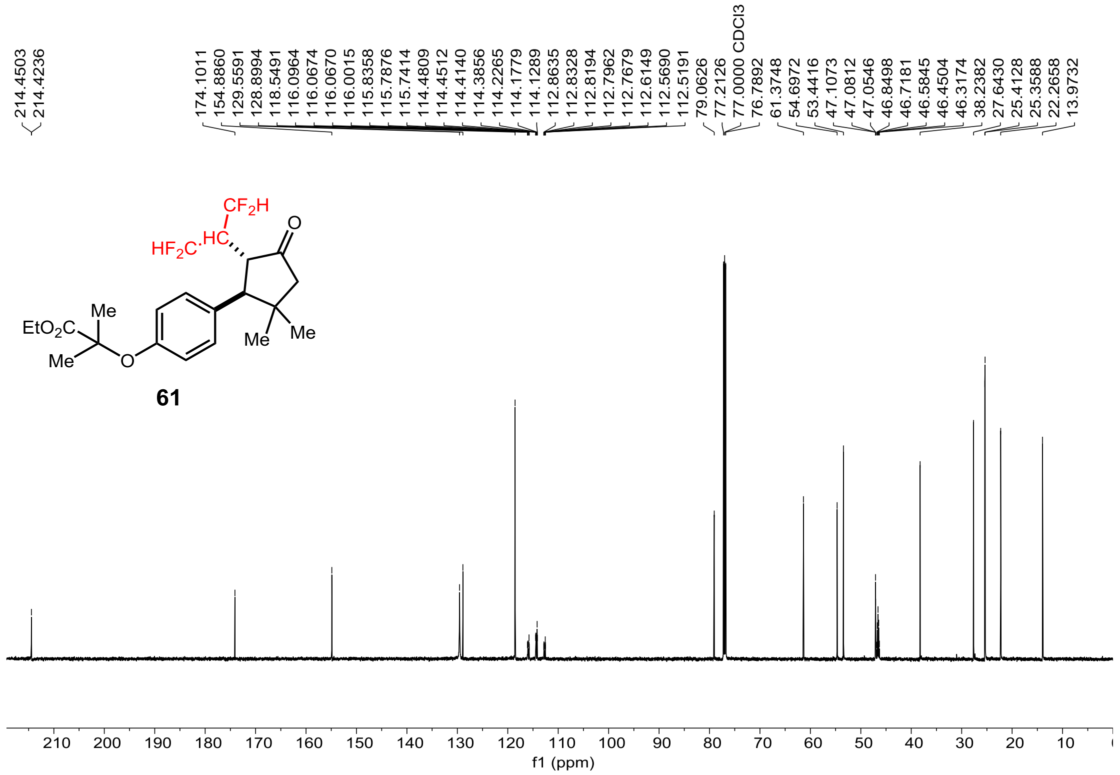

Supplementary Figure 172 ^{13}C NMR (151 MHz, $CDCl_3$) spectrum of compound 61.

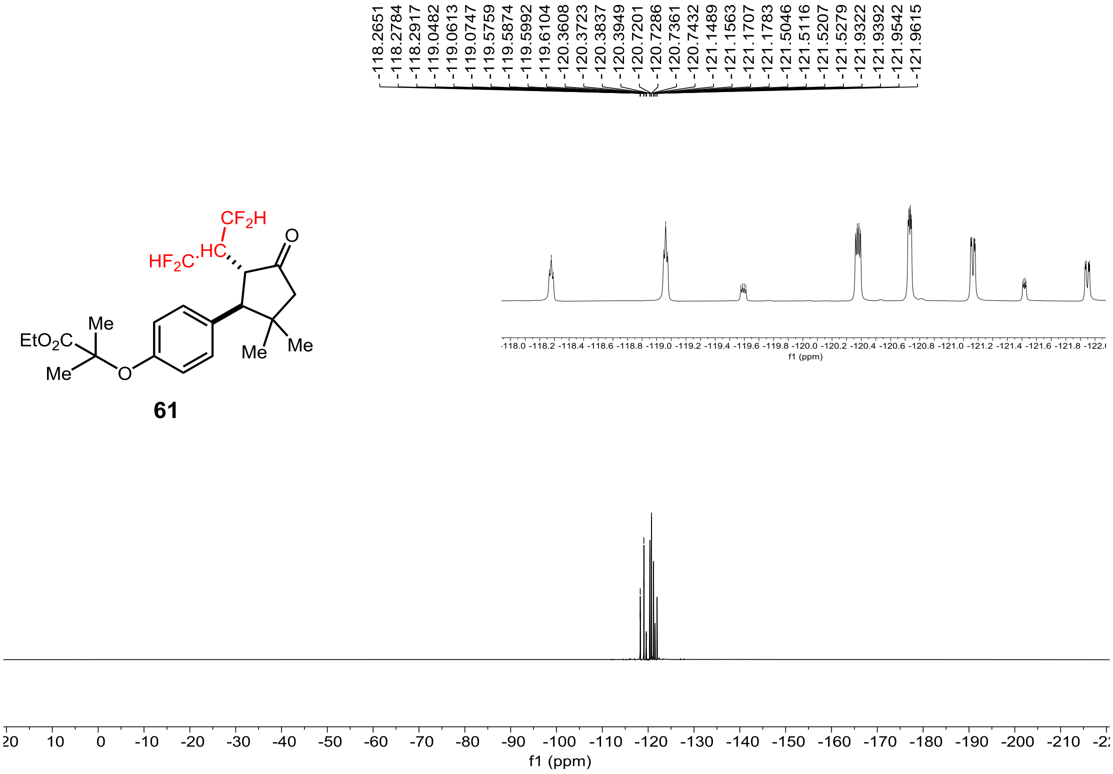

Supplementary Figure 173 ^{19}F NMR (377 MHz, $CDCl_3$) spectrum of compound 61.

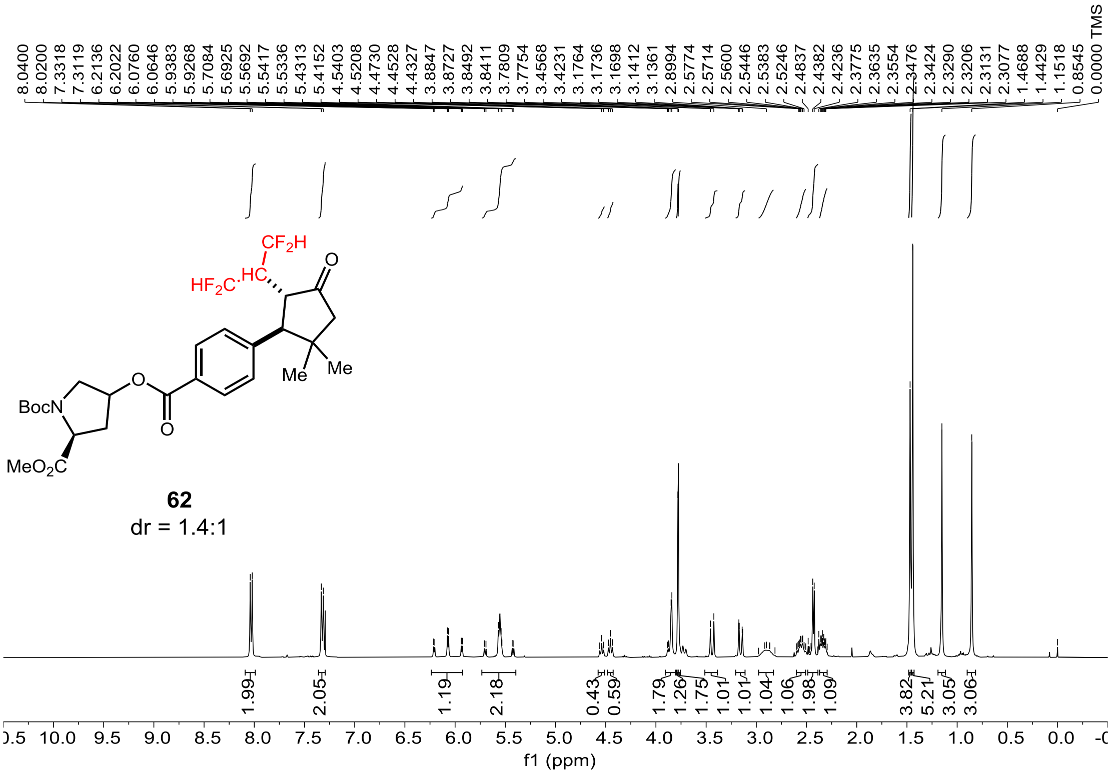

Supplementary Figure 174 ^{1}H NMR (400 MHz, $CDCl_3$) spectrum of compound 62.

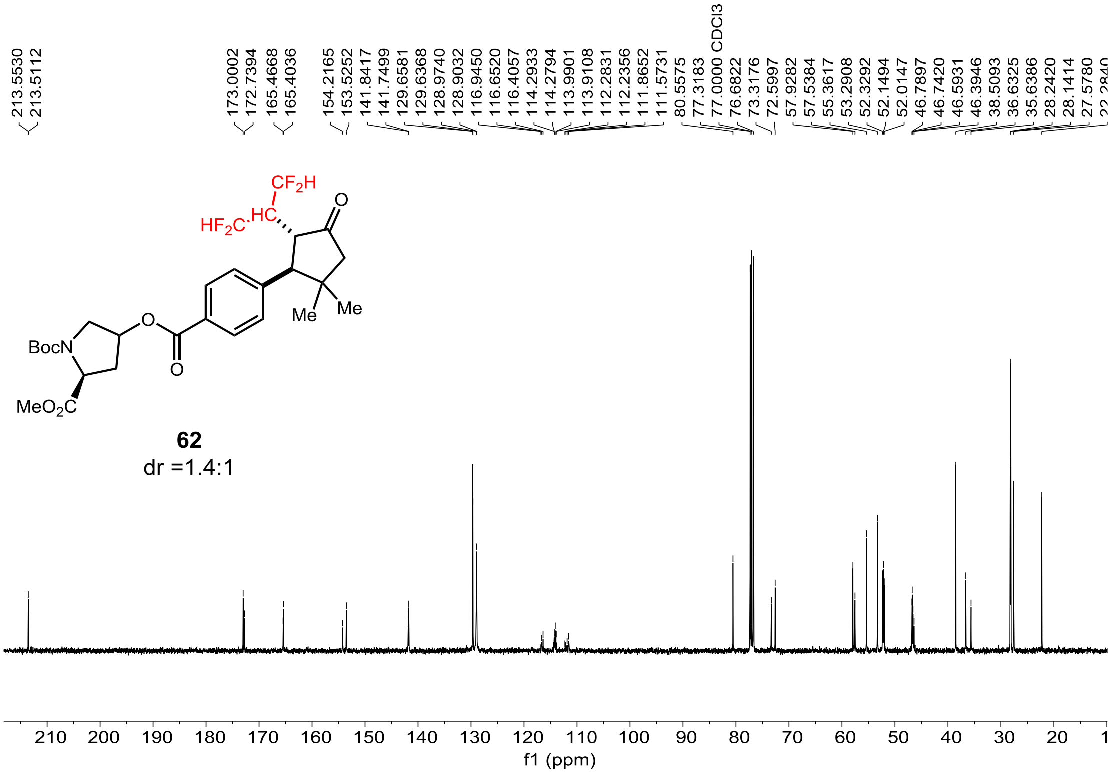

Supplementary Figure 175 ^{13}C NMR (101 MHz, $CDCl_3$) spectrum of compound 62.

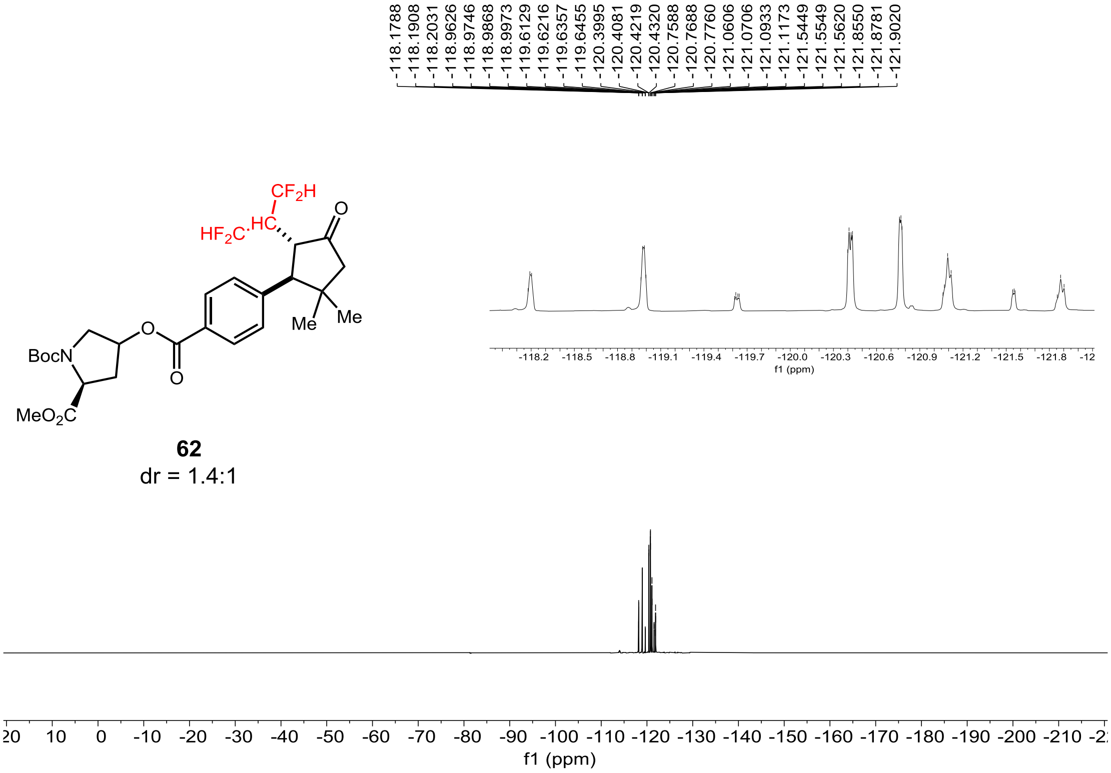

Supplementary Figure 176 ^{19}F NMR (377 MHz, $CDCl_3$) spectrum of compound 62.

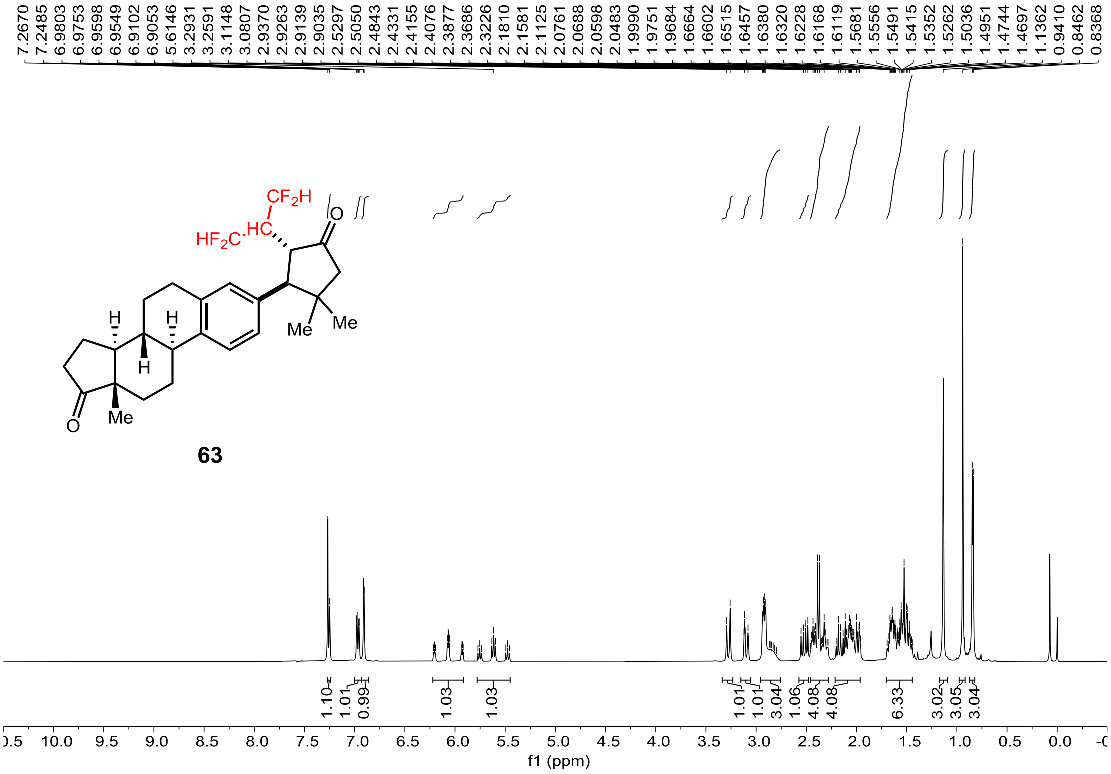

Supplementary Figure 177 ^{1}H NMR (400 MHz, $CDCl_3$) spectrum of compound 63.

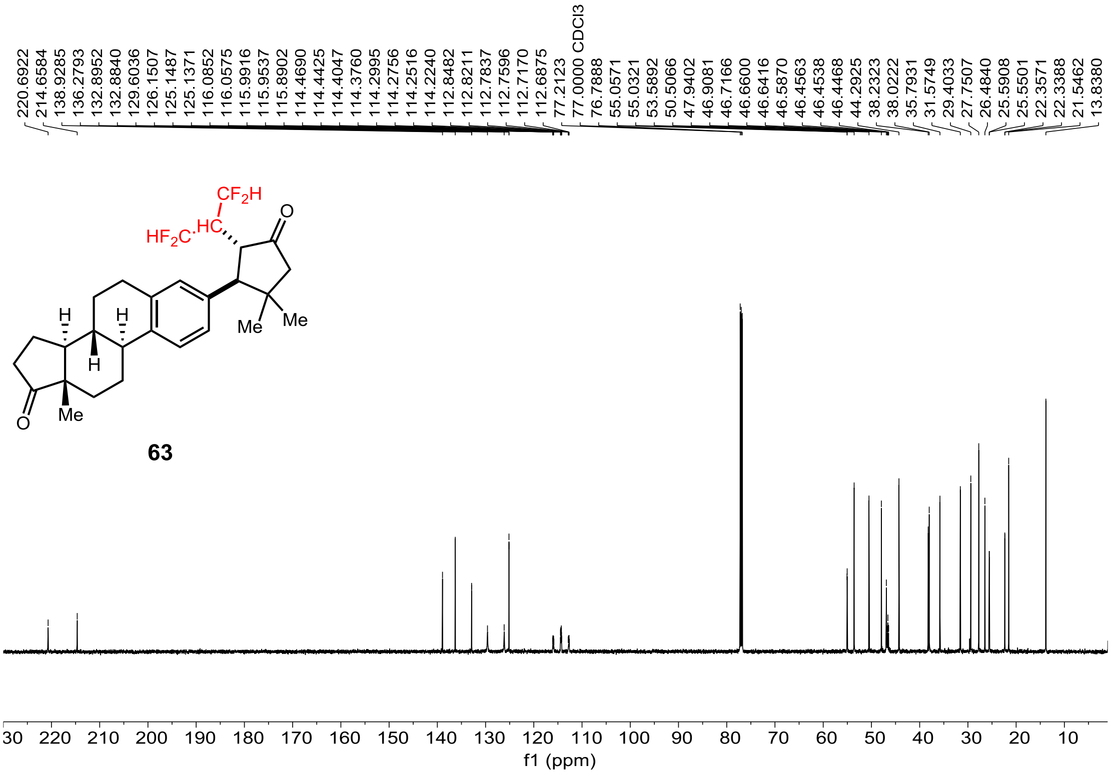

Supplementary Figure 178 ^{13}C NMR (151 MHz, $CDCl_3$) spectrum of compound 63.

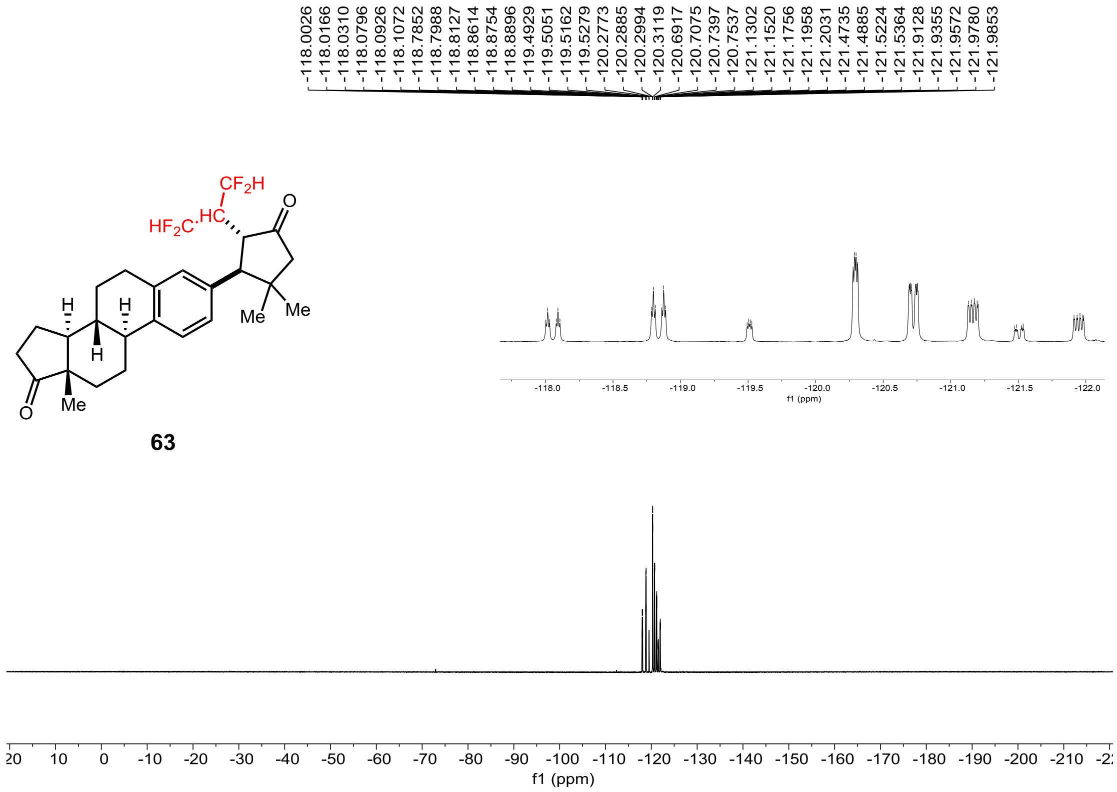

Supplementary Figure 179 ^{19}F NMR (377 MHz, $CDCl_3$) spectrum of compound 63.

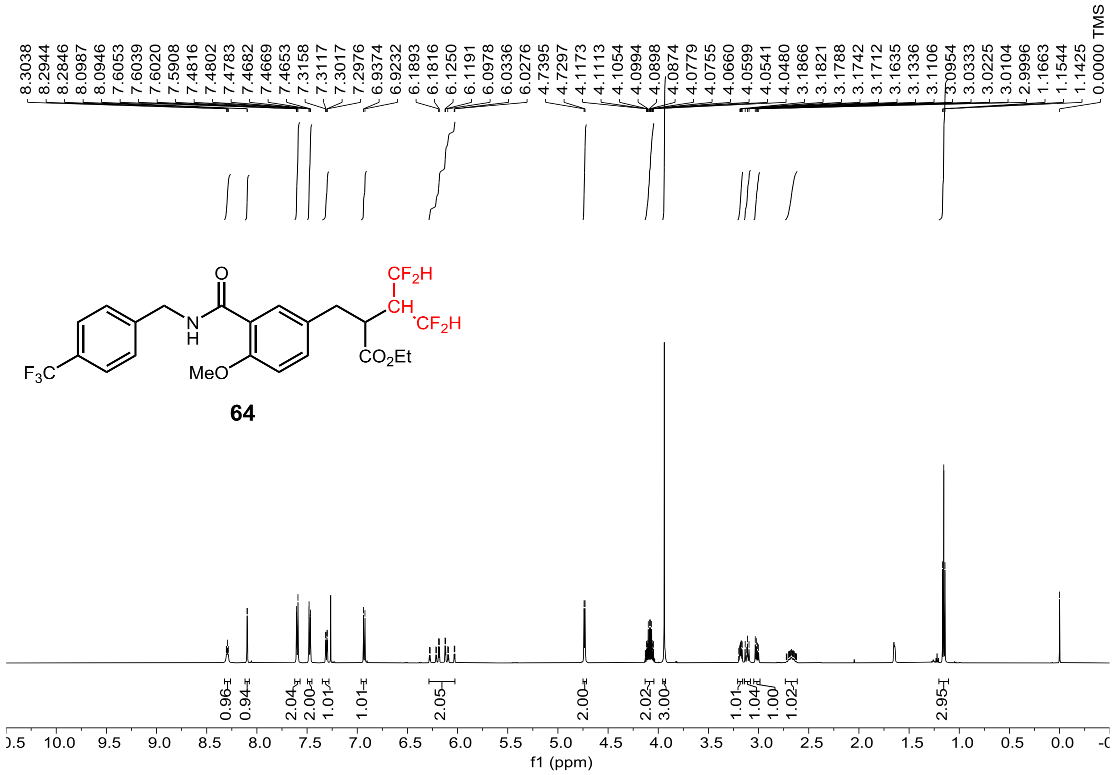

Supplementary Figure 180 ^{1}H NMR (600 MHz, $CDCl_3$) spectrum of compound 64.

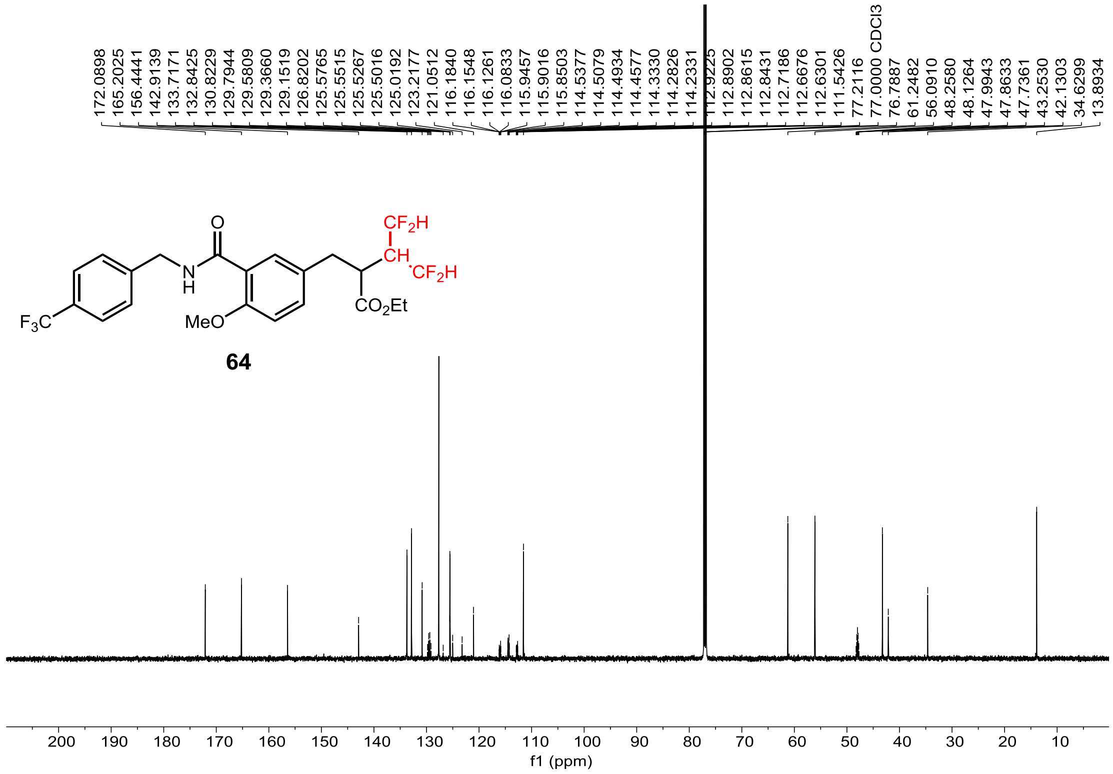

Supplementary Figure 181 ^{13}C NMR (151 MHz, $CDCl_3$) spectrum of compound 64.

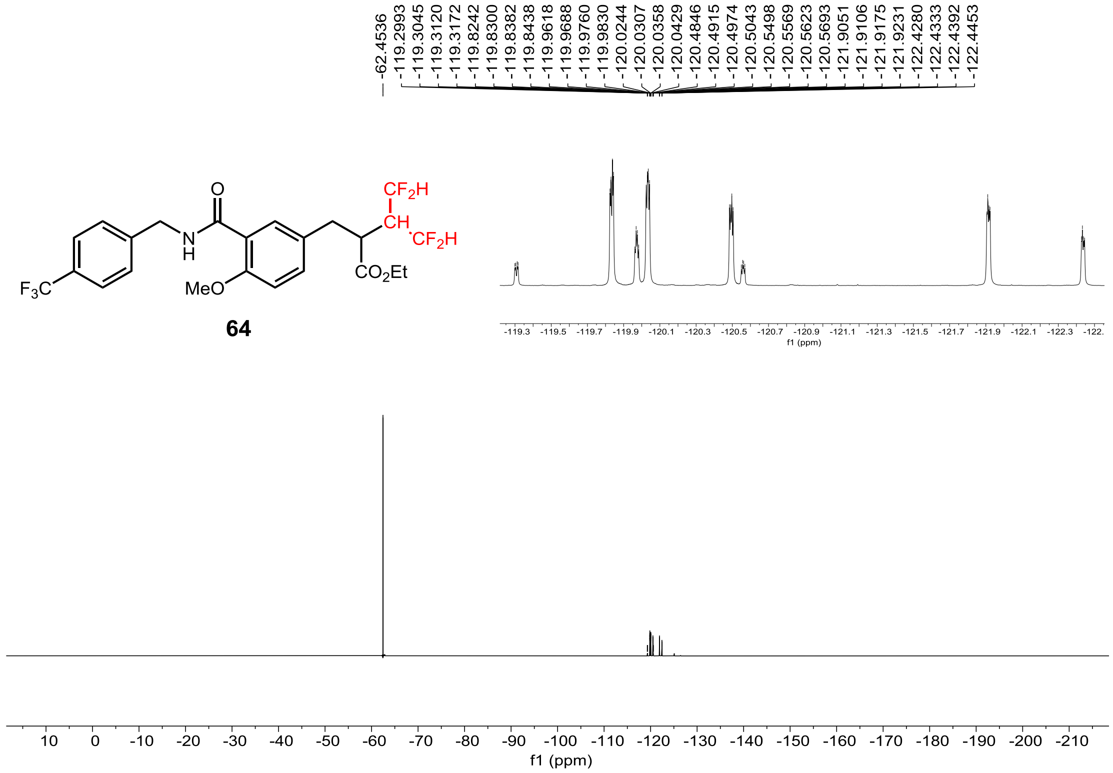

Supplementary Figure 182 ^{19}F NMR (565 MHz, $CDCl_3$) spectrum of compound 64.

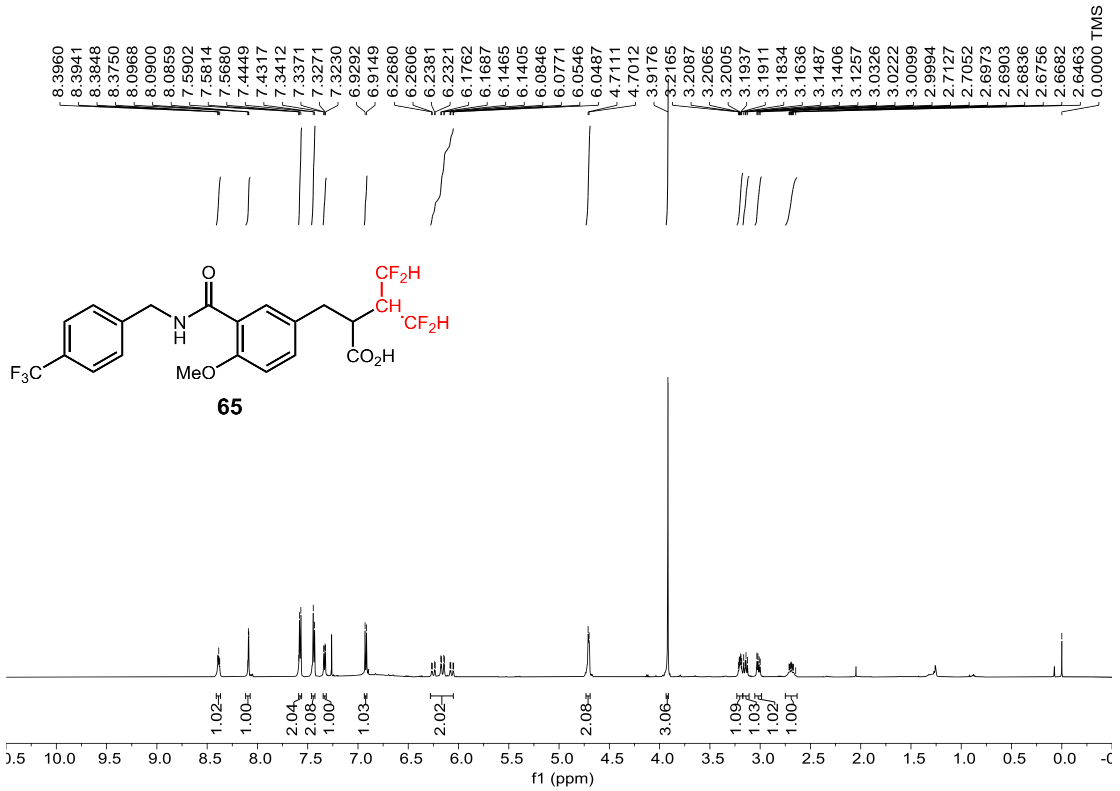

Supplementary Figure 183 ^{1}H NMR (600 MHz, $CDCl_3$) spectrum of compound 65.

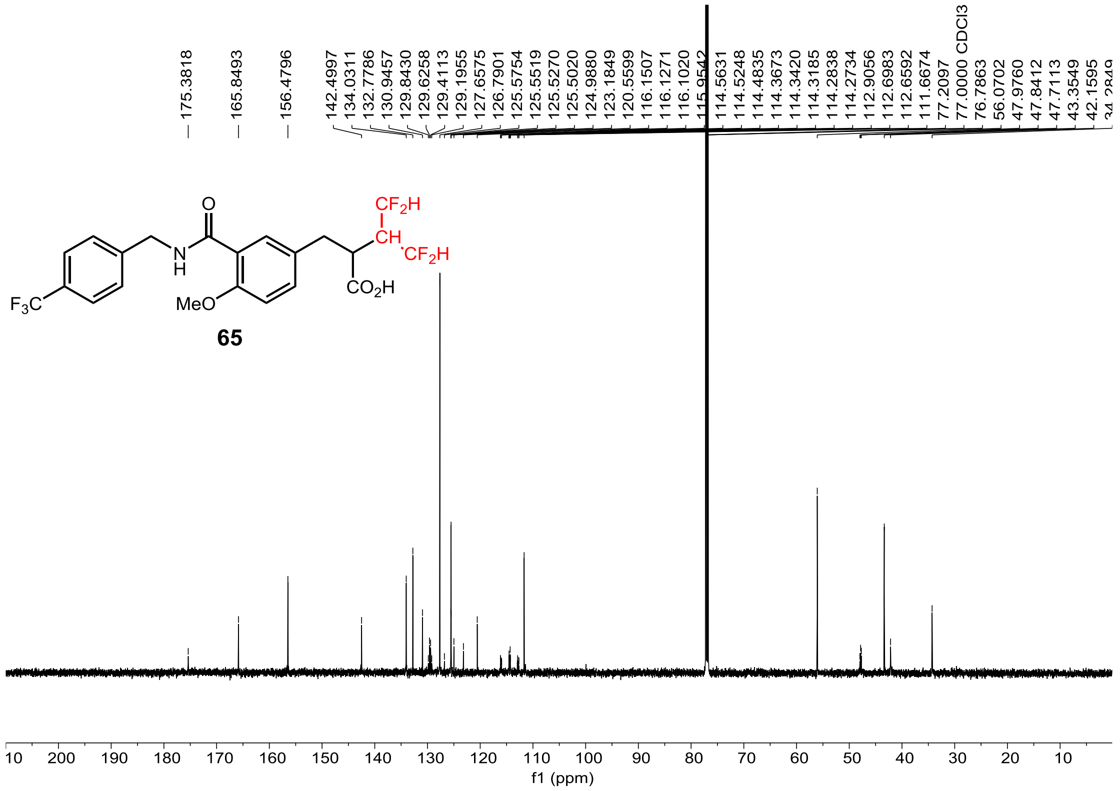

Supplementary Figure 184 ^{13}C NMR (151 MHz, $CDCl_3$) spectrum of compound 65.

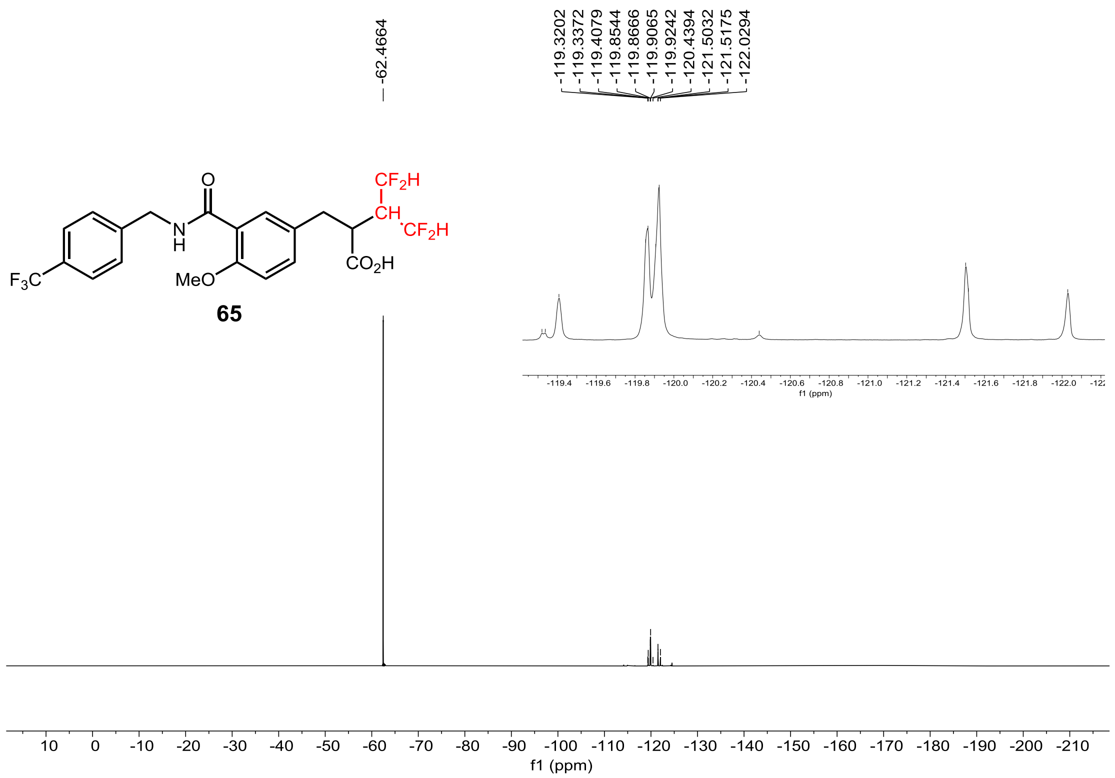

Supplementary Figure 185 ^{19}F NMR (565 MHz, $CDCl_3$) spectrum of compound 65.

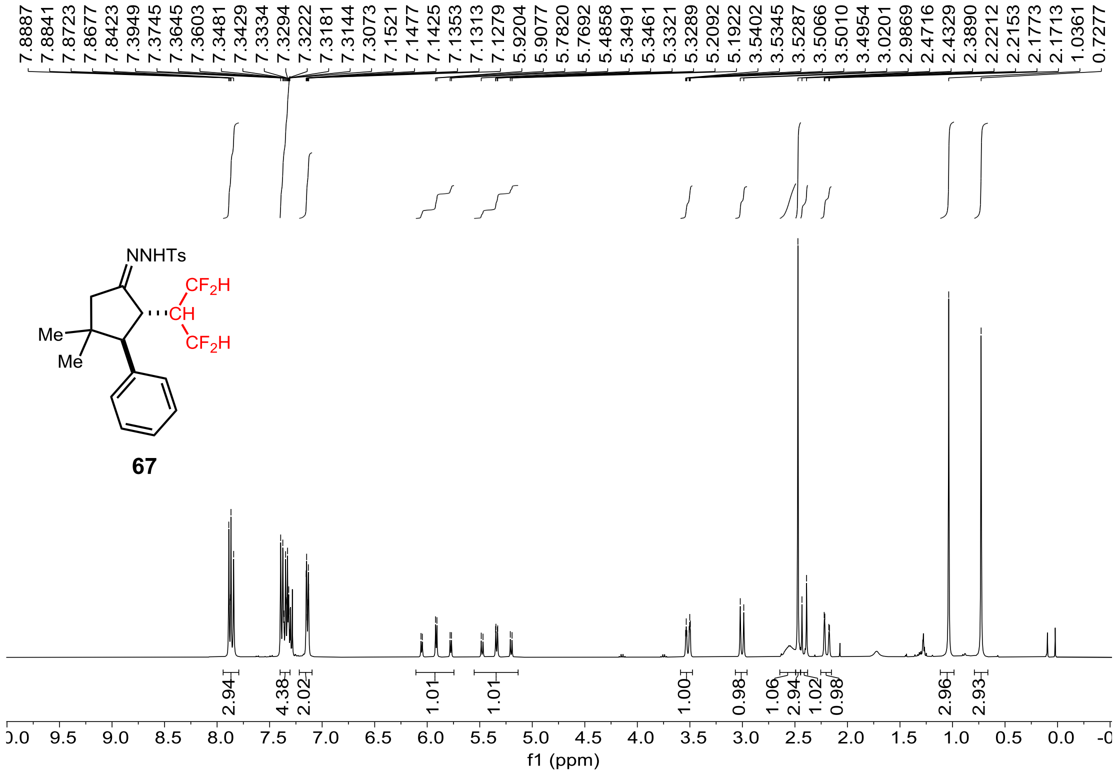

Supplementary Figure 186 ^{1}H NMR (400 MHz, $CDCl_3$) spectrum of compound 67.

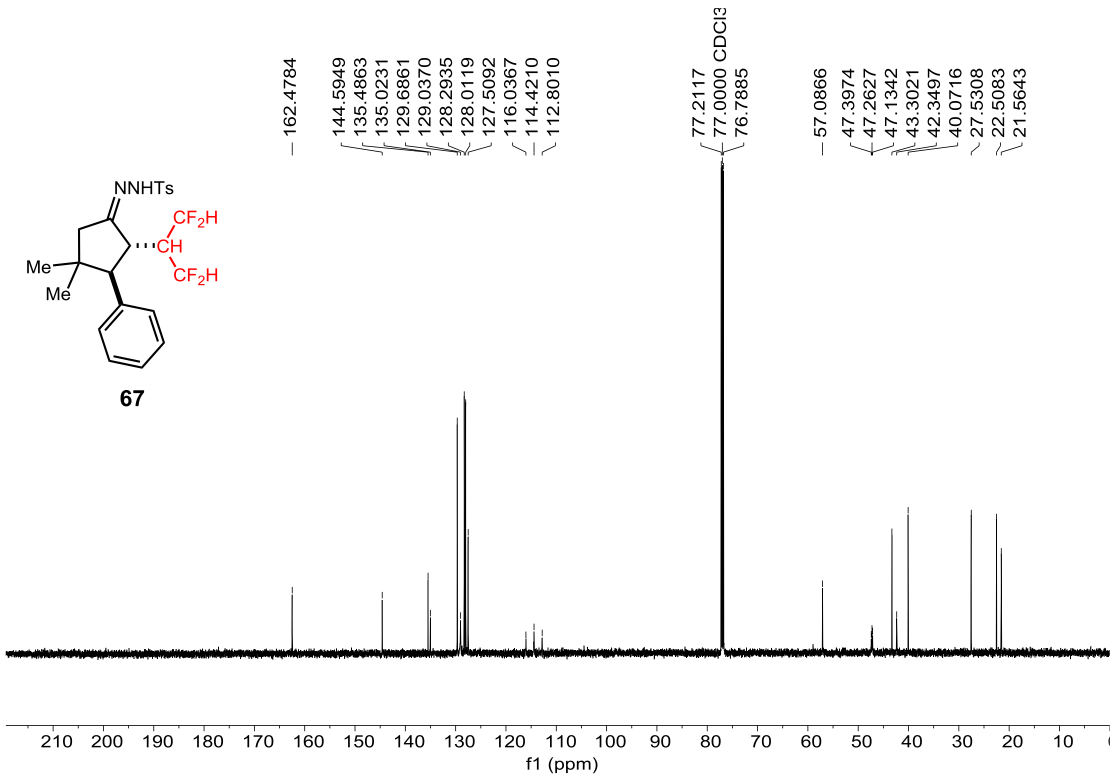

Supplementary Figure 187 ^{13}C NMR (151 MHz, CDCl$_3$) spectrum of compound 67.

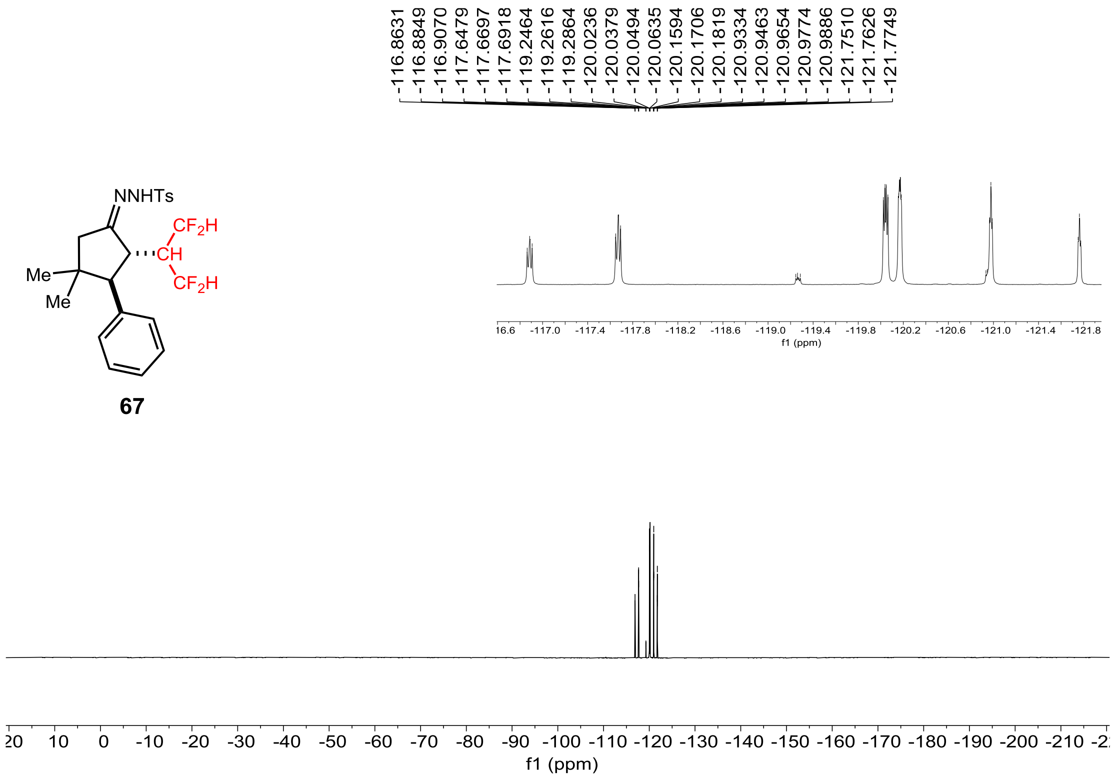

Supplementary Figure 188 ^{19}F NMR (377 MHz, CDCl$_3$) spectrum of compound 67.

8. X-Ray crystallographic data

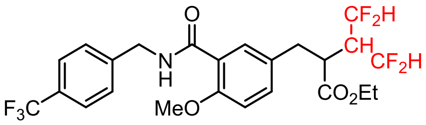

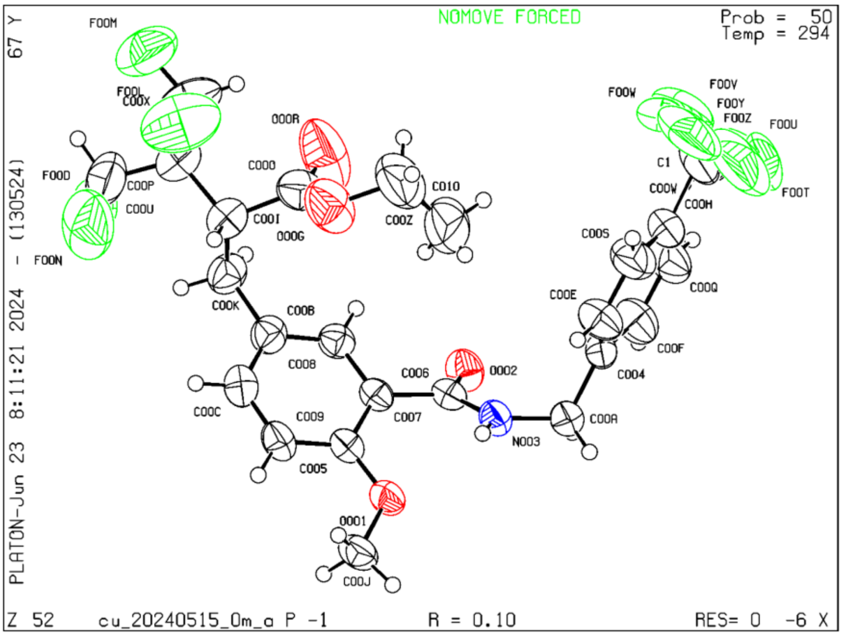

Supplementary Figure 189 Thermal ellipsoid plot at 50% probability for 64 (CCDC 2355597).

Supplementary Table 2 Crystal data and structure refinement for compound 64.

Chemical formula	$C_{24}H_{24}F_7NO_4$	
Formula weight	523.44	
Temperature	294.0 K	
Radiation type and wavelength	Cu*Kα* / 1.54178 Å	
Crystal system	Triclinic	
Space group	*P*-1	
Unit cell dimensions	a = 5.2059(2) Å	*a* = 70.921°(2)
	b = 13.7148(5) Å	*b* = 84.626°(2)
	c = 17.9467(6) Å	*g* = 84.574°(2)
V	1202.86(8) $Å^3$	
Z	2	
Density (calculated)	1.445 g/cm^3	
Absorption coefficient	1.172 mm^{-1}	
F (000)	540.0	
θ range for data collection	2.6° to 68.4°	
Reflections collected	13928	
Independent reflections	4372 R(int) = 0.0347	
Refinement method	Full-matrix least-squares on F^2	
Data / restraints / parameters	4372 / 115 / 364	
Goodness-of-fit on F^2	1.594	
Final R indices [I>2σ(I)]	R_1 = 0.0980, wR_2 = 0.3294	
R indices (all data)	R_1 = 0.1035, wR_2 = 0.3449	
Largest diff. peak and hole	0.722 and -0.536 e.$Å^{-3}$	

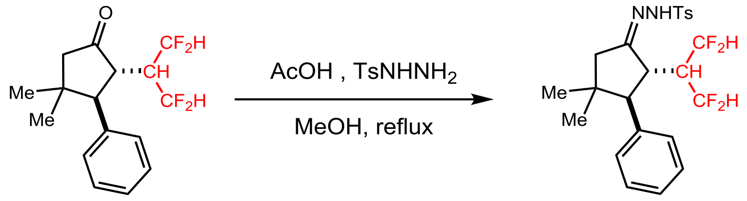

42 **67**

To a solution of **42** (30.0 mg, 0.1 mmol) in 1 mL of dry MeOH was added $TsNHNH_2$ (22.3 mg, 0.12 mmol) and HOAc (1.2 mg, 0.02 mmol) at room temperature. After refluxing for 18 h, the reaction mixture was quenched with water, extracted with EtOAc, dried over anhydrous Na_2SO_4, and concentrated. Column chromatography on silica gel (PE/EtOAc = 5:1) gave 37 mg (78% yield) of **67** as a white solid, mp 168–176 °C, dr > 20:1. ^{1}H NMR (400 MHz, $CDCl_3$) δ 7.94–7.79 (m, 3H), 7.40–7.31 (m, 4H), 7.22–7.10 (m, 2H), 5.91 (td, J = 55.4, 5.1 Hz, 1H), 5.34 (td, J = 55.9, 6.8 Hz, 1H), 3.52 (dt, J = 13.3, 2.3 Hz, 1H), 3.00 (d, J = 13.3 Hz, 1H), 2.64–2.49 (m, 1H), 2.47 (s, 3H), 2.41 (d, J = 17.6 Hz, 1H), 2.20 (dd, J = 17.6, 2.4 Hz, 1H), 1.04 (s, 3H), 0.73 (s, 3H); ^{13}C NMR (151 MHz, $CDCl_3$) δ 162.5, 144.6, 135.5, 135.0, 129.7, 129.0, 128.3, 128.0, 127.5, 116.0–112.5 (m), 116.2–112.8 (m), 57.1, 47.2 (quintet, J = 20.2 Hz), 43.3, 42.3, 40.1, 27.5, 22.5, 21.6; ^{19}F NMR (377 MHz, $CDCl_3$) δ -116.7 – -121.8 (m), -119.2 – -121.0 (m); HRMS (ESI): Calcd for $C_{23}H_{26}F_4N_2O_2S$ $[M + Na]^+$ 493.1543; Found: 493.1543.

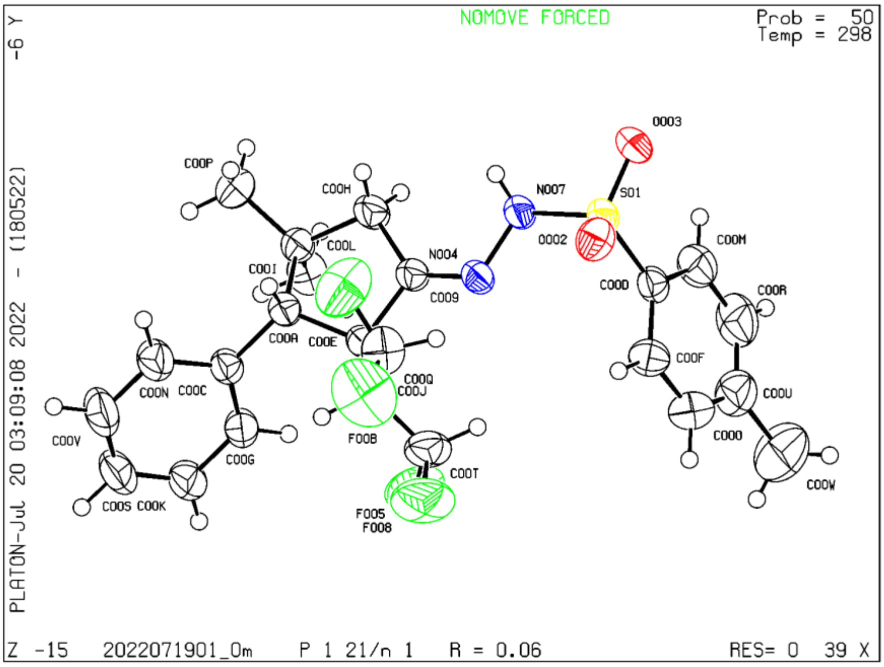

Supplementary Figure 190 Thermal ellipsoid plot at 50% probability for 67 (CCDC 2323408).

Supplementary Table 3 Crystal data and structure refinement for compound 67.

Chemical formula	$C_{23}H_{26}F_4N_2O_2S$	
Formula weight	470.52	
Temperature	298 K	
Radiation type and wavelength	Mo$K\alpha$ / 0.71073 Å	
Crystal system	monoclinic	
Space group	$P2_1/n$ (no. 14)	
Unit cell dimensions	a = 15.292(8) Å	α = 90 °
	b = 9.862(4) Å	β = 105.725 °(2)
	c = 16.105(9) Å	γ = 90 °
V	2337.8(2) Å^3	
Z	4	
Density (calculated)	1.337 mg/m^3	
Absorption coefficient	0.192 mm^{-1}	
F (000)	984	
θ range for data collection	2.5 °to 27.1 °	
Reflections collected	5921	
Independent reflections	5147 R(int) = 0.0505	
Refinement method	Full-matrix least-squares on F^2	
Data / restraints / parameters	5147 / 0 / 292	
Goodness-of-fit on F^2	1.056	
Final R indices [I>2σ(I)]	R_1 = 0.0581, wR_2 = 0.1563	
R indices (all data)	R_1 = 0.0809, wR_2 = 0.1749	
Largest diff. peak and hole	0.531 and -0.482 e.Å^{-3}	

9. Supplementary references

1. (a) Einaru, S., Shitamichi, K., Nagano, T., Matsumoto, A., Asano, K. & Matsubara, S. *trans*-Cyclooctenes as halolactonization catalysts. *Angew. Chem. Int. Ed.* **57**, 13863–13867 (2018). (b) Rivero-Crespo, M. A., Toupalas, G. & Morandi, B. Preparation of recyclable and versatile porous poly(aryl thioether)s by reversible Pd-catalyzed C–S/C–S metathesis. *J. Am. Chem. Soc.* **143**, 21331–21339 (2021).

2. (a) Wang, Q. & May, J. A. Formation of β-oxo-*N*-vinylimidates via intermolecular ester incorporation in huisgen cyclization/carbene cascade reactions. *Org. Lett.* **22**, 9579–9584 (2020). (b) Reddy, T. P., Gujral, J., Roy, P. & Ramachary, D. B. Catalytic ynone–amidine formal [4 + 2]-cycloaddition for the regioselective synthesis of tricyclic azepines. *Org. Lett.* **22**, 9653–9657 (2020).

3. Rong, J., Deng, L., Tan, P., Ni, C., Gu, Y. & Hu, J. Radical fluoroalkylation of isocyanides with fluorinated sulfones by visible-light photoredox catalysis. *Angew. Chem. Int. Ed*. **55**, 2743–2747 (2016).

4. Ma, Q., Liu, C. & Tsui, G. C. Palladium-catalyzed stereoselective hydrodefluorination of tetrasubstituted *gem*-difluoroalkenes. *Org. Lett.* **22**, 5193–5197 (2020).

5. Miyachi, H., Nomura, M., Tanase, T., Takahashi, Y., Ide, T., Tsunoda, M. & Murakami, K., Awano,

K. Design, synthesis and evaluation of substituted phenylpropanoic acid derivatives as peroxisome proliferator-activated receptor (PPAR) activators: novel human PPARα-selective activators. *Bioorg. Med. Chem. Lett.* **12**, 77–80 (2002).
